# Theranostic Fluorescent Probes

**DOI:** 10.1021/acs.chemrev.3c00778

**Published:** 2024-02-29

**Authors:** Amit Sharma, Peter Verwilst, Mingle Li, Dandan Ma, Nem Singh, Jiyoung Yoo, Yujin Kim, Ying Yang, Jing-Hui Zhu, Haiqiao Huang, Xi-Le Hu, Xiao-Peng He, Lintao Zeng, Tony D. James, Xiaojun Peng, Jonathan L. Sessler, Jong Seung Kim

**Affiliations:** † Amity School of Chemical Sciences, Amity University Punjab, Sector 82A, Mohali 140 306, India; ‡ Rega Institute for Medical Research, Medicinal Chemistry, KU Leuven, Herestraat 49, Box 1041, 3000 Leuven, Belgium; § College of Materials Science and Engineering, Shenzhen University, Shenzhen 518060, China; ∥ College of Physics and Optoelectronic Engineering, Shenzhen University, Shenzhen 518060, China; ⊥ Department of Chemistry, Korea University, Seoul 02841, Korea; # School of Light Industry and Food Engineering, Guangxi University, Nanning, Guangxi 530004, China; ⊗ Key Laboratory for Advanced Materials and Joint International Research Laboratory of Precision Chemistry and Molecular Engineering, Feringa Nobel Prize Scientist Joint Research Center, School of Chemistry and Molecular Engineering, East China University of Science and Technology, 130 Meilong Road, Shanghai 200237, China; ¶ National Center for Liver Cancer, the International Cooperation Laboratory on Signal Transduction, Eastern Hepatobiliary Surgery Hospital, Shanghai 200438, China; $ Department of Chemistry, University of Bath, Bath BA2 7AY, United Kingdom; ▼ School of Chemistry and Chemical Engineering, Henan Normal University, Xinxiang 453007, China; ▲ State Key Laboratory of Fine Chemicals, Dalian University of Technology, Dalian 116024, China; ○ Department of Chemistry, The University of Texas at Austin, Texas 78712-1224, United States; ● TheranoChem Incorporation, Seongbuk-gu, Seoul 02841, Korea

## Abstract

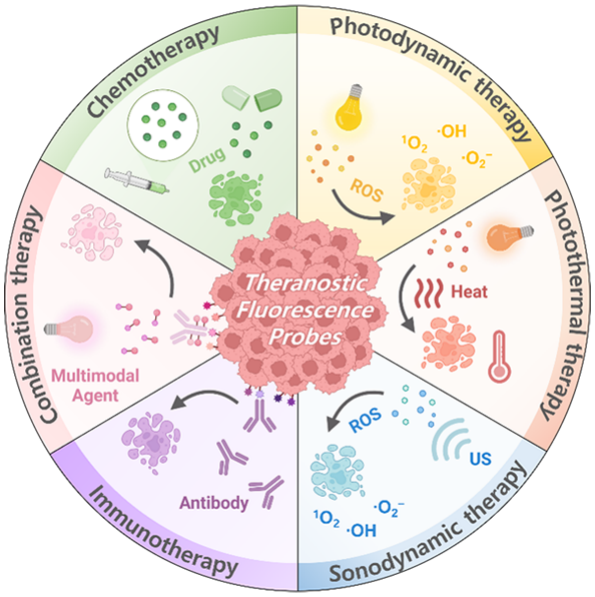

The ability to gain spatiotemporal information, and in some cases achieve spatiotemporal control, in the context of drug delivery makes theranostic fluorescent probes an attractive and intensely investigated research topic. This interest is reflected in the steep rise in publications on the topic that have appeared over the past decade. Theranostic fluorescent probes, in their various incarnations, generally comprise a fluorophore linked to a masked drug, in which the drug is released as the result of certain stimuli, with both intrinsic and extrinsic stimuli being reported. This release is then signaled by the emergence of a fluorescent signal. Importantly, the use of appropriate fluorophores has enabled not only this emerging fluorescence as a spatiotemporal marker for drug delivery but also has provided modalities useful in photodynamic, photothermal, and sonodynamic therapeutic applications. In this review we highlight recent work on theranostic fluorescent probes with a particular focus on probes that are activated in tumor microenvironments. We also summarize efforts to develop probes for other applications, such as neurodegenerative diseases and antibacterials. This review celebrates the diversity of designs reported to date, from discrete small-molecule systems to nanomaterials. Our aim is to provide insights into the potential clinical impact of this still-emerging research direction.

## 1 Introduction

The term cancer is used to refer to a group of diseases caused by the uncontrolled proliferation of cell phenotypes that generate growth signals, but which are insensitive to anti-growth signals. These cells can replicate indefinitely, resist apoptosis, induce angiogenesis, and promote invasion and metastasis.^1^ With more than 10 million cases being diagnosed each year, cancer-related fatalities are expected to rise in the near future. According to an estimate by the World Health Organization (WHO), over 13.1 million deaths caused by cancer are expected by 2030.^2^ In the past 10 years, the cancer-related mortality rate has been reduced owing to decreased smoking, a better understanding of tumor biology and improvements in diagnosis, and more effective therapeutic designs. At present, cancer treatment options consist largely of surgical intervention, chemotherapy, radiation therapy, immunotherapy, and various combinations. Conventional chemotherapeutics work largely by disrupting DNA synthesis and mitosis, resulting in the death of rapidly proliferating cancer cells. These agents are non-selective and can harm normal healthy tissues, resulting in significant unanticipated and undesired side effects, such as nausea and appetite loss. Indeed, the deleterious effects of chemotherapies on normal healthy tissues and organs are often dose-limiting and can contribute to poor clinical outcomes. Furthermore, many chemotherapeutics in current use suffer from poor bioavailability and limited uptake in tumors. As a consequence, relatively high doses need to be administrated. This can result in increased toxicity in normal cellular environments and the onset of multiple drug resistance, which is a major limiting factor in controlling metastatic cancer.^3^ Disease relapse is another major challenge. It is now well-documented that cancer cells develop drug resistance after prolonged exposure to anticancer drugs. The majority of chemotherapeutic drugs are administrated to prolong the patient’s survival and alleviate symptoms, referred to as “palliative chemotherapy”. However, such treatment plans may also have a significant adverse effect on the patient’s mental and physical health, making it difficult to maintain treatment and increase the life expectancy of terminally ill patients. Innovative drug delivery systems with improved targeting abilities offer the promise of addressing these limitations and can thus allow for improvements in cancer therapy.

The limitations of conventional cancer therapy have prompted efforts to understand the real causes of cancerous disease at the molecular and cellular levels, as well as the design of therapeutic agents for treatment. The concept of targeted therapy, developed in the lengthy aftermath of “oncogene addiction” (the reliance of certain tumor cells on a single active oncogenic pathway to retain their malignant characteristics), has spawned the development of several strategies to overcome the primary drawbacks of conventional cancer therapies by targeting disease-related mechanisms and characteristics.^4^ In principle, an ideal cancer therapy would provide the proper medication at an optimal level to the correct target so as to achieve localized disease control with minimum systemic toxicity. More rigorous diagnostic and therapeutic coordination, better classification of patient characteristics, improved stratification of tumor subpopulations, and the development of therapies customized to individuals are likely needed if this long-standing goal is to be met.

The term “theranostics” refers to systems that combine imaging from non-invasive modalities with a therapeutic component. By definition, the imaging and therapeutic components must be contained within a single construct. Specific functionalization with targeting moieties can allow the direction of the theranostic to cancerous lesions. Optical (absorption, fluorescence, or bioluminescence), nuclear (PET, SPECT) photoacoustic, ultrasound, and MR imaging techniques are widely utilized in theranostics.^5−7^ They are favored since the expression of receptors corresponding to targets of interest can vary in different tumor types, as well as in distant metastatic locations, which, in turn, makes classic biopsy-based approaches less than ideal.^8,9^ In contrast, images provided by appropriately designed theranostics can allow the delivery of a cytotoxic agent to a tumor to be confirmed. Imaging data can also be used to monitor therapeutic outcomes. Currently, fluorescence-based optical imaging is receiving considerable attention in the context of theranostic development.^10−12^ Although such imaging benefits from high sensitivity and resolution, it is currently only employed clinically for superficial regions such as those associated with image-guided surgical resection and ocular imaging.^13−15^ When applied to deep-seated tumors, auto-fluorescence and scattering are major limitations. These limitations can be partly overcome in a preclinical setting through the use of near-infrared (NIR) fluorophores (excitation in the 650–900 nm range). Translating the promise of NIR-based optical imaging into a clinical setting represents a current challenge in theranostics development to effectively deliver therapeutic payloads.

**Figure 1:**
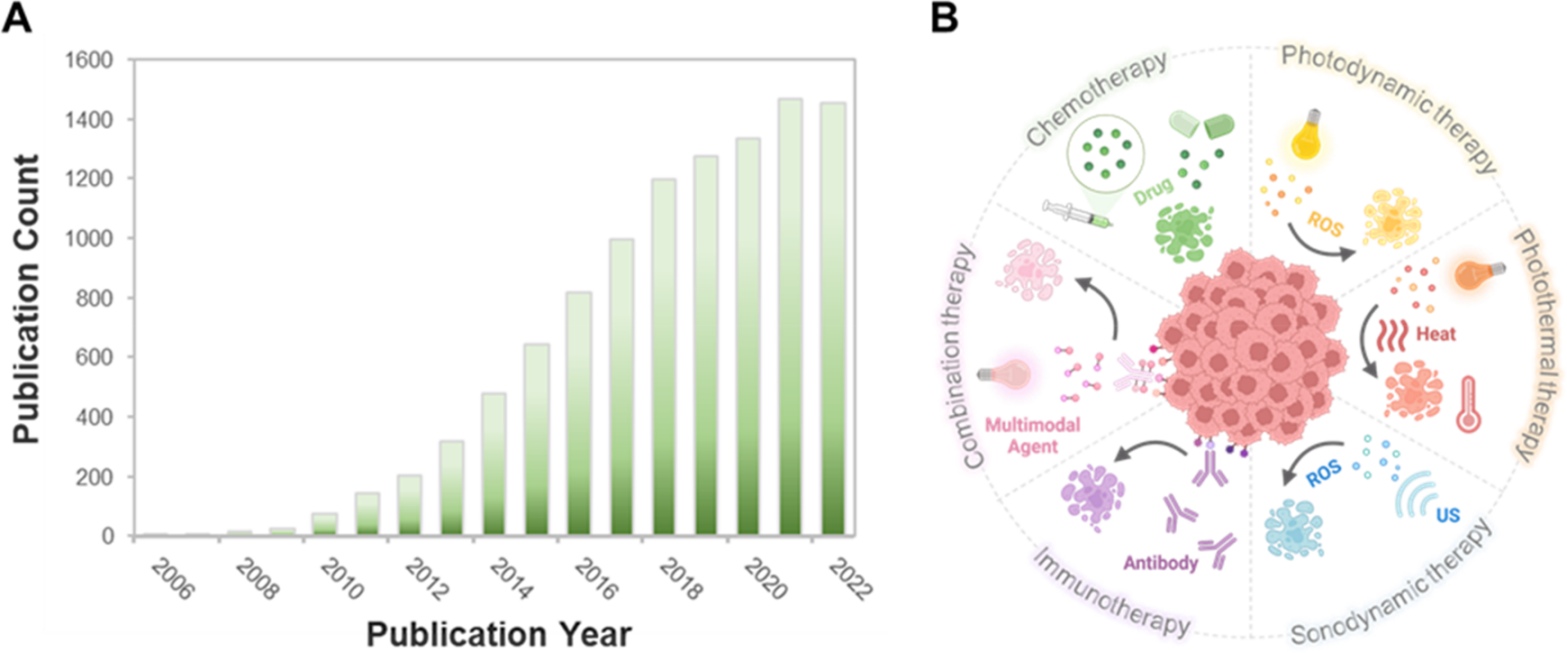
(A) Number of publications per year on “theranostic fluorescent probes” (Web of Science). Search keywords are “theranostic probes and theranostic fluorescent probes”. (B) Schematic illustration of theranostic probes and their application as targeted diagnostics and therapeutics.

To deliver cytotoxic payloads effectively, it has proved useful to “mask” them with specific chemical moieties to furnish prodrugs with limited pharmacological activity. To date, the prodrug strategy has been used to optimize the pharmacokinetic or pharmacodynamics properties of drugs that are currently available in the market. Ideally, the constituent therapeutic agents regain their original activity in the presence of cancer-specific biomarkers through a “masked-to-unmasked” conversion thereby providing targeted therapeutic effects with minimal off-target toxicities.^16,17^ Although prodrugs are generally regarded by the regulatory authorities as being new chemical entities, their enhanced performance in comparison to the parent drug can speed up the drug development process, potentially saving labor, resources and time. Currently, work on theranostic fluorescent probes constitutes a major research area, as underscored by the exponential increase in publications on this topic over the past decade (Figure 1).

In this review, we aim to highlight gaps in our knowledge and address why the field of cancer theranostics has yet to deliver on its promise of improving patient survival. We will provide an overview of our current understanding of tumor biology as it relates to targeted drug delivery systems and rationally designed cancer therapeutics. Particular focus will be placed on fluorogenic theranostic probes that become activated in tumor-specific environments and their application to localization-enhanced chemotherapy, photodynamic therapy (PDT), photothermal therapy (PTT), sonodynamic therapy (SDT), and various mixed modalities (Figure 1). Fluorogenic theranostic probes developed for use in other fields, such as Alzheimer’s, antiaging, and antibacterial applications, will also be covered briefly. For each system, the activation strategy is discussed and the therapeutic potential is compared with the corresponding conventional therapeutic. This treatment, it is hoped, will allow the putative benefits of the theranostic approach to be assessed while guiding further improvements. Barriers to clinical translation will also be discussed. We believe that the present review will promote further research in this field and streamline the development of theranostic agents for clinical use. While nanoparticle-based theranostics are beyond the scope of this review, in certain cases we note the benefits of nanoformulations in the study of specific theranostics. As indicated above, the field has grown rapidly in recent years. As such, the current review is not intended to be comprehensive. Rather, major advances made in the field over the last five years have been highlighted using selected examples. The exclusion of certain papers in this review is not meant to indicate a lack of significance. It simply reflects the vastness of the field, which has necessarily required that a selection be made. We refer the readers to the reviews cited in each section for further information.

## 2 Theranostic Fluorescence Probes in Cancer Therapy

Tumors are collections of abnormally growing cells that can be benign or malignant. While the difference between benign and malignant tumors is a significant topic in cancer pathology, in broad brushstrokes, benign tumors, such as a skin wart, are largely limited to the parent site, with no appreciable invasion of neighboring normal organs or tissues. Nor, does distant dissemination occur. In contrast, malignant tumors are prone to metastasize or spread throughout the body through the circulatory or lymphatic systems. As a result, malignant tumors are frequently referred to as cancerous, and they are usually more resistant to localized therapy due to their spreading potential. The complex microenvironment of malignant cells, which includes cooperative support via several mechanisms, plays a crucial role in cell survival, progression, and metastatic potential.^18−20^

**Figure 2:**
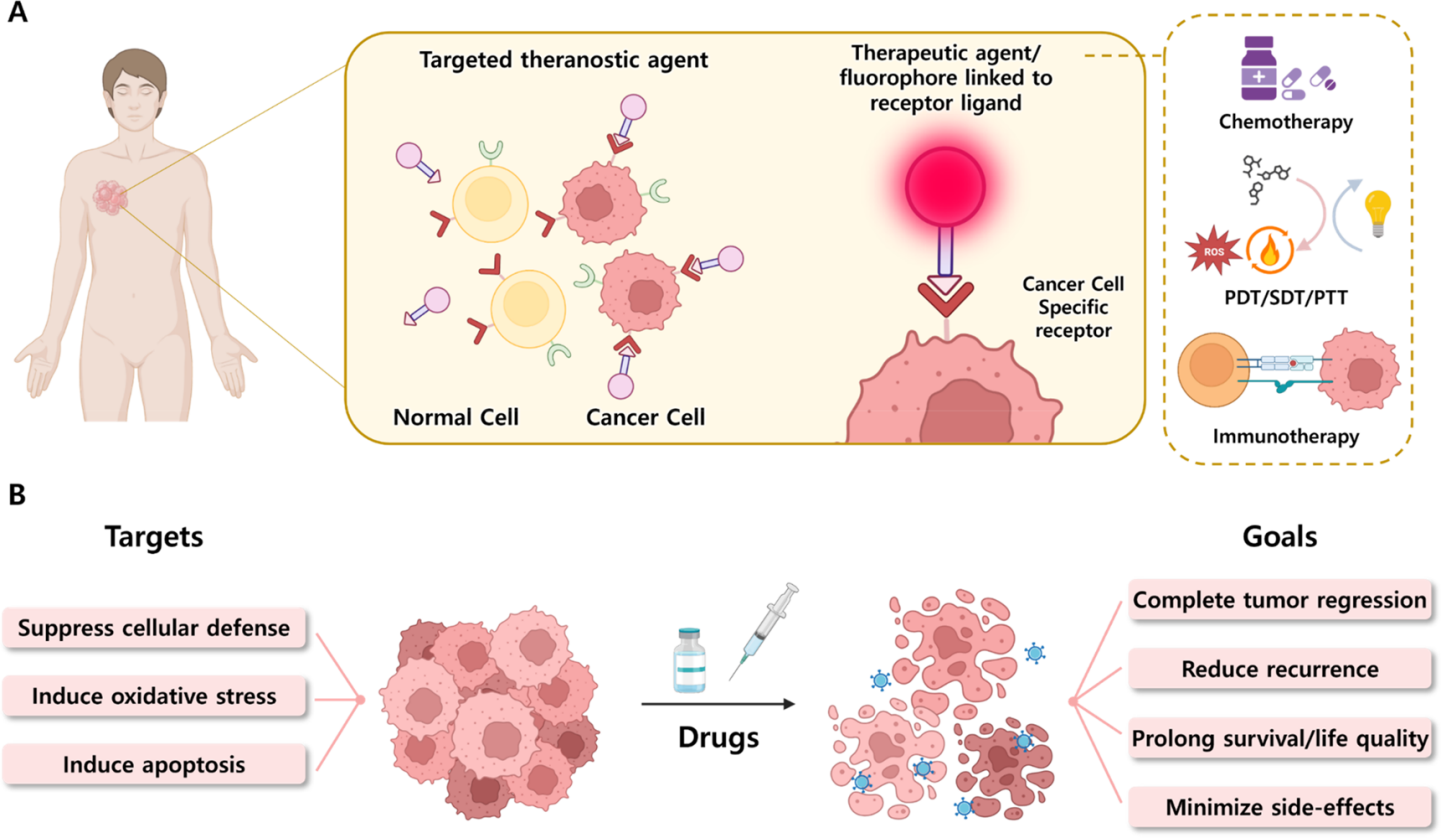
Schematic diagram showing targeted theranostic agents for cancer treatment. (A) Model showing a generalized targeted theranostic agent (chemotherapy/PDT/SDT/PTT/immunotherapy) conjugated with receptor substrate specific to a cancer cell receptor. (B) Targeted cancer therapeutic approach to achieve maximum therapeutic outcomes with reduced side-effects.

Despite considerable progress in cancer-related research, many cancer types can still not be successfully cured. One of the major reasons is the inability to diagnose the oncogenic alterations inside the body during the early stages of cancer development. The liquid biopsy, which relies primarily on screening mutated proteins, DNA, RNA, as well as other elevated markers in patient blood samples, has emerged as a promising strategy in recent years.^21−23^ More classic tissue biopsies and clinically validated imaging tools are also used widely to detect cancerous diseases at early stages.^24−26^ However, additional advances are needed. A further limitation to successful patient outcomes is the inability of current therapeutic regimens (e.g., chemotherapy, PDT, PTT, SDT, and immunotherapy) to identify and target malignant cells directly. This lack of specificity represents a major therapeutic constraint reflected in a failure to deliver therapeutic regimens locally to cancerous tissues. Over the past few decades, several novel approaches have been developed, raising hopes for future drug delivery programs. Cancer-targeted therapeutics and their formulations in particular have shown promise in mitigating the drug-mediated toxicities to nearby tissues and organs (Figure 2). This could be helpful in preventing collateral damage, including the one that causes stress and organ failure. At present, these strategies for the most part have only been subject to preclinical testing. There is thus a need for critical clinical studies that might allow a robust assessment of their merit.

### 2.1 Theranostic Fluorescence Probes in Chemotherapy

**Figure 3:**
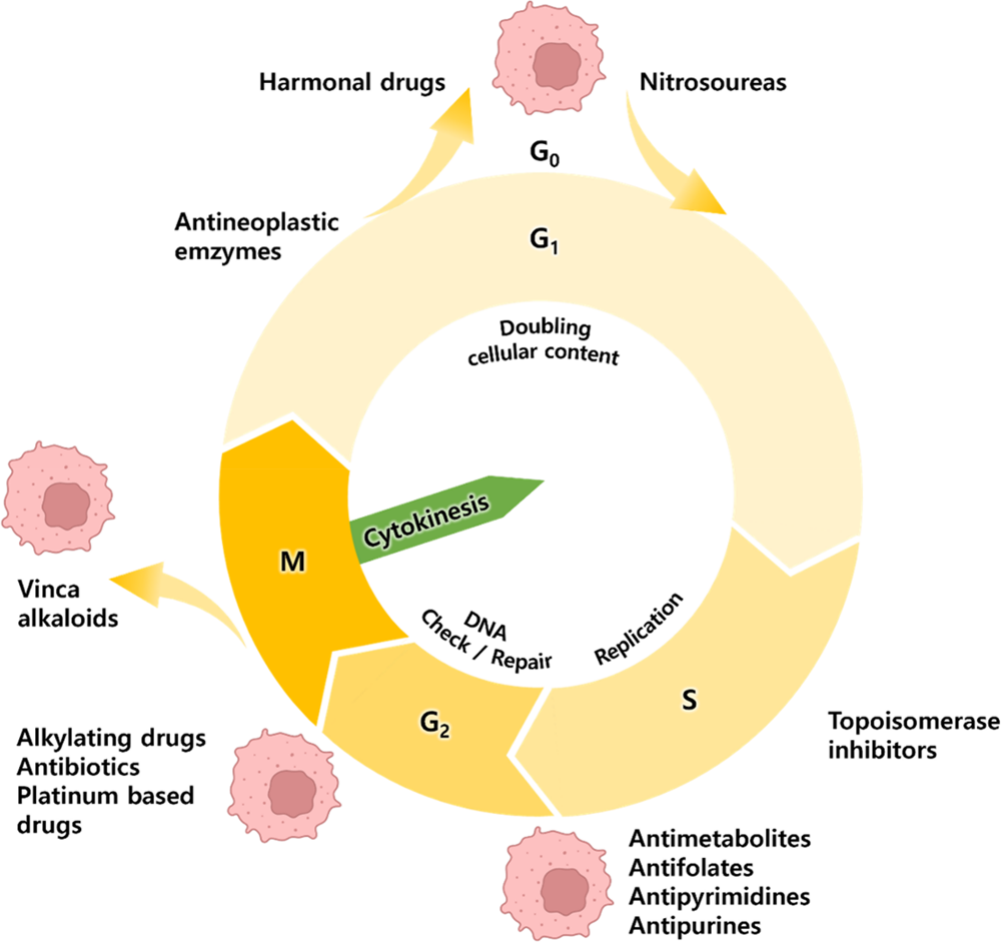
Different classes of chemotherapeutic agents in clinical practices. G_o_ - Resting phase; G_1_ - growth; S - DNA synthesis and replication; G_2_ - growth and preparation for mitosis; M - mitosis.

The concept of chemotherapy, i.e., destroying cancerous cells through cytotoxic drugs and agents, came into the picture after the first report published in 1947 that mustard gas could harm lymphatic tissues. Later, the results were confirmed in animal models (mice) with nitrogen mustards being shown effective in inhibiting lymphoma tissue.^27,28^ Chemotherapeutic drugs usually work by mitigating cancer cell growth and ameliorating tumor-related stress. Compared to normal cells, cancer cells are characterized by rapid growth and enhanced proliferation rates. Hence, these drugs typically have a greater effect on cancer cells than normal cells. A plethora of anticancer therapeutic agents have been developed over the years and, not surprisingly, they operate by diverse mechanisms of action. While some agents perturb cellular metabolism, others target crucial cellular enzymes. A majority of reported agents interfere with vital cellular processes, such as DNA damage repair and DNA replication, immune response, apoptosis regulation, etc. (Figure 3).^29,30^ These modes of action can lead to high activity but also a lack of specificity for tumors over normal tissues. This lack of specificity poses a significant therapeutic constraint in that it can lead to off-target toxicities. One approach to overcoming this limitation involves modifying the structure of the agent in question to create so-called theranostic probes that target selectively cancer cells or the TME and which then release an active payload. In the limit, this strategy, which relies on an appropriate choice of masking/demasking steps can be used to deliver intrinsically nonselective chemotoxins (parent drugs) to cancer cells selectively.^31,32^ Similar approaches have been used to create activatable theranostic fluorescent probes. In this section, we review various classes of fluorescent theranostic probes organized according to their mode of activation.

**Figure 4:**
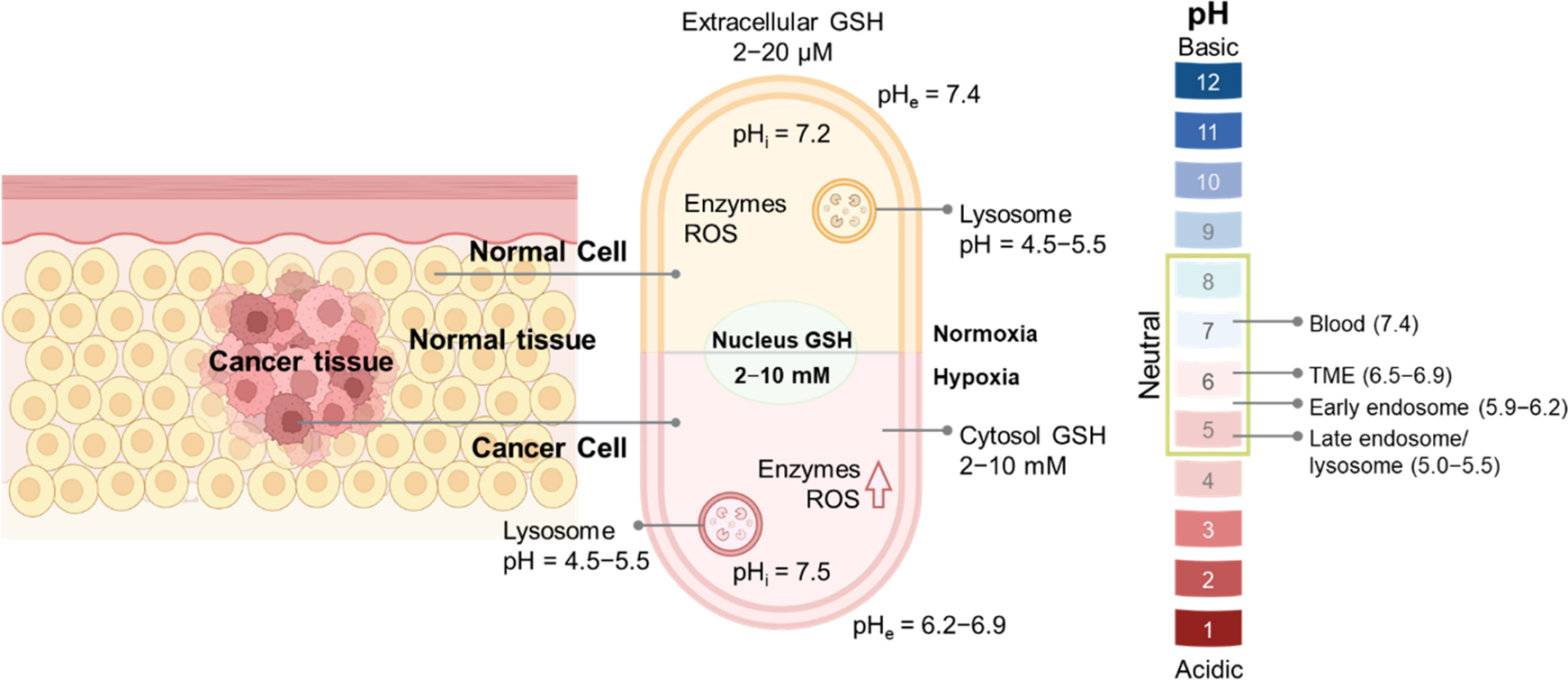
Schematic diagram showing different microenvironments of normal and tumor tissues [pH_i_ = intracellular pH; pH_e_ = extracellular pH, ROS = reactive oxygen species].

There is a distinction between the microenvironment of normal tissues and that of malignant tumors. Certain specific physiological indicators, such as acidic pH, enhanced reactive oxygen species (ROS), greater intracellular glutathione (GSH) levels, enzyme overexpression, and a reductive or hypoxic microenvironment, distinguish malignant tissues from nearby normal tissues and organs (Figure 4).

We have classified theranostic probes used in various treatment modalities (chemotherapy, PDT, PTT, SDT, immune therapy, etc.) into several subsections in this part to differentiate their mode of activation in the tumor microenvironment.

#### 2.1.1 pH-Responsive Fluorescent Probes

The pH values for normal tissues are typically around 7.4, while those of a tumor microenvironment are typically 0.5–1.0 units lower.^33,34^ This is due to the vigorous metabolism within tumor cells that promotes rapid glucose uptake and lactic acid production under the hypoxic conditions that characterize most solid tumors. The acidic pH of cancerous lesions plays a significant role in tumor development, recurrence, metastatic spread, and the development of drug resistance. However, not all tumors show a particularly acidic TME. For instance, pH_e_ values of 6.94 ± 0.08 have been reported for soft tissue sarcoma and adenocarcinoma, whereas values of 7.20 ± 0.07 have been noted in malignant melanoma and squamous cell carcinomas.^35^ Nevertheless, an aberrant pH is regarded as a universal marker of solid tumors, irrespective of tumor type and stage. As a result, pH is often exploited as an endogenous stimulus for cancer-targeting drug delivery systems (DDS). To date, pH-responsive DDS has mainly been reported in MCF-7,^36,37^ HeLa,^38^ and BxPC-3^39^ cancer models.

An overarching goal of DDS development is to enhance the therapeutic efficacy of the parent drugs or cytotoxic agents upon which they are based and to minimize deleterious side effects.^40,41^ To achieve these objectives the system in question must be stable enough to allow delivery and circulation but release the drug efficiently at the target site. In pH-triggered DDS, an increased local proton concentration at the lysosomal, endosomal, cellular, or tumor tissue levels serves as the stimulus to promote drug release. In recent decades, a number of pH-responsive theranostic systems have been reported that take advantage of this strategy. For the most part, the systems in question involve drugs that are attached to acid-labile chemical linkers such as acetals, hydrazines, oximes, and imines. These chemical linkers are attractive because they are relatively stable at physiological pH (7.4), but undergo rapid hydrolysis in acidic endosomes, thus providing for acidic tumor site specificity.^25^ Unfortunately, this strategy is only applicable to drugs with free aldehyde or ketone groups that can support the formation of this type of labile functionality.

##### 2.1.1.1 Hydrazone/Oxime/Imine-Based Theranostic Probes

**Figure 5:**
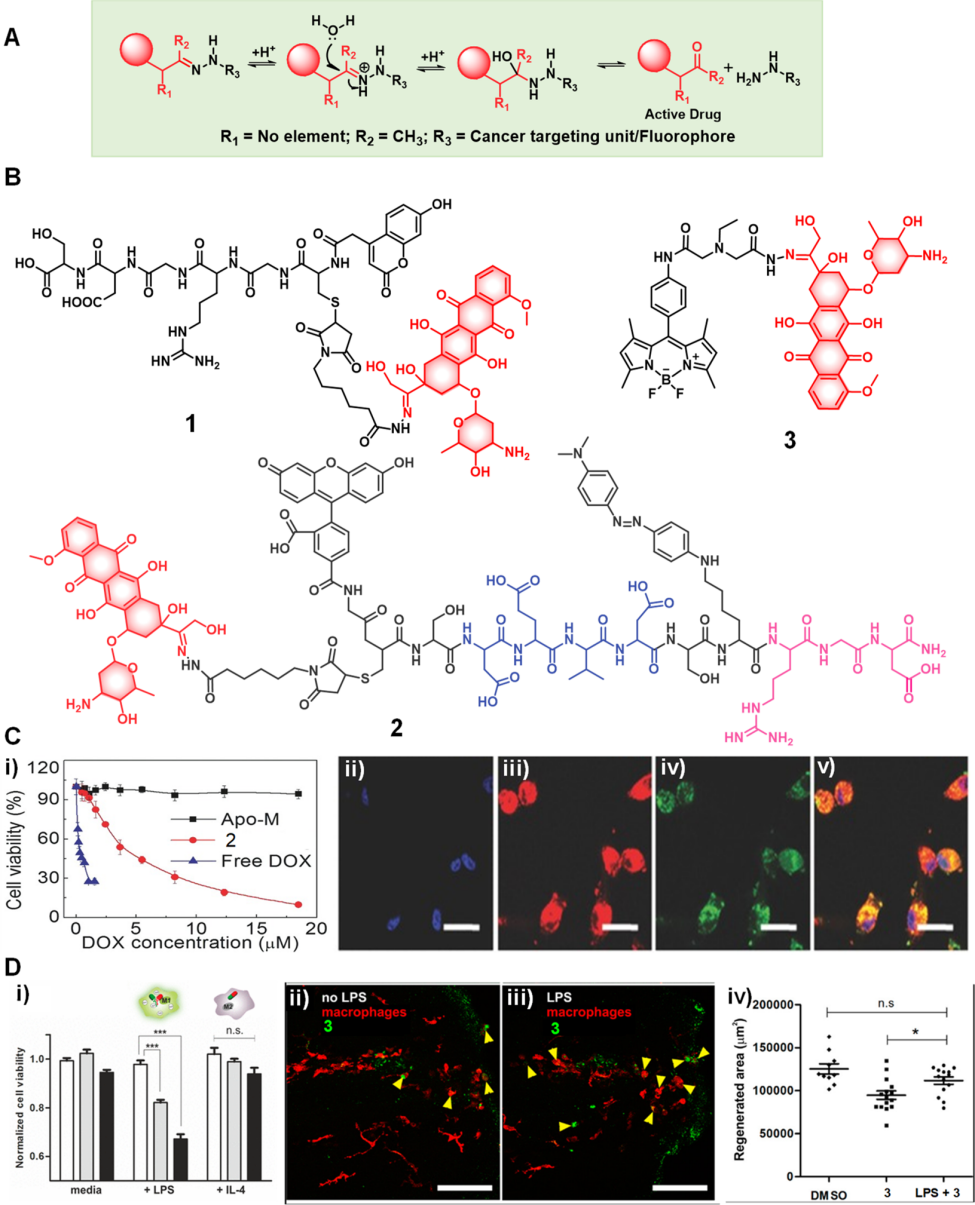
(A) Mode of activation of hydrazone-based pH-responsive theranostic agents. (B) Chemical structures of acid-sensitive theranostic agents (**1**–**3**). (C) (i) Cytotoxicity studies performed on U87 cells upon treatment with Apo-M, theranostic agent **2**, and free doxorubicin (Dox) at different Dox equivalent concentrations. (ii–v) Confocal images of U87 cells incubated with theranostic agent **2** at a Dox equivalent concentration of 10 μg mL^–1^ for 42 h. (ii) Hoechst blue, (iii) Dox red, (iv) 5(6)-carboxylfluorescein (FAM) green, and (v) merged (scale bar = 20 μm). Reproduced with permission from ref (53). Copyright 2015 Wiley Intersciences. (D i) Normalized cell viability of theranostic agent **3** (10 μM, black; 5 μM, gray) and amide analogue (white) in nonactivated media, Lipopolysaccharide (LPS)-induced (100 ng mL^–1^, 18 h) and Interleukin (IL)-4-treated macrophages. (D ii, iii) Fluorescence images of zebrafish treated with **3** (3 μM) without (ii) LPS and (iii) with LPS treatment (100 ng mL^–1^). Scale bar = 50 μm. (D iv) In vivo macrophage quantification in the regenerated tissue area of zebrafish treated with DMSO, theranostic agent **3** with and without LPS. Errors are ± SD (n 10). n.s. = not significant; * *p* < 0.05. Reproduced with permission from ref (54). Copyright 2017 American Chemical Society.

The acid-catalyzed hydrolysis of hydrazone linkers has been widely explored in developing pH-sensitive DDS, particularly in the development of small molecule-based theranostics, as well as nanocarriers,^42^ and polymer-based systems,^43^ for delivering cytotoxins to tumor sites. Detailed NMR spectral studies conducted in the deuterated buffer by Kalia et al. revealed that in the hydrolysis of hydrazone- and oxime-linked drug molecules, nucleophilic attack of a water molecule on the imine carbon is the rate-determining step.^44^ This attack is followed by protonation of the imine nitrogen and subsequent hydrolysis to furnish the free drug molecules (Figure 5). Electron-withdrawing substituents reduce the propensity of the nitrogen atom to undergo protonation resulting in a reduced overall hydrolysis rate. Based upon experiments carried out at pH 7.4, the stability order of various linkers was found to be trialkylhydrazonium ≫ oxime ≫ acyl hydrazone > primary-hydrazone ≫ *sec*-hydrazone > imine. As quaternary ammonium hydrazones are quite stable at pH 5, they are not preferred for acid-sensitive drug delivery applications. On the other hand, acyl hydrazone linkers are inherently attractive due to their high stability at neutral pH and hydrolytic lability in acidic media (pH 5).

The anticancer drug doxorubicin (Dox), an anthracycline drug, used alone or in combination with other agents, constitutes the standard of care for several malignancies, including breast, lung, ovarian, and gastric cancers, multiple myeloma, as well as pediatric cancers, including Hodgkin’s and non-Hodgkin’s lymphomas.^45^ However, Dox use is often limited by side effects, such as fatigue, hepatotoxicity, and cardiotoxicity.^45−47^ Several strategies have been proposed to overcome these side-effects. Within this context, considerable effort has been devoted to improving the delivery of Dox to tumor sites. For instance, Dox has been linked through an acid-sensitive hydrazone linker to a cancer-guiding integrin, GRDS-oligopeptide, as well as a coumarin moiety to give the theranostic agent **1** (Figure 5).^48^ Dox in its free state is intrinsically fluorescent with an emission band at 595 nm (excitation at 470 nm). Hence, it has been widely used in cancer biology-related imaging applications.^49−52^ Before the release of Dox, the fluorescence of the Dox moiety in **1** is quenched due to a contact-mediated quenching process involving the Dox and coumarin moieties. It was found that about 94% of the Dox originally in **1** was released at pH 5 over 11 h. In contrast, under neutral pH (7.4) only 41% drug release was observed. In integrin-positive human glioblastoma U87cells, a dose-dependent toxicity was observed (IC_50_ = 0.19 μg mL^–1^). Further, the fluorescence nature of both free Dox (red) and coumarin (blue) allows prodrug activation and drug localization to be monitored in real-time.

Incorporating cell apoptosis markers in the scaffold design can allow the activation and localization of a cytotoxin to be assessed in a non-invasive manner and the therapeutic dose to be fine-tuned. This strategy is embodied in the dual Forster resonance energy transfer (FRET) agent **2** (Figure 5).^53^ Here, the anticancer drug (Dox) is linked through an acid-labile hydrazone linker to a potent fluorescence quencher, (4-(dimethylamino azo)benzene-4-carboxylic acid (Dabcyl), and a peptide sequence (Asp-Glu-Val-Asp, DEVD) that is responsive to the apoptosis marker, caspase-3. To achieve the real-time monitoring of drug activation at the cellular level, a 5(6)-carboxylfluorescein (FAM) unit was incorporated into the design. The construct was further tagged with an integrin-specific sequence (Arg-Gly-Asp, RGD) to achieve cancer-selective targeting and uptake. Preliminary solution studies confirmed that agent **2** released 90% of the Dox in active form in acidic environments (pH = 5.0) as compared to a much lower degree of Dox release at pH 7.4 (19%). In U87 cancer cells (integrin positive), agent **2** provided for time-dependent fluorescence enhancement ascribed to DEVD peptide cleavage to furnish a green color fluorescence (corresponding to the free FAM group from its quenched state). The corresponding Dox release was associated with noticeable therapeutic effects (IC_50_ = 4.3 × 10^–6^ M) and concomitant caspase-3 activation.

Considerable effort has been devoted to the preparation of constructs that are simpler than **2**, and permit both cancer-specific activation and real-time monitoring of drug release at the cellular level. For example, theranostic agent **3** was developed by connecting Dox to a fluorophore, 4,4-difluoro-4-bora-3a,4a-diaza-*s*-indacene (BODIPY), through a hydrazone bond (Figure 5).^54^ As prepared, construct **3** displayed relatively weak fluorescence at physiological pH. In phagosomes (pH 6.5–4.5), the hydrazone linker is readily cleaved, resulting in an observable fluorescence emission due to the resulting free BODIPY. It was thus tested as a target-based approach for treating immune-related diseases. RAW264.7 macrophages were used as a model system and treated with lipopolysaccharide (LPS) to generate proinflammatory M1 macrophages. This resulted in phagosome acidification. Dose-dependent toxicity was observed in LPS-mediated proinflammatory M1 macrophages treated with **3**, along with a fluorescence “Turn-On” response. In contrast, no such benefits were observed in IL-4-mediated anti-inflammatory quiescent or M2 macrophages. A similar pattern was observed in LPS-treated zebra fish, where apoptotic macrophages produced a red fluorescence, corresponding to Dox and a nearby region produced a green fluorescence, corresponding to BODIPY. The theranostic agent **3** was also tested in an in vivo regeneration model (zebra fish) to monitor phagocytic M1 macrophage activity. Treatment with **3** in combination with LPS resulted in improved M1 macrophage polarization, a finding consistent with enhanced tissue regeneration.

**Figure 6:**
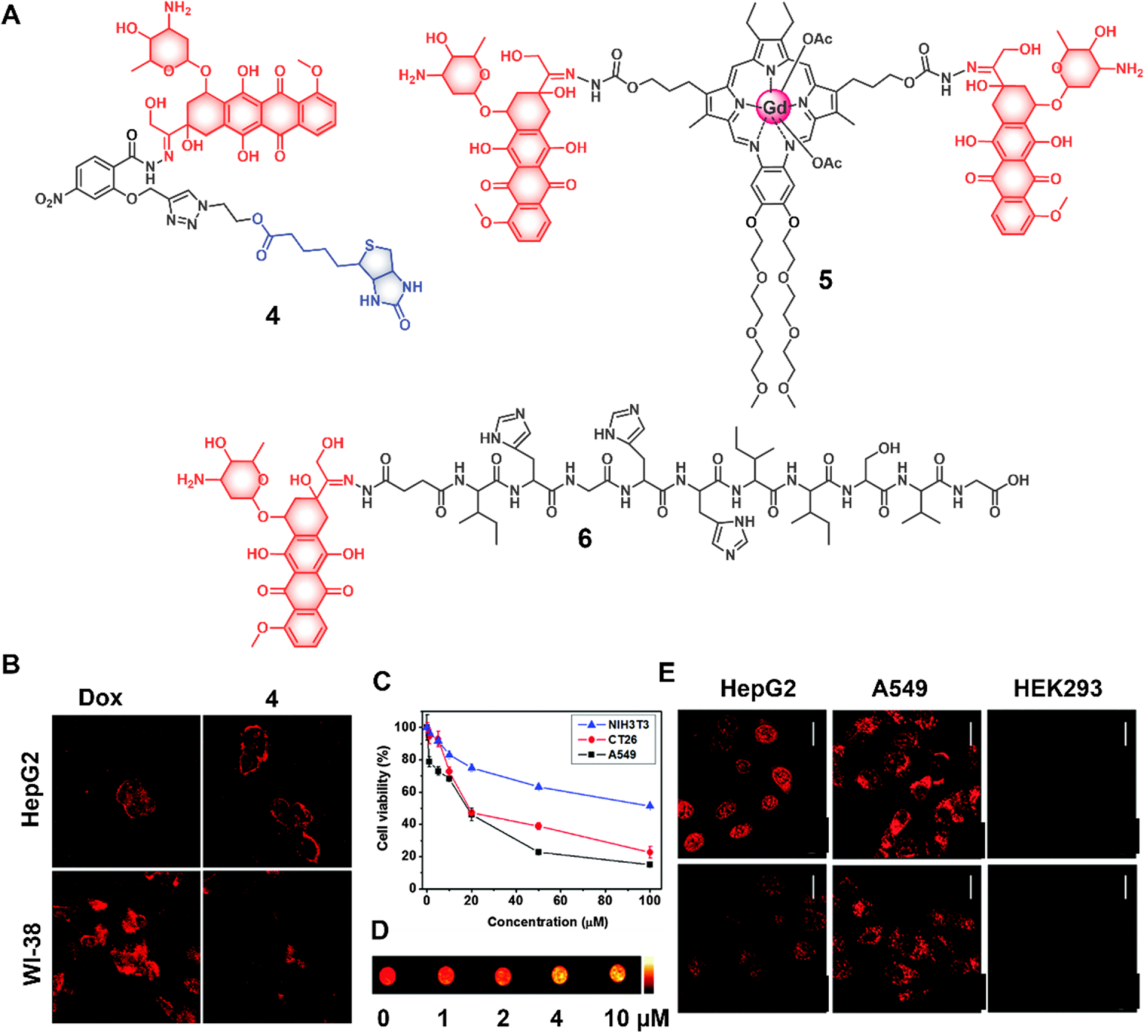
(A) Chemical structures of acid-sensitive theranostic agents (**4**–**6**). (B) Confocal images of HepG2 and WI-38 cells after treatment with Dox and theranostic agent **4** (5 μM), 1 h, Exc. wavelength 488 nm). Reproduced with permission from ref (55). Copyright 2015 Royal Society of Chemistry. (C) Concentration-dependent cytotoxicity of theranostic agent **5** in A549 (black), CT26 (red) cancer cells and normal fibroblast NIH3T3 (blue) cells. (D) T1-weighted MR images of an A549 cell pellet recorded following treatment with different concentrations of theranostic agent **5**. Reproduced with permission from ref (56). Copyright 2016 Royal Society of Chemistry. (E) CLSM images showing cellular uptake and intracellular Dox localization of **6** in HepG2 and A549 cancer cells, as well as HEK293 normal cells at 6 h (1st row) and 2 h (2nd row) post-treatment. Scale bar = 20 μm. Reproduced with permission from ref (57). Copyright 2015 Royal Society of Chemistry.

Theranostic agent **4** was developed and tested for its ability to improve cancer cell-specific uptake of Dox relative to normal cells (Figure 6).^55^ Here, Kim et al. utilized the intrinsic Dox fluorescence to monitor prodrug activation and intracellular localization. Specifically, a nitrobenzene moiety was used as a photoinduced electron transfer (PET)-mediated fluorescence quencher and linked to Dox through a hydrazone bond. For cancer-selective targeting, a biotin unit was incorporated into the design. The agent remained largely intact under physiological conditions up to 7 h. However, under acidic conditions, the hydrazone bond underwent hydrolysis, leading to a fluorescence “Turn-On” response (resulting from free Dox). When tested in vitro, agent **4** demonstrated a good selectivity in biotin- positive cancer cells (HepG2) as compared to normal WI38 (biotin-negative) cells.

The same group further developed a multimodal strategy that was designed to assess cellular uptake and activation by means of two different imaging modalities (fluorescence imaging and magnetic resonance imaging (MRI)).^56^ This strategy, embodied in compound **5** (Figure 6), relies on a hydrazone linkage between two Dox (fluorescent) units and a single paramagnetic motexafin gadolinium (MGd) core, the latter being a specific water-soluble texaphyrin. The diminished fluorescence of intact agent **5** was recovered under acidic conditions. The same phenomenon was seen in cancer cells (A549, CT26). By contrast, no noticeable toxicity was observed in normal fibroblast NIH3T3 cells up to 100 μM concentrations. Construct **5** displayed *T*_1_-contrast relaxivities of about 20.1 ± 0.4 mM^–1^ s^–1^ (at 60 MHz) and 6.1 ± 0.2 mM^–1^ s^–1^ (at 200 MHz) in phosphate buffered saline (PBS) that were greater than those seen for a standard Gd^3+^ contrast agent. Moreover, at low concentrations (4 μM), cell pellet phantoms treated with **5** reached saturation at a relatively high *T*_1_ relaxivity value (cf. Figure 6). Likewise, a tumor-targeting theranostic **6** was developed wherein a targeting sequence AP2H (IHGHHIISVG) was linked through a hydrazone bond to Dox.^57^ This system, designed to undergo cleavage under mildly acidic conditions, was found to display dose-dependent toxicity in a lysosomal protein transmembrane 4 beta (LAPTM4B) positive lung cancer cell line A549 (IC_50_ = 1.14 μM) and a liver cancer cell line HepG2 (IC_50_ = 4.0 μM). In these cell lines, fluorescence studies confirmed that **6** was activated in endosomes and lysosomes with subsequent translocation to the nucleus (Figure 6). In contrast, negligible activation was seen in normal HEK293 cells.

**Figure 7:**
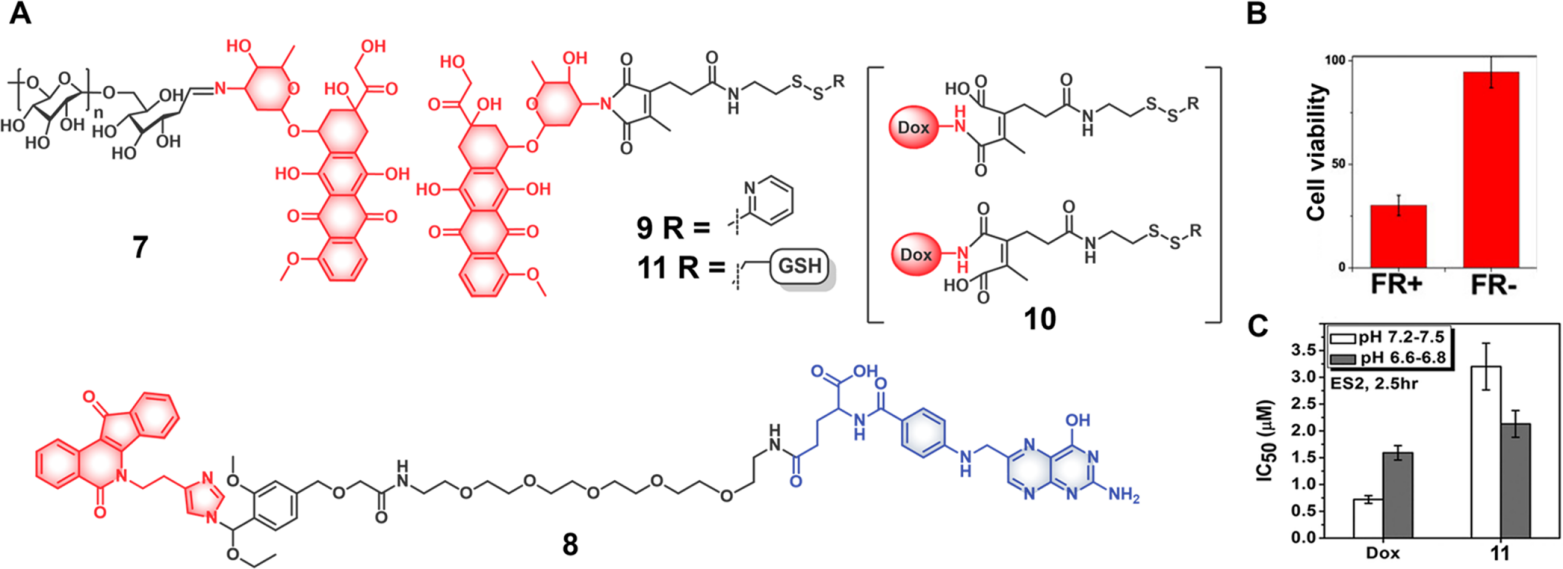
(A) Chemical structure of acid-sensitive theranostic agents (**7**–**11**). (B) Cell viability studies of theranostic **8** in KB Folate receptor (FR+) cells and FR-knockdown KB cells. Reproduced with permission from ref (60). Copyright 2014 American Chemical Society. (C) IC_50_ values of Dox and theranostic **11** in ES-2 cells at different pH. Reproduced with permission from ref (62). Copyright 2017 Royal Society of Chemistry.

While hydrazone-based linkers have been widely used in small molecule-based systems, some effort has been devoted to the use of oxime-based linkers. However, reports on their use are limited and most of these reports have concerned polymeric systems. In the current review article, we restrict our focus to discrete molecule-based theranostic systems. In a report by Jin et al., a terephthaladehyde moiety was used to form an oxime linker within a PEG-Dox micelle system. Slow hydrolysis was seen at pH 5.0 (*t*_1/2_ = 15 h).^58^ In contrast, a higher drug release rate was observed at neutral pH (20% at 12 h). These results underscore the low stability margin of oxime linkages as compared to acyl hydrazone-based linkers. A dextran-Dox conjugate (**7**) was developed by Xu et al. as a potential theranostic agent designed to permit acid-sensitive Dox delivery into human hepatoma HepG2 cancer cells (Figure 7). Fluorescence-based drug release experiments revealed that **7** at neutral pH (7.4) showed approximately 25.9% of active Dox release over a period of 72 h. In contrast, under acidic conditions, increased oxime hydrolysis and enhanced Dox release were seen (i.e., pH 6.8 = 40.4%, pH 6.0 = 64.7%, and pH 5.0 = 87%, 72 h).^59^ A reduced analogue of **7** that was prepared by reduction of the oxime linker was not expected to exhibit acid lability. Endocytosis-mediated cellular uptake of theranostic **7** and further hydrolysis in acidic environments were demonstrated using confocal laser scanning microscopy and HepG2 cancer cells. A cell viability assay conducted on HepG2 cells revealed that treatment with **7** (72 h incubation) led to toxicity (IC_50_ = 0.73 μg mL^–1^) comparable to free Dox (IC_50_ = 0.62 μ g mL^–1^). Furthermore, tumor inhibition studies conducted on the H22-xenograft murine model revealed that compared to other controls (Dox 45.4%, reduced analog of **7** 19.2%), treatment with **7** (intravenous administration) resulted in significant tumor growth inhibition (71.0%) and improved survival rates with decreased side-effects.

The above studies highlight the potential utility of acid-sensitive chemical linkers. However, they underscore a seemingly obvious truism, namely the importance of having a good understanding of the underlying chemistry before choosing a specific type of chemical linker for a particular DDS application.

##### 2.1.1.2 Other Acid Responsive Theranostic Probes

Yang et al. developed the *N*-ethoxybenzylimidazole (NEBI) moiety as a tunable acid-sensitive chemical linker and used it to achieve the folate-positive cancer cell-selective delivery of the anticancer drug indenoisoquinoline.^60^ Here, acid-based hydrolysis involves the “aminol” ether functionality. Construct **8** relies on using an imidazole ring nitrogen to form an aminol ether group (Figure 7). In acidic environments, protonation of the imidazole ring facilitated the spontaneous release of the parent drug molecule. The hydrolysis and drug release rate can be tuned by the choice of groups on the phenyl ring. While the addition of an electron-withdrawing group (nitro) resulted in a slower hydrolysis rate (*t*_1/2_ = 6900 h, at pH 5.5), substitution with an electron-donating group (methoxy) facilitated a rapid drug release (*t*_1/2_ = 0.6 h, at pH 5.5). Further, no matter what type of substituent was employed, the half-life of the prodrug at pH 7.4 was 10-fold higher than at pH 5.5. In vitro studies involving folate-positive KB cells revealed the targeted delivery and release of active drug with an IC_50_ of 60 μM. In contrast, knockdown of the folate receptor resulted in a decrease in efficacy (IC_50_ = 655 μM). Further modification of prodrugs with PEG linkers resulted in diminished activity in the folate-positive KB cells (IC_50_ = 250 μM). Due to the intrinsic fluorescence of the parent drug, prodrug **8** offered the possibility of monitoring its cellular uptake and activation through fluorescence microscopy. This study highlights the potential utility of the NEBI chemical linker to deliver drugs via processes that rely on folate receptor-mediated endocytosis.

Maleic acid-based derivatives have also been used as acid-sensitive linkers for drug delivery applications. In this case, the drug release mechanism is based upon the intramolecular cyclization of the malonyl amine at a pH less than the p*K*_a_ of the free carboxylic acid moiety. Early research focused on the use of the *cis*-aconitic anhydride moiety, which allowed the formation of the final conjugate. However, the resulting *cis*-aconitric acid-based conjugates often suffer from decarboxylation and trans-isomerization.^61^ An example of this approach is embodied in prodrug **9** that is based on Dox (Figure 7).^62^ At a pH below the p*K*_a_ of the carboxylic acid, intermediate **10** is the dominant species. Prodrug **9** could be linked to GSH, resulting in theranostic agent **11**. Upon a decrease in pH, an excellent Dox release response was observed with about 70% Dox release being observed at pH 6.0. In contrast, only a 10% Dox release was seen at pH 7.0 over the course of 5 h. Dox, bearing a free amino group (p*K*_a_ = 8.2), is sensitive to acidic tumor environments in terms of both its uptake and toxicity. In ES-2 (ovarian cancer cells), Dox possesses higher toxicity (IC_50_ = 1.6 ± 0.1 μM) at extracellular pH 6.7 than at neutral pH 7.4 (IC_50_ = 0.7 ± 0.1 μM). With maleamic acid-based chemical linkers, this preference is perturbed. Conjugate **10** showed a comparable potency at pH 6.7 (IC_50_ = 2.1 ± 0.3 μM) and pH 7.4 (IC_50_ = 3.2 ± 0.4 μM). It is interesting to note that although conjugate **11** was less potent than the parent drug, it mitigates the undesired pH-dependent toxicity profile of Dox. Moreover, the intrinsic fluorescence of Dox offered the possibility of monitoring drug activation and cellular localization, thereby allowing **11** to serve as an acid-sensitive theranostic agent.

In summary, acid-labile chemical linkers continue to be used to develop; small molecule-based drug delivery systems. In acidic environments, ideally tumor microenvironments, the pH-sensitive linker undergoes acid-catalyzed drug release. Depending on the target site, a range of acid-sensitive linkers can be used. For example, for targeted delivery of cytotoxins to solid tumors (pH = 6.0–7.0), maleamic acid-based chemical linkers appear to be an excellent choice as they are sensitive to subtle pH value changes. However, to target lysosomes and endosomes (pH = 4.5–6.0), acyl hydrazone-based linkers appear more suited. In any event, further research devoted to developing chemical linkers appears warranted and could translate into improvements in cancer-targeted drug delivery.

#### 2.1.2 GSH Responsive Fluorescent Probes

A variety of redox processes take place within different intra- and extracellular environments and in tissues. Some of these most common redox couples include nicotinamide adenine dinucleotide phosphate (NADP^+^/NADPH), oxygen/superoxide (O_2_/O_2_^•–^), thioredoxin (TrxSS/Trx(SH)_2_), and glutathione (l-γ-glutamyl-l-cysteinyl-glycine) (GSH/GSSG). The glutathione redox couple has garnered considerable attention in the context of theranostic and drug delivery applications owing to the relatively high GSH concentrations inside cells (1–10 mM) and its role in maintaining cellular integrity, as well as mediating cellular differentiation, metabolism, and apoptosis. GSH also plays a key role as an antioxidant by preventing ROS-mediated damage.^63−65^ In contrast, in the extracellular matrix, blood, and even on cell surfaces, GSH is present in much lower concentrations (2–20 μM). This is due to higher protein concentrations in these regions that are capable of stabilizing disulfides. In contrast, the intracellular microenvironment is kept reductive through the action of nicotinamide adenine dinucleotide phosphate (NADPH), as well as GSH reductase. Thus, within cells, GSH is largely maintained in its reduced form.^66,67^ It is interesting to note that a significant GSH concentration gradient exists within the intracellular compartments.^68^ Of significance is that the GSH concentrations are about 4-fold higher in tumor tissues compared to healthy tissues, a finding ascribed to the rapid proliferation of cancerous cells.^69−71^ On the one hand, these elevated levels of GSH can reduce the efficacy of administered therapeutics in a number of cancers, including ovary, breast, lung, liver, bone marrow, and colon cancer.^72^ However, the relatively high GSH levels in cancerous lesions can be exploited to create redox-responsive theranostic agents that release active drugs/fluorophores within tumor cells. Since the GSH concentration in the extracellular environment is typically lower, the prodrugs can be tuned in such a way as to possess adequate stability in the extracellular matrix before releasing the cytotoxins once internalized within the cancerous cells. Among the most widely used chemical linkers to achieve this purpose is a disulfide (S–S) bridge, which undergoes thiol–disulfide exchange in the presence of free bithiols.^73,74^ In fact, under appropriate conditions of design, GSH can serve as a potent bioactivator capable of triggering drug release from S–S containing prodrugs.

**Figure 8:**
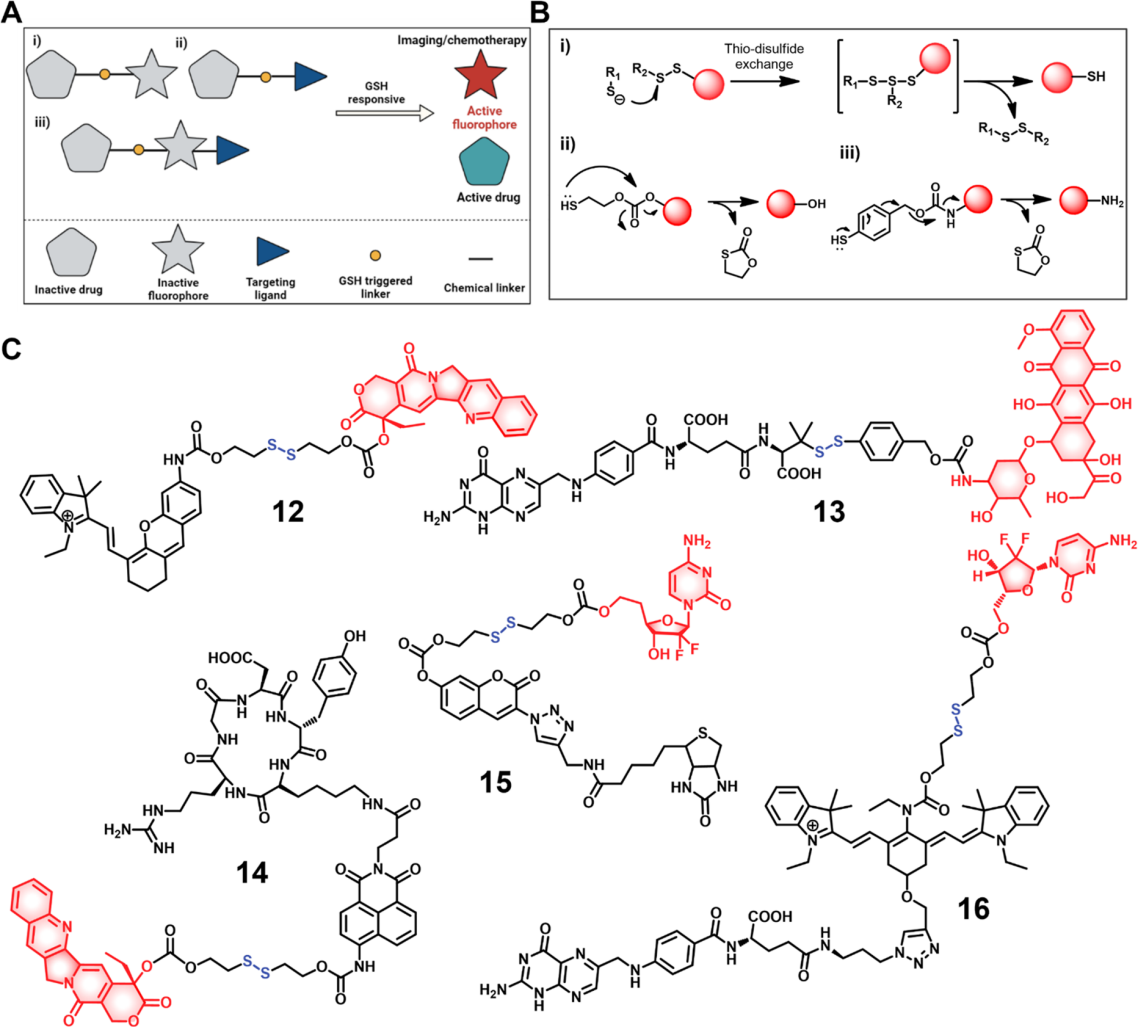
(A) Schematic illustration of GSH-responsive activatable molecular theranostic probes. (B) Disulfide cleavage and self-immolative drug release mechanisms. (C) Chemical structures of selected GSH-responsive theranostic probes (**12**–**16**).

The disulfide strategy has been extensively explored in developing prodrugs using a variety of carriers, including antibodies, peptides, and small molecules. In the context of small molecule-based theranostics, several drug candidates and fluorophores, including Dox, camptothecin,^75,76^ paclitaxel,^77,78^ gemcitabine,^79−81^ napthalimide,^82^ hemicyanine,^83^ dicyano-methylene-4H-pyran,^84−86^ and fluorescein,^87^ have been explored. To achieve the essential disulfide linkage required for GSH-mediated release (and producing a free thiol group), the drug molecules and fluorophores are typically modified before disulfide linker construction. To date three types of strategies have been used predominantly: (i) chemotherapeutic drugs are linked to a fluorophore through a cleavable chemical linker, (ii) chemotherapeutic drugs with intrinsic fluorescence are connected to a targeting ligand through chemical linkers, and (iii) chemotherapeutic drugs are connected to fluorophores and a targeting ligand through a multicomponent strategy (Figure 8).

**Figure 9:**
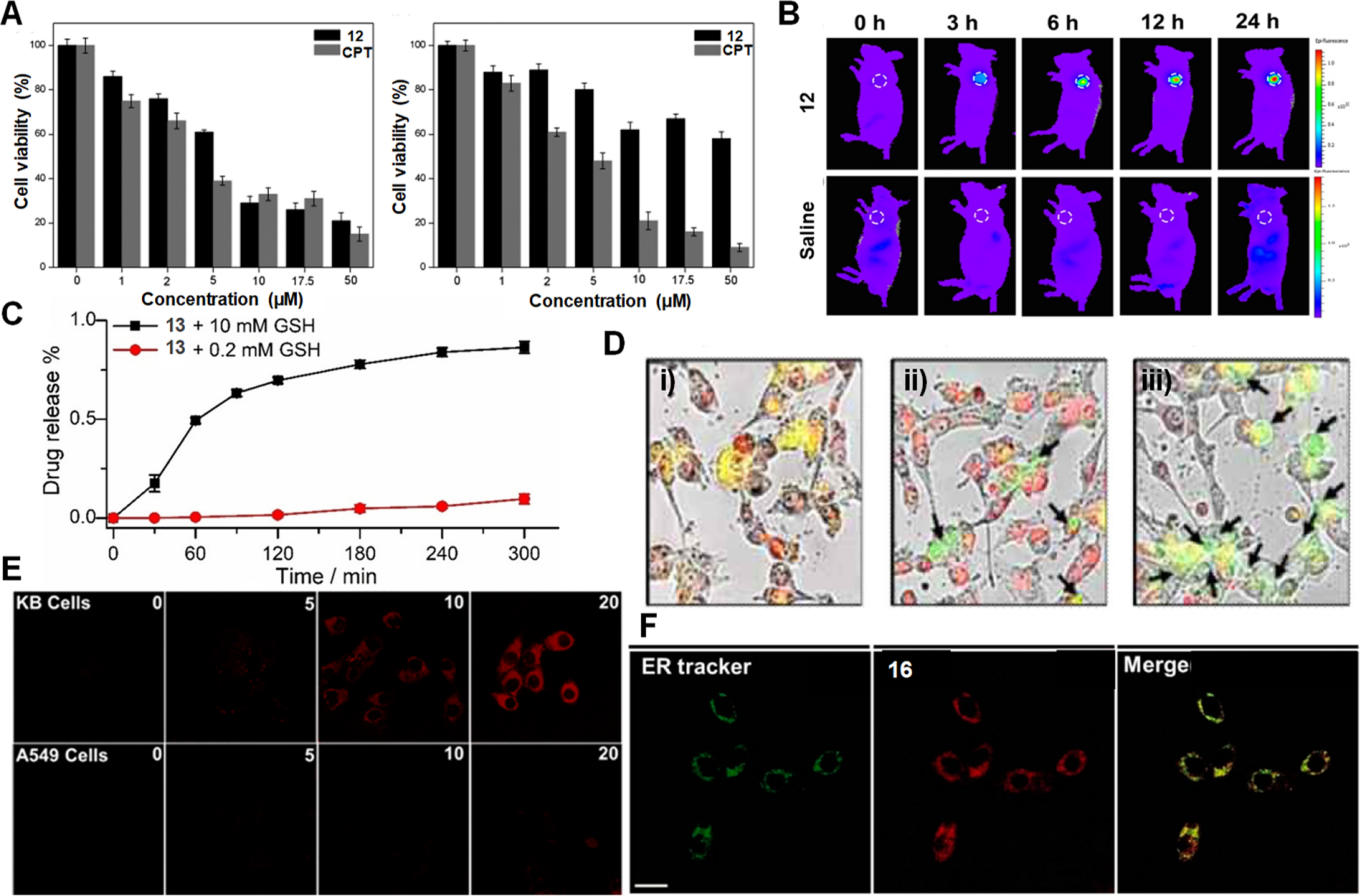
(A) Cell viability of theranostic probe **12** and parent drug CPT at different concentrations as tested in HepG2 cancer and HL-7702 normal cells. (B) In vivo images of H22-tumor bearing mice after administrating **12** (i.v.) at different time intervals. Reproduced with permission from ref (83). Copyright 2016 American Chemical Society. (C) Drug release profile of theranostic probe **13** after incubation with 0.2 mM and 10 mM GSH at pH 7.0 (mean ± SD, *n* = 3). Reproduced with permission from ref (95). Copyright 2019 Wiley Intersciences. (D) Colocalization studies of theranostic probe **14** (10 μM) with (i) ER Tracker Red, (ii) Lyso Tracker Red DND-99, and (iii) Mito Tracker Red FM. Excitation 458 nm, 543 nm band-path (505–530 nm), and long-path (>585 nm) emission filters were used. Reproduced with permission from ref (82). Copyright 2014 American Chemical Society. (E) Confocal images of theranostic probes **16** (1.0 μM) treated KB and A549 cells at different time points (excitation = 633 nm, emission = 650–750 nm, scale bar = 20 μm). (F) Colocalization studies showing confocal images of KB cells treated with ER Tracker (excitation = 514, emission = 530–570 nm), theranostic probe **16**, and merged image. Scale bar = 20 μm. Reproduced with permission from ref (80). Copyright 2013 American Chemical Society.

Under category (i), several strategies have been pursued. For instance, in the case of drugs of fluorophores bearing free hydroxyl and amine groups carbonate or carbamate esters, respectively, may be used to make the connection through a disulfide-containing linker. This approach is illustrated by theranostic agent **12** (Figure 8), which was developed to monitor the GSH-responsive activation of camptothecin (CPT) in an H22 tumor-based mice model.^83^ Here, the anticancer drug CPT was conjugated to a hemicyanine-based fluorophore through an S–S linkage. Theranostic **12** was found to be weakly fluorescence, presumably because the free amino group of the fluorophore is blocked as a result of linker formation. Upon exposure to GSH, the disulfide bond undergoes reductive cleavage with concomitant fluorescence enhancement (at 702 nm), owing to the formation of the free hemicyanine fluorophore and the simultaneous release of free CPT. Cell-based studies confirmed that theranostic **12** exhibited significant cytotoxicity in the HepG2 cancer cell line, as compared to the HL-7702 normal liver cell line (Figure 9). Further, an intense fluorescence signal (at 702 nm) corresponding to the free hemicyanine fluorophore was observed in the HepG2 cells as compared to a normal liver cell line. This difference was ascribed to the elevated GSH levels in the HepG2 cancer cells. The enhanced emission was also taken as evidence that theranostic **12** provides for preferential tumor localization, as confirmed through an IVIS luminal in vivo imaging (intravenous administration). A similar strategy has been used by numerous groups to monitor the GSH-mediated activation of theranostic agents in various tumor models.^79,83−86,88−95^

Per strategy (ii), anticancer drugs with an intrinsic fluorescence are typically linked to cancer-targeting ligands, through a cleavable disulfide linker. As true for strategy (i), this approach has been used for drug molecules bearing hydroxyl or amine functionalities. An example is the GSH-responsive folate-doxorubicin theranostic **13**.^95^ This system incorporates an α,α-dimethyl-substituted *p*-thiophenyl urethane-based disulfide, as well as a carbamate ester. The presence of two methyl groups improves the stability of aromatic thiols 2-fold as compared to typical aliphatic disulfide linkers. Further, unlike an aliphatic thiol (p*K*_a_ 8–9), the low p*K*_a_ value of aromatic thiols (≈6) was expected to improve the release kinetics. Theranostic probe **13** also incorporates a folate targeting unit. Folate receptors (FR) are known to be upregulated in various cancer types. It was thus expected that conjugate **13** (Figure 8) would be internalized through recycling endosomal pathways.^96^ Preliminary solution studies confirmed that theranostic **13** exhibits good stability in 0.2 mM GSH in a phosphate-buffered solution, a condition mimicking the redox environment associated with blood circulation. By contrast, upon incubation with 10 mM GSH (mimicking the cancer cell cytosol), a smooth drug release pattern was observed characterized by a *t*_1/2_ of about 1.3 h (Figure 9). These results led to the suggestion that **13** might be amenable to clinical translation, where it would be expected to be eliminated from the circulatory system with a *t*_1/2_ of about 30 min.^97^ In addition to **13**, several other theranostic agents that embody strategy (ii) have been prepared and tested in various cancer models both in vitro and in vivo.^98,99^

Strategy (iii) offers the prospect of improving the specificity of the therapeutic payload in a theranostic with a commensurate reduction in systemic toxicity. This is because it involves tagging the drug components with cancer-targeting units. In the case of nonfluorescent drug candidates, this strategy also allows for the visualization of the drug delivery system through additional fluorescence labeling. The potential of this approach was shown by Kim et al., who developed an RGD peptide-decorated naphthalimide pro-camptothecin (pro-CPT) theranostic agent **14** (Figure 8) for image-guided chemotherapy.^82^ The multifunctional agent **14** consists of an RGD cyclic peptide for cancer targeting, a naphthalimide component as a fluorophore, and the topoisomerase I inhibitor CPT as the anticancer drug, linked through a GSH-responsive disulfide linker. Theranostic **14** was designed to be endocytosed into U87 cancer cells as the result of interactions between the RGD moiety and the a_v_β_3_ integrin receptor, which is overexpressed in these cancer cells. The relatively high GSH levels in cancerous cells were then expected to trigger the cleavage of the disulfide bond, resulting in the release of the active cytotoxin CPT and the naphthalimide fluorophore. Incubating U87 cancer cells with **14** gave rise to an intense red-shifted emission output at 535 nm in the endoplasmic reticulum (Figure 9). Meanwhile, the CPT was found to diffuse to the cell nucleus and induce an antiproliferative effect. Further research was conducted to validate the universality of this strategy for otherwise nonfluorescent drug candidates. For example, theranostic agent **15** (Figure 8) was developed to monitor the GSH-selective delivery of gemcitabine, another anticancer drug.^81^ System **15** contains biotin as the cancer-targeting moiety, coumarin as the fluorophore, and gemcitabine as the model anticancer drug. Tracking the coumarin fluorescence revealed that the theranostic is trapped in the cell lysosomes and that drug activation occurs in the cytoplasm. Thanks to coumarin’s intrinsic two-photon absorption,^100^ the therapeutic action of **15** could be assessed at the subcellular level.

Other theranostic agents were prepared and tested in the search for improved therapeutic efficacies.^101^ A representative example is theranostic **16** (Figure 8). This system is a Cy7-gemcitabine drug conjugate linked through a disulfide bond and incorporating cancer-targeting folate at the cyclohexene ring of the fluorophore.^80^ Cyanine dyes contain two nitrogen atoms linked to a π-conjugated polymethine chain.^102^ These fluorophores display high molar absorptivity, narrow absorption, and emission bands with tunable spectroscopic profiles in the UV/vis and NIR regions.^103−105^ Further, they generally display low systematic toxicity and excellent biocompatibility. Cyanines (>650 nm) have been widely used for in vivo applications due to their minimal auto fluorescence, deep tissue penetration, reduced light scattering, and minimal phototoxicity.^106−108^ Upon incubation with theranostic **16** folate-positive KB cancer cells displayed a prominent fluorescence signal within 20 min. In contrast, no fluorescence signal was observed in folate-negative A549 cancer cells (Figure 9). Based on these results, it is thought that theranostic **16** is preferentially taken up by folate-positive KB cells, through a folate-mediated endocytosis mechanism. Fluorescence-based colocalization studies revealed that the drug is activated in the endoplasmic reticulum and induces apoptosis.

**Figure 10:**
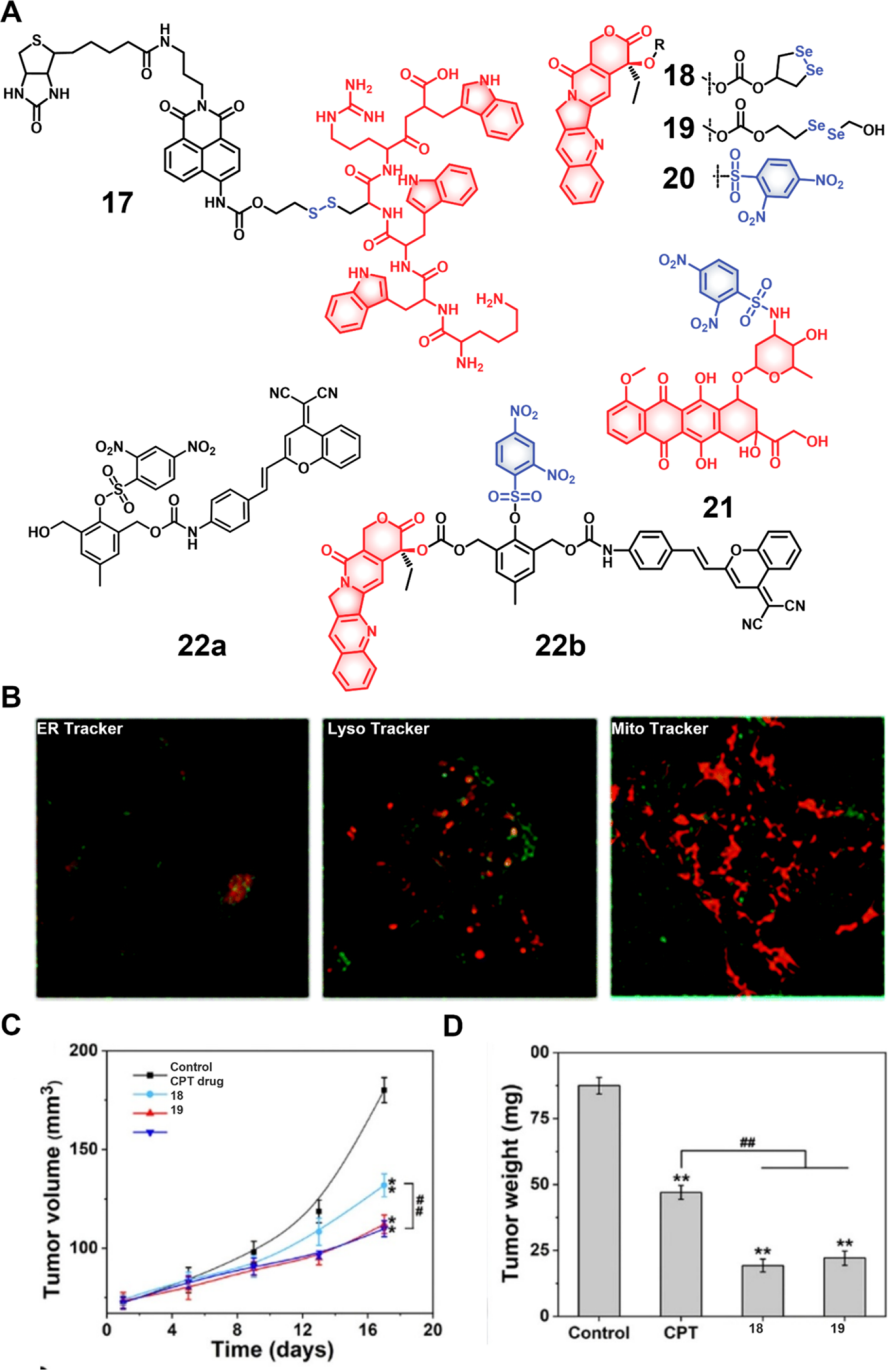
(A) Chemical structures of GSH-responsive theranostic probes **17**–**22**. (B) Colocalization images of theranostic probe **17** (10 μM) with ER tracker Red, Lyso Tracker Red, and Mito Tracker Red FM in HepG2 cancer cells. Reproduced with permission from ref (115). Copyright 2014 Royal Society of Chemistry. (C) Tumor growth inhibition in HepG2 xenografted mice upon treatment with control, CPT, and theranostic probes **18** and **19**. ^##^*p <* 0.01 compared to CPT treated group. (D) Tumor weights after treatment per (C). **p < 0.01 compared to the control group and ^##^*p <* 0.01 compared to CPT treated group. Reproduced with permission from ref (120). Copyright 2021 American Chemical Society.

Extensions of the above strategies have been used for the targeted delivery of peptide-based anticancer agents.^109−112^ There are several peptide-based drug candidates possessing good anticancer activity that are devoid of appreciable side effects; however, their translational into the clinic has been limited due to their low metabolic stability, poor membrane permeability, and low blood circulation residence times.^113,114^ Peptide-based theranostic agents could overcome some or all of these limitations. With such considerations in mind, Kim et al. developed the theranostic system **17** (Figure 10). This system was designed to provide for the cancer-targeted delivery of the Holliday junction (HJ) inhibitor peptide 2 (KWWCRW), which possesses antimicrobial and anticancer activity. In **17**, the cysteine residue on the HJ inhibitor is used to form disulfide linkages with naphthalimide, which was further connected to a biotin moiety.^115^ Theranostic **17** was selectively taken up in biotin-positive HepG2 cells and displayed a red-shifted fluorescence enhancement at 540 nm. Compared with the HJ inhibitor alone, theranostic **17** showed improved concentration-dependent toxicity in HepG2 cells with the drug being localized in the endoplasmic reticulum. Collectively, these results support the contention that theranostics derived from peptide-based drug candidates may be able to improve the inherent therapeutic efficacy of the parent drug through improved delivery, better therapeutic targeting, and fluorescence-based tracking.

In addition to disulfide linkers, other chemical linkers have also been explored for GSH-responsive drug delivery applications, with selenium receiving particular attention. Selenium and sulfur belong to same elemental group and possess similar chemical properties.^116^ The Se–Se bond exhibits a low redox potential at 172 kJ mol^–1^, as compared to the C–Se bond (244 kJ mol^–1^) or S–S bond (268 kJ mol^–1^).^117−119^ Therefore, Se–Se linkers typically display higher GSH sensitivity than analogous S–S linked systems. Fang et al. developed two seleno-based CPT theranostic agents, **18** and **19** (Figure 10).^120^ Incubation with GSH activates both theranostic agents and produces an enhanced fluorescence emission at 430 nm (excitation = 365 nm), as validated by HPLC analyses. In the presence of 1 mM GSH, both theranostic agents demonstrated nearly full drug release in 5 min in a Tris-EDTA buffer. The released selenol intermediates were thought to improve the potency of **18** and **19** through the depletion of intracellular of GSH while providing for increased ROS generation. In a HepG2 xenograft mouse model, treatment with **18** and **19** reduced the tumor weight by around 75%; in contrast, CPT therapy reduced the tumor weight by 53%. These promising results notwithstanding, diselenide-based linkers have been used less frequently than disulfide linkers. This may reflect synthetic barriers, as well as the fact that diselenide linkers display dual sensitivity toward both GSH and ROS, which can cause unexpected activation and unwanted drug release.^121^

Other biothiols, including cysteine (Cys) and homocysteine (Hcy) also play key roles in maintaining the redox balance of cellular microenvironments.^121^ Cys deficiency can result in a variety of diseases, including cardiovascular diseases, leukopenia, liver damage, neurological disorders, cancer, and hematopoietic malfunction.^122,123^ Many GSH-sensitive linkers show sensitivity toward Cys, which can result in undesired drug activation in healthy tissues where both Cys and GSH coexist at certain concentrations. On the other hand, systems that respond to only Cys could allow for selectivity. Recently, 2,4-dinitrobenzenesulfonyl (DBS) has emerged as a Cys-sensitive linker for drug delivery applications. It has seen increased use for use with drug candidates that possess free hydroxyl (OH) or amine (NH_2_) groups. In separate studies from the groups of Jo and Johansson et al, theranostic agents **20** (based on SN-38, an active metabolite of irinotecan)^124^ and **21** (using DOX)^125^ were prepared by connecting the DBS group directly to the respective parent drugs (Figure 10). Functionalization of DBS results in fluorescence quenching in both theranostic agents.

In reductive environments, DBS undergoes aromatic nucleophilic substitution followed by sulfur dioxide (SO_2_) release, to allow active drug release with concomitant fluorescence enhancement. Introducing self-immolative chemical linkers into DBS linkers can further increase their capacity to support a range of drug candidates and fluorophores. This benefit was demonstrated by Wu et al., who developed theranostic agent **22b**. This system contains a thiol-responsive DBS trigger, CPT, and the NIR fluorophore dicyanomethylene-4H-chromene (DCM), connected through a 2,6-bis(hydroxymethyl)-4-cresol linker.^126^ As prepared, theranostic **22b** is nonemissive due to the presence of the DBS and carbamate linkages. In the presence of thiols, a reaction cascade takes place to release both CPT and produce an NIR fluorescence arising from the free DCM. Theranostic agent **22b** displayed a dose-dependent cytotoxicity in HeLa cancer cells (IC_50_ = 5.8 μM) and L929 cells (IC_50_ = 8.9 μM) that was enhanced compared to a control conjugate without a drug (**22a**). Intratumoral administration of a liposomal formulation of theranostic **22b** demonstrated significant tumor growth inhibition.

#### 2.1.3 Hydrogen Sulfide Responsive Theranostic Probes

**Figure 11:**
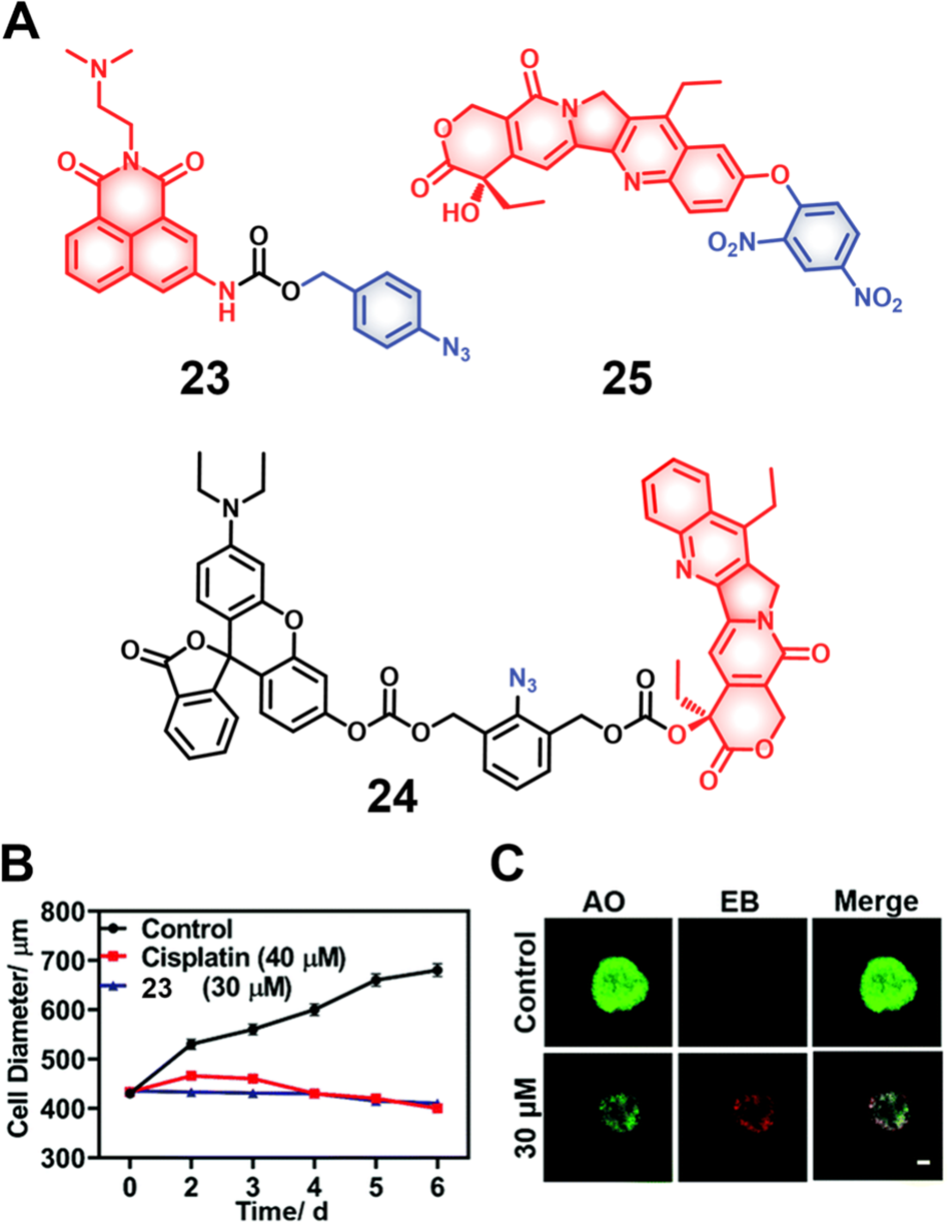
(A) Chemical structures of hydrogen sulfide-responsive theranostic probes **23**–**25**. (B) Changes in 3D U87MG tumor spheroids upon treatment with theranostic probe **23** (30 μM), cisplatin (40 μM), and control at different time points. (C) Confocal images of U87MG tumor spheroids after treatment with **23** (30 μM, 48 h) and staining with acridine orange (AO) (green) ethidium bromide (EB) (red). Scale bar = 100 μm. Reproduced with permission from ref (134). Copyright 2021 Royal Society of Chemistry.

Hydrogen sulfide (H_2_S) is a poisonous gas and is known for its flammability, rotten egg-like smell, and causticity.^127^ Endogenous H_2_S, along with carbon monoxide (CO) and nitric oxide (NO) is a primary gasotransmitter.^128,129^ It plays a vital role in the regulation of several critical physiological processes, including cell differentiation, proliferation, survival/death, and metabolism.^130,131^ Dysregulation of H_2_S has been linked to several diseases, including cancer, Alzheimer’s, and diabetes.^132^ H_2_S protects neurons from oxidative damage by elevating GSH levels, thus indirectly scavenging ROS.^133^ The importance of H_2_S has prompted the development of H_2_S-responsive theranostic agents as potential cancer treatments. H_2_S possesses reductive character in addition to being a recognized nucleophilic, particularly in its deprotonated forms. It is capable of selectively reducing azides to amines. The strong electron-donating nature of amines can be further exploited to facilitate fluorophore/drug release. Qian et al. developed the H_2_S-responsive theranostic agent **23** (Figure 11), containing amonafide, an anticancer drug of interest as a targeted therapy for glioblastoma cancer.^134,135^ The intrinsic fluorescence of amonafide is quenched due to a PET effect. Incubation with H_2_S results in the reduction of the azide to an amine, with subsequent self-immolative linker breakdown, thereby furnishing the active drug via a 1,6-elimination reaction. Fluorescence studies confirmed that the drug was localized in the lysosomes in U87MG cells and further translocated to the nucleus, where it induced cytotoxicity through a combination of DNA damage and mitochondrial dysfunction mechanisms. In a U87MG 3D spheroid model, this theranostic agent demonstrated a significant toxicity at a relatively low dose (30 μM, 2 days treatment) and was found to outperform cisplatin (40 μM, 2 days treatment) as inferred from the observed destruction of tumor spheroid integrity. A similar strategy was used to develop theranostic probe **24** (Figure 11) for H_2_S-mediated drug delivery of SN-38 in colon cancer cells.^136^

Taking the nucleophilic character of H_2_S into consideration, Li et al. developed the theranostic agent **25** (Figure 11), which is composed of the anticancer drug SN-38 linked to an electron-withdrawing dinitrophenyl (DNP) ether group.^137^ As prepared, **25** is non-emissive since possible intramolecular charge transfer (ICT) is blocked. In the presence of H_2_S, the ether bond is cleaved with a concomitant fluorescence enhancement and active drug release. In vitro, studies confirmed that theranostic agent **25** was preferentially taken up and activated in H_2_S-elevated HCT116 and 4T1 cancer cells and displayed cytotoxicity. The SN-38 component possesses intrinsic fluorescence and its release allows for the real-time monitoring of drug activation and therapeutic action. Clearly, the use of the nucleophilicity of H_2_S represents an attractive mode for prodrug activation, although further studies will be needed to study the selectivity of these designs for H_2_S in the presence of other nucleophiles. Despite this promising report, at present, the authors are unaware of other theranostic agents that exploit this feature of H_2_S.

#### 2.1.4 Hydrogen Peroxide Responsive Theranostic Probes

The term “ROS” refers to relatively unstable molecules and radicals generated by the reduction of molecular oxygen (O_2_), including hydrogen peroxide (H_2_O_2_), superoxide (O_2_^–^), singlet oxygen (^1^O_2_), and hydroxyl radicals (•OH).^138^ Endogenous ROS are primarily generated through mitochondrial metabolism and NADPH-dependent enzyme-catalyzed reactions.^139,140^ Further, ROS can also be produced exogenously through UV light exposure and xenobiotic agents. When produced in a controlled manner, ROS plays a critical role in cellular homeostasis and acts as a messengers in cellular growth, proliferation, migration, and apoptosis.^141^ Due to their ability to alter specific protein activities, ROS are also involved in blood vessel modulation, oxygen sensing, immune function, and gene activation.^142−144^ However, elevated ROS levels may cause oxidative stress, resulting in nucleic acid, lipid, and protein damage. Nonhomeostatic ROS has been correlated with several pathological disorders such as aging,^145^ cancer,^146^ cardiovascular diseases,^147^ diabetes,^148^ and neurodegenerative diseases.^149,150^

In mitochondria, H_2_O_2_ is produced from superoxide ions through superoxide dismutase (SOD)-mediated processes. Inflamed and cancerous cells generate elevated H_2_O_2_ levels (0.5 nmol/10^4^ cells/h) as compared to normal cells (0.050 ± 0.004 nmol/10^4^cells/h).^151^ H_2_O_2_ can act as a precursor for other highly reactive species, such as peroxynitrite, hypochlorite, and hydroxyl radicals. Due to the relatively high stability of H_2_O_2_ (*t*_1/2_ = 1 ms) in comparison to other ROS (*t*_1/2_ < 1 μs in most instances), and the accumulation of H_2_O_2_ during oxidative stress, H_2_O_2_ has attracted attention as a trigger for ROS-responsive drug delivery systems.^152^ In the context of cancer-specific theranostic probes, arylboronic esters, thioethers/thioketal, amino acrylates, selenium/tellurium, and polyproline have been explored as H_2_O_2_-responsive chemical linkers. In this section, we will summarize theranostic probes activated under oxidative stress (i.e., H_2_O_2_).

**Figure 12:**
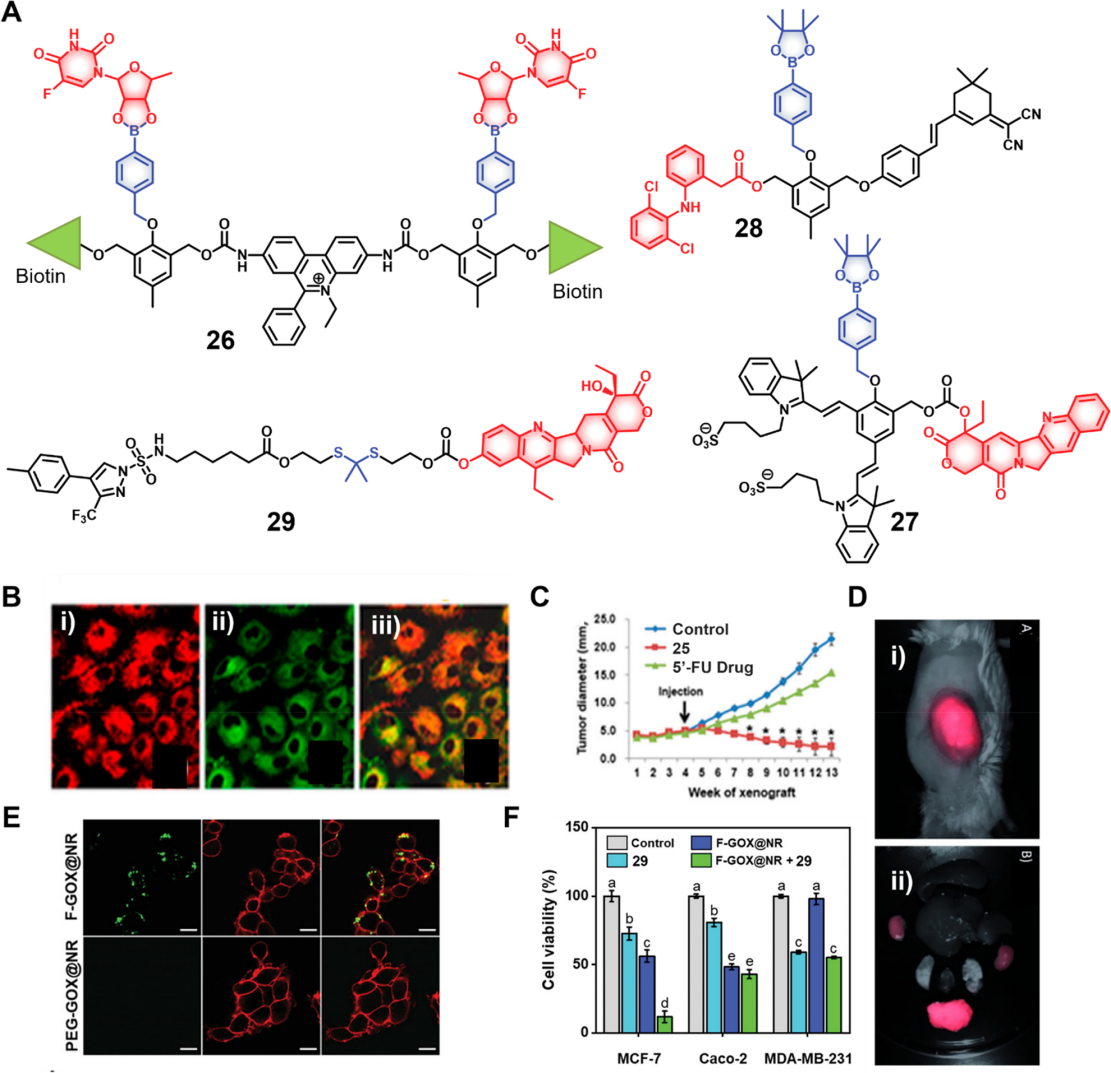
(A) Chemical structures of H_2_O_2_-responsive theranostic probes (**26**–**29**). (B) Confocal images showing mitochondrial localization of theranostic probe **26**. (i) **26** (50 μM), (ii) Mito Tracker (iii), and merged image in A549 cancer cells. (C) Tumor inhibition of A549 xenograft model upon treatment with PBS as control, theranostic probe **26**, and 5′-FU drug (tail vein administration, *n* = 8). Reproduced with permission from ref (153). Copyright 2014 American Chemical Society. (D) Severe combined immunodeficiency (SCID) image of (i) U87 MG tumor-bearing mouse after i.v. administration of theranostic probe **27** (1 min post administration) and (ii) images of dissected organs (lungs, heart, kidney, liver, and spleen) from a mouse 5 min post administration (excitation = 595 nm, emission = 700 nm). Reproduced with permission from ref (154). Copyright 2015 Wiley Intersciences. (E) Confocal images showing folate receptor-mediated uptake of nanoreactor F-GOX@NR (folate decorated silica-based glucose oxidase encapsulated nanoreactor. (F) Cell viabilities after treating with **29**, nanoreactor F-GOX@NR and controls in the MCF (COX-2 positive, folate positive), Caco-2 (COX-2 negative, folate receptor negative) and MDA-MB-231 (COX-2 positive, Folate receptor negative) cell lines. Reproduced with permission from ref (160). Copyright 2022 Wiley Intersciences.

An example of an H_2_O_2_-activated probe was reported by Kim and co-workers who developed a molecular theranostic **26** (Figure 12) composed of two anticancer drug units, 5′-deoxy-5-fluorouridine (5-fluorouracil as the active toxin) linked to a self-immolative linker through two phenyl boronic acid moieties, as well as an ethidium fluorophore (a mitochondrial apoptosis marker).^153^ In biotin-positive A549 lung cancer cells, theranostic **26** was preferentially taken up and further accumulated into mitochondria due to the positively charged ethidium. The elevated H_2_O_2_ levels in these cancer cells served to trigger the release of 5′-deoxy-5-fluorouridine, which was further converted to 5-fluorouridine (5′-FU) by thymidine phosphorylase. The concomitant fluorescence enhancement arising from the free ethidium and subsequent DNA intercalation was used as a fluorescence reporter and allowed the real-time monitoring of cellular apoptosis. Theranostic probe **26** was further evaluated in an A549 xenograft mouse model. Here, tail vein administration of **26** produced a fluorescent signal output corresponding to theranostic probe activation by endogenous H_2_O_2_ in tumor tissues. Further, enhanced fluorescence output was observed in the lipopolysaccharide (LPS, 10 μL, directly injected into tumor tissue) treated group, consistent with oxidative stress-mediated theranostic activation. Theranostic probe **26** showed preferential tumor accumulation and promoted increased tumor reduction as compared to other test groups (PBS as control and 5′-FU), respectively.

Shabat and co-workers used a NIR fluorophore (Cy7) to monitor the drug activation and localization with a level of precision commensurate with in vivo applications.^154^ Toward this end, these researchers created the theranostic agent **27** (Figure 12) composed of boronic ester tagged covalently to a cyanine-based fluorophore and CPT. Incubation with H_2_O_2_ resulted in the cleavage of the boronate moiety, followed by a reaction cascade to release the active CPT over approximately 90 min with a concomitant release of free Cy7 and production of an enhanced fluorescence signal with a maximum at 720 nm. In human glioblastoma multiform (GBM) U87 cells, **27** showed good cytotoxicity upon treatment with H_2_O_2_ (IC_50_ = 40 nM), but not so in the absence of H_2_O_2_ (IC_50_ = 250 nM). The authors noted that the theranostic agent exhibited lower toxicity as compared to the parent drug (CPT, IC_50_ = 20 nM); however, considering the reduced off-target side-effects of theranostic **27**, it was deemed of potential benefit in the context of, e.g., personalized therapy. Further animal-based studies confirmed that theranostic probe **27** provided for a distinct fluorescence enhancement (Cy7), which was taken as signaling CPT release, when subject to either intratumoral or intravenous administration. An ostensibly related NIR-based theranostic probe **28** was developed for H_2_O_2_-mediated release and monitoring of diclofenac (a nonsteroid anti-inflammatory drug, NSAID) delivery in inflammation-induced macrophages.^155^

While phenyl boronic ester-based linkers have been widely used for H_2_O_2_-mediated theranostic development, their instability under acidic conditions potentially limits their utility.^156^ This might result in unanticipated drug activation in acidic cellular compartments, such as the lysosomes and endosomes.^157^ To address this putative stability issue, a thioketal-based linker was developed by Yan Li^158^ and Nam^159^ groups. Per the authors’ design expectations, this linker was found to possess better stability in both acidic and basic milieus. Thioketals are oxidatively cleaved to produce the corresponding thiols and ketones and their use in drug delivery systems has already been the subject of several investigations.^158,159^

As noted above, H_2_O_2_ is predominantly produced in the mitochondria. Thus, any theranostic that fails to accumulate effectively in the mitochondria of cancer cells may not produce a robust anticancer response. Landfester and co-workers showed that the direct in situ generation of H_2_O_2_ inside cancer cells might be one way to overcome this bottleneck. These researchers developed a multicomponent theranostic probe **29** (Figure 12), composed of celecoxib-tagged SN-38 tethered through an H_2_O_2_ responsive thioketal linker. The probe was encapsulated into folate-decorated silica nanoparticles that were further decorated with covalently attached GOX enzymes to give the nanoreactor F-GOX@NR.^160^ Once internalized into folate-positive cancer cells as the result of receptor-mediated endocytosis, the nanoreactor F-GOX@NR consumes glucose to generate H_2_O_2_ via GOX catalysis. Celecoxib tagged SN-38 in its inactive form, binds to COX-2, which facilitates its accumulation within the cytoplasm. The excessive H_2_O_2_ produced through nanoreactor F-GOX@NR + **29** triggers cleavage of the thioketal linker and release of active SN-38. Subsequent topoisomerase inhibition then results in cell death. Control experiments revealed that compared with intracellular H_2_O_2_-mediated drug activation, the nanoreactor system F-GOX@NR + **29** worked synergistically to release the active drug with a significantly improved potency in folate and COX-2 dual positive MCF-7 cancer cells (IC_50_ = 0.14 μM, 24 h) as compared to SN-38 (IC_50_ = 1.2 μM, 24 h) and celecoxib-SN-38 conjugates (IC_50_ = 2.8 μM, 24 h), respectively. Further, the intrinsic fluorescence of SN-38 provides for a ratiometric response (shift in the maximum from 452 to 560 nm in the presence of H_2_O_2_), allowing drug activation and localization to be monitored readily.

#### 2.1.5 Other ROS-Responsive Theranostic Probes

In addition to H_2_O_2_, other ROS, including both ^1^O_2_ and •OH, have also attracted interest as possible release triggers due to their high reactivity. The hydroxyl radical •OH can react with numerous biomolecules, including amino acids, carbohydrates, lipids, and nucleotides, but typically does so in an indiscriminate manner.^161^^1^O_2_, an excited state of O_2_, has a higher oxidative potential than that of the •OH radical.^162,163^ While •OH is produced through H_2_O_2_ decomposition in vivo by copper(I) or iron(II)-mediated catalysis, a process also referred to as the Fenton reaction,^164^^1^O_2_ is primarily produced by PSs (PSs) (see Section 2.2 below).^165^ Both ^1^O_2_ and •OH have shorter half-lives than H_2_O_2_ in biological environments.

**Figure 13:**
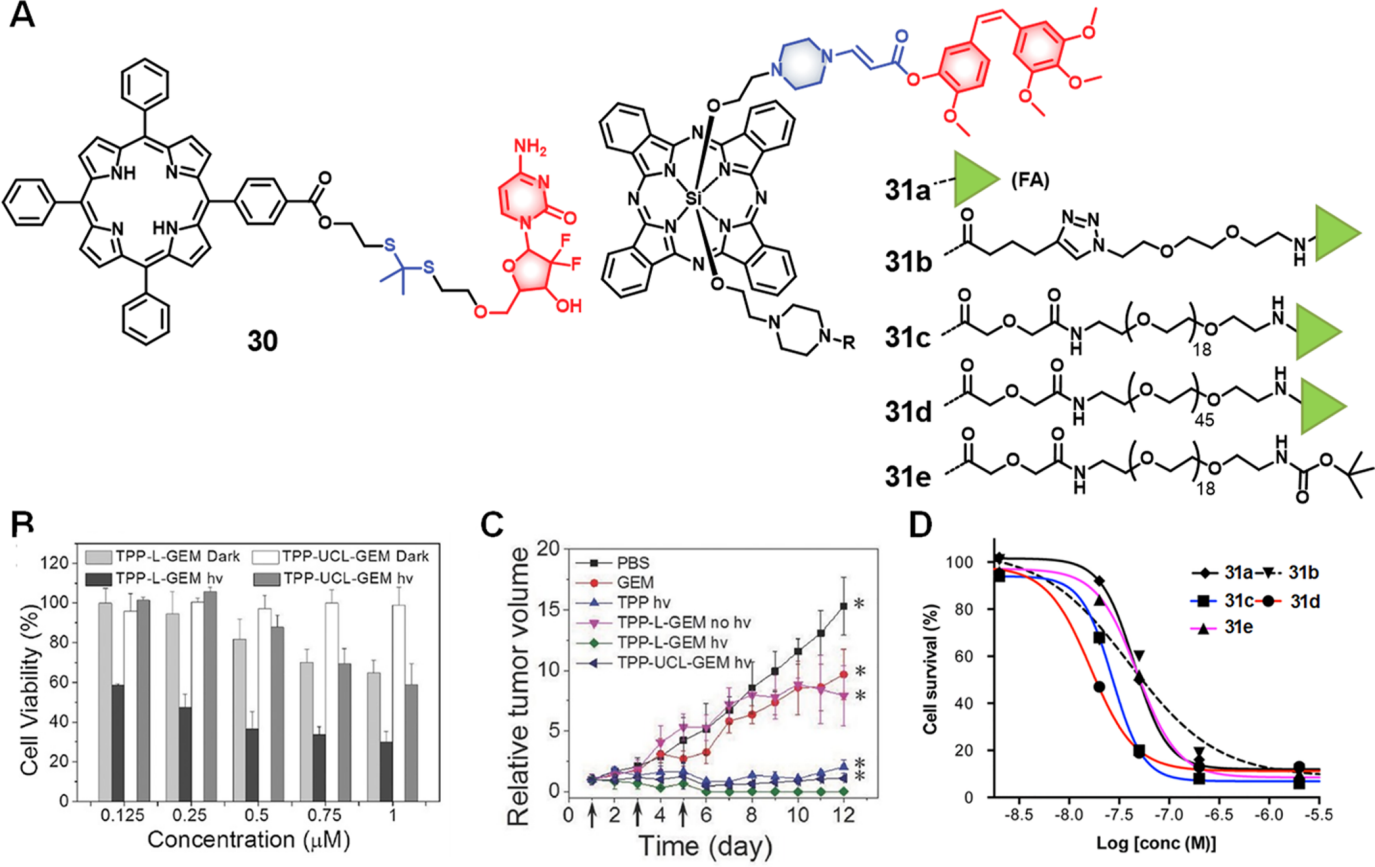
(A) Chemical structures of ROS-responsive theranostic probes (**30** and **31**). (B) Cell viability of treated HeLa cells treated with theranostic **30** (TPP-L-GEM) or control (TPP-UCL-GEM) in the presence and absence of light (280 mW/cm^2^, 1 min). (C) Relative tumor volume in an H22-bearing mouse model after treatment with PBS, GEM, TPP, **30**, and TPP-UCL-GEM with and without light (658 nm, 280 mW/cm^2^, 10 min) (*p < 0.01). Reproduced with permission from ref (166). Copyright 2016 Wiley Intersciences. (D) Cell viabilities of colon 26 cells upon treatment with probes **31a**–**31e** and light illumination (690 nm, 5.6 mW/cm^2^, 30 min) (±SD *n* = 3). Reproduced with permission from ref (170). Copyright 2014 American Chemical Society.

Zhang and co-workers developed theranostic probe **30** (Figure 13) composed of gemcitabine linked to a red light-activatable fluorescent PS (meso-tetraphenylporphyrin; TPP) through a ROS-responsive thioketal linker, which was designed to allow for fluorescence-based image-guided therapy.^166^ Here, the 5′-OH group of the drug was modified to block its anticancer properties. Since ^1^O_2_ has a short half-life and the diffusion rate in aqueous media is very short (≈40 ns and 20–200 nm, respectively), the limited amount of ^1^O_2_ present in unilluminated cells fails to elicit appreciable cytotoxicity.^167^ In contrast, under photoillumination (658 nm, 280 mW cm^–2^), the TPP acts as a PS and generates ^1^O_2_. This results in thioketal bond cleavage followed by cascade-like drug release. The free drug can diffuse to nearby cells and induce toxicity in the unilluminated area as well (so-called bystander effect). Compared to controls (TPP, TPP-UCL-GEM; UCL stands for ROS insensitive alkyl linker), theranostic agent **30** exhibited concentration-dependent toxicity in HeLa cells under illumination (IC_50_ = 0.25 μM). Tumor inhibition studies conducted using subcutaneous H22-bearing mouse models revealed that a PEG_2000_-PLA_2000_-modified version of theranostic **30** (intravenous administration) showed preferential tumor accumulation, and provided for tumor growth inhibition under low-power light illumination. The intrinsic fluorescence of TPP was also exploited to garner information related to the in vivo distribution of this theranostic. Wang and co-workers employed a similar strategy to deliver Dox to cancerous sites in breast cancer mouse models in vivo with minimal side-effects.^168^

Other theranostic agents that rely on ^1^O_2_-responsive activation have been explored. Among these are aminoacrylate-based chemical linkers that are used to tether covalently a PS with a parent drug. For hydroxyl-bearing drug candidates, coupling with the carboxylic acid of an aminoacrylate linker offers a synthetically facile drug-loading platform. Conversely, the amino portion of the acrylate linker may be linked to a PS to furnish theranostic agents. Utilizing this strategy, researchers have developed various delivery systems that rely on SN-38,^169^ combretastine,^170,171^ paclitaxel,^172^ and NSAID (ibuprofen and naproxen)^173^ as the active drug payloads. For example, You et al. reported a series of conjugates **31a**–**e** (Figure 13) consisting of far-red-activatable PSs (silicon phthalocyanine, Pc), covalently connected to an anticancer drug, paclitaxel, via an aminoacrylate chemical linker through the 2′-OH position (a site critical for drug action, tubulin binding).^170^ The resulting conjugate was further modified with folic acid bearing different PEG spacers (1 kDa, 2 kDa, 3.5 kDa, and 5 kDa) to improve their cancer-targeting ability and solubility. Further experiments led to the conclusion that medium-chain PEGylated conjugates (1k to 3.5k) are optimal for achieving folate receptor-mediated uptake. These systems were also found to provide for greater cytotoxicity than a longer PEG chain (5 kDa) or conjugates lacking the PEG linker. Cell-based experiments conducted on colon-26 cancer cells revealed that one of the theranostic agents (**31b**) showed folate receptor-mediated uptake and greater toxicity (IC_50_ = 1.65 nM) upon photoillumination (690 nm) as compared to other conjugates (IC_50_ = 2.71, 4.03, 4.47, and 4.85 nM) under similar conditions (Figure 13).

#### 2.1.6 Enzyme Responsive Theranostic Probes

Enzymes are mostly proteinaceous biomolecules that accelerate chemical reactions both intra- and extracellularly.^174^ Their high substrate specificity, robust response, and cell-specific presence make them of interest as potential chemical triggering agents.^175^ Dysregulation of key enzymes is also a hallmark of pathology across a wide range of diseases, including inflammation,^176,177^ cancer,^178,179^ and neurodegenerative disease.^180,181^ By carefully choosing enzyme-specific substrates it is possible to mask an anticancer drug and create theranostic probes that display target-specific activation, relatively improved stability, and enhanced therapeutic effects. A summary of work along these lines now follows.

##### 2.1.6.1 DT-Diaphorase Responsive Theranostic Probes

Quinone is a common subunit in many natural products, as well as a range of synthetic and semisynthetic molecules, including anticancer agents, antimicrobial drugs, dyes, vitamin K, and enzyme cofactors.^182^ Quinones play key roles in redox cycling due to their reducible nature. Quinone reductase 1 (NQO1), also referred to as DT-diaphorase, is a two-electron reductase localized primarily in the cell cytosol and at lower levels in the endoplasmic reticulum and the mitochondria.^183,184^ DT-diaphorase is involved in detoxification processes and is associated with early carcinogenic events with elevated levels being found in several cancer types, including ovarian, thyroid, breast, colon, and pancreatic cancers.^185^ Hence, the higher level of DT-diaphorase in cancerous tissues over normal healthy tissues have been used as an endogenous trigger for tumor-specific drug delivery applications.

**Figure 14:**
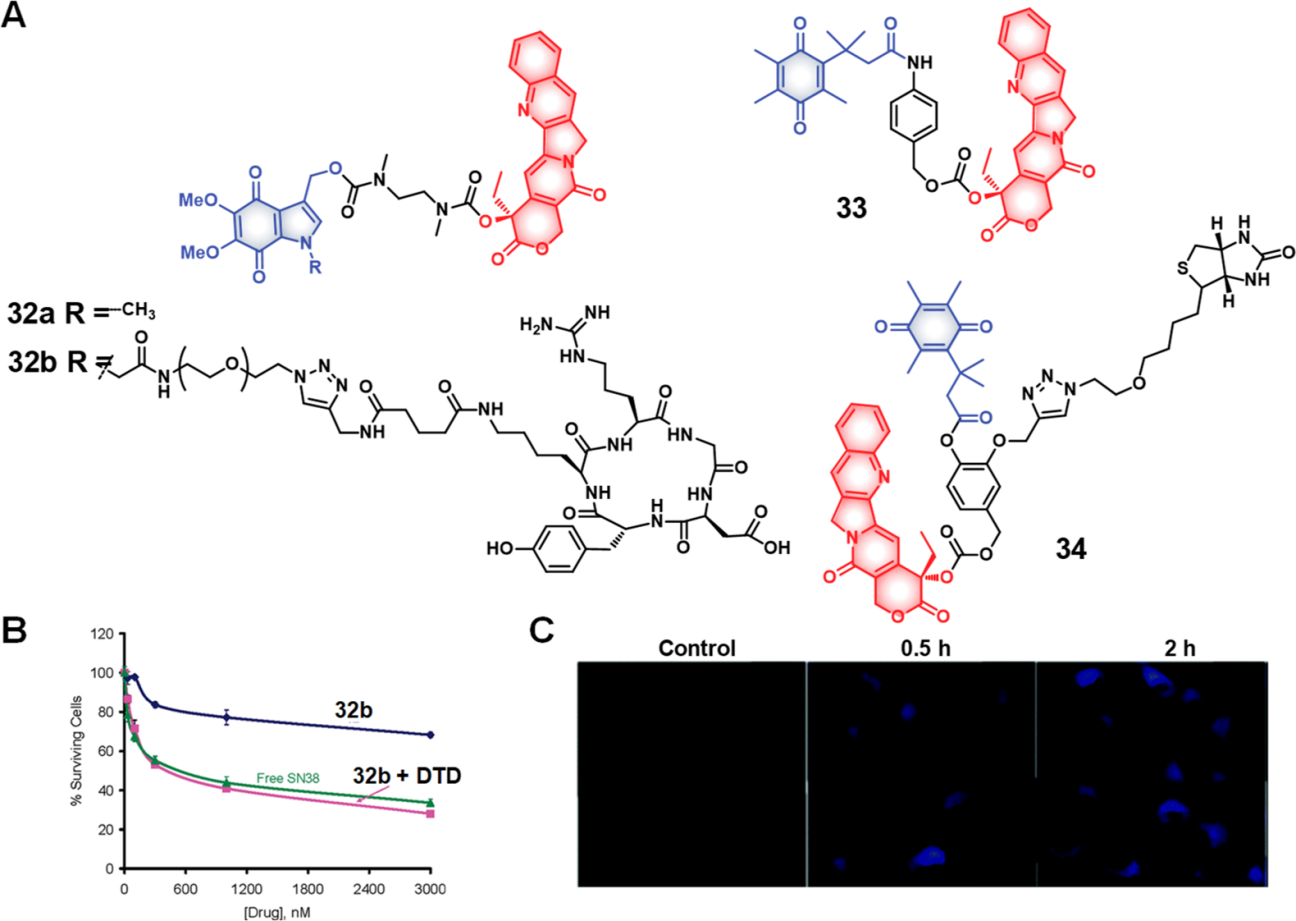
(A) Chemical structures of DT-diaphorase responsive theranostic probes (**32**–**34**). (B) Cell viability of theranostic probe **32b**, SN-38, and **32b** + DTD in KB cells. Reproduced with permission from ref (187). Copyright 2010 American Chemical Society. (C) Fluorescence images of A549 cells in the absence and presence of theranostic probe **33** (10 μM, 2 h) as recorded at different time points. Reproduced with permission from ref (190). Copyright 2015 Royal Society of Chemistry.

Indolequinone is a recognized substrate for NQO1. Appreciating this, Nishimoto and co-workers prepared the theranostic agent **32a** (Figure 14) designed to deliver the cytotoxin SN-38 to cancer cells.^186^ DT-diaphorase mediated-reduction was then expected to release the active SN-38 drug with concomitant fluorescence changes. To improve the cancer-selective uptake, theranostic agent **32b**, composed of an SN-38 moiety linked to indolequinone and an integrin-selective peptide as a cancer-targeting unit was prepared.^187^ Theranostic agent **32b** exhibited preferential uptake in α_v_β_3_ integrin-positive cancer cells with presumed DT-diaphorase-mediated reduction serving to release the active SN-38 and produce 50–70% cancer cell growth inhibition in a human cervical carcinoma (KB) cell line. Studies showed that the alkenyliminium intermediate that is formed after drug release is effective for DNA-alkylation, which was thought to account for the observed cytotoxicity.^188^

Several researchers have explored quinone-based prodrugs with the goal of achieving better drug release profiles. In this regard, a quinone with a “trialkyl lock” produced by Wang and co-workers has garnered particular attention.^189^ Under DT-diaphorase-mediated reduction conditions, the quinone is converted to the corresponding hydroquinone, thereby enabling a hydroxyl moiety to form a six-membered ring with a carbonyl moiety through lactonization. This results in the smooth release of the drug payload attached through the carbonyl group of hydroquinone. This strategy is embodied in theranostic agent **33** reported by Wu et al., which is designed to deliver SN-38 to cancer cells.^190^ The intrinsic fluorescence of SN-38 facilitated the simultaneous monitoring of active drug release and therapeutic action (Figure 14). Later, Kim et al. used an additional functional group to tag biotin as a cancer-targeting unit in the context of theranostic **34**,^191^ a system that displayed preferential cancer cell uptake, as well as an improved therapeutic efficacy following presumed drug activation.

##### 2.1.6.2 Azoreductase Responsive Theranostic Probes

Azoreductases are flavin-dependent enzymes found in eukaryotic and bacterial organisms. They are for the most part cytosolic enzymes that play an important role in homeostasis. They mediate the reduction of substrates in the presence of an electron donor, typically NADH or NADPH. Azoreductases are overexpressed in many cancer types, such as lung,^192^ breast,^193^ and pancreatic cancers.^194^ As a result, efforts have been made to develop theranostic agents based on azoreductases.

**Figure 15:**
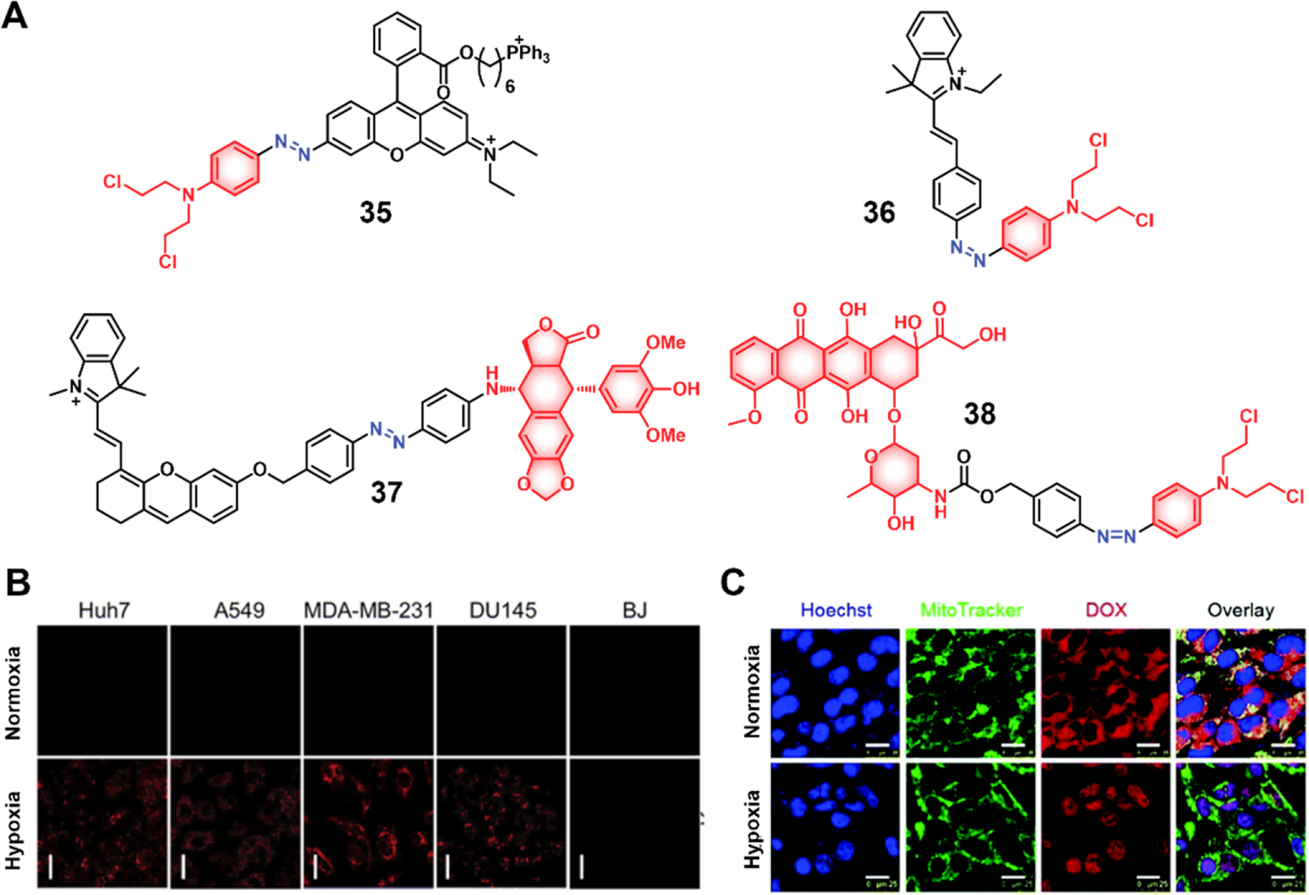
(A) Chemical structure of azoreductase-responsive theranostic probes (**35**–**38**). (B) Fluorescence images of theranostic probe **35** under normoxic (21%) and hypoxic (3%) conditions in various cell lines (scale bar = 10 μm, excitation = 555 nm, emission = 585 nm). Reproduced with permission from ref (195). Copyright 2017 Elsevier Ltd. (C) Confocal images of 4T1 cancer cells recorded following treatment with theranostic probe **38** (20 μM) under normoxic and hypoxic conditions after staining with Hoechst 33342 and MitoTracker Green (scale bar = 20 μm). Reproduced with permission from ref (198). Copyright 2018 Royal Society of Chemistry.

Kim et al. developed theranostic agent **35** (Figure 15) for the targeted delivery of a chemotherapeutic drug to the mitochondria of cancer cells.^195^ System **35** is composed of a rhodamine 123/B analogue conjugated to a *N*,*N*′-bis(2-chloroethyl)-1,4-benzenediamine, which serves as a nitrogen mustard analogue, through an azo linkage. Additionally, a lipophilic triphenyl phosphonium moiety is included in the overall construct to provide for mitochondrial targeting. Upon reduction, the azo bond is cleaved to release simultaneously the active drug and a fluorescent reporter, thereby providing a tool to monitor drug activation and localization under hypoxic conditions in cancer cells. However, a complex multistep synthesis and poor solubility resulted in a low translational potential for **35**. To address these limitations, Xie developed theranostic **36** (Figure 15), where the positive charge on the fluorophore assured mitochondrial targeting, and the lower apolar surface area enabled a better aqueous solubility.^196^

Shi and co-workers used a similar strategy to develop theranostic agent **37** (Figure 15). This system relies on Cy as a NIR fluorescent reporter and proved useful for in vivo drug monitoring.^197^ Likewise, Yu et al. developed a theranostic system **38** (Figure 15) composed of nitrogen mustard and Dox linked through an azo bond.^198^ In reductive environments, both drugs were released with concomitant fluorescence enhancement being seen that was ascribed to free Dox. This increase in emission intensity was utilized to monitor drug activation and cellular localization. In vitro and in vivo studies involving 4T1 cell-based models served to confirm that theranostic agent **38** has improved cytotoxicity and displays reduced side-effects, as compared to controls (i.e., PBS, free Dox).

##### 2.1.6.3 Nitoreductase Responsive Theranostic Probes

Nitroredutases (NTRs) are flavin mononucleotide (FMN)-containing enzymes that are overexpressed in several tumor types. The levels of NTR expression are closely related to hypoxia in solid tumors.^199^ Type 1 NTRs are mostly found in bacteria and are oxygen-insensitive enzymes containing FMN as the active center, while type 2 NTRs are oxygen-sensitive, and contain FMN or flavin adenine dinucleotide.^200^ Hypoxic regions in tumors arise from impaired vascular networks or those insufficient to support rapid growth, resulting in limited blood and oxygen supply. Hypoxia is observed in 50–60% of solid tumors, particularly the inner core of tumors.^201^ Hypoxia-responsive DDS have been thoroughly investigated and are the topic of a number of published articles.^202−205^ In this section, we summarize recent advances involving hypoxia-responsive DDS in the area of molecular theranostics.

One of the biggest challenges in hypoxia-responsive therapeutic formulations is tumor angiogenesis, which acts to enhance the tumor oxygen supply, resulting in low therapeutic efficacies. Hence, blocking angiogenetic pathways in combination with hypoxia-responsive drug delivery systems could lead to the production of improved hypoxia-responsive DDS.

**Figure 16:**
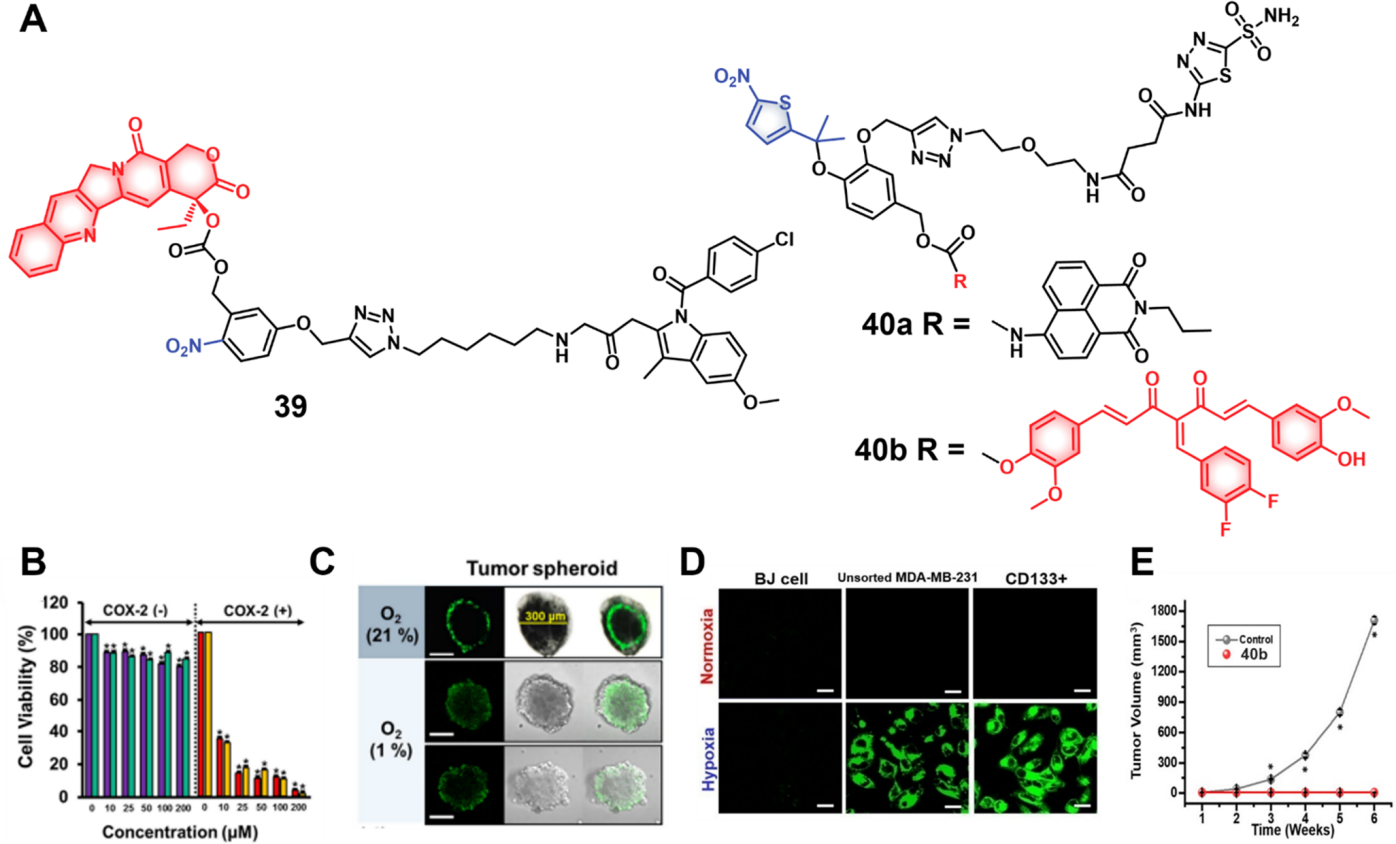
(A) Chemical structures of nitroreductase-responsive theranostic probes (**39** and **40**). (B) Cell viabilities of theranostic probe **39** in COX-2 positive (A549, HeLa) and COX-2 negative (WI-38, BJ) cells at different concentrations. (*p < 0.05). (C) Fluorescence images (at day 4) of HeLa cell tumor spheroids under normoxic and hypoxic conditions after treatment with probe **39** (25 μM). Reproduced with permission from ref (206). Copyright 2018 Elsevier Ltd. (D) Fluorescence images of different cell populations recorded after treatment with **40a** under normoxic and hypoxic conditions (3% oxygen) (scale bar = 50 μm). (E) Tumor volume vs time plot of CD^+^133 MDA-MB-231 cells after treatment with DMSO or **40b** (5.0 nM, 24 h) and then administered to mice to gauge tumorigenesis. Reproduced with permission from ref (207). Copyright 2021 American Chemical Society.

Nitroaromatics serve as excellent substrates for both NTRs, resulting in the formation of hydroxylamines and amine derivatives. When presented within a self-immolative system, the subsequent electron redistribution in the aromatic ring results in the release of linked drugs. Appreciating this, Kim et al. developed theranostic agent **39** (Figure 16) composed of an NSAID (indomethacin) linked to SN-38 through a nitrobenzyl alcohol-based linker.^206^ The incorporation of indomethacin in the theranostic design satisfied two roles; to achieve tumor targeting based on COX-2 overexpression, as well as angiogenesis inhibition mediated by COX-2. Theranostic agent **39** showed concentration-dependent toxicity in COX-2 expressing A549 and HeLa cells under hypoxic conditions (1% O_2_). Further, theranostic probe **39** exhibited a strong fluorescence corresponding to active SN-38 in multicellular tumor spheroids (A549) of varying sizes (110, 235, 300 μm). This finding was taken as evidence of deep tissue penetration and prodrug activation through indomethacin-mediated antiangiogenesis.

The same group developed theranostic pair **40a** and **40b** for imaging and therapy of cancer stem cells (CSCs) (Figure 16).^207^ Here, the authors used dimethylnitrothiophene as the hypoxia-responsive trigger instead of the *p*-nitrobenzyl group, owing to its lower reduction potential, which was expected to facilitate drug release. For therapeutic purposes, a curcumin analogue, 3,4-difluorobenzylidene curcumin, was used. Further, active targeting of CSCs was achieved via a well-known carbonic anhydrase (CAIX) inhibitor, acetazolamide.^208^ For imaging, a naphthylamide fluorophore was employed in lieu of the drug. The main difference within this effective theranostic pair (designed to allow separate imaging and therapy) was the presence of the drug or the phthalimide unit with the targeting unit and hypoxia-responsive trigger entities remaining the same. Because of this similarity, it was assumed that the cellular uptake behavior and activation profile of this theranostic pair would be comparable. Support for this supposition came from cell-based studies using CD^+^133 MDA-MB-231 breast cancer cells. Both compounds (**40a** and **40b**) demonstrated hypoxia-sensitive activation allowing for imaging and anticancer activity. Treatment with theranostic **40b** (tail vein delivery) retarded tumor development in CD^+^133 MDA-MB-231 xenograft mice compared to the control.

##### 2.1.6.4 Esterase Responsive Theranostic Probes

**Figure 17:**
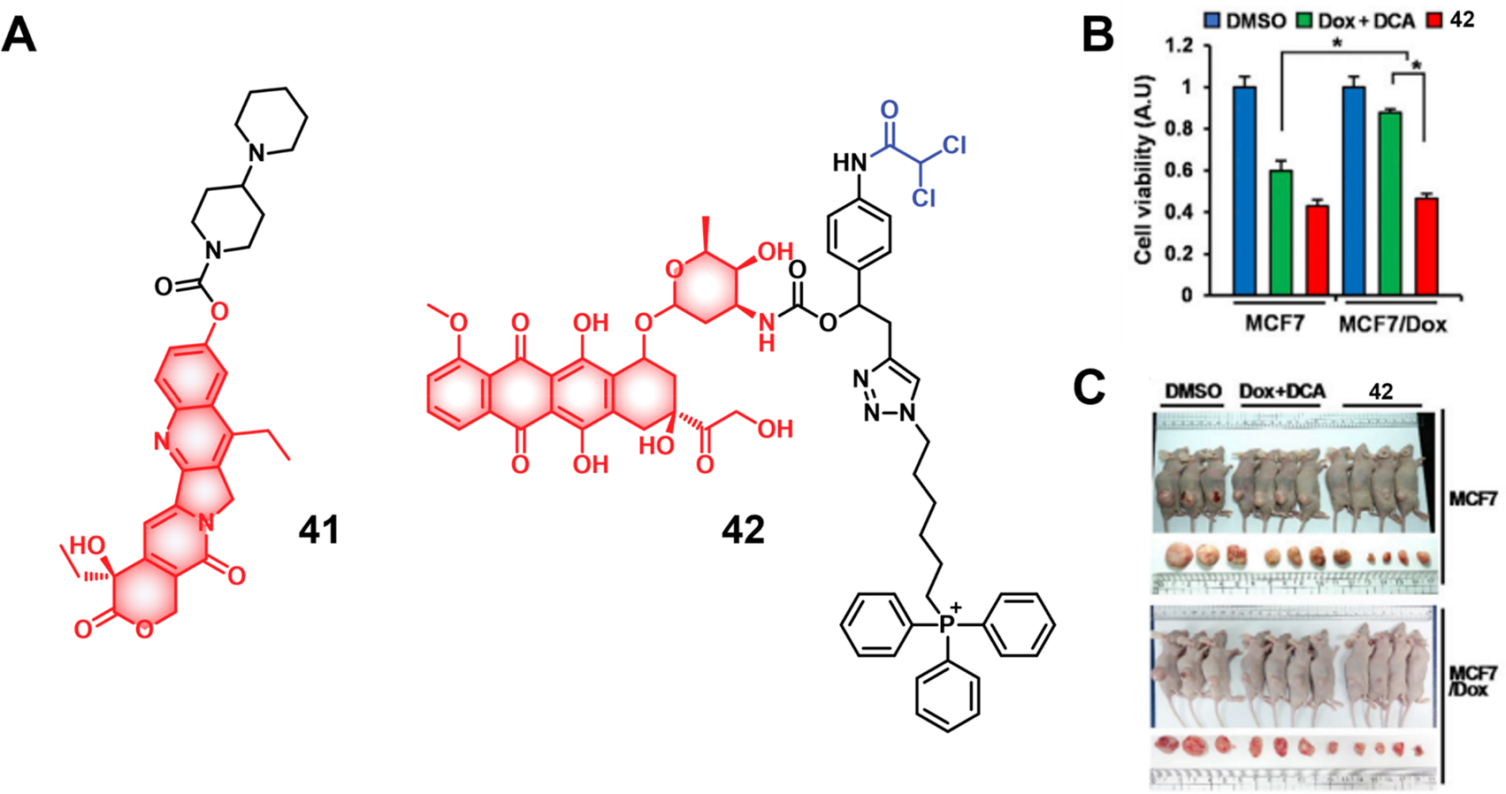
(A) Chemical structures of esterase-responsive theranostic probes **41** and **42**. (B) Cell viabilities of Dox-sensitive MCF7 and Dox-resistant MCF7/Dox cells after treatment with DMSO, Dox + DCA (1:1), or theranostic probe **42**. (C) Representative images of MCF7 and MCF/Dox xenograft tumor models after treatment with DMSO control, Dox + DCA (1:1), or theranostic probe **42**, respectively. Reproduced with permission from ref (223). Copyright 2018 Elsevier Ltd.

Carboxylesterases (CESs) are a common class of hydrolases that promote ester, amide, and carbamate bond cleavage.^209,210^ CESs are key protective enzymes that play a role in detoxifying xenobiotics. Two prominent CEs, namely CES1 and CES2, have been thoroughly studied. Several reports have highlighted the elevated expression of CES2 in pathological tissues as compared to healthy tissues.^211,212^ Overexpressed CES levels in cancer cells are thought to abet invasion, migration, survival, and tumor growth.^213,214^ Compared to normal cells (0.17 ± 0.09 U/L male; 0.12 ± 0.07 U/L female), about a 2–4-fold increase in CES activity in malignant colorectal cancer (0.45 ± 0.25 U/L male; 0.45 ± 0.35 U/L female) has been reported.^215^ Hence, several esterase-responsive theranostic agents have been developed to achieve tumor-specific imaging and improve therapeutic outcomes. For example, Kunimoto and co-workers developed theranostic agent **41** as a CEs-responsive SN-38 analogue (Figure 17).^216^ The authors used a water-soluble δ-lactone ring linked to the SN-38 drug through a carbamate linker. Incubation with CES resulted in hydrolysis of the carbamate bond to release SN-38.

One of the biggest challenges associated with cancer chemotherapy is the development of multidrug resistance (MDR), a state defined as the ability of cancer cells to survive despite the presence of various chemotherapeutics.^217^ Statistically, about 90% of cancer mortality is attributed to MDR.^218^ MDR is predominantly attributed to several factors such as reduced drug uptake, enhanced drug efflux, increased DNA repair, elevated xenobiotic metabolism, and genetic factors (gene amplifications, mutations, and epigenetic alterations).^219−222^ This results in reduced therapeutic potency of the administrated drugs. To address this limitation, Kim and co-workers developed theranostic agent **42** (Figure 17), composed of a dichloroacetic acid (DCA) moiety linked through an amide linkage to a self-immolative linker, Dox, and a lipophilic TPP mitochondrial targeting unit.^223^ The rationale behind this design was to sensitize cancerous cells by shifting the aberrant metabolism of cancer cells, which rely largely on glycolysis, back to mitochondrial phosphorylation. It was also appreciated that the delayed release of active Dox would likely lead to improved therapeutic efficacy. DCA is a well-known pyruvate dehydrogenase inhibitor (PDK) used to facilitate the shunting of glycolysis to glucose oxidation. It thus reduces lactate accumulation, reduces intracellular ATP, and mitochondria dysfunction.^224^ Another aspect of **42** is that it was expected to initially be located within the mitochondria, thus avoiding initial drug efflux through ATP-driven ABC transporters.^225^ Over time, Dox was expected to translocate to the nucleus to produce the desired anticancer activity. Cell-based studies confirmed that the cytotoxicity was CEs-dependent and that **42** was preferentially taken up by A549 and HepG2 cancerous cells over normal cells (NHDF, IMR90). Theranostic agent **42** outperformed Dox and a coadministered 1:1 combination of DCA and Dox. Moreover, **42** was able to perform its action in both drug-sensitive MCF and drug-resistant MCF/Dox breast cancer cells, as well as in both drug-sensitive and resistant MCF xenograft mouse models.

##### 2.1.6.5 Protease Responsive Theranostic Probes

Proteases are a large family of enzymes that promote the cleavage of peptide bonds.^226^ They play a vital role in protein metabolism (protein catabolism, protein digestion, and cell signaling), and elevated protease levels are closely associated with several diseases, including inflammatory disease,^227^ cancer,^228,229^ cardiovascular,^230^ and neurodegenerative disease.^231^ To date, considerable effort has been devoted to the development of protease inhibitors, as well as to protease-responsive diagnostic and therapeutic probes. Caspases are among the proteases involved in inflammation, cancer, and programmed cell death. Specifically, caspase-3, a cysteine-aspartic acid protease is activated through endogenous (programmed cell death) and exogenous (radio/chemotherapy treatment) means.^232−234^

**Figure 18:**
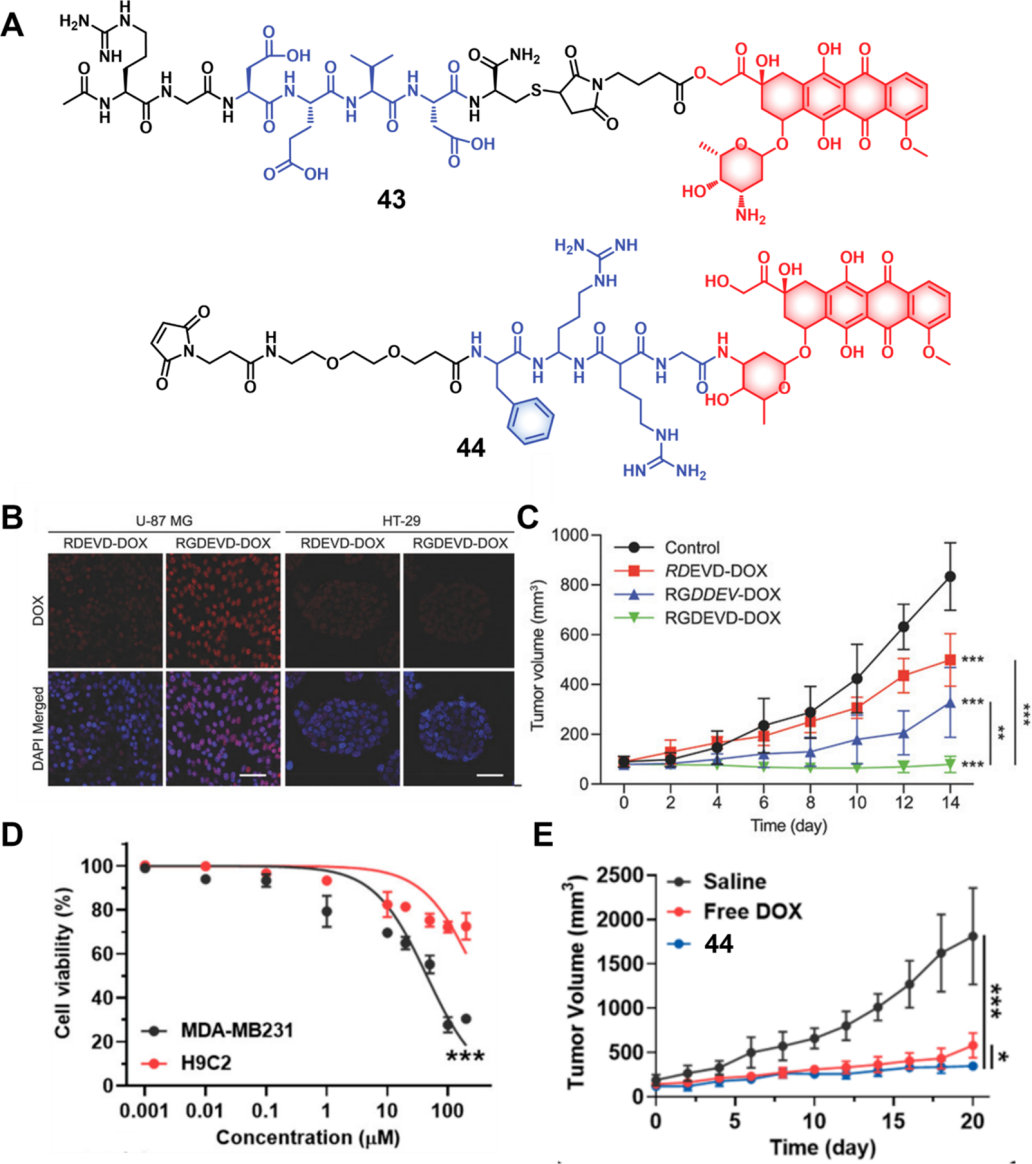
(A) Chemical structures of theranostic probes **43** and **44**. (B) Confocal images of U-87 MG and HT-29 cancer cells recorded upon treatment with RDEVD-DOX (control) and RGDEVD-DOX (**43**). The red color is ascribed to doxorubicin fluorescence while the blue color indicates the cell nucleus (scale bar = 50 μm). (C) Tumor inhibition of U-87 MG xenografted mice after treatment with saline, RDEVD-DOX, RGDDEV-DOX, or **43** (RGDEVD-DOX), respectively (*n* = 6) (±SD * *p* < 0.05, **p < 0.01, *** *p* < 0.001 vs control). Reproduced with permission from ref (235). Copyright 2018 Wiley Intersciences Ltd. (D) Cell viability of theranostic probe **44** in MDA-MB-231 and H9C2 cells (*** *p* < 0.001). (E) Tumor inhibition of MDA-MB-231 tumor-bearing mice after treatment with saline, free Dox, or theranostic probe **44** (* *p* < 0.05, *** *p* < 0.001). Reproduced with permission from ref (241). Copyright MDPI.com.

Byun et al. developed the caspase-3-responsive theranostic agent **43** (Figure 18) composed of a cancer-targeting integrin (Arg-Gly-Asp) tripeptide, a caspase-3-responsive linker (DEVD, Asp-Glu-Val-Asp tetrapeptide), a cellular ester responsive linkage and Dox, as the anticancer drug.^235^ Dox is known to activate caspase-3, an apoptosis related marker.^236^ As a result, it was expected that Dox-induced activated caspase-3 would release Dox after ester hydrolysis, resulting in additional caspase-3 activation. This cycle was expected to continue to exert an improved therapeutic efficacy. Per these expectations, caspase-3 was found to induce cleavage of the DEVD linker, followed by ester bond hydrolysis to release the active drug, along with concomitant fluorescence enhancement. In integrin-positive human glioma U-87MG cells, theranostic **43** exhibited preferential uptake ascribed to integrin-mediated endocytosis and demonstrated an improved cytotoxicity as compared to integrin-negative HT-29 colon cancer cells. Cell-based studies confirmed that this self-amplified enzyme apoptosis theranostic agent induced a roughly 154-fold enhancement in caspase-3 activity. In vivo studies conducted using U-87 MF tumor-bearing mice revealed that treatment with **43** produced a near-complete tumor inhibition as compared to various controls.

Cathepsins are lysosomal proteolytic enzymes that primarily metabolize proteins and peptides. They play a fundamental role in maintaining tissue homeostasis and are involved in the immune response, as well as cell development, differentiation, and apoptosis.^237^ Alteration in expression levels of these enzymes is correlated with several pathological disorders, including cancer, and poor prognoses.^238,239^ Cathepsin B is especially important in terms of promoting proteolysis in the extracellular matrix (ECM), thereby promoting tumor angiogenesis, invasion, and metastasis.^240^ Special efforts have therefore been made to develop cathepsin B-responsive diagnostic and therapeutic platforms. For example, Kim et al. developed theranostic agent **44** (Figure 18), composed of Dox linked to an albumin-binding maleimide moiety through a cathepsin B-responsive peptide sequence (FRRG).^241^ Compared with the parent drug (Dox itself; *t*_1/2_ = 0.25 h), the maleimide moiety in the scaffold was found to improve the half-life (*t*_1/2_ = 3.1 h), presumably as a result of being bound to the plasma albumin. After presumed albumin-mediated passive tumor accumulation, theranostic **44** is activated by cathepsin B to release Dox inside the cancer cells. Cytotoxicity studies revealed that theranostic **44** exhibited enhanced antitumor effects in MDA-MB231 breast cancer cells (IC_50_ = 7.33 μM) as compared to rat cardiomyocytes H9C2 (IC_50_ > 200 μM), a result ascribed to the higher cathepsin B activity (24.26 ± 3.08-fold) in the former cells. In vivo studies in MDA-MB231 tumor-bearing mice demonstrated tumor growth inhibition in the theranostic **44-**treated group as compared to various controls.

**Figure 19:**
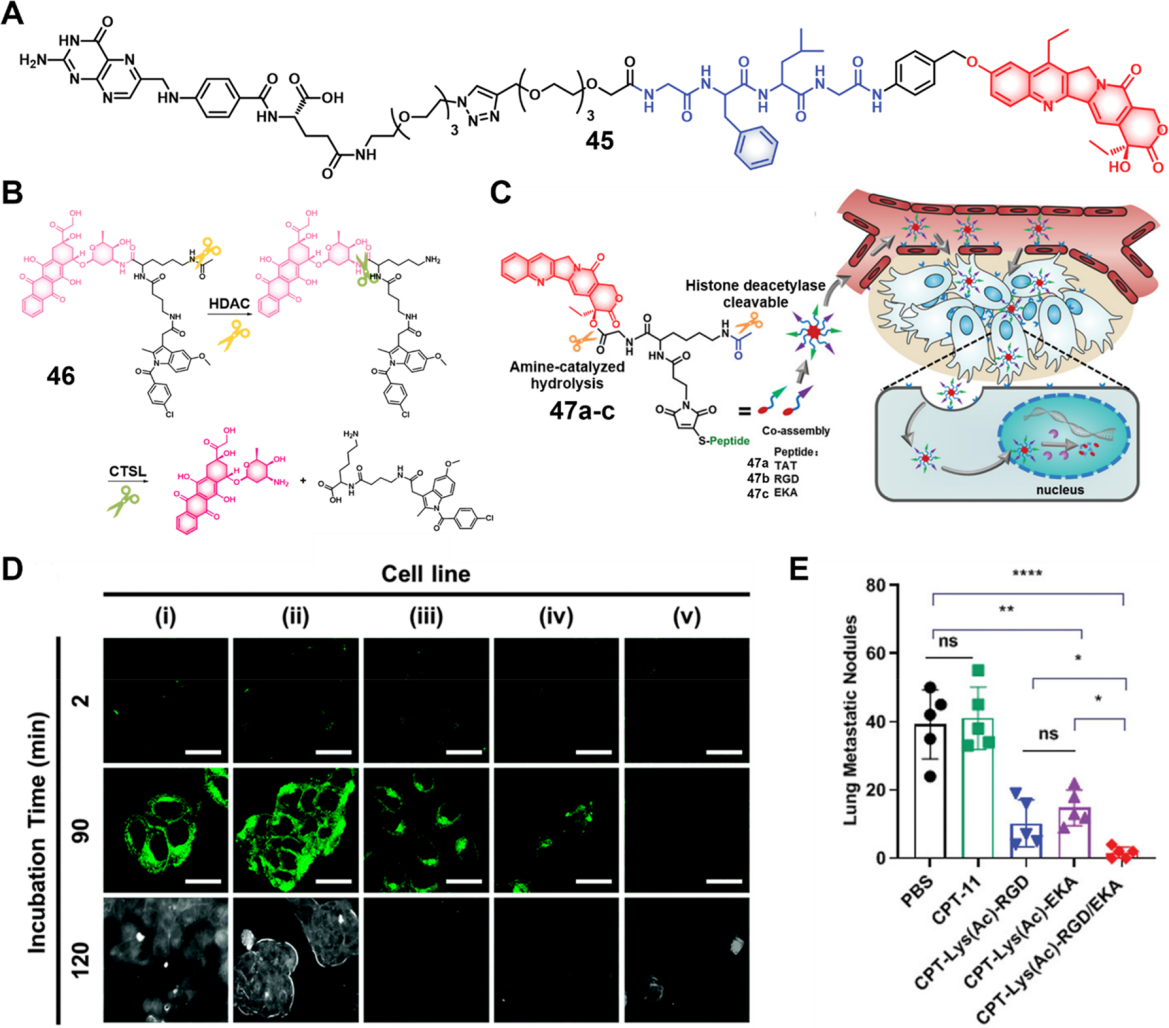
(A) Chemical structures of theranostic probes **45**–**47**. (B) Schematic showing the activation of theranostic probe **46** and drug release. (C) Chemical structure and mode of activation proposed for theranostic probes **47a**–**c** and their preferential accumulation in tumor tissues via active and passive tumor targeting and nucleus accumulation to release the specific drug payload. (D) Time-dependent fluorescence images of theranostic probe **46** in (i) HeLa, (ii) HepG2, (iii) HCT116, (iv) MIA PaCa-2, and (v) Caco-2 cells. Reproduced with permission from ref (248). Copyright 2016 Royal Society of Chemistry. (E) Antimetastatic activity of **47** and controls showing a number of pulmonary metastatic nodules at the experimental end point (*n* = 5). Reproduced with permission from ref (249). Copyright 2023 Wiley Intersciences.

In a separate work, Tian et al. developed theranostic agent **45** (Figure 19) for the cathepsin B-mediated delivery of SN-38 within folate-positive cancer cells.^242^ Through folate receptor (FR)-mediated endocytosis, the theranostic agent was taken up in FR-positive cancer cells (SK-Hep-1, HeLa, and Siha cells). Further, cathepsin B triggered activation to release the active drug with a concomitant fluorescence enhancement within the nucleus. Cell-based cytotoxicity studies revealed that **45** exhibited significant cytotoxicity in these cancer cells with IC_50_ values of about 2–3 μM being observed. By contrast, low toxicity was seen in normal cells (16-HBE) and FR-negative A549 lung cancer cells (IC_50_ = 20 μM). It was thus proposed that the theranostic agent **45** could be used to avoid the off-target side effects seen for the parent drug (SN-38).

Histone deacetylases (HDACs) are an important class of epigenetic modulators that regulate the activity and expression of numerous proteins in cellular processes. Several studies have found elevated levels of HDAC in close association with various malignancies, including neuroblastoma,^243^ gastric,^244^ ovarian,^245^ colon,^246^ and multiple myeloma,^247^ with poor patient outcomes. Hence, efforts have been made to design HDAC-responsive drug delivery systems. For example, Kim et al. reported theranostic agent **46**, consisting of an acetylated lysine residue linked to Dox and indomethacin as a COX-2 positive cancer-targeting unit.^248^ Preliminary solution studies revealed that **46** on sequential treatment with HDAC and cysteine cathepsin L (CTSL), respectively, resulted in active Dox release with fluorescence enhancement (Figure 19). In cell-based experiments conducted on various cell lines, **46** showed an enhanced fluorescence that was ascribed to the release of Dox. A cell line dependence was seen in the order HepG2, HeLa > HCT116, MIA PaCa-2 > Caco-2 cells that was correlated with their HDAC and CTSL levels, as well as their COX-2 expression levels. Theranostic **46** displayed concentration-dependent cytotoxicity in HeLa cells and preferential tumor localization in COX-2-positive HeLa and HepG2 tumor xenograft mouse models.

In another report involving a dual-mode peptide design strategy, Zheng and co-workers developed a small library of HDAC-responsive theranostic agents **47a**–**c** based on CPT decorated with various peptide sequences for multistage tumor targeting purposes.^249^ The peptides used by the authors were 1) CRGDK, an RGD sequence for targeting α_v_β_3_ integrins in the tumor extracellular matrix,^250^ 2) CREKA for targeting the tumor vasculature,^251^ and 3) TAT to target the nucleus^252^ (Figure 19). Nanoassemblies of these systems were expected to exhibit preferential tumor targeting and HDAC-mediated drug release. Incubation with HDAC1 with these theranostic agents for different time intervals resulted in appreciable CPT release over the course of 1 h (for **47a**: 44.1%, **47b**: 68.2%, and **47c**: 67.7%). Greater than 90% release was seen after 5 h for all three derivatives. These systems exhibited only minimal drug release in the absence of HDAC1, a finding that underscores the high stability and enzyme-selective drug release profile of these systems. All three probes showed significant toxicity in HDAC1-expressing cancer cell lines (**47b**, IC_50_ = 22.3 μM in 4T1 cells, IC_50_ = 0.6 μM in MDA-MB-231 cells; **47a**, IC_50_ = 7.2 μM in 4T1 cells, IC_50_ = 0.2 μM in MDA-MB-231 cells). Using the 4T1 breast tumor mouse model, the authors were able to show that a dual combo (CPT-Lys(Ac)-RGD/TAT) exerted about 72% tumor growth inhibition while **47b** (50%) and **47a** (negligible effect) performed significantly more poorly. Likewise, the nanoassemblies also demonstrated potent antitumor activity in the 4T1-luc orthotopic breast cancer mouse model (metastatic cancer model). This was taken as evidence that both vascular and nucleus targeting could be exploited to improve therapeutic efficacy.

**Figure 20:**
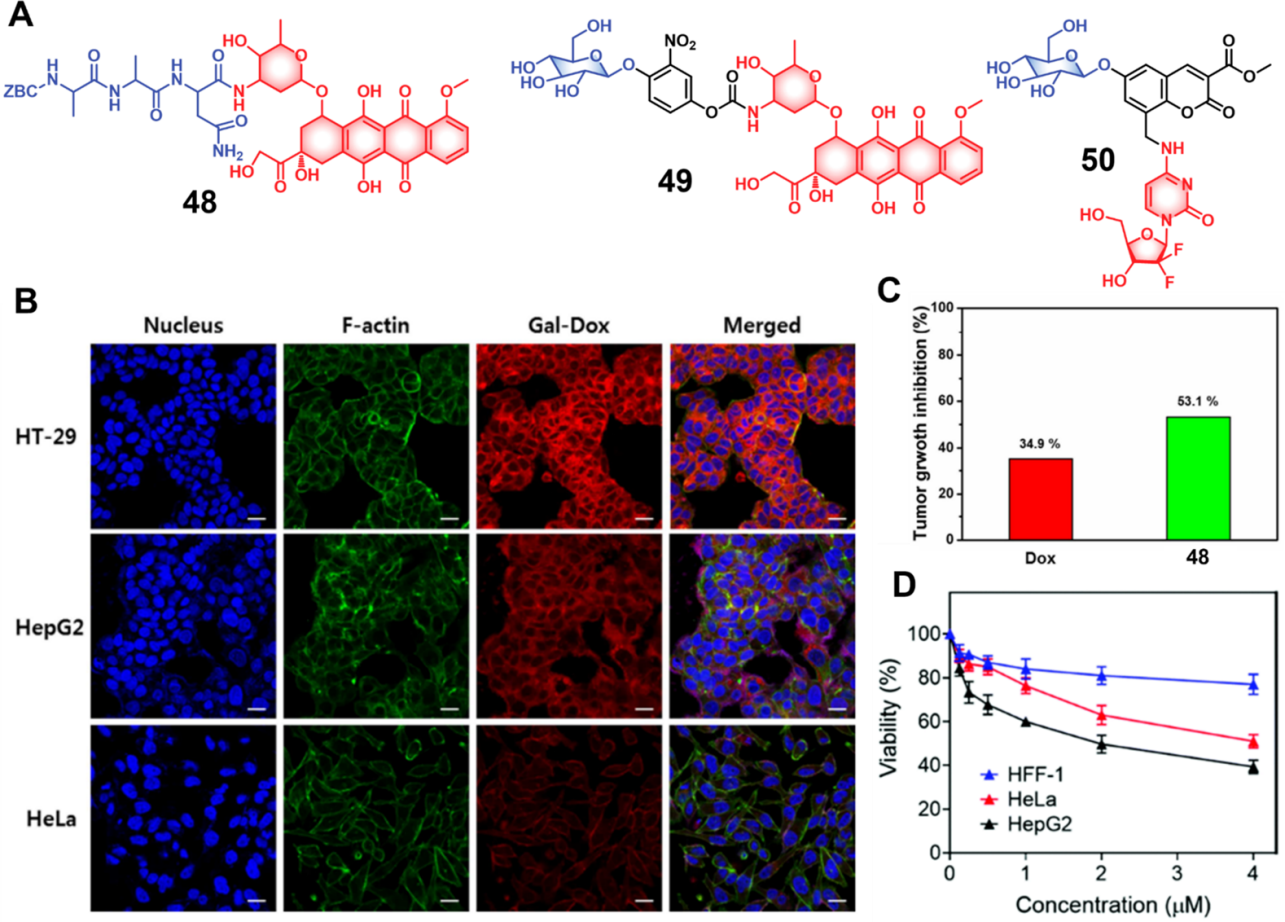
(A) Chemical structures of theranostic probes **48**–**50**. (B) Confocal images of HT-29, HepG2, and HeLa cells after treatment with theranostic probe **49** (10 μM). (C) Tumor growth inhibition of HT-29 xenografts (mouse model) after treatment with Dox and probe **49**. Reproduced with permission from ref (273). Copyright 2018 Elsevier Ltd. (D) Cell viability of HepG2, HeLa, and HFF-1 cells upon treatment with theranostic probe **50** at different concentrations. Reproduced with permission from ref (274). Copyright 2022 Royal Society of Chemistry.

Legumain, a lysosomal/vascular asparaginyl endopeptidases (AEP) enzyme, is a cysteine protease that was originally discovered in legumes; however, it is a lysosomal enzyme in mammals.^253,254^ Legumain is highly expressed in kidney tubulin under physiological conditions and aids renal tubular reabsorption.^255^ Several studies have shown that legumain is highly expressed in a variety of solid tumors such as prostate,^256^ breast,^257^ gastric,^258^ ovarian,^259^ and colon cancers^260^ and is closely associated with risk of malignancy.^261^ Enhanced activity of the enzyme is observed within the acidic microenvironments of cancer cells. To date, efforts have been made to develop cancer-targeted theranostic agents by incorporating a legumain-selective peptide sequence.^262,263^ For example, Riu et al. developed a theranostic agent **48** by using a legumain-selective tripeptide sequence, Ala-Ala-Asn, linked to Dox through an amide linker.^264^ Preliminary activation studies that relied on fluorescence spectroscopy revealed that in the presence of human legumain theranostic **48** (Figure 20) released about 70% of the possible Dox over a 24 h period under acidic conditions (pH 5.5 and 6.5). In contrast, under neutral pH conditions, no drug release was observed. Further confocal studies confirmed that theranostic **48** was taken up by cancer cells through endocytosis and provided for enhanced cytotoxicity and reduced side-effects relative to normal cells. More importantly, this putative theranostic provided for cytotoxicity in both tumor and stromal cells via a “bystander effect”.

Galactosidases are a class of enzymes involved in several vital catabolic processes.^265,266^ β-Galactosidase (β-gal) is a particularly important lysosomal hydrolase that acts to cleave the terminal galactoside residue from glycoconjugates.^267^ β-gal is known to be highly expressed in various cancers^268−270^ and has been used to develop both cancer-targeted imaging and therapeutic agents.^271,272^ In the context of theranostics, Kim et al. developed the β-gal-responsive agent **49** (Figure 20), consisting of an anticancer drug linked covalently to a galactosidase moiety through a self-immolative linker.^273^ As prepared, theranostic agent **49** is stable and nonemissive in PBS. However, when treated with β-gal, hydrolysis of the galactosidase moiety occurs, which results in the release of active Dox with a concomitant fluorescence enhancement. Cell-based studies conducted in HT-29 and HepG2 cells revealed that **49** is taken up preferentially through overexpressed asialoglycoprotein (ASGP) receptors. Reduced uptake was seen in HeLa cells that have lower ASGP expression levels. Probe **49** exhibited a concentration-dependent toxicity in the HT-29 and HepG2 cell lines. In vivo tumor inhibition studies conducted using HT-29 cancer xenograft-bearing mice revealed a strong tumor growth inhibition upon treatment with **49**, as compared to Dox alone.

Buniya et al. developed the theranostic agent **50** (Figure 20), where a β-gal-responsive galactosidase moiety was linked to a coumarin fluorophore, and further connected to gemcitabine.^274^ Incubation with β-gal (0.1 U mL^–1^) served to hydrolyze the galactosidase moiety and trigger an intramolecular electronic rearrangement in the coumarin scaffold thus releasing the active drug and producing a fluorescence enhancement within 30 min. Gemcitabine has a low half-life (*t*_1/2_ = 8–94 min) due to rapid metabolism by intracellular enzymes.^275^ The strategy embodied in **50** was thus expected to enhance drug potency through reductions in the undesired metabolism of gemcitabine. Cytotoxicity studies revealed higher toxicity for theranostic **50** in HepG2 cells (IC_50_ = 1.6 ± 0.4 μM) as compared to free gemcitabine (IC_50_ = 3.5 ± 0.4 μM). Theranostic **50** also proved about 12-fold less toxic in normal HFF-1 cells (IC_50_ = 12.3 ± 0.7 μM).

#### 2.1.7 Dual Stimuli-Responsive Theranostic Probes

As discussed in the above subsections, theranostic probes that are responsive to a single stimulus, such as pH, GSH, H_2_O_2_, enzyme, etc., represent a promising approach to increasing the utility of a given drug while reducing side-effects. It is thus perhaps not surprising that to enhance the further effectiveness and tumor specificity in complicated pathological microenvironments, such as the tumor microenvironment, theranostic probes that rely on dual activation strategies have been developed. In principle, it is possible to design systems where the activation mechanisms occur sequentially in distinct environments or concurrently within the same site of action. In this subsection, we review progress made along both of these limiting directions.

**Figure 21:**
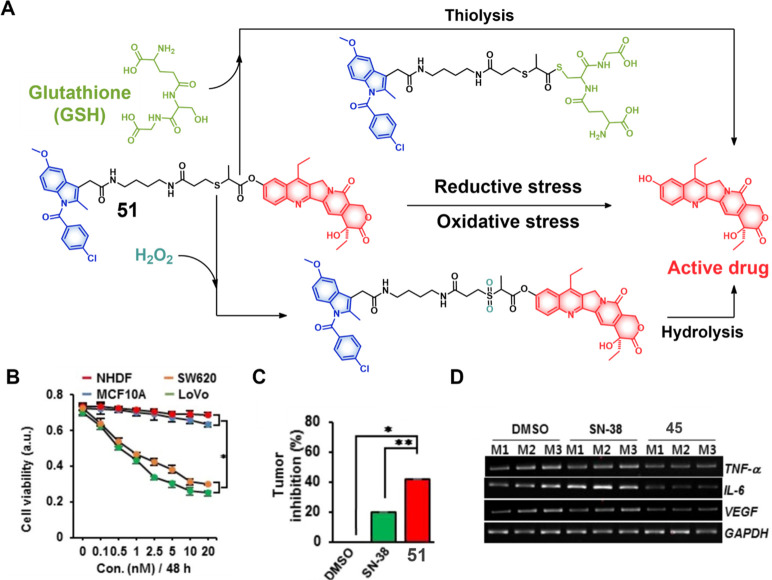
(A) Chemical structure and proposed mode of activation of theranostic probe **51** under conditions of reductive and oxidative stress. (B) Cell viabilities of probe **51** in normal and cancer cell lines were seen upon incubation with different concentrations. (C) Tumor inhibition provided by DMSO and equal concentrations of SN-38 and probe **51** in a SW620 xenograft mouse model (*n* = 5, *p < 0.05, **p < 0.01). (D) Anti-inflammatory cytokine mRNA levels seen in the tissues of mice treated with DMSO, SN-38, and **51** (M1, M2, and M3 define three mice). Reproduced with permission from ref (282). Copyright 2019 American Chemical Society.

Cancerous cells are notoriously heterogeneous. For example, they may constitute reducing environments characterized by elevated levels of intracellular GSH,^276,277^ or be formally oxidative as the result of overproduction.^278−280^ These opposed redox conditions (reductive or oxidative) can exist in different tumors or coexist in the same tumor in different areas, or even in a single cancer cell at different time points.^281^ Recognizing this disparity, Kim and co-workers developed the theranostic agent **51** (Figure 21). This system was designed to enable the redox-responsive delivery of SN-38 under both reductive and oxidative conditions.^282^ Here, a thioether-based linker was used to connect covalently an SN-38 moiety with a COX-2 inhibitor, indomethacin, incorporated into the structure to provide for cancer targeting as well as a potential immunotherapeutic trigger. Preliminary solution studies confirmed that **51** in the presence of GSH underwent thiol-mediated hydrolysis and drug release, while exposure to H_2_O_2_ resulted in sulfone/sulfoxide formation followed by hydrolysis and thus also served to release the active drug. In vitro studies in COX-2-positive LoVo and SW620 cells revealed uptake of **51** and an improvement in the cytotoxicity, relative to COX-2-negative cells (NHDFs, MCF10A). In a colon cancer tumor-bearing mouse model (SW620), treatment with **51** (5 mg/kg/d; intraperitoneal administration) provided for a statistically significant reduction in the tumor burden as compared to controls. Also, as a presumed result of the indomethacin component in **51**, a reduction in key pro-inflammatory markers (IL-6, TNF-α, VEGF) was observed. Other studies have provided support for the conclusion that the incorporation of thioether-based redox-responsive linkers can provide an advantage in the design of functional theranostics.^283,284^

**Figure 22:**
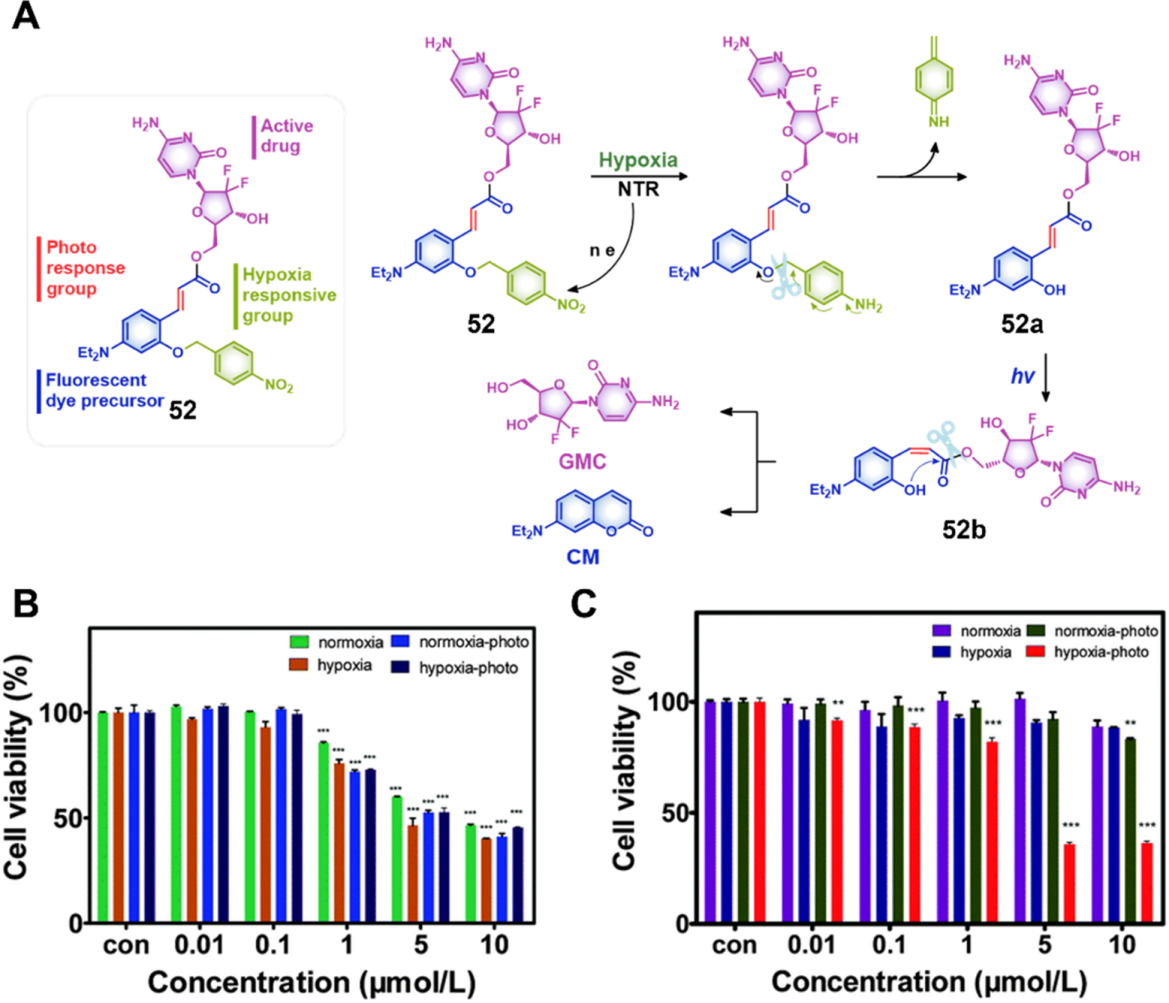
(A) Chemical structure and mode of activation of theranostic probe **52** controlled by hypoxia and sequential light irradiation. Cell viability of MCF-7 cells upon treatment with (B) parent drug GEM and (C) theranostic probe **52** at different concentrations under normoxic and hypoxic conditions in the presence and absence of photoillumination. Reproduced with permission from ref (285). Copyright 2016 Royal Society of Chemistry.

Light-responsive therapies offer the possibility of illumination-based control over drug release and activation. Unfortunately, phototoxicity to the nearby normal tissues, as well as nonspecific drug release due to exposure to sunlight, can limit the potential applicability of this approach. To overcome this possible limitation, Feng and co-workers developed hypoxia- and photoresponsive theranostic probe **52** (Figure 22). This system incorporates a photoresponsive moiety, *o*-hydroxyl *E*-cinnamic acid (CAE), linked to gemcitabine.^285^ The other end of the CAE group is masked with a hypoxia-responsive 4-nitrobenzyl group. Hence, probe **52** cannot be activated by light under normoxic conditions (i.e., ion cells and tumor environments that lack nitroreductase activity). In contrast, in cancer cells, the prevailing hypoxic conditions allow nitroreductase to reduce the 4-nitrobenzyl group, generating intermediate **52a**. This intermediate is sensitive to UV-illumination, generating the active drug through intermediate **52b**. Self-immolative cyclizationleads to a fluorescent coumarin derivative characterized by a blue emission. When tested (10 μM) in MCF-7 cells, a strong blue emission was observed under hypoxic conditions (2% oxygen, 6 h), followed by UV irradiation (365 nm, 10 min). Appreciable cytotoxicity was observed. No such emission or cytotoxicity was observed under normoxic conditions (20% oxygen). Thus, this dual-triggered theranostic probe provides more precise drug delivery to the targeted cancer cells, as well as real-time monitoring of the hypoxic status. As importantly, cytotoxin release can be achieved with high spatiotemporal control. However, analogues of **52** incorporating a red-shifted light trigger and a fluorophore that emits further to the red are needed before systems of this general design are likely to see translation into a clinical setting.

### 2.2 Theranostic Fluorescence Probes in PDT

#### 2.2.1 pH-Activatable Theranostic Probes in PDT

An acidic tumor microenvironment (TME) is considered a characteristic of cancer and provides an opportunity for the development of activatable PSs. Typically, pH-activated PSs contain components that are responsive to H^+^ ions, such as pyridine, aniline, piperazine, morpholine, or other tertiary alkyl amine groups. These components can quench the fluorescence and hinder the generation of photoinduced ^1^O_2_ by the PSs. These smart PSs are usually inactive under normal physiological pH but can exhibit strong fluorescence emission and efficiently produce ROS under acidic conditions. This offers new possibilities for precise identification and PDT treatment of cancers.

**Figure 23:**
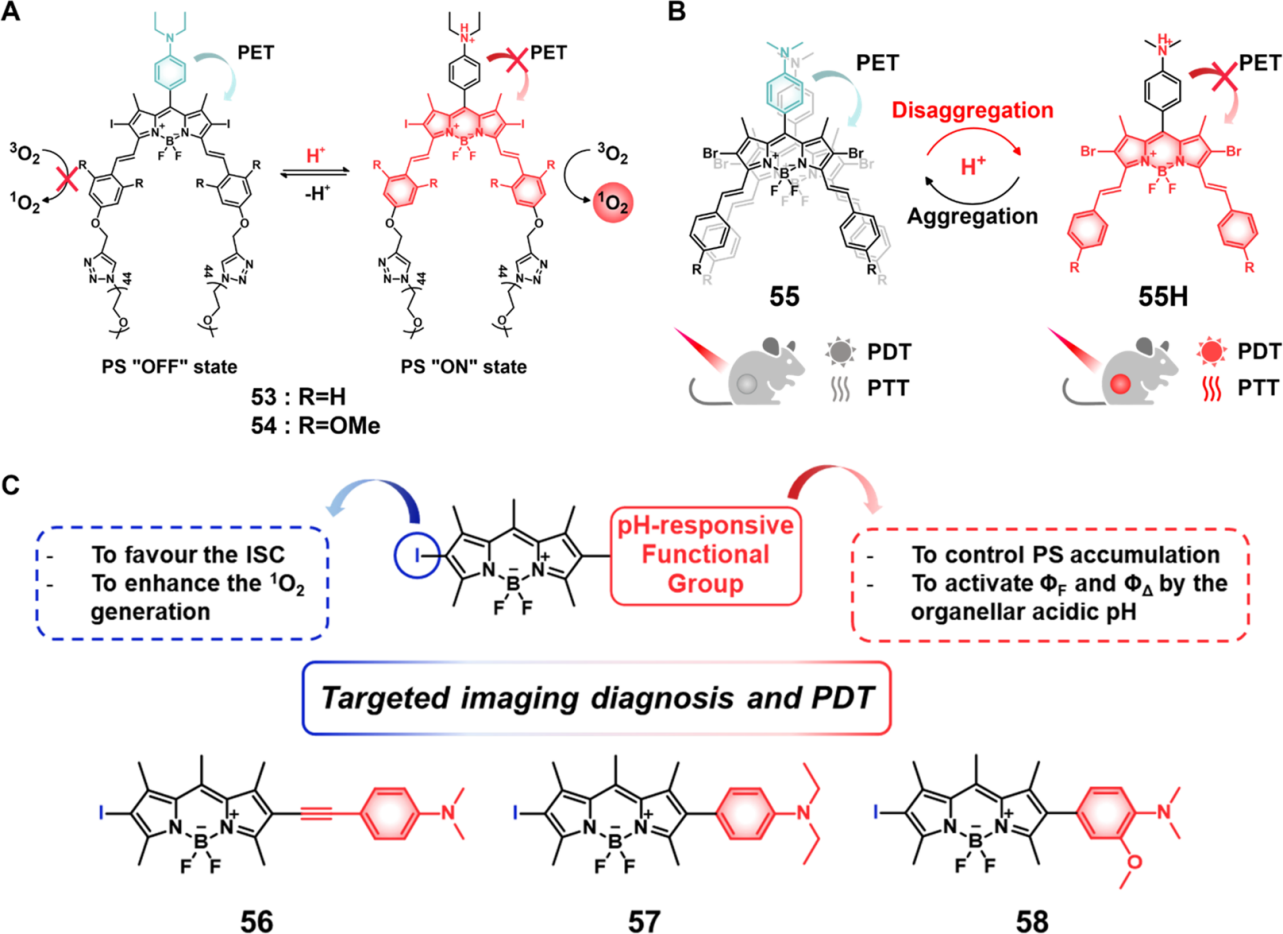
(A) Structures of pH-activatable BODIPY-based **53** and **54**.^286^ (B) Mechanism diagram of pH-mediated disaggregation of **55** for activated PDT and PTT.^287^ (C) Structures of **56**–**58** featuring pH-reversible ISC and PDT action.^288^

For instance, Siegwart et al. designed water-soluble and pH-activatable near-infrared (NIR) absorbing iodinated BODIPY-based PSs for image-guided PDT against cancer (Figure 23).^286^ Spectral analysis revealed that compounds **53** and **54** displayed strong NIR absorption and stable NIR emission with absorption maxima at 660 and 690 nm, and emission peaks at 692 and 742 nm, respectively, in acidic media. This makes them ideal for noninvasive imaging of deep tumor tissues. Importantly, the ^1^O_2_ quantum yields and fluorescence intensities of **53** and **54b** were significantly increased by the acidic pH in the TME. This is due to the protonation of the diethylaminophenyl moieties, which prevents PET quenching effects. Moreover, these BODIPY derivatives selectively accumulated in tumors after intravenous injection, without the need for an additional cancer-targeting agent. This highlights their excellent inherent tumor-targeting properties and their ability to induce significant tumor photoablation under NIR light illumination at low pH.

Based on a similar BODIPY dye parent core, Tang et al. also designed a BODIPY sensitizer with pH-dependent aggregation behavior (**55**), using bromine as a heavy atom to achieve pH-dependent photodynamic/photothermal effects combined with tumor therapy (Figure 23). This sensitizer exhibited low background toxicity and showed weak fluorescence and ^1^O_2_ production capacity at pH 7.4 due to a dual quenching mechanism (a PET effect and the aggregation state).^287^ In acidic solutions (pH 4.0), the dimethylaminophenyl moiety of **55** became protonated, leading to effective disaggregation of the protonated **55** due to enhanced solubility and charge repulsion. Consequently, both PDT and PTT activity were successfully triggered.

The lysosome, an organelle with a pH of 4.5–5.5, plays a crucial role in the cellular degradation of circulating biomacromolecules and the maintenance of homeostasis. Lysosomal damage can activate various cell death modes, and the lysosome-mediated cell death pathway (LCD) bypasses the classical caspase-dependent apoptotic pathway. Therefore, targeting lysosomes has become an important objective for antitumor therapy. In line with this, Ulrich et al. reported a series of aniline- and iodine-substituted BODIPY derivatives (**56**–**58**) as promising lysosome-targeting and pH-activated theranostic PDT agents, exhibiting significant in vitro light-induced cytotoxicity (Figure 23).^288^

**Figure 24:**
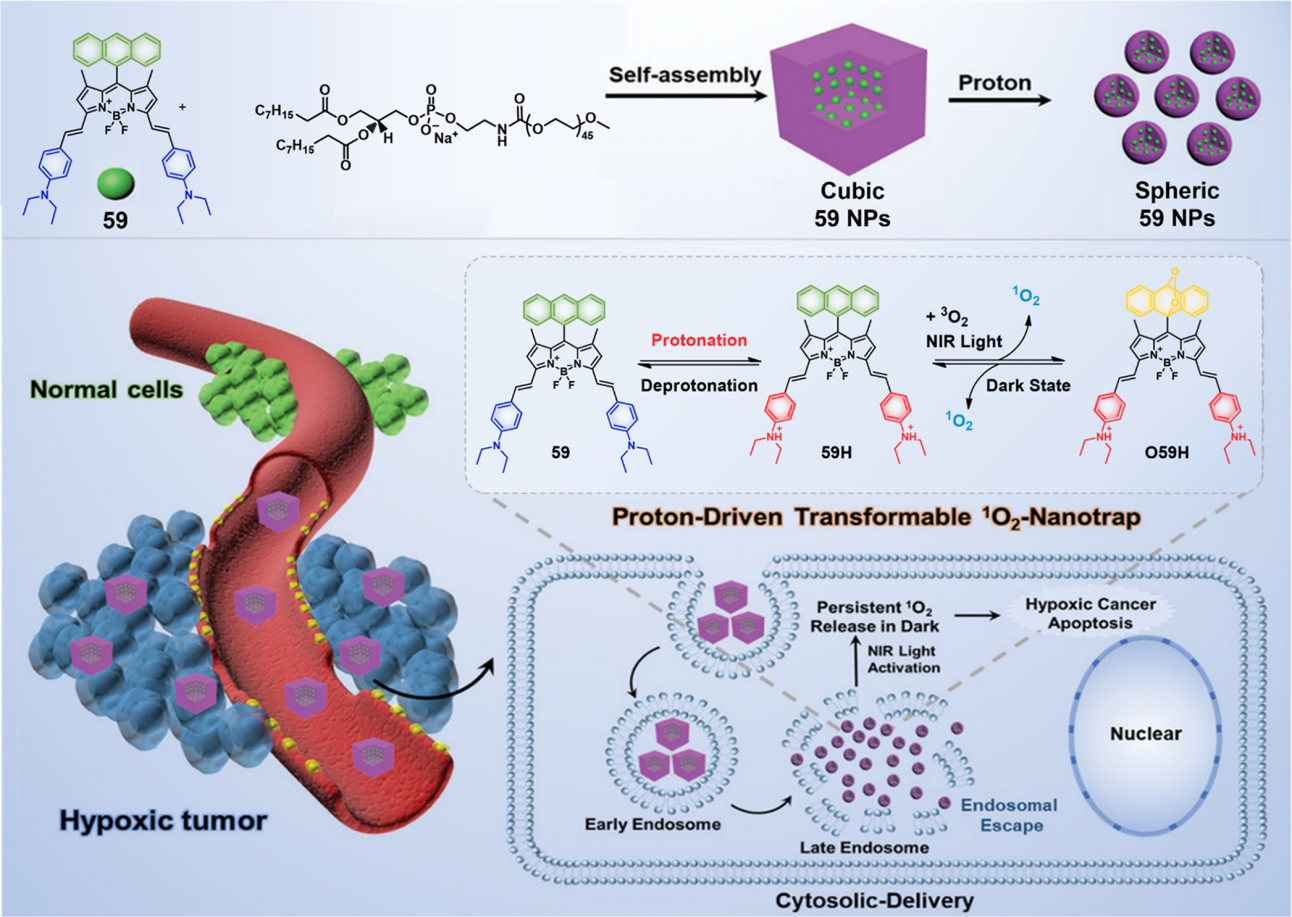
Preparation of proton-driven transformable ^1^O_2_-nanotrap and its mechanism of hypoxic cancer PDT. Reproduced with permission from ref (289). Copyright 2022 Wiley Intersciences.

To enhance the effectiveness of PDT against hypoxic tumors, Mou and co-workers developed a proton-driven transformable ^1^O_2_-trap nanoparticle system. This system includes pH-reactive dimethylaniline and polymer-encapsulated anthracenyl BODIPY (**59**) as a smart PDT agent. It is capable of releasing ^1^O_2_ in the dark and under hypoxic conditions, as depicted in Figure 24.^289^ In the acidic endosomal microenvironment, the nanoparticle system achieves efficient endosomal escape through a “proton-sponge” effect. Additionally, it undergoes a transformation from a cubic ^1^O_2_ nanotrap (94.1 nm in length) to nanospheres (12.3 nm in diameter). Meanwhile, the protonated form of **59**, with diethylamino phenyl groups receiving two protons to form ANBDPH, exhibits stronger fluorescence emission, longer fluorescence lifetime, and higher ^1^O_2_ generation capacity compared to the unprotonated form. **59** is conjugated with an anthracenyl group, known to produce endoperoxides, which can prolong the lifespan of ^1^O_2_ in dark and hypoxic conditions. In comparison to BODIPY derivatives without anthracene, **59** NPs achieved a 96.7% suppression rate of tumor growth, surpassing NBDP NPs and BDP NPs. This research provides new insights into PDT for hypoxic cancer.

**Figure 25:**
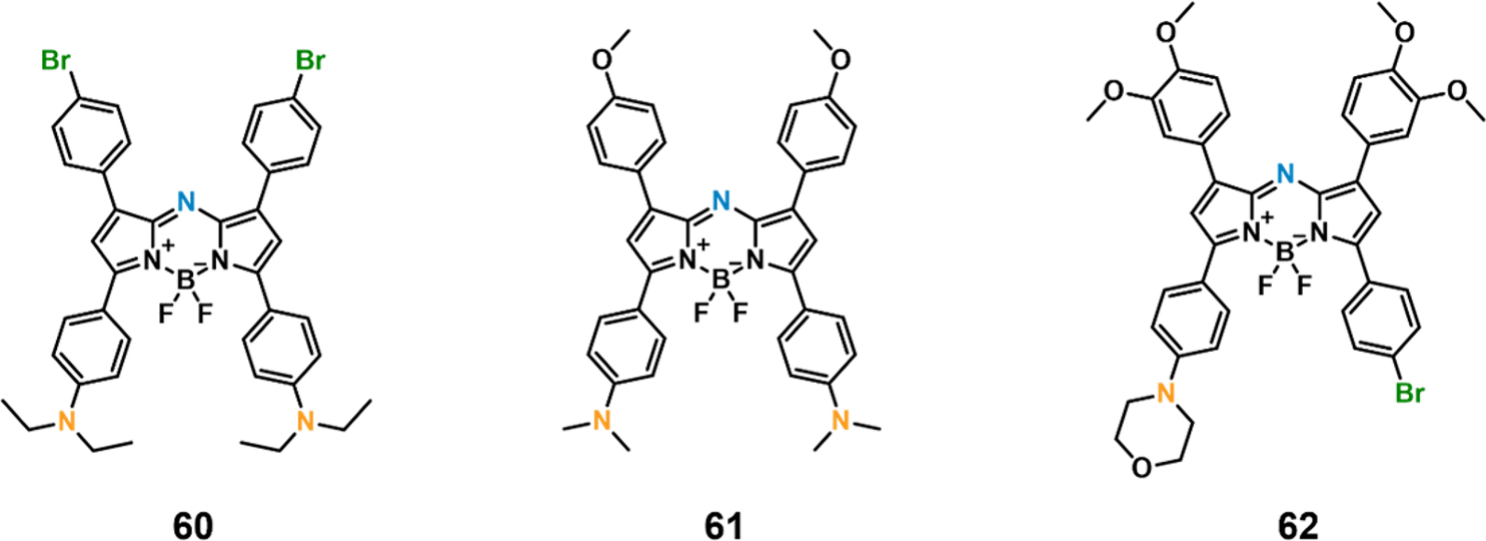
Chemical structures of theranostic probes (**60**–**62**).

Due to their easy synthesis and modifications, robust photostability, and high molar extinction coefficients, azo-BODIPY-based structures serve as an excellent platform for advanced PSs. Taking advantage of these characteristics, a family of pH-activated aza-BODIPY-based PSs has been extensively studied. These PSs incorporate anilines or morpholines as proton receptors to BODIPY, resulting in intrinsic BODIPY emission in acidic media. For example, Ju et al. developed a pH-activated nanoprobe consisting of bromophenyl aza-BODIPY (**60**). This nanoprobe was further encapsulated in a cRGD-functionalized nanomicelle to target integrin-overexpressing tumor cells for near-infrared (NIR) PDT treatment (Figure 25).^290^ In addition, Dong et al. reported two pH-triggered aza-BODIPY nanoparticles decorated with dimethylaminophenyl units (NAB, **61**) or morpholine moieties (MAB, **62**), respectively, for photoacoustic (PA) and photothermal imaging-guided simultaneous PTT/PDT (Figure 25).^291,292^

**Figure 26:**
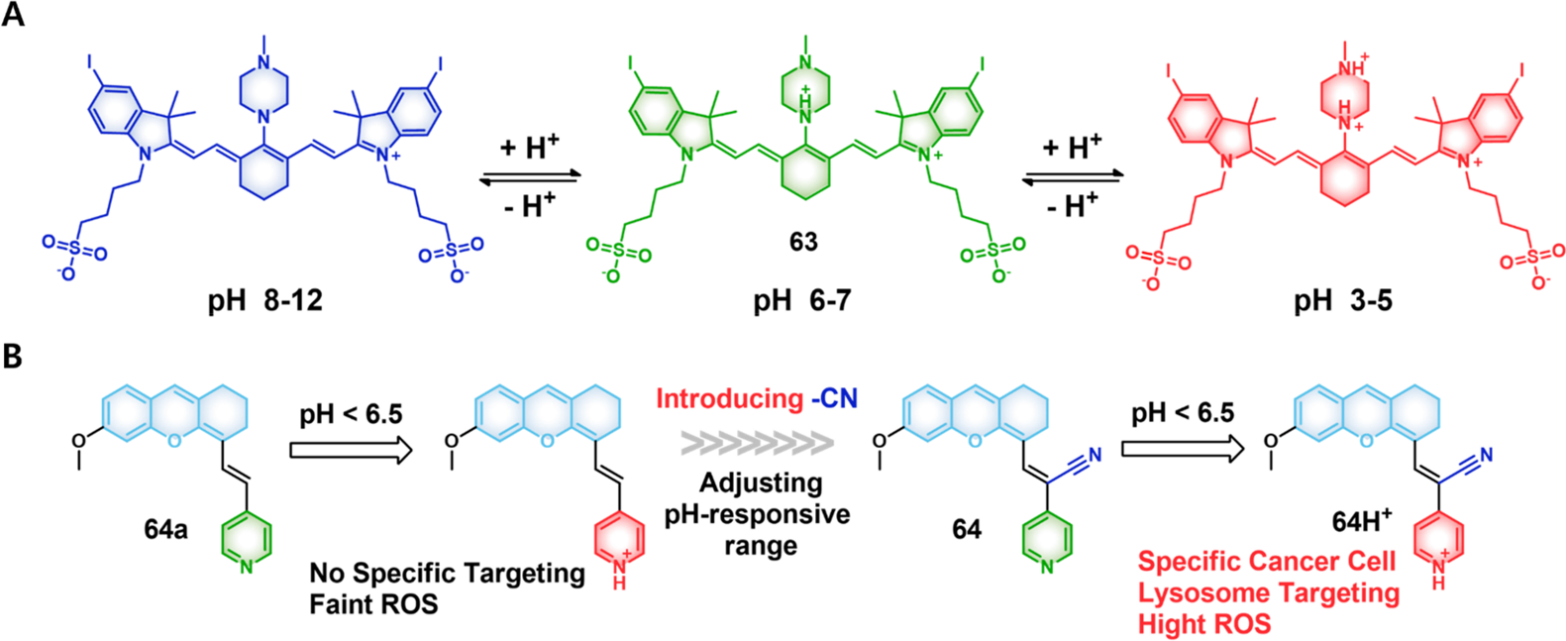
(A) Chemical structure and the pH-activated mechanism of **63**.^293^ (B) Schematic diagram of the design concept of low pH-responsive PS **64**.^294^

Cyanine dyes are a type of dye that possess excellent optical properties and have been widely used in biosensing, bioimaging, and phototherapy. Specifically, cyanine dye-derived PSs with pH-responsive groups have been developed for the selective recognition and eradication of cancer. In a study by Kamkaew et al., a pH-responsive heptamethine cyanine-based theranostic PDT agent (**63**) was developed for the treatment of HepG2 cells (Figure 26).^293^ Probe **63** exhibited a high Φ_Δ_ value and enabled NIR imaging-guided PDT under acidic conditions when the pH-sensitive *N*-methylpiperazine unit was protonated. The absorption spectra of **63** showed a red shift with decreasing pH values, which was attributed to the inhibition of an intramolecular charge transfer (ICT) process at low pH. Furthermore, theranostic **63** could selectively enter cancer cells and displayed high photocytotoxicity against HepG2 cells under acidic conditions when exposed to 850 nm LED light for 30 min.

Recently, Wang and Zhou et al. used a pyridine unit as a pH-responsive component to construct an efficient pre-PS (**64**).^294^Figure 26 shows that cyano units, which have electron-absorbing properties, made **64** exhibit a stronger pH response at lower acidities (pH < 5.0) than the control molecule ZWZ (**64a**). Moreover, probe **64** demonstrated better cellular uptake and ^1^O_2_ generation abilities. It could be activated by intracellular H^+^ to enhance intramolecular charge transfer (ICT), leading to efficient PDT in the lysosomal environment of cancer cells (such as HepG2, HeLa, and 4T1 cell lines).

**Figure 27:**
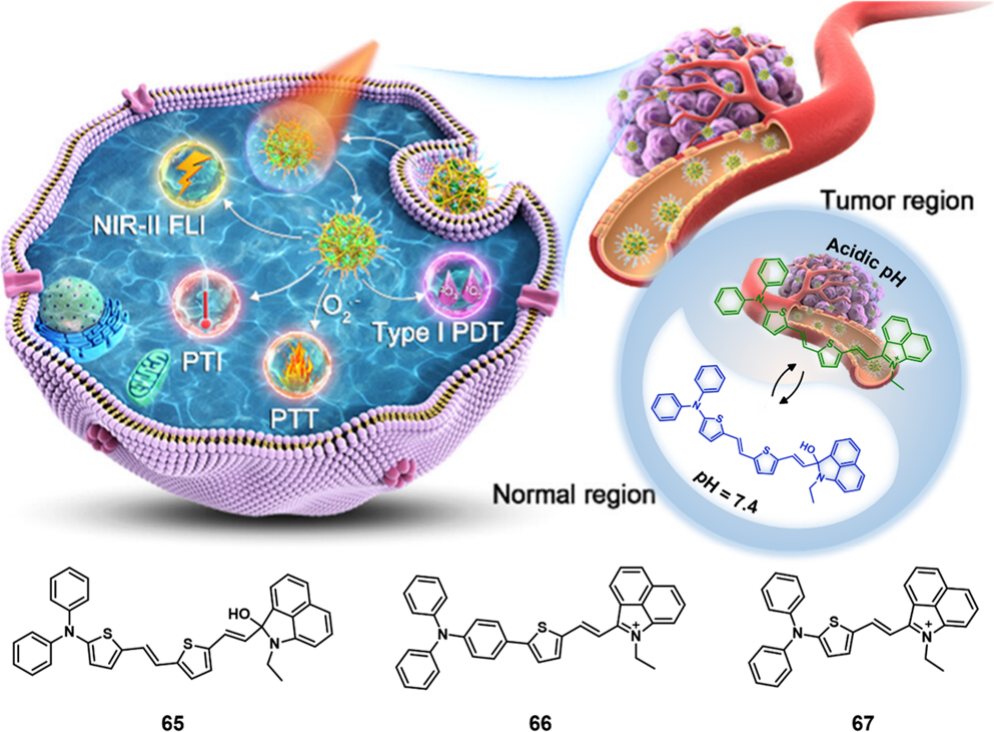
Schematic illustration of **65** NPs as a tumor reversibly pH-responsive theranostic platform. Reproduced with permission from ref (295). Copyright 2023 American Chemical Society.

Tang and Wang et al. developed a pH-switchable phototheranostic (**65**) with AIE features for NIR-II FLI-guided type I PDT/PTT in a colorectal cancer model (Figure 27).^295^**65** had higher absorptivity at 808 nm compared to the two control molecules (**66**, **67**). The maximum emission peak of **65** in DMSO was located at 1114 nm within the NIR-II window, enabling FLI-guided phototherapy. Due to strong intramolecular charge transfer, highly efficient intersystem crossing, and sufficient intramolecular motion, compound **65** generated boosted superoxide anions through a type I process and exhibited excellent photothermal performance under 808 nm laser irradiation. Notably, **65** nanoparticles, with a C=N double bond as the pH-responsive site, displayed higher ^1^O_2_ production capacity and heat generation capabilities at pH 6.5 than at pH 7.4. These biocompatible **65** nanoparticles showed significantly enhanced type I PDT/PTT in tumors, leading to significant antitumor effects in vitro and in patient-derived colon cancer xenograft models.

**Figure 28:**
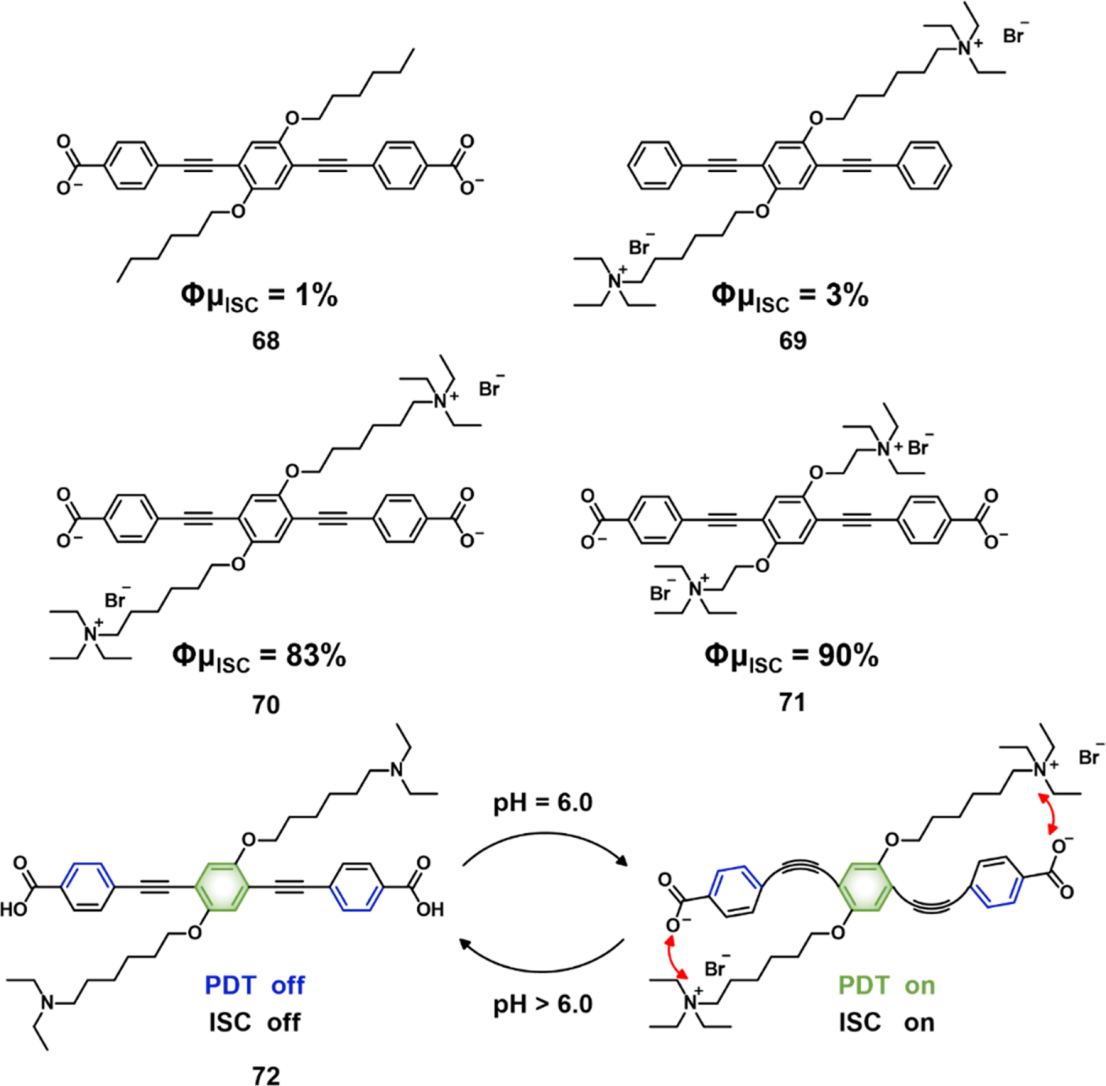
Chemical structures of **68**–**71** and schematic diagram of the reversible ISC and PDT effects of **72** at different pH values.^296^

Huang et al. utilized *p*-phenylethynylene to design a series of purely organic materials (POMs) **68**–**72** displaying a pH-responsive reversible switching of ISC for smart PDT (Figure 28).^296^ The organic structures displayed efficient ISC properties, which were estimated by femtosecond transient absorption (fs-TA) spectroscopy and quantum chemical calculations. The results showed that as the degree of torsion increased, the ISC efficiency increased from 1% to 90%. The ^1^O_2_ quantum yield of **72** was calculated to be about 0.48 at pH 6.0 and 0.05 at pH 7.4, respectively, indicating that ISC is enhanced in acidic environments. In vitro, **72** showed outstanding selectivity in tumor cells compared to the commercial PS TMPyP4. This work provides a reversible switching method for adjusting ISC by pH, thereby increasing the accuracy of PDT.

#### 2.2.2 Biothiol-Activatable Theranostic Probes in PDT

Biothiols, such as GSH, cysteine (Cys), and homocysteine (Hcy), play crucial roles in biological systems, particularly in maintaining the redox balance of living cells.^297^ Cancer cells, which are under constant oxidative stress, often increase their levels of biothiols as a compensatory mechanism.^298^ For instance, the concentration of GSH in cancer cells can be 1–10 mM, significantly higher than that in normal cells (more than 5 times higher).^299−301^ Therefore, abnormal biothiol concentrations can serve as indicators to differentiate cancer cells from normal cells and can also be used to develop biothiol-activatable smart PSs to enhance the effectiveness of PDT against tumors while minimizing damage to healthy tissues.^302−305^

The sulfhydryl (−SH) group present in cysteine enables GSH or Cys to exhibit strong reductive and nucleophilic properties, allowing them to participate in various chemical and biological reactions. Depending on the reactivity of the −SH group, biothiol-mediated activation strategies or mechanisms can be further categorized into sulfonate ester cleavage, disulfide exchange, nucleophilic substitution reactions, cyclization with aldehydes or cyano groups, GSH-mediated reduction, and Michael additions.

##### 2.2.2.1 Cleavage of Sulfonate Esters

**Figure 29:**
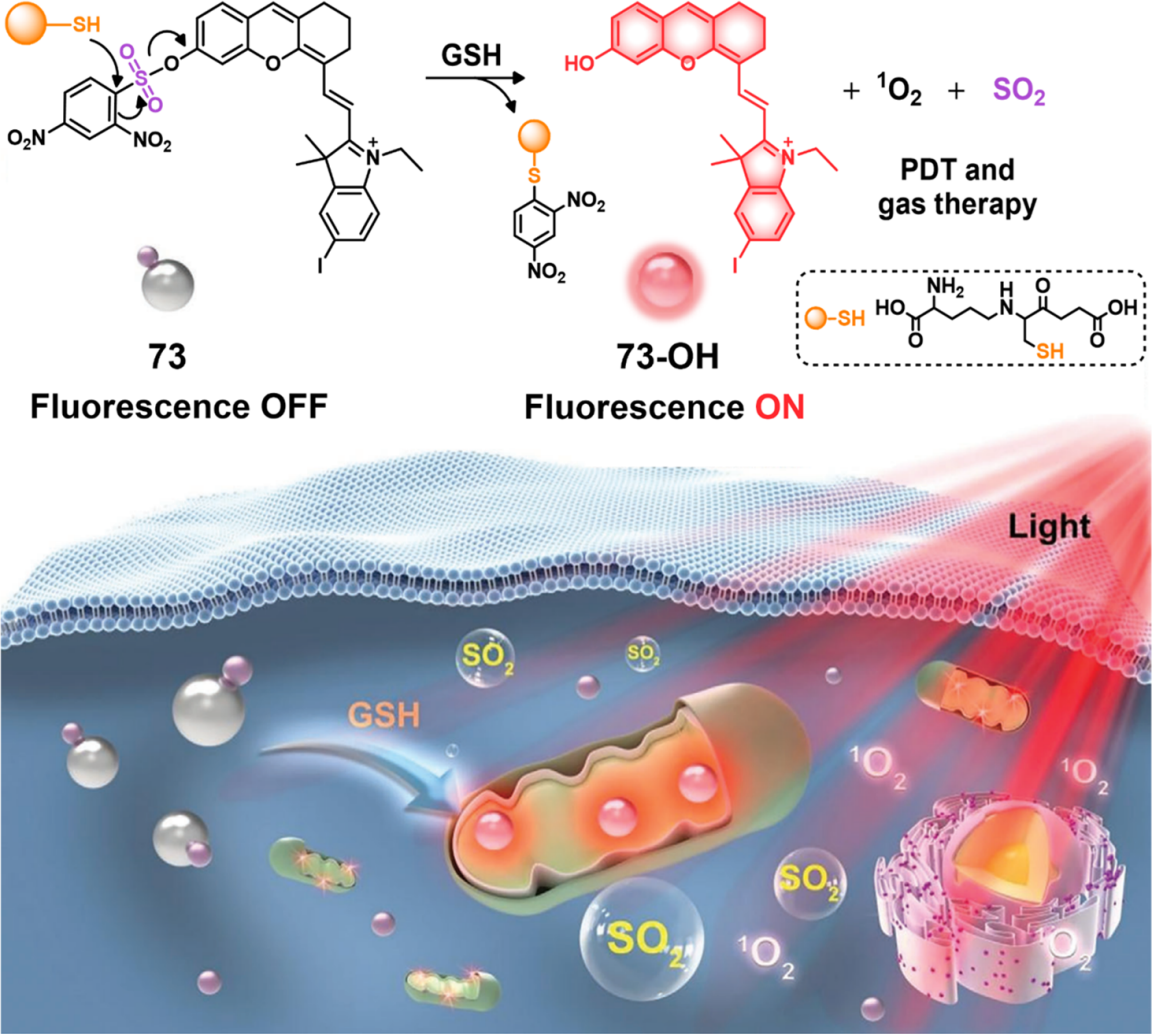
Schematic illustration of **73** for combined PDT and SO_2_ gas therapy. Reproduced with permission from ref (306). Copyright 2021 Wiley Intersciences.

Previous investigations have confirmed that 2,4-dinitrobenzenesulfonyl is a responsive group to thiols. This group possesses a strong electron-withdrawing capability, which has been utilized in the development of GSH-activated PSs. In a recent study by Sun et al., a GSH-triggered near-infrared (NIR) PS (**73**) was designed by incorporating iodine-substituted hemicyanine (**73**-OH) with 2,4-dinitrobenzenesulfonate (DNBS) (Figure 29). This PS was intended for use in combined PDT and sulfur dioxide (SO_2_) therapy.^306^ SO_2_, a member of the gasotransmitter family, has traditionally been recognized as an air pollutant. However, recent reports have unveiled its potential in disease treatment, particularly in synergistic cancer therapy to overcome drug resistance. In this study, SO_2_ was generated by the removal of the DNBS cage group through nucleophilic substitution by intracellular GSH. In addition, the active PS **73**-OH was released, enabling the production of cytotoxic ^1^O_2_ upon red light irradiation. This PS exhibited a significant decrease in cancer cell viability and inhibited tumor growth, demonstrating its excellent anticancer effect. The structure of **73** consisted of two parts with distinct functions: DNBS served as a GSH recognition site, an effective quencher of the excited state, and an SO_2_ generator, while hemicyanine, containing a heavy iodine atom, acted as the PS core for light-induced production of cytotoxic ROS. In normal tissues, **73** remained in an “off” state due to the suppression of an intramolecular charge transfer (ICT) process. The excited state energy was mostly released through nonradiative decay. However, upon uptake by tumor cells, the intracellular GSH selectively removed the cage group (DNBS) via nucleophilic substitution, resulting in the generation of SO_2_. Simultaneously, the active PS **73**-OH was released, leading to the production of cytotoxic ^1^O_2_ and concomitant red emission for fluorescence imaging-guided PDT. This work introduced a novel approach for designing PSs that combine PDT and SO_2_ therapy, demonstrating their potential in synergistic anticancer treatments.

**Figure 30:**
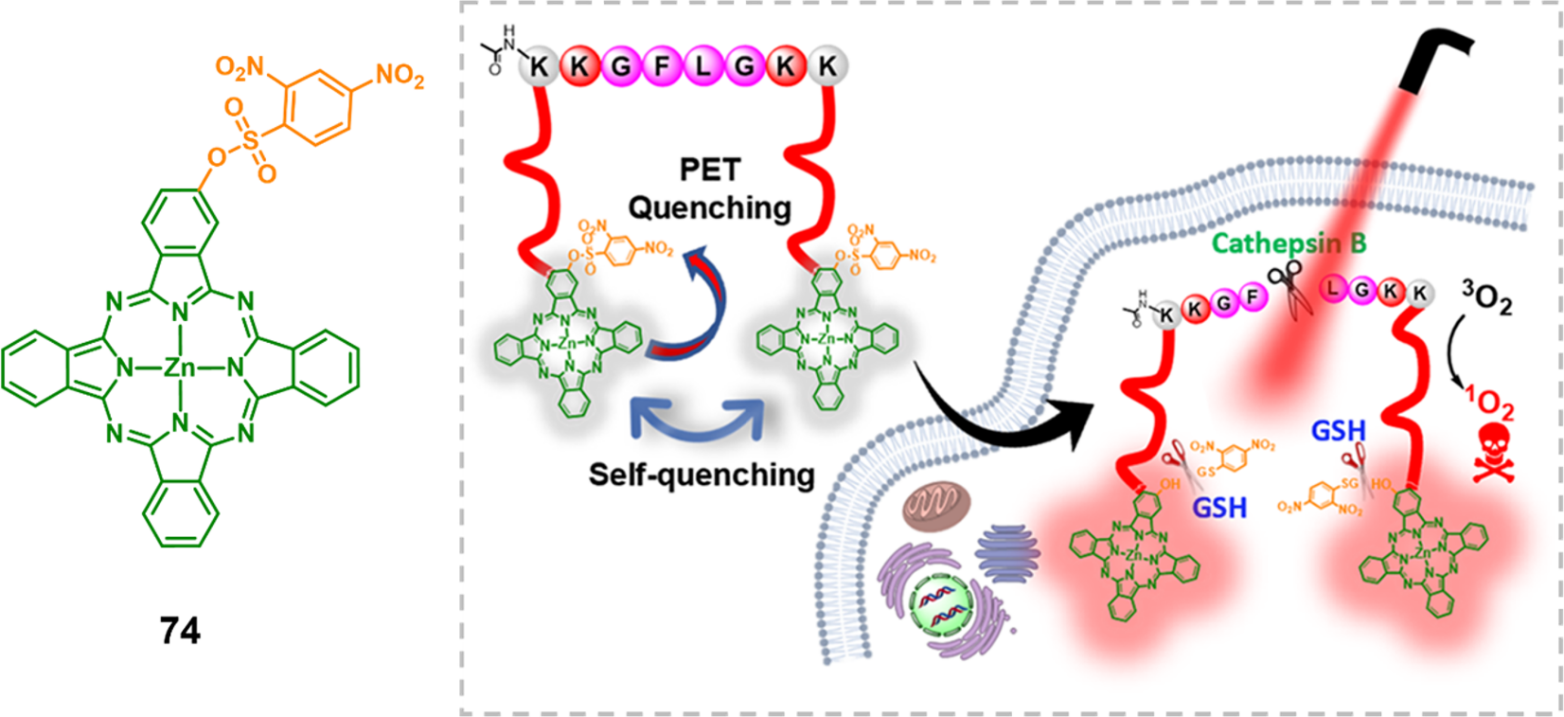
Schematic illustration of **74** for dual stimuli-activated (cathepsin B and GSH) PDT. Reproduced with permission from ref (307). Copyright 2021 American Chemical Society.

To minimize nonspecific activation and achieve more precise PDT, Lo et al. designed and synthesized two dual stimuli-activated (cathepsin B and GSH) PSs (**74**),^307^ where two or three DNBS-caged ZnPc units were covalently connected with one or two cathepsin B-cleavable peptide linkers (Gly-Phe-LeuGly) through the click reaction (Figure 30). Due to the strong PET effect induced by DNBS moieties and significant self-quenching between ZnPc units, compound **74** (dimeric ZnPc-based PMB 1 and trimeric ZnPc-based PMB 2) was fully quenched in terms of fluorescence emission and ^1^O_2_ generation (Φ_F_ = 0.001–0.005, Φ_Δ_ = 0.03–0.07) in contrast to ZnPc (Φ_F_ = 0.28, Φ_Δ_ = 0.56). In the presence of both stimuli conditions (GSH and cathepsin B), or upon internalization into A549 and HepG2 cancer cells, compound **74** was activated to release free phthalocyanine via the cleavage of the peptide linkage and subsequent removal of the DNBS moieties, realizing 80–87% fluorescence recovery and efficient ROS generation, as a result of disaggregation of the photosensitizing units and the disappearance of the PET effect. When A549 and HepG2 were pretreated with **74**, followed by light irradiation (λ > 610 nm, 23 mW cm^–2^, 28 J cm^–2^), a substantial decrease in viability of both cell lines with low IC_50_ values of 0.21–0.39 μM was observed. Dual cathepsin B and GSH-responsive PSs offer a promising approach for increasing the specificity of the photodynamic action.

##### 2.2.2.2 Disulfide Exchange

**Figure 31:**
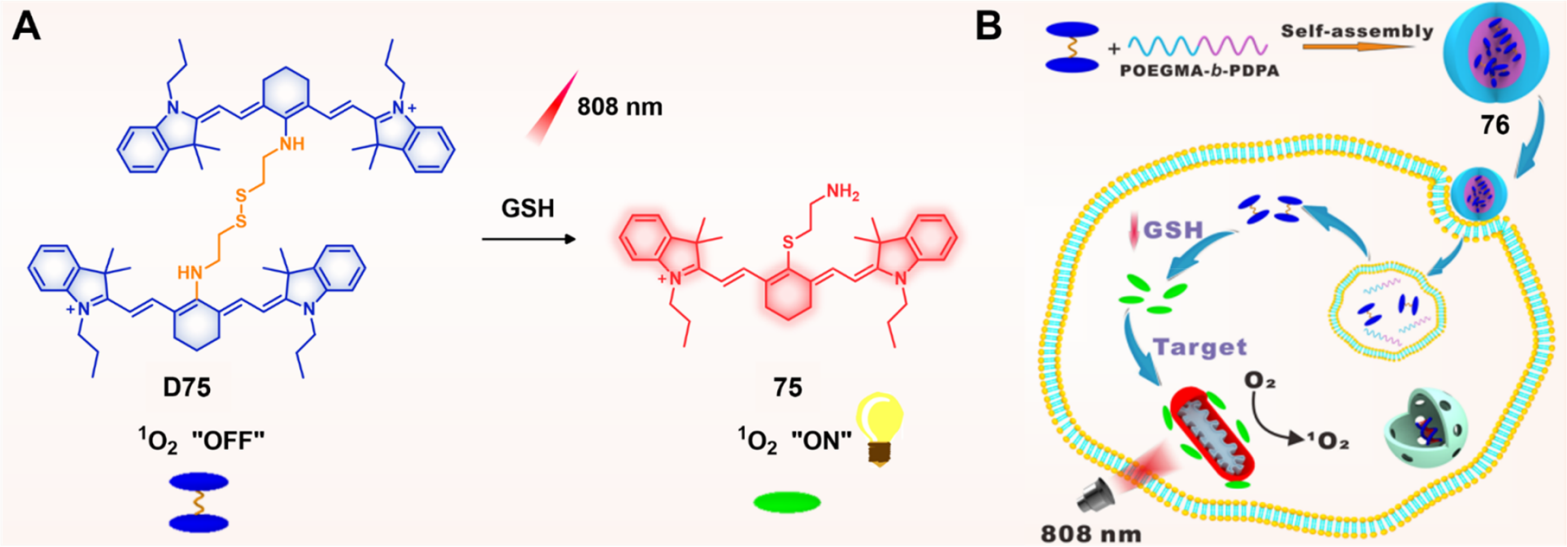
Schematic illustration of GSH-activatable photoactivity of **D75**. Reproduced with permission from ref (311). Copyright 2019 American Chemical Society.

The disulfide bond is a thiol-responsive group that has been widely used as a linker in the fabrication of “smart” therapeutic prodrugs or reduction-sensitive drug delivery systems.^308−310^ Mitochondria targeting is recognized as an effective strategy to enhance cancer phototherapy, as intracellular mitochondria are more susceptible to cytotoxic ^1^O_2_ than other organelles. Taking this into consideration, Zhang et al. developed a GSH-activatable and mitochondria-targeted pro-photosensitizer (**75**) by covalently linking two *meso*-amine-substituted cyanine moieties with a disulfide bond (Figure 31). This pro-photosensitizer was then encapsulated within an ultrasensitive pH-responsive polymer to form **D75**-loaded nanoparticles (**76**).^311^ After being endocytosed into cancer cells, **76** dissociated in endosomes, releasing a large quantity of encapsulated **D75**. The released **D75** successfully escaped from endosomes due to the “proton sponge” effect caused by the presence of diisopropyl-substituted tertiary amines. In the cytoplasm, the free **D75** was cleaved by abundant intracellular GSH, forming a thiolate-substituted new cyanine. This new compound specifically concentrated in the mitochondria of tumor cells and induced the generation of ^1^O_2_ under 808 nm laser irradiation. MTT results showed that **76** exhibited selective phototoxicity against tumor cells compared to normal cells and significantly enhanced PDT efficacy by depleting intracellular GSH. In conclusion, the GSH-activatable and mitochondria-targeted nano PS, based on covalently linked cyanine dyes, offers a new approach for precise tumor phototheranostics.

**Figure 32:**
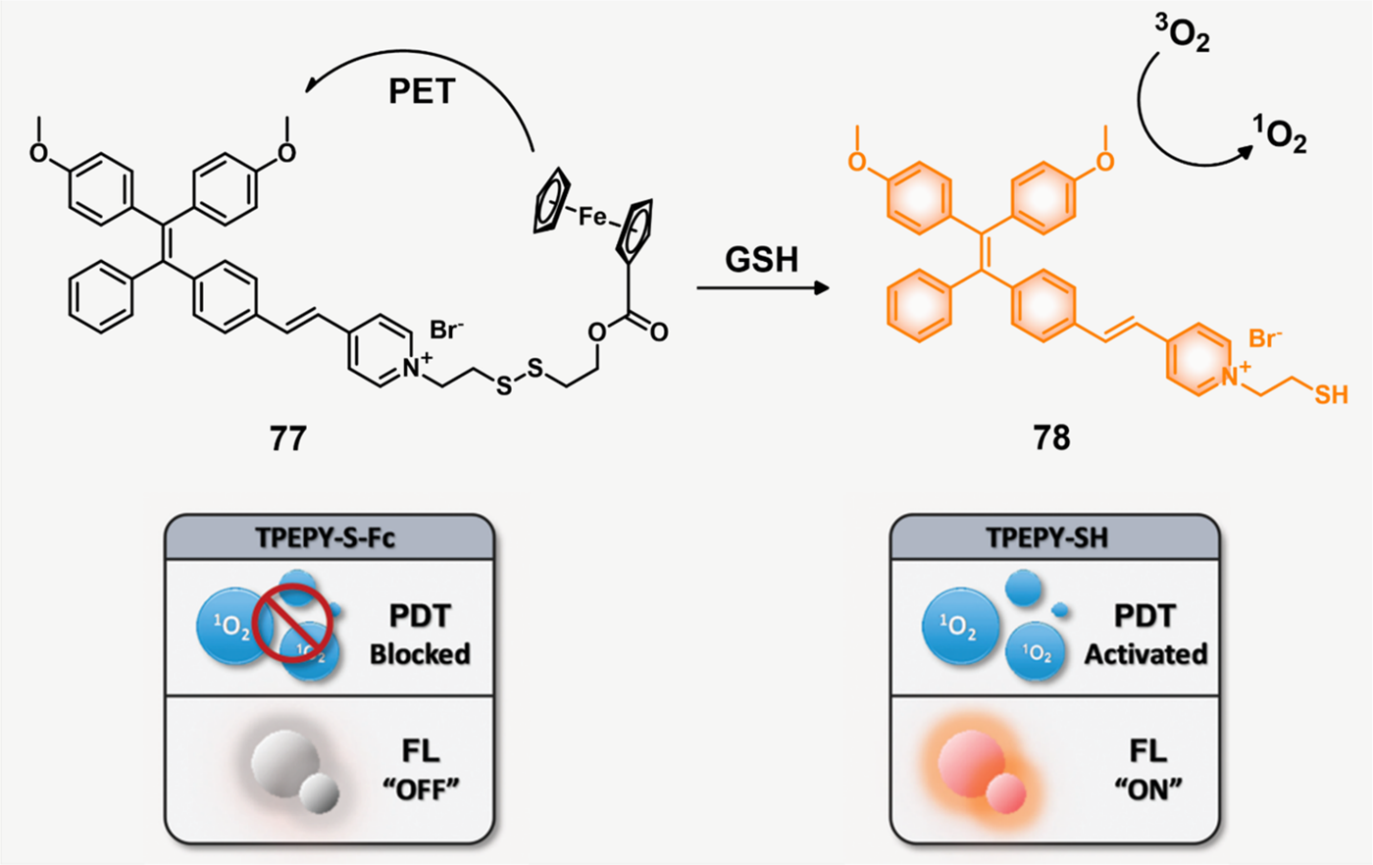
Structure of **77** and the proposed mechanism of GSH activation of PDT. Reproduced with permission from ref (315). Copyright 2020 Royal Society of Chemistry.

Compared with conventional PSs that often have undesired aggregation-caused quenching (ACQ) effects, the aggregation-induced emission fluorogens (AIEgens) developed by Tang’s group exhibit desirable properties such as a large Stokes shift, high brightness, and good photostability. This opens a new approach for designing bright PSs.^312−314^ Recognizing these advantages, Kim et al. reported a GSH-activatable PS based on AIE technology. In this design, a ferrocene unit was attached to vinylpyridinium-substituted tetraphenylethylene (**77**) through a disulfide bond.^315^ As shown in Figure 32, compound **77** undergoes a photoinduced electron transfer (PET) process between the dye and the ferrocene subunits, resulting in quenching of the excited states and blocking of fluorescence and ^1^O_2_ generation (Φ_Δ_ = 2.4%). When the disulfide bond is cleaved by GSH, the PDT-active compound **78** is released, leading to enhanced fluorescence and activated ^1^O_2_ generation (Φ_Δ_ = 21.3%). Additionally, **78** exhibits cancer cell-specific imaging and induces apoptosis after irradiation, an effect attributed to the higher concentrations of GSH in cancer cells. This work presents a novel smart PS that combines the characteristics of GSH activation and aggregation-induced emission for imaging-guided PDT.

**Figure 33:**
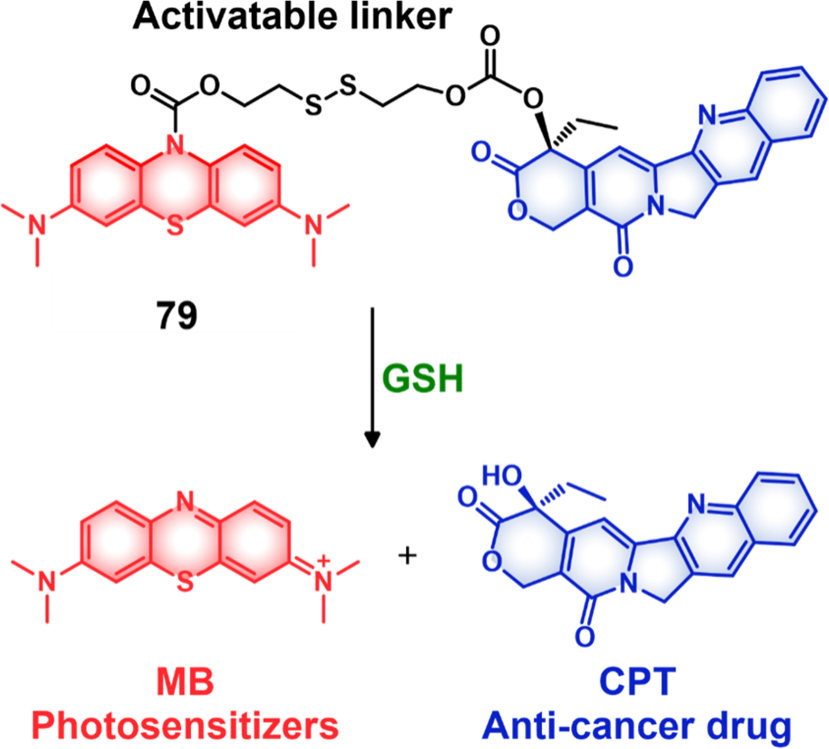
MB and CPT release mechanism of the activable **79** by GSH. Reproduced with permission from ref (321). Copyright 2022 Elsevier Ltd.

Numerous studies have shown that dual-mode therapy such as combined chemo- and phototherapy exhibits better anticancer effects than monotherapy, even producing synergistic effects.^316−320^ However, the simple combination of conventional chemotherapeutics and “always on” PSs often causes strong side effects on normal tissues due to the lack of targeting. To solve the problem, Sun et al. reported a “pro-drug-PS” agent (**79**), where both the cytotoxicity of the drug (CPT) and the photodynamic activity of MB were blocked by a GSH-activatable disulfide linker (Figure 33).^321^ Upon being taken up by cancer cells, the enhanced GSH concentration inside cells cleaved the S–S bond to release both CPT and MB, resulting in simultaneous activation of chemical toxicity and photosensitivity. Under a very low laser power density (660 nm, 1 mW cm^–2^), compound **79** could trigger severe apoptosis and markedly enhanced cytotoxicity toward cancer cells compared to monotherapy, thus revealing a synergetic chemo-photodynamic killing effect. Moreover, in a 4T1-bearing tumor mouse model, **79** exhibited excellent tumor-activatable performance with negligible toxic side effects. This work suggests an intelligent strategy for improving the specificity of targeted PDT and minimizing toxic side effects.

##### 2.2.2.3 Nucleophilic Substitution Reaction

**Figure 34:**
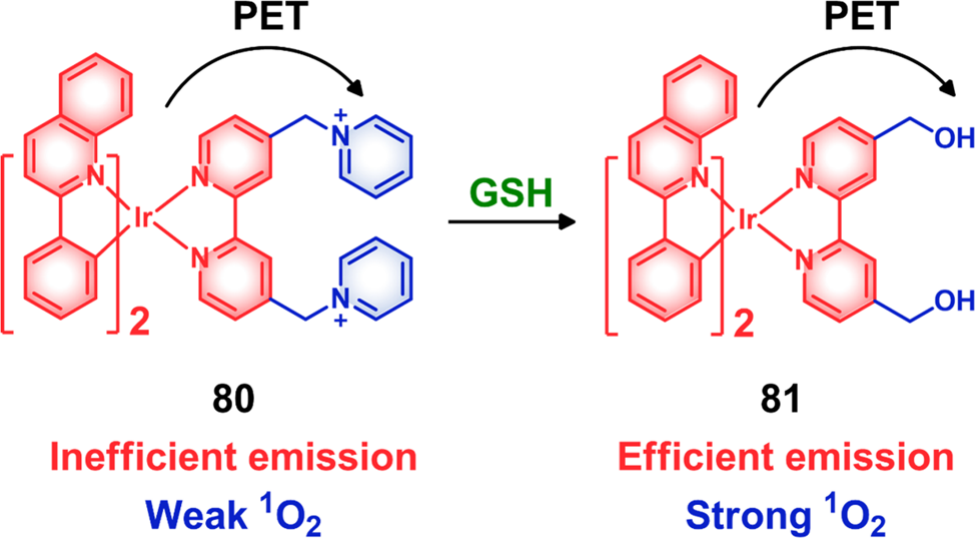
Chemical structures of **80** and **81**, and the process of GSH-activatable PDT.

Owning to the strongly reductive power of GSH, electron-deficient pyridine groups are theoretically sensitive to GSH, suggesting that the latter can be used as a recognition site to design GSH-activated PSs. As a proof-of-concept, Zhao et al. designed a class of GSH-activatable phosphorescent iridium(III) complexes bearing various types of benzylpyridiniums (Figure 34).^322^ By tuning the electron-donating abilities of the pyridinium scaffold, a PET process occurred between the iridium(III) core and the electron-deficient pyridinium, which limited the energy transfer from the excited state of the Ir complex to the ground state of oxygen to generate ^1^O_2_, and at the same time facilitated a GSH-induced nucleophilic substitution reaction. In the case of **80**, electron transfer from GSH to the positive nitrogen of pyridinium enhanced the susceptibility toward hydroxide nucleophilic attack and promoted the occurrence of a substitution reaction by hydroxide to form **81**, thus resulting in irreversible changes of spectral properties, the fluorescence lifetime, and the PDT effect of this PS. For example, after the addition of an aqueous GSH solution (20 μM), the phosphorescence of **80** was gradually blue-shifted (from 627 to 586 nm) accompanied by a 12.5-fold enhancement of emission intensity and an extended emission lifetime (from 84.7 to 690.5 ns). More importantly, the abundant GSH in tumor cells inhibited the intramolecular PET process of **80**, achieving GSH-dependent phototoxicity. Hence, this novel Ir complex-based PS can not only selectively distinguish cancer cells by luminescence or lifetime imaging but also amplify PDT effects in cancer cells, providing a new avenue for the design of a smart, responsive theranostic platform. This work demonstrated an effective design strategy for smart GSH-activatable PSs, providing guidance for the exploitation of near-infrared light PSs with a GSH-specific response in the future.

**Figure 35:**
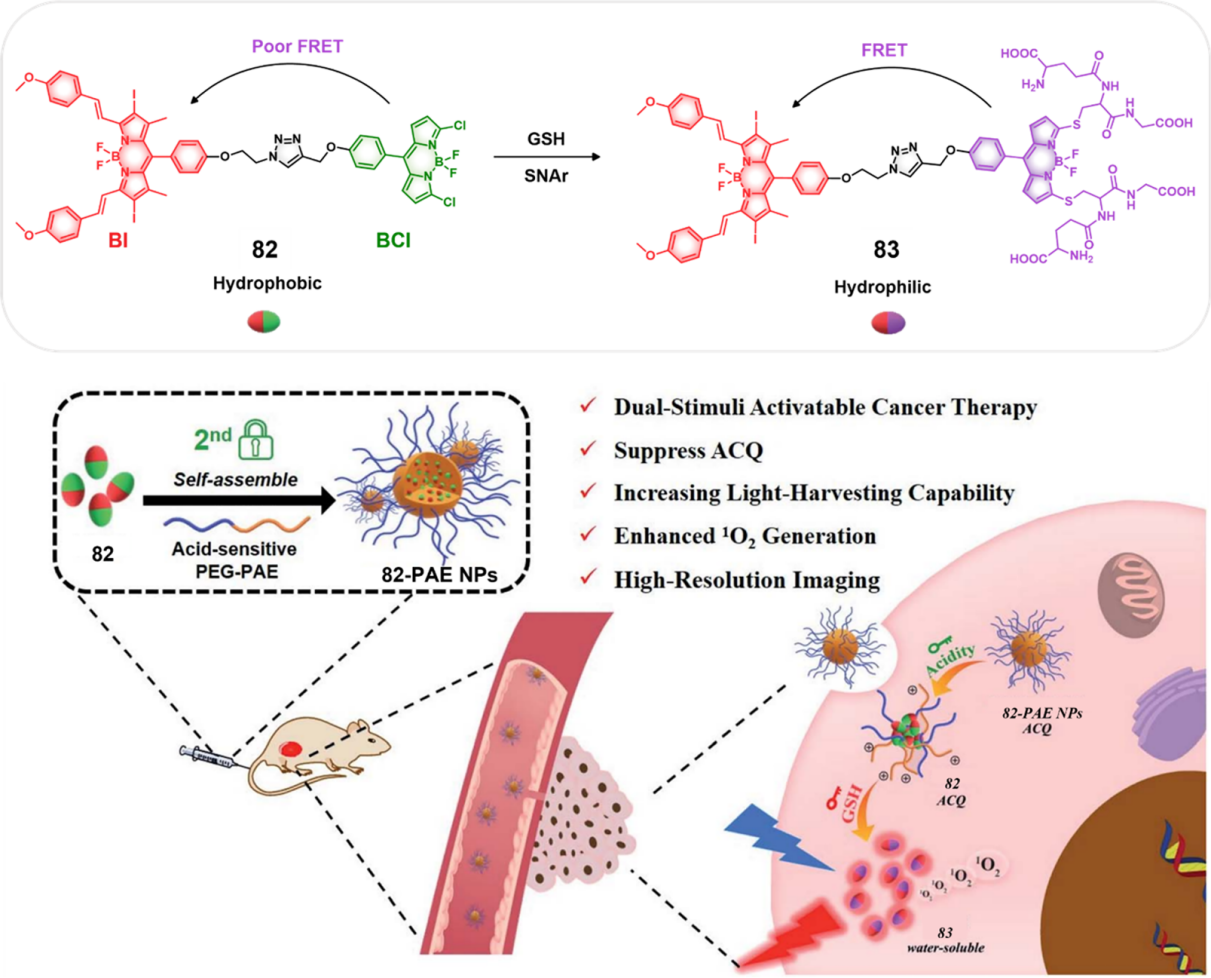
Schematic illustration for the fabrication of BIBCl-PAE NPs, and the processes underlying “dual lock-and-key” strategy in a tumor microenvironment to achieve activated and enhanced generation of ^1^O_2_. Reproduced with permission from ref (323). Copyright 2020 The Royal Society of Chemistry.

In contrast to single-stimuli-activatable PSs that often encounter nonspecific activation and may even result in false positive signals in complex environments, smart PSs activated by multiple combined stimuli can provide robust and precise therapy. For this purpose, Yang et al. developed a pH and GSH coactivatable supramolecular PS (**82-PAE** NPs) using the “dual lock-and-key” strategy by encapsulating a GSH-activatable PS **82** in a pH-responsive diblock copolymer poly(ethylene glycol)-poly(β-amino ester) (PEG-PAE) (Figure 35).^323^ In normal tissues, the aggregation of **82** in polymeric micelles results in quenched excited states. When PEG-PAE was decomposed by the low pH in the TME, compound **82** was released and quickly reacted with GSH to form a water-soluble sensitizer (**83**) through nucleophilic substitution. The resulting hydrophilic **83** avoided the ACQ issue of conventional BODIPY dyes and possessed a 70 nm redshift in the fluorescence spectrum due to GSH substitution, which allowed for a good spectral overlap with the absorption spectrum of **BI**, enabling efficient FRET. Thus, an improved light harvesting and ^1^O_2_ production ability was achieved simultaneously, and the ^1^O_2_ yield of **83** is even better than that of commercial PS Ce6. In vivo results revealed that **82-PAE** NPs can be rapidly enriched in cancer cells and can selectively “light up” tumors, further causing irreversible cytotoxicity to tumors without influence on normal tissues. This work represents the first example of a GSH-activated PS based on the mechanism of aromatic nucleophilic substitution.

##### 2.2.2.4 Cyclization with Cyano Groups

**Figure 36:**
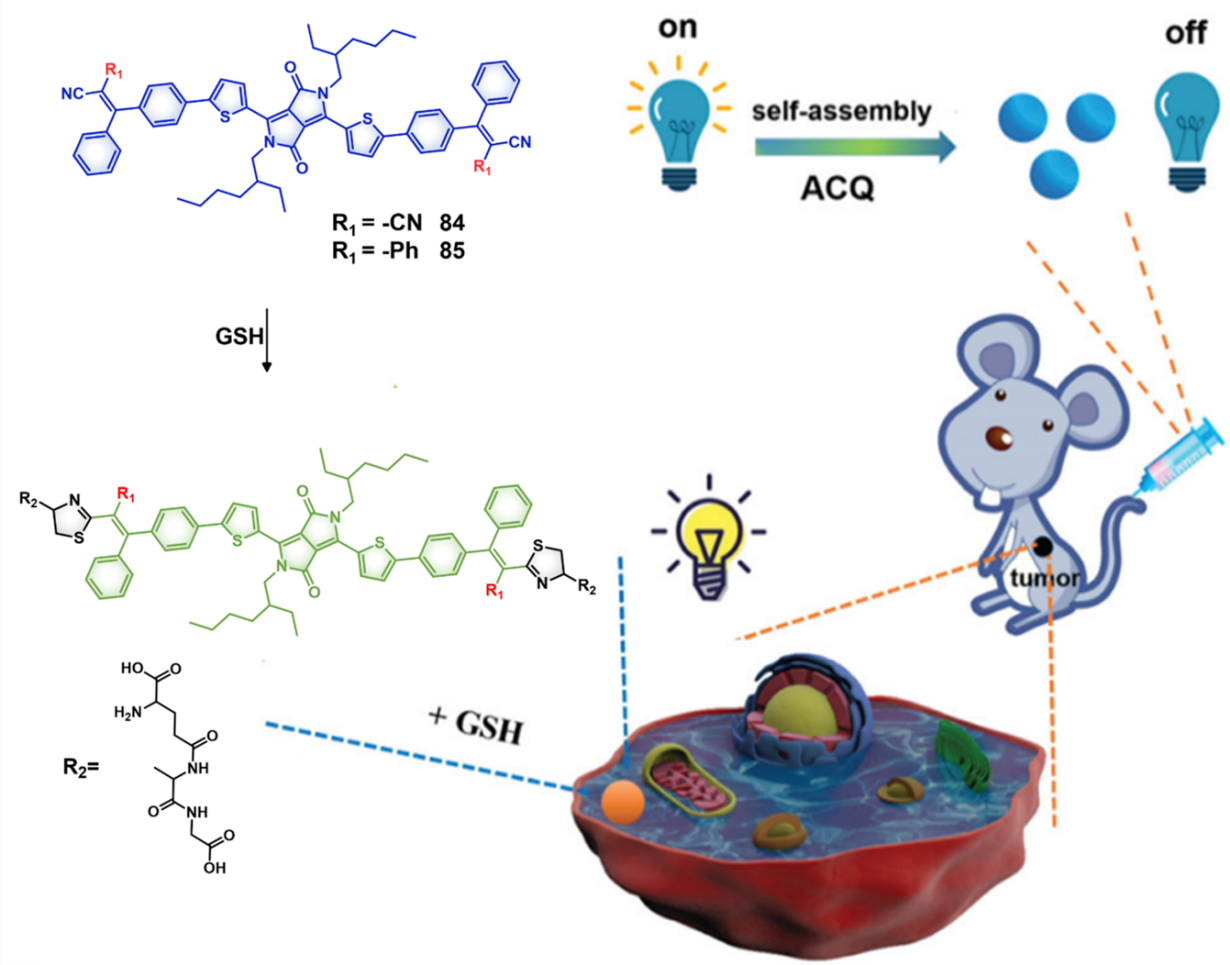
Illustration of GSH-responsive DPP derivatives (**84**, **85**) for imaging-guided phototherapy. Reproduced with permission from ref (324). Copyright 2019 The Royal Society of Chemistry.

The CN group can recognize the thiol of GSH to form a thiazole ring via a Michael addition. Based on this reaction, Dong et al. designed and synthesized two kinds of GSH-responsive pyrrolopyrrolidone (DPP) derivatives bearing with different regio-isomers of the CN group, **84** (4-CN groups) and **85** (2-CN groups), which further assembled into corresponding nanoparticles **84** NPs and **85** NPs, via the nanoprecipitation method (Figure 36).^324^ Both can react with GSH to form a thiazole through the Michael addition, realizing the colorimetric GSH detection without aggregation-induced fluorescence quenching, as well as enhanced PDT/PTT efficacy. Interestingly, **84** NPs showed a higher ^1^O_2_ quantum yield (22.3% vs 12.5%) and photothermal conversion efficiency (45.2% vs 34.5%) than **85** NPs. Thus, **84** NPs exhibited a better therapeutic efficacy than **85** NPs even at a low dose (0.2 mg kg^–1^) without adverse effects.

##### 2.2.2.5 GSH Mediated Reduction

**Figure 37:**
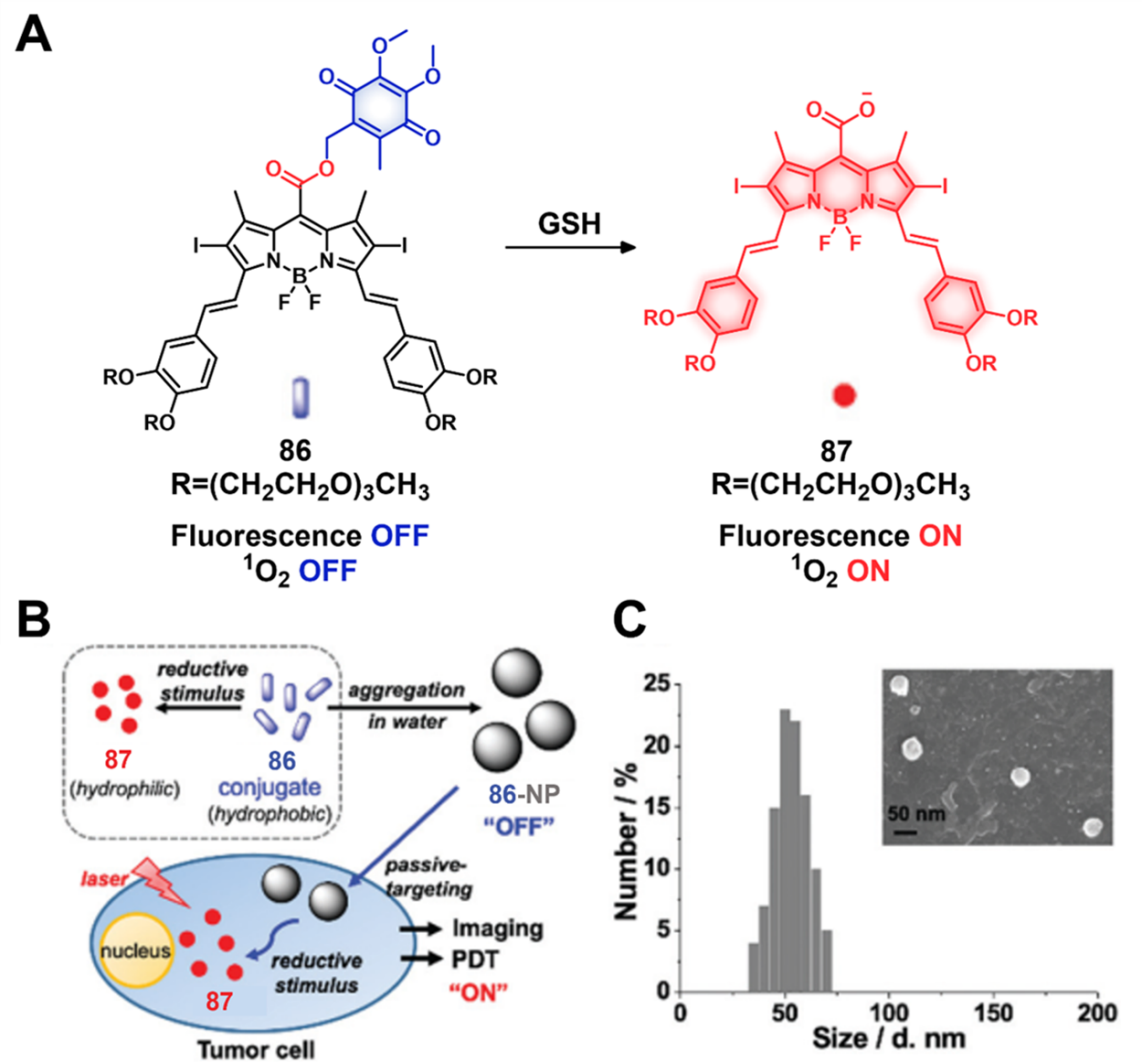
Chemical structure of **86** conjugate and conversion to **87** under reductive conditions. Reproduced with permission from ref (325). Copyright 2019 The Royal Society of Chemistry.

To achieve efficient tumor enrichment and cell uptake PSs that exhibit a switch in polarity from a hydrophobic inactive state to a hydrophilic active state are highly desirable. To this end, Kim et al. reported a GSH-activatable PDT agent (**86**), where a GSH-sensitive moiety (a ubiquinone analogue) was covalently linked to a near-infrared PS (a meso-ester-2,6-iodinated-BODIPY) via an ester linkage (Figure 37).^325^ Compound **86** is strongly hydrophobic and easily aggregates to form nanoparticles in aqueous solutions. Not only does this result in quenched photosensitivity and minimal systemic toxicity, but this also favors tumor enrichment through the EPR effect. Upon endocytosis by cancer cells, the ubiquinone moiety was rapidly reduced by GSH to produce the corresponding ubiquinol that underwent a spontaneous elimination reaction to release the anionic **87** and further induced the deaggregation of **86** NPs. The conversion of the aggregated hydrophobic precursor (**86**) into the photoactive hydrophilic **87** resulted in a “Turn-On” of the fluorescence and ^1^O_2_ generation (**87**, Φ_Δ_ = 0.79), enabling the selective photodynamic killing of cancer cells and the ablation of the tumor guided by fluorescence imaging.

##### 2.2.2.6 Michael Additions

**Figure 38:**
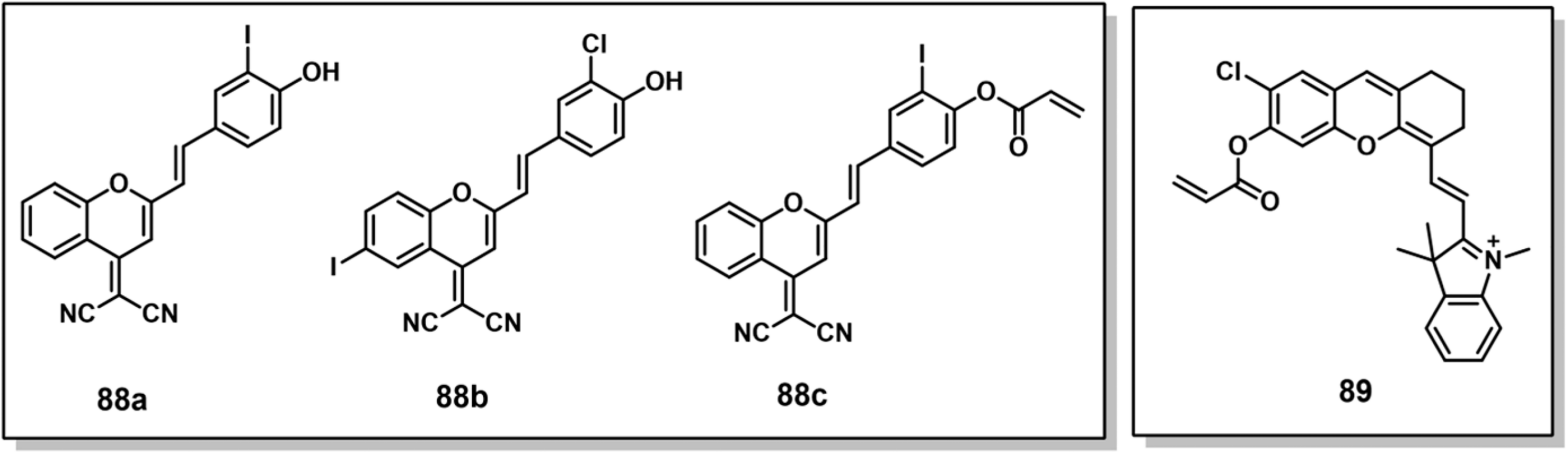
Structures of theranostic probes **88a**, **88b**, **88c**, and **89**.

Like other biothiols, Cys plays critical roles in physiological processes, including redox homeostasis and as an antioxidant. Elevated levels of Cys are directly associated with different pathogenic states, such as cancers and neurodegenerative disorders. Selective imaging of intracellular Cys fluxes by specific fluorescence probes has attracted a lot of attention. However, Cys-activated PSs are quite rare, and to the best of our knowledge, only two cases have been reported so far. Kolemen et al. modified the dicyanomethylene-4H-chromene (DCM) core with a heavy iodine atom at different positions and investigated two PSs (**88a**, **88b**) (Figure 38).^326^ It was found that the presence of the iodine atom by itself is not enough to obtain a high ^1^O_2_ generation yield, but the location of iodine is highly critical. **88a** with the iodine atom on the phenolate ring exhibited a much higher ^1^O_2_ quantum yield (Φ_Δ_ = 5.2%) than **88b** (Φ_Δ_ = 0.6%) where the iodine was placed on the chromone ring of the DCM core. Based on this result, they developed a Cys-activatable PS (**88c**) by masking the phenol of **88a** with a Cys-responsive unit (acrylic ester). Probe **88c** could be selectively activated by endogenous Cys, resulting in significant photocytotoxicity to HeLa cells (with high intracellular Cys concentrations) as compared to L920 cells (with low intracellular Cys concentrations), along with a “Turn-On” fluorescence output. In the same year, Kolemen et al. also developed a Cys-responsive pro-PS based on a chlorinated hemicyanine dye.^327^ Theranostic probe **89** exhibited a significant “Turn-On” NIR fluorescence and enhanced ^1^O_2_ generation efficacy (Φ_Δ_ = 1.8%) as well as photothermal conversion (η = 69%) after activated by Cys, triggering significant cancer cell death and causing no harm to normal cells. Overall, the same research group contributed two key cysteine-activated PSs and introduced a new gateway for cancer cell-specific therapeutics triggered by Cys.

#### 2.2.3 ROS/RNS/H_2_S-Activatable Theranostic Probes in PDT

Reactive oxygen/nitrogen species (ROS/RNS) are the natural byproducts of metabolism in living organisms.^328−330^ These chemically reactive substances play critical roles in cellular signal transduction and the maintenance of intracellular redox homeostasis.^331−333^ Due to the distinctive metabolic capacity between cancerous and normal cells, elevated levels of ROS/RNS have been discovered in cancer cells, as compared to their normal counterparts, suggesting a tight relationship between intracellular ROS/RNS and the occurrence and development of cancers.^334−338^ Moreover, there is accumulating evidence that the vast production of ROS/RNS inside the cancer cells can also lead to various kinds of cell death.^339^ Therefore, the exploration of specific ROS/RNS-responsive PSs represents an indispensable direction for the development of innovative cancer-targeting phototheranostics.

##### 2.2.3.1 H_2_O_2_-Activatable Theranostic Probes in PDT

**Figure 39:**
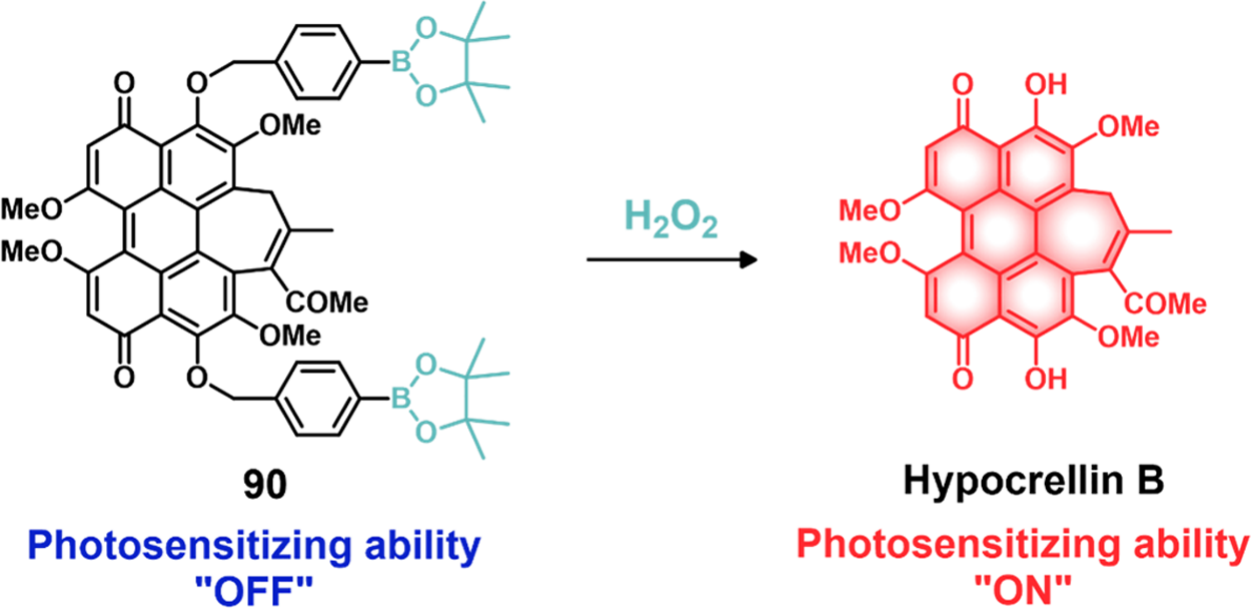
Activation of **90** by H_2_O_2_. Reproduced with permission from ref (342). Copyright 2022 Royal Society of Chemistry.

The exploration of stimulus-responsive PSs that utilize an oxidative activation strategy has gained increasing research interest.^339−341^ In particular, regarding the relatively higher chemostability and significantly larger concentration of H_2_O_2_ (5 μM to 1.0 mM) in tumorous cells, versus healthy cells,^335^ a vast number of H_2_O_2_-activatable phototheranostics with different types of PSs and diverse responsive mechanisms has been successfully constructed. For example, Takahashi and Toshima et al. studied a natural pigment hypocrellin B-derived pro-PS featuring an arylboronic ester (**90**, Figure 39) for the specific photodestruction of H_2_O_2_-overproducing cancer cells.^342^ Interestingly, upon modification of the two hydroxy groups of hypocrellin B with two H_2_O_2_-cleavable phenylboronic esters, the caged hypocrellin B mainly absorbed in the UV–vis region, and its photosensitizing ability was substantially attenuated. After the specific removal of phenylboronic esters by H_2_O_2_, the PS displayed a red-shifted absorption band in the phototherapeutic window (600–900 nm), and its ^1^O_2_-generating ability was restored. Additionally, light-initiated protein-destruction assays indicated a more pronounced degradation of BSA by hypocrellin B rather than its boronic ester-caged form, confirming the tunability of the photosensitizing ability. Importantly, photocytotoxicity assays suggested that the caged PS showed a considerably higher photocytotoxicity to H_2_O_2_-overexpressing cancerous cells (B16F10) than to normal WI-38 cells under 660 nm-light irradiation conditions, which emphasized the feasibility of designing innovative H_2_O_2_-activatable phototheranostics to reduce side effects for cancer-selective PDT.

**Figure 40:**
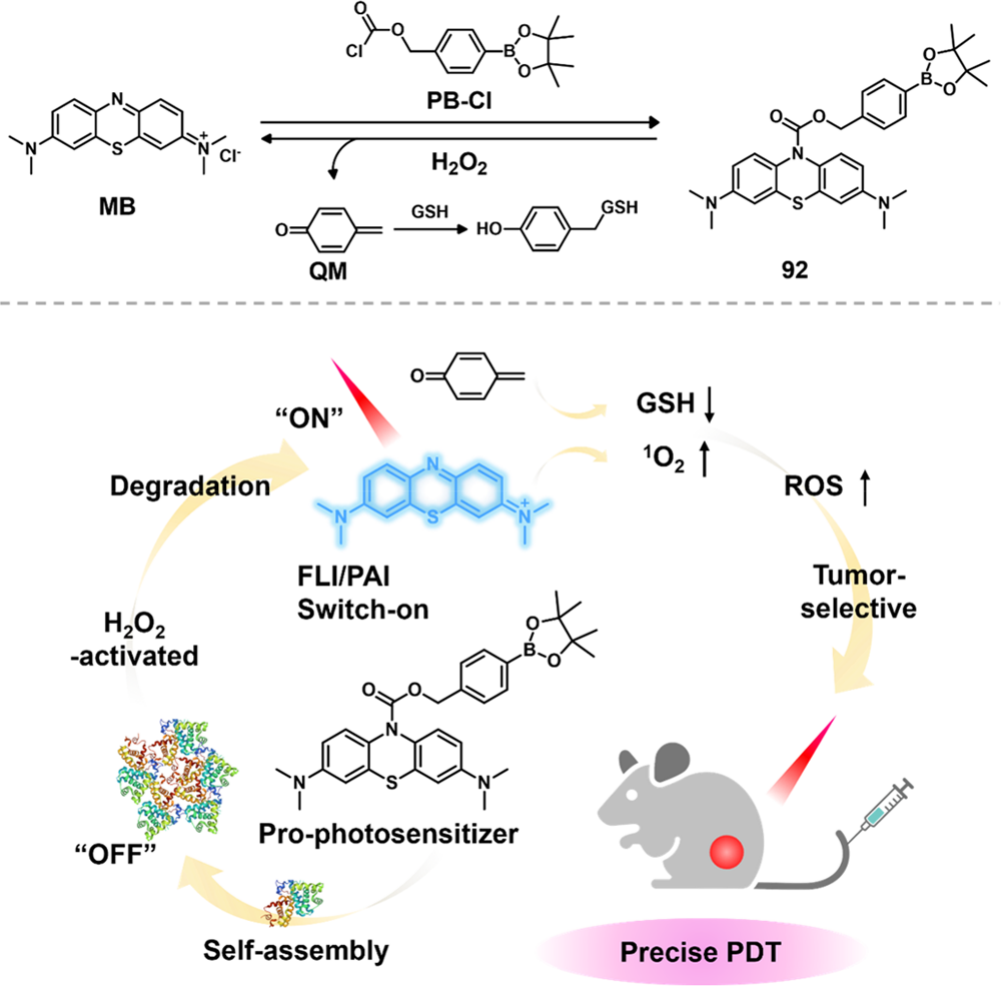
Synthesis of **91** for H_2_O_2_-activated bioimaging and amplified PDT via GSH depletion. Reproduced with permission from ref (343). Copyright 2019 Elsevier Ltd.

To improve tumor-targeting capabilities, Ding and Zhang et al. fabricated a dual-targeting nanophototheranostic consisting of a phenylboronic ester-modified methylene blue-based pro-photosensitizer (**91**, Figure 40) and biodegradable BSA for tumor-targeted imaging and PDT.^343^ Upon intravenous injection into a HepG2 cell-bearing tumor mouse model, this nanomedicine displayed specific accumulation in the tumor region via the notable EPR effect, and a selective deboronation reaction triggered by elevated levels of H_2_O_2_ in tumorous cells not only switched on the MB fluorescence but also restored its photosensitizing properties. Additionally, the side product, quinone methide, could rapidly and irreversibly deplete intratumoral GSH, leading to amplified oxidative stress, which synergized with the PDT effects. Notably, as MB is a clinically approved PS and the above-mentioned nanomedicine would rapidly degrade to biocompatible small molecules that can be readily excreted, this nanophototheranostic has good potential for future clinical translation.

**Figure 41:**
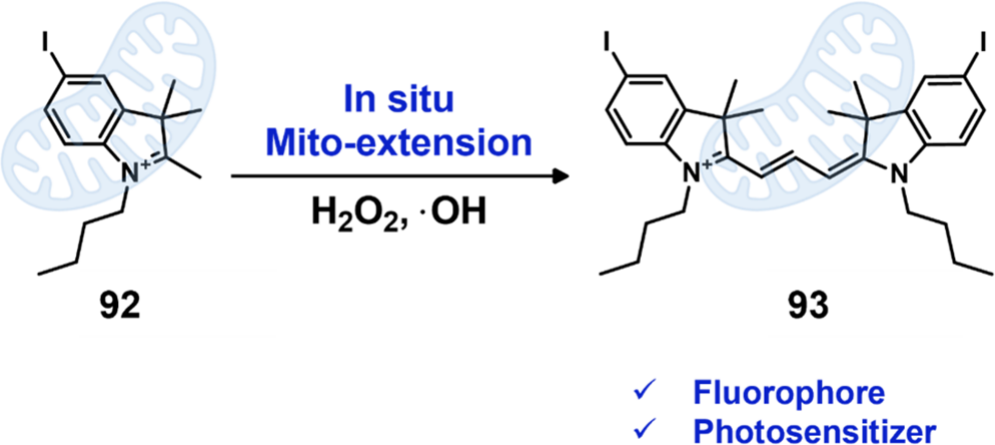
In situ generation of **93** via reaction with mitochondrial ROS for PDT. Reproduced with permission from ref (345). Copyright 2021 Wiley Intersciences.

Mitochondria play indispensable roles in anticipating cellular bioenergetic and metabolic activities. Innovative PDT agents that effectively induce mitochondrial dysfunction are highly attractive for cancer therapy.^344^ In this context, Feng and Wang et al. reported that the iodinate indolium (**92**), a common starting material for the synthesis of cyanine dyes, is capable of generating an iodinated trimethine cyanine dye (**93**, Figure 41) upon reaction with ROS, such as H_2_O_2_ and •OH.^345^ Importantly, as the TME features a higher concentration of ROS, and the cationic nature of **93** allowed its efficient accumulation in mitochondria, the intracellular ROS initiated the in situ generation of a cyanine-based bioimaging reagent and PS in living cancer cells. Moreover, the heavy atom effects rendered **93** a potent PS generating ^1^O_2_, resulting in remarkable photodamage of HeLa cells under light irradiation. This intracellular ROS-responsive in situ synthesis of a phototheranostic agent offers an exciting new avenue for preferential cyanine expression and PDT in tumors.

**Figure 42:**
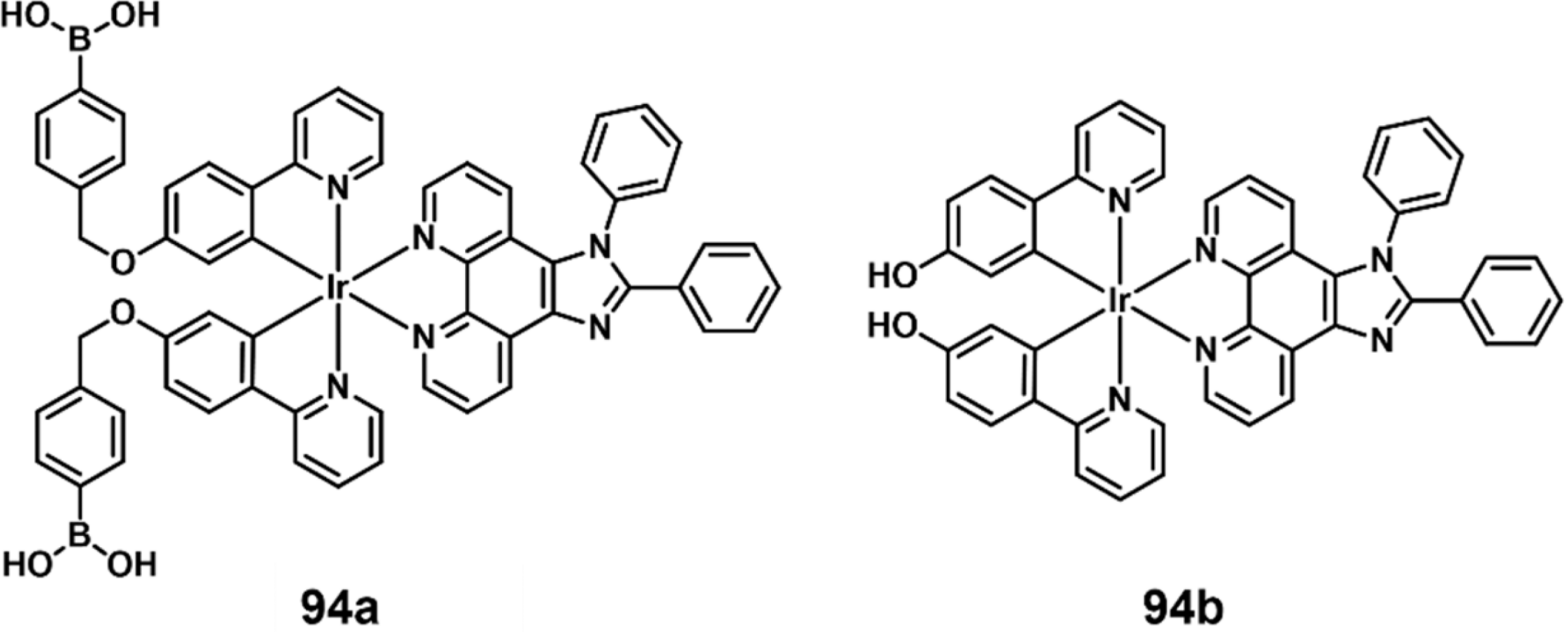
Chemical structures of theranostic probes **94a** and **94b**.

Luminescent transition metal complexes are considered promising alternatives to organic phototheranostics due to their competitive photophysical properties and excellent photosensitizing abilities.^346^ Transition metal complex-based PSs are gaining interest in PDT, with one of them already undergoing clinical trials.^347^ To take advantage of these benefits, Chao et al. developed a phenylboronic ester-decorated cyclometalated iridium(III) prodrug (**94a**, Figure 42). This prodrug was then encapsulated into selenium nanoparticles and cancer cell membranes to enhance cancer targeting, membrane permeability, and pharmacokinetics. Selenium nanoparticles were chosen as nanocarriers for their drug-loading capacity and biocompatibility. Inside cancer cells, selenium nanoparticles decompose, releasing selenium, which disrupts mitochondrial function and induces cell apoptosis. In the presence of intracellular H_2_O_2_, the nanoformulation rapidly decomposes, resulting in the release of an iridium(III) PS (**94b**), a GSH scavenger (quinone methide), and a chemotherapeutic agent (selenium nanoparticles). Under two-photon irradiation (730 nm), the generated ^1^O_2_ works synergistically with the reduced intracellular GSH and the chemotherapeutic effect of selenium nanoparticles, effectively eradicating a melanoma tumor in a mouse model. This combined PDT and chemotherapy strategy opens new possibilities for designing intelligent phototheranostics for cancer therapy.

**Figure 43:**
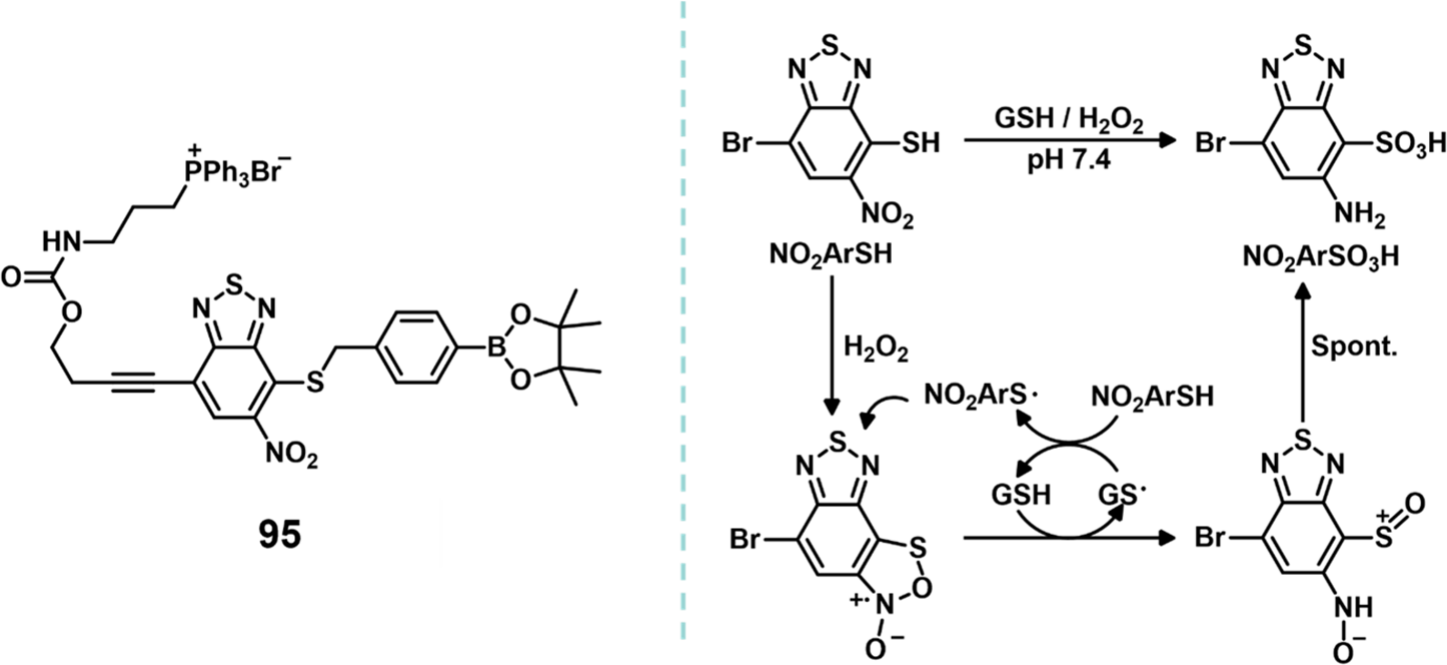
Chemical structure of **95** and an illustration of its activation mechanism.

The emergence of molecular logic has led to active research in developing multiresponsive fluorescent probes that can react to two or more distinct substrates.^348^ Dual-activatable theranostic agents are typically highly selective toward cancer cells.^349^ Feng and Wang et al. presented a coactivatable phototheranostic probe (**95**, Figure 43) that can be activated by both H_2_O_2_ and GSH, specifically targeting mitochondria and enabling tumor-specific PDT.^350^ Notably, probe **95** did not react with GSH alone, even with its high concentration in mitochondria. It required the sequential action of intratumoral H_2_O_2_ and GSH to activate, thus forming an efficient PS that avoids nonspecific activation by other redox-active biomolecules. The activated PS demonstrated the simultaneous generation of both ^1^O_2_ and superoxide radicals (•O_2_^–^) for PDT, helping alleviate the hypoxic microenvironment in solid tumors. This is because type I PSs are significantly less oxygen-dependent. Additionally, the PS could be excited by two-photon irradiation (808 nm), overcoming the limitation of poor penetration depth associated with short excitation wavelengths. This research emphasizes the utilization of the tumor microenvironment as a stimulus for the activation of dual-locked phototheranostics.

**Figure 44:**
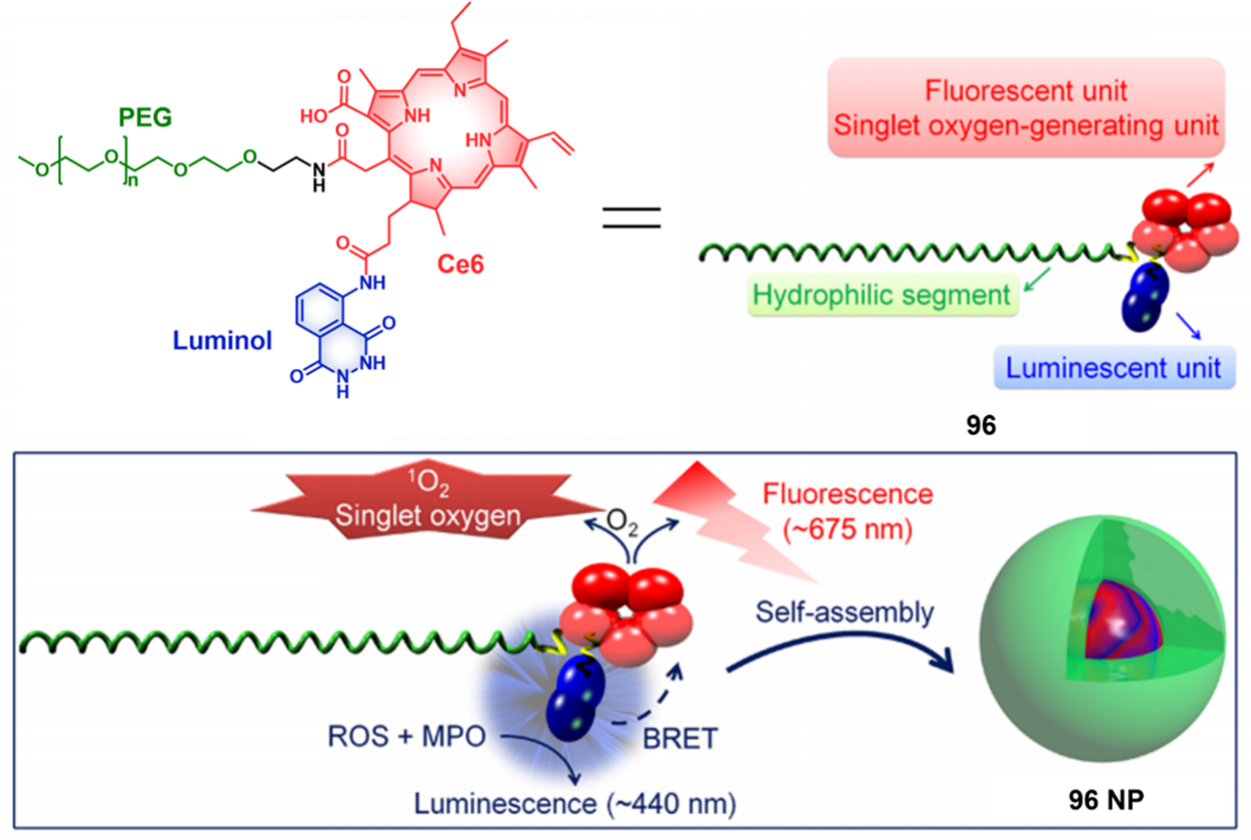
Chemical structure of **96** and an illustration of its activation mechanism. Reproduced with permission from ref (352). Copyright 2019 Science Publishing Group.

The limited penetration depth of the excitation light source for PSs is one of the major obstacles to deep tissue PDT. NIR light penetrates deeper but is still limited to only a few centimeters.^351^ To solve this problem, Xu and An designed a self-illuminating unimolecular nanoparticle for luminescence imaging of inflammation and tumor PDT in vivo via a luminol-modified and PEG-tethered Ce6 PS (**96**, Figure 44).^352^ The PS self-assembled from an amphiphilic polymeric conjugate to appropriate-sized nanoparticles. Interestingly, the increased H_2_O_2_ concentration and corresponding activating enzymes in the TME initiated luminol bioluminescence, which was subsequently absorbed by the Ce6 unit to trigger the generation of ^1^O_2_ through efficient bioluminescence energy transfer, facilitating in vivo imaging and deep tissue PDT, respectively. As this chemiluminescence energy transfer-based phototheranostic system does not require the use of an external light source, it is considered a more favorable therapeutic modality compared to traditional PDT.

##### 2.2.3.2 ClO^–^/HClO-Activatable Theranostic Probes in PDT

**Figure 45:**
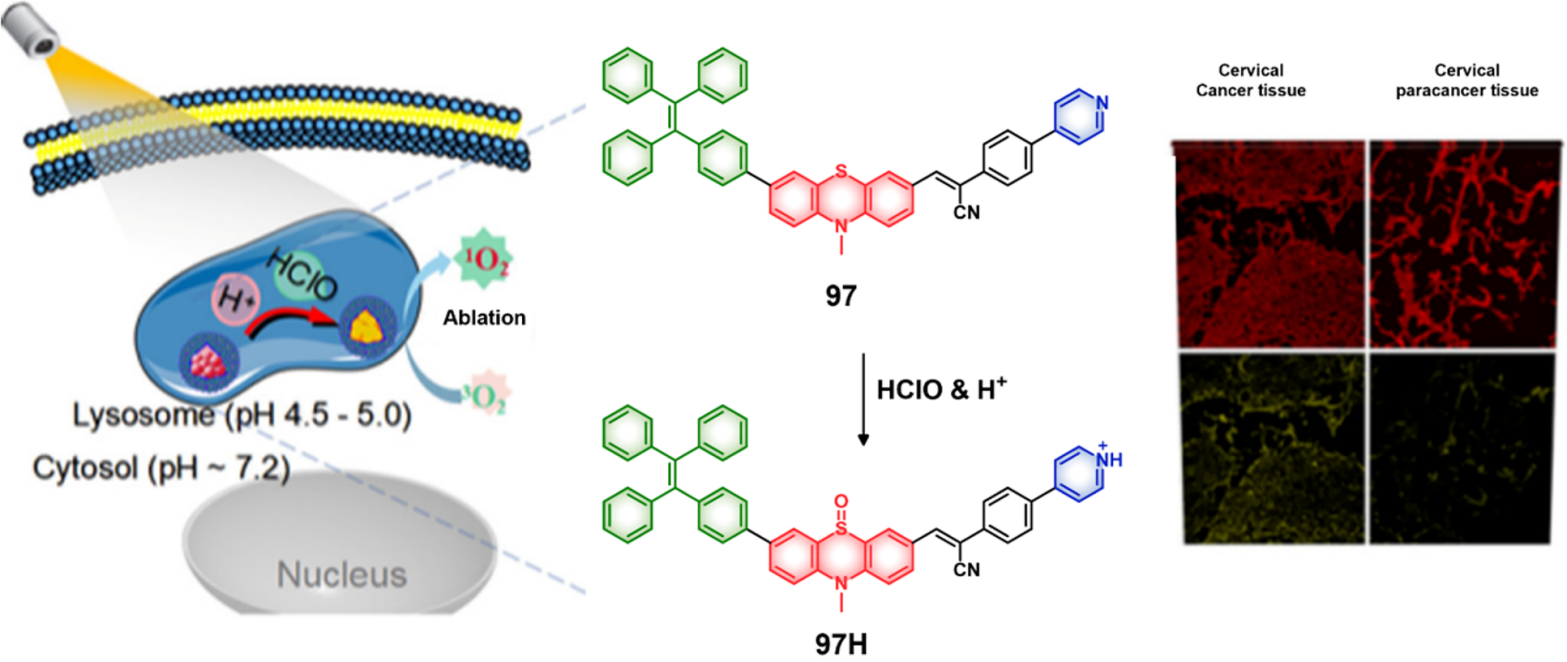
Schematic of synergistic dual-stimuli-regulated **97** by HClO and H^+^ andfluorescence imaging of cervical cancer tissue and paracancer tissue pretreated with TPE-PTZ-Py and TPE-PTZO-Py, respectively. Red channel: λ_ex_ = 425 nm, λ_em_ = 635–675 nm; yellow channel: λ_ex_ = 425 nm, λ_em_ = 505–545 nm (laser power = 2 mW). Reproduced with permission from ref (354). Copyright 2022 American Chemical Society.

Hypochlorite/Hypochlorous acids (ClO^–^/HClO), which are enzymatically produced by the intracellular enzyme myeloperoxidase, play essential roles in living organisms and are closely associated with many pathological processes, such as bacterial infection, cardiovascular and neurodegenerative diseases, arthritis and cancer.^353^ A higher level of ROS including ClO^–^/HClO facilitates oncogenic transformation and drives the cancer cells to proliferate.^328^ The basal ClO^–^/HClO levels in cancerous cells are estimated to reach ca. 10 nM,^336^ implicating that facile cancer-targeting might be realized by ClO^–^/HClO-activatable phototheranostic agents. To employ this strategy, Wang and Zhang et al. reported monocomponent nanodots with lysosome-localizing properties to specifically visualize and enable PDT of cervical tumors.^354^ Interestingly, these Pluronic F127-encapsulated nanodots featuring an AIE-active phenothiazine derivative (**97**, Figure 45) could respond ratiometrically to elevated ClO^–^/HClO levels in tumorous tissues, which gave rise to the production of an increased level of ^1^O_2_ (nearly 2-fold more efficient than commercial Rose Bengal) and elicited strong photocytotoxicity (IC_50_ < 7.6 μM). Furthermore, bioimaging studies implicated that these nanodots could be used to visualize the fluctuation of ClO^–^/HClO in human cervical tissues. This research represents a pioneering ClO^–^/HClO-responsive nanodrug for the PDT treatment of inflammation-related tumors without causing severe side effects.

**Figure 46:**
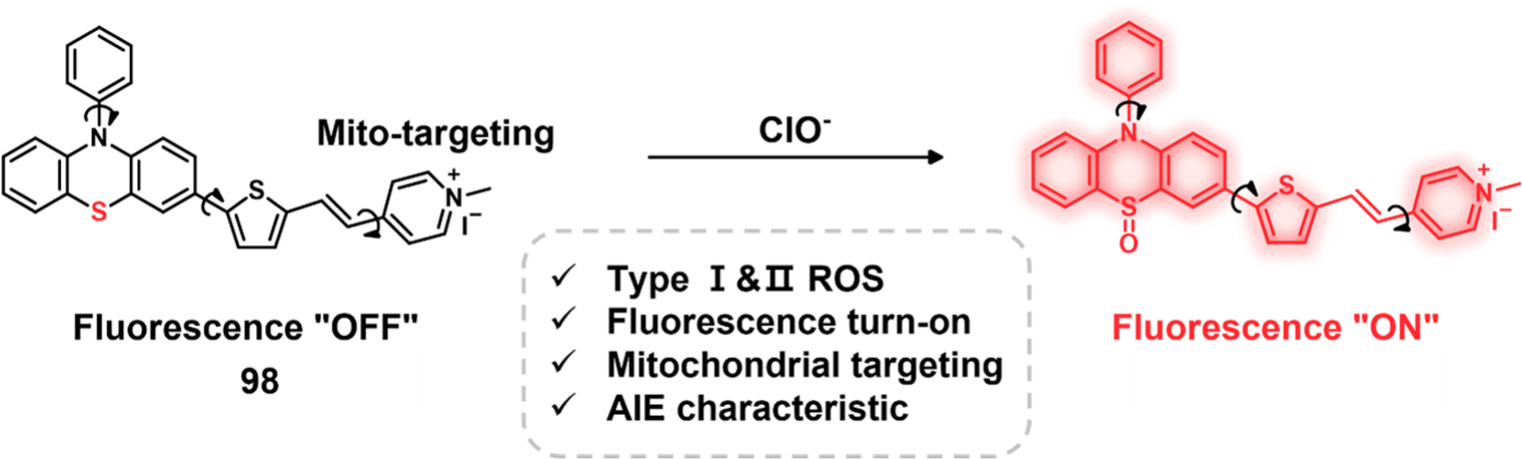
Chemical structure of **98** and activation by ClO^–^. Reproduced with permission from ref (355). Copyright 2023 Elsevier Ltd.

To date, numerous ClO^–^/HClO-responsive fluorogenic probes have been developed and applied for both in vivo and in vitro imaging studies. However, ClO^–^/HClO-responsive PSs that integrate both type I and II photosensitizing mechanisms remain rarely explored. In one recent example, Tang and Han et al. reported a mitochondria-targeted and bioimaging-enabled PDT agent by using an AIE-based PS (**98**, Figure 46) that is specifically activated by intratumoral hypochlorite (ClO^–^).^355^ Intriguingly, PS **98** displayed type II photosensitizing features before reacting with ClO^–^, predominantly generating ^1^O_2_ upon irradiation, while upon converting to its oxidized counterpart, bright emission accompanied by type I radical generation was observed. Further studies suggested that PS **98** was able to differentiate tumorous cells from normal cells, rendering this compound a promising phototheranostic agent for tumor-seeking PDT.

**Figure 47:**
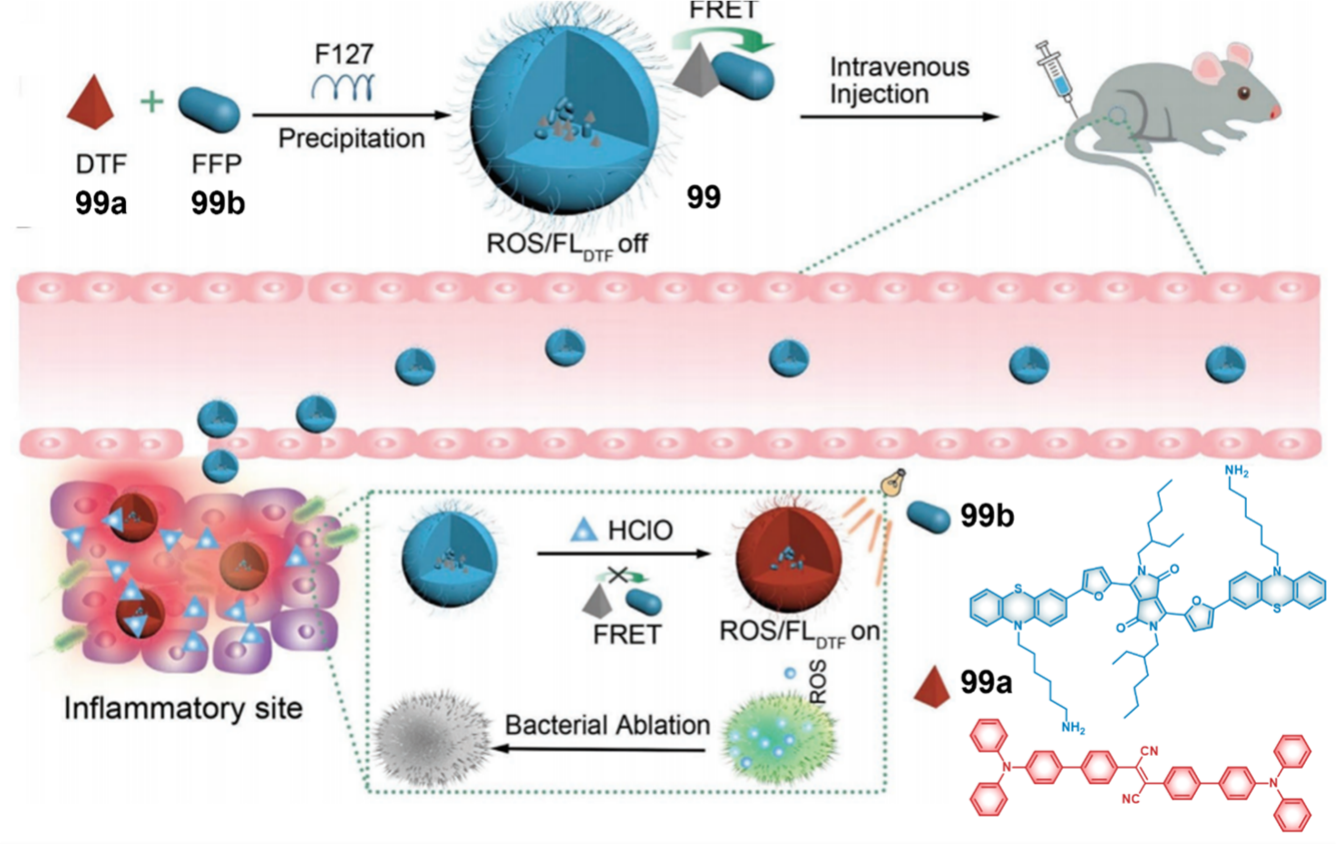
Preparation of **99** NPs and their fluorescence and photosensitization activation by HClO at the bacterial inflammatory site for effectively imaging and ablating the bacteria inside invaded phagocytes. Reproduced with permission from ref (356). Copyright 2020 Wiley Intersciences.

HClO-activated PDT has also been applied to antibacterial studies. Liu and Tang et al. formulated an HClO-activatable nanomedicine that consists of an AIE-type PS (DTF, **99a**) and an HCIO-responsive probe (FFP, **99b**, Figure 47) to detect and treat bacterial inflammatory diseases in vivo.^356^ The nanoformulation (**99**) was easily prepared by encapsulating **99a** and **99b** into Pluronic F127. The coexistence of these two substances leads to a quenched photosensitizing ability, owing to the efficient Förster resonance energy transfer process between **99a** and **99b**. However, upon oxidative destruction of the phenothiazine moiety by endogenous HClO, **99b** displayed a dramatic decrease in both absorption and emission intensities, while the emission of **99a** remained unchanged, contributing to the disruption of the energy transfer process and the recovery of the photoinduced ^1^O_2_ generation. Physicochemical characterization revealed that nanoparticles (**99**) were highly selective toward HClO over other biological ROS. This nanomedicine has been successfully applied to the tracking of the infection site, which enables in vivo antibacterial PDT. Furthermore, the inhibitory effect of the nanoparticles against *S. aureus* could last up to 7 days, which was remarkably longer than that of the common antimicrobial drug vancomycin, which only exhibited an antimicrobial effect during 72 h. In conclusion, this HClO-stimulated phototheranostic nanoformulation with tunable luminescence and photosensitizing behavior offers a desirable approach toward infection-origin nanotheranostics.

##### 2.2.3.3 NO-Activatable Theranostic Probes in PDT

**Figure 48:**
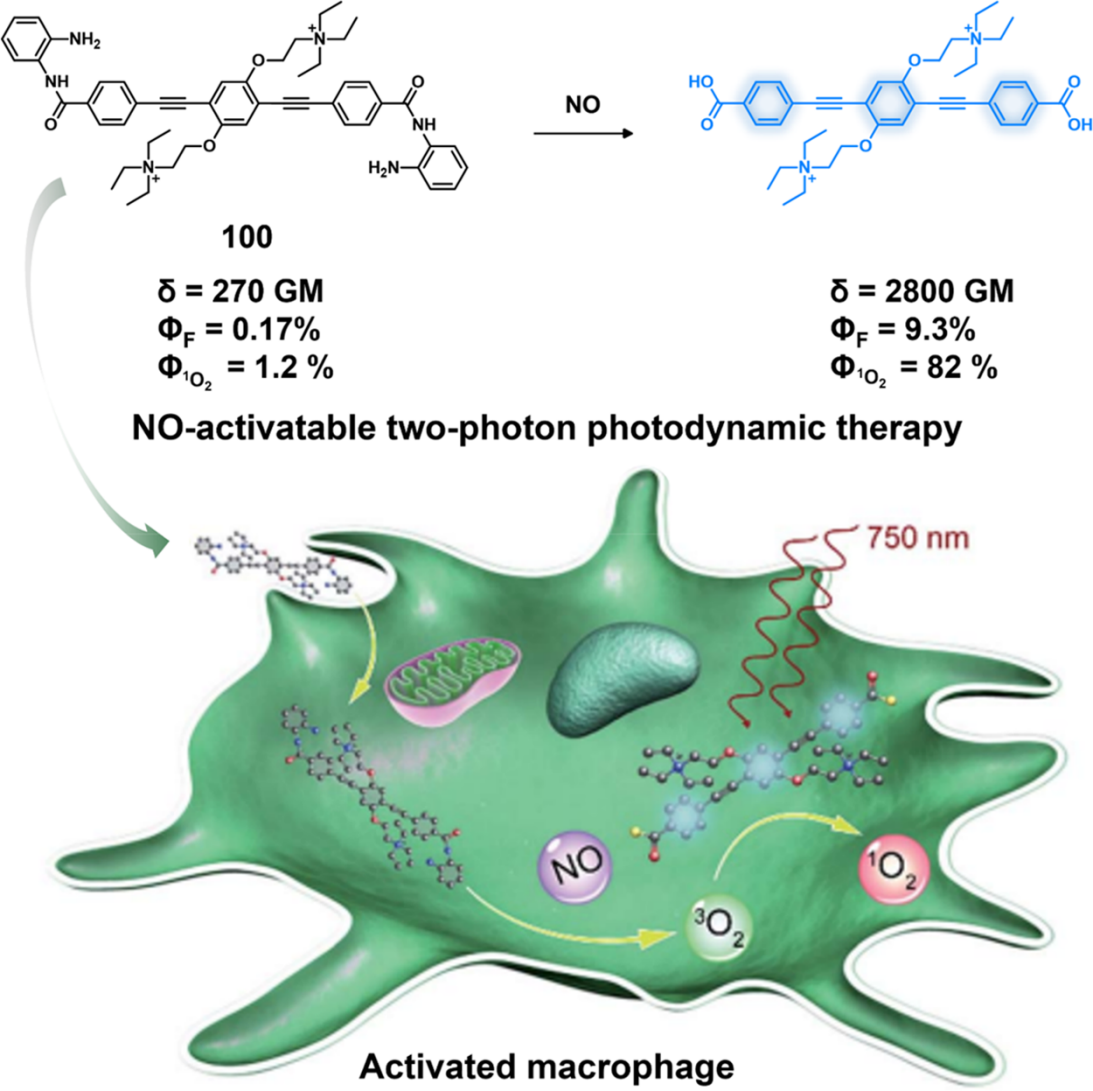
Molecular structure and activation of **100** by NO and schematic illustration of the NO-activatable 100 for TP-imaging and TP-PDT in an activated macrophage (in which NO is upregulated). Reproduced with permission from ref (359). Copyright 2018 Royal Society of Chemistry.

An elevated NO concentration in cancer tissues is usually produced by the enzymatic reaction of intracellular guanidyl-containing substrates and endogenous inducible nitric oxide synthase (iNOS),^337,338^ which has been utilized as an initiator to activate anticancer phototherapy.^357^ NO plays crucial roles in many inflammation-associated diseases.^337,338,358^ Considering these points, Fan et al. developed the first example of a NO-activatable and two-photon excitable fluorogenic pro-PS (**100**, Figure 48) for bioimaging and PDT applications.^359^ Upon reaction with NO in cancerous cells, **100** displayed not only a markedly improved emission quantum yield (more than 50-fold higher) and a photoinduced ^1^O_2_ generation quantum yield (up to 82%) but also an exponentially increased TPA cross-section (up to 2800 GM), providing proof-of-concept support for bioapplications of NO-triggered two-photon fluorescence imaging and PDT in lipopolysaccharide and interferon-γ coactivated macrophages. Regarding the fact that inflammation predisposes tissues to develop cancer and facilitates the tumorigenic process, the potential of NO-activatable cancer phototheranostics has not been fully exploited, and we anticipate that more intricate NO-activatable PSs will be developed for the treatment of inflammatory cancers in the future.

##### 2.2.3.4 Peroxynitrite-Activatable Theranostic Probes in PDT

**Figure 49:**
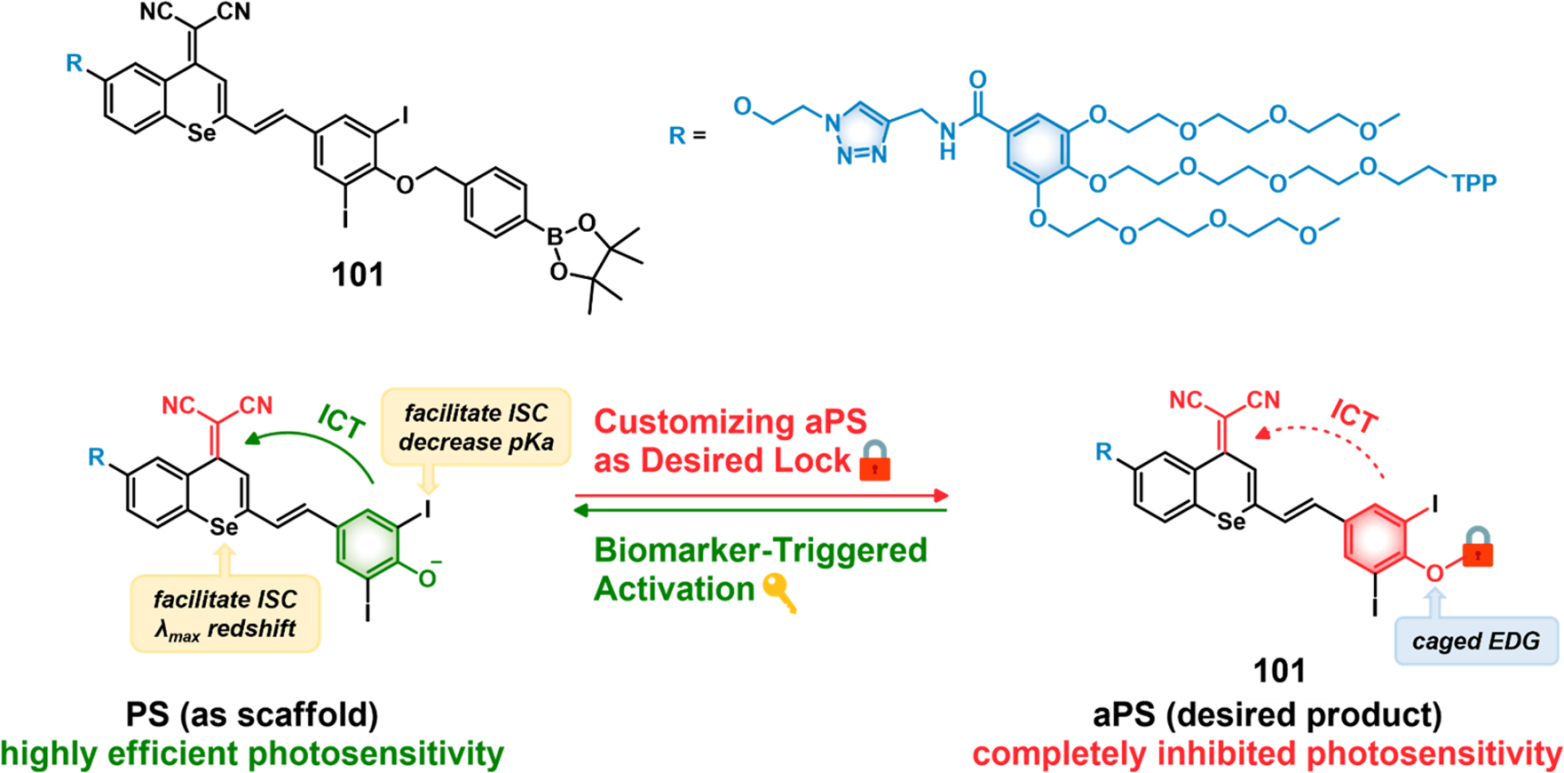
Chemical structure of **101** and schematic illustration of the proposed PS activation strategy. Reproduced with permission from ref (360). Copyright 2019 Wiley Intersciences.

Peroxynitrite (OONO^–^) refers to a highly oxidative RNS generated by •O_2_^–^ and NO and is a signal-transducing molecule that plays diverse roles in cellular processes. OONO^–^ has been regarded as a crucial biomarker for many cancers. Consequently, Li et al. explored a proof-of-principle design, based on inhibiting the photosensitizing process, to yield a pro-PS whose photosensitivity could be restored from a suppressed state after reacting with this specific biomarker. A mitochondria-anchoring long-wavelength excitable pro-PS (**101**, Figure 49) with highly efficient activable photosensitivity for the production of ^1^O_2_ was developed.^360^ Protecting the phenol hydroxy moiety by an OONO^–^-cleavable functional group offered a simple method for designing smart PSs. A clear activation of the photosensitizing ability of **101** by raised OONO^–^ concentrations in living cancer cells was demonstrated. To further improve the solubility of the PS, several PEG chains with a mitochondria-targeting moiety were introduced. Superior bioimaging performance as well as an outstanding PDT efficiency in tumor-related RAW 264.7 cells was observed.

**Figure 50:**
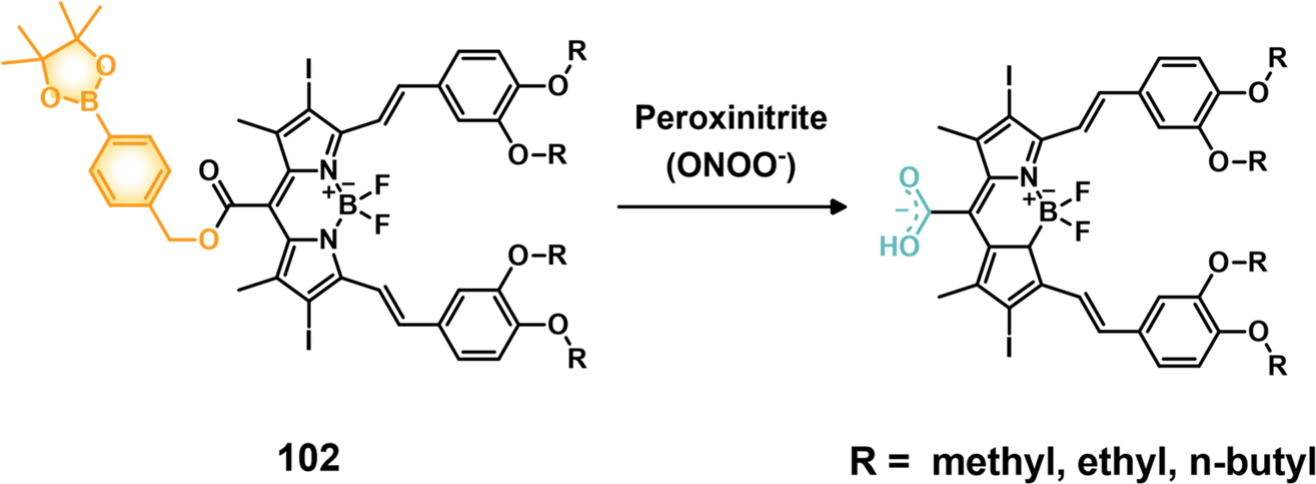
Activation of **102** by OONO^–^. Reproduced with permission from ref (361). Copyright 2019 American Chemical Society.

Manipulating the molecular packing modes of PSs is a facile and efficient way to optimize their photosensitizing performance. Based on this, Li et al. proposed an effective approach for the preparation of biocompatible J-aggregated nanoparticles by integrating amphiphilic polymeric micelles and an OONO^–^-activatable iodinated BODIPY dye (**102**, Figure 50).^361^ Dye **102** tends to self-assemble into stable plate-like core–shell nanoarchitectures in PEG-PCL-derived polymeric micelles. Interestingly, these nanoassemblies displayed an intrinsic OONO^–^ responsiveness as a result of the phenylboronic ester-modified *meso*-carboxylate moiety, which could be effectively detached upon the reaction with endogenous OONO^–^, resulting in reassembly from J-aggregates to amorphous nanospheres, by virtue of the exposure of the generated negative charges. Accordingly, the photoinitiated ^1^O_2_ generation of the nanoparticles was turned on, which was further employed for the selective photoeradication of tumor-associated macrophages, expressing elevated OONO^–^ under the influence of LPS and INF-γ. In consideration of the easy access and simple activating principle, this research opens up a new avenue for developing stimuli-responsive phototheranostics that meet clinical needs.

**Figure 51:**
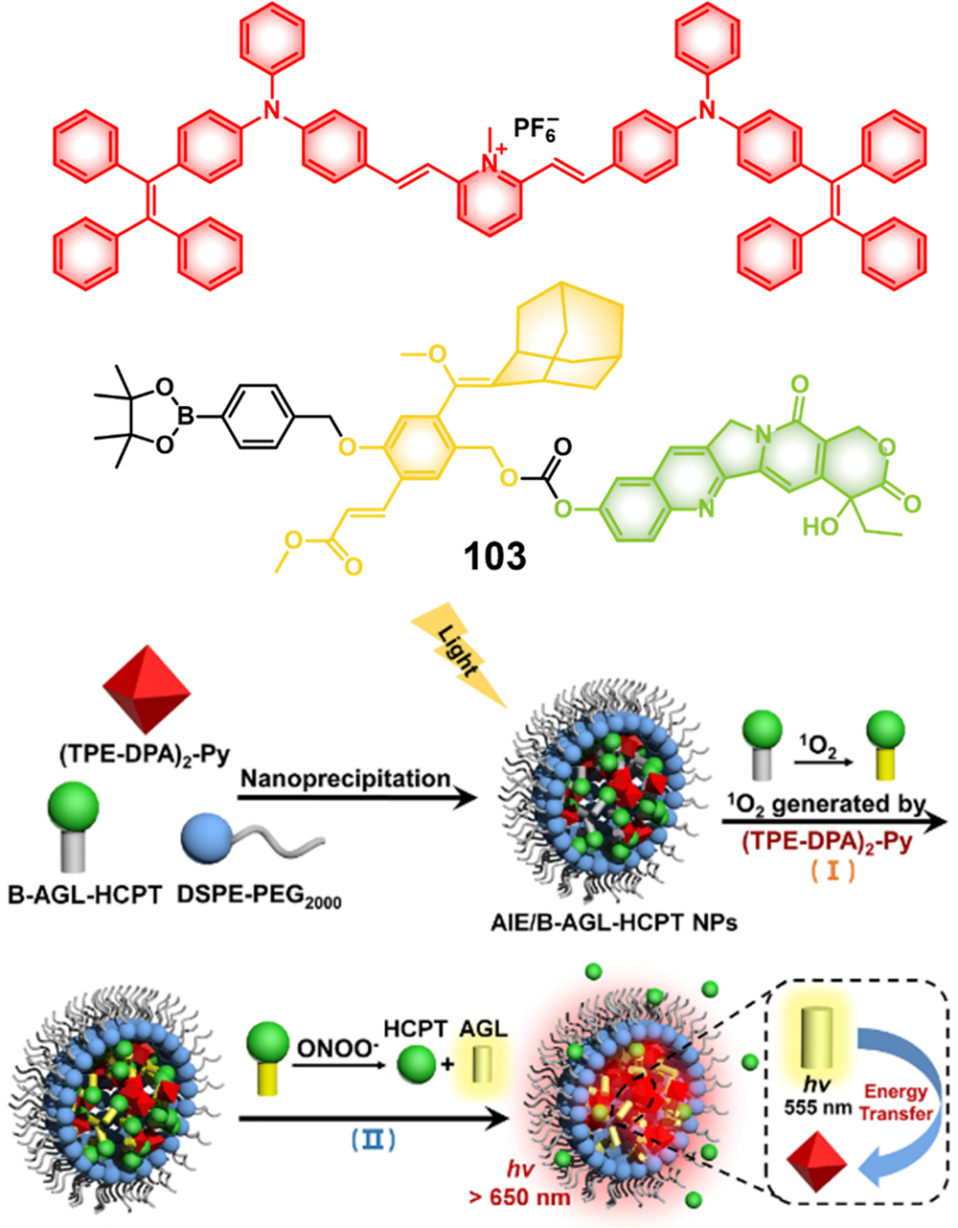
Chemical structure of **103** and schematic showing the AIE/B-AGL-HCPT NPs fabrication and ONOO^–^-mediated activation of drug release and NIR afterglow luminescence within preirradiated AIE/B-AGL-HCPT NPs. Reproduced with permission from ref (362). Copyright 2022 Wiley Intersciences.

Persistent luminescent materials possess great potential for bioimaging and phototherapeutic applications, owing to their unique ability to retain photon-releasing properties after the removal of incident light exposure or to exploit certain chemical reactions to fuel chemiluminescence. Luminescence can minimize the interference of tissue autofluorescence and thus holds a significantly higher signal-to-noise ratio, which is preferable for biomedical applications. Nevertheless, the clinical application of persistent luminescent materials still suffers from some severe disadvantages, such as undesired cytotoxicity, synthetic difficulties, and unsatisfactory excitation/emission wavelengths. To address these issues, Ding et al. contributed a phototheranostic prodrug nanoassembly (**103**, Figure 51) that not only exhibited self-reporting drug-release behavior but also prominently stimulated immunogenic cell death processes upon activation of afterglow emissions in the near-infrared region.^362^ The nanoassemblies are fabricated by sealing an ONOO^–^-activable afterglow prodrug **103** and an AIE-active PS with NIR emission into DSPE-PEG_2000_ nanoparticles. To maximize the photocytotoxity, the nanoassemblies were first photoirradiated to produce certain amounts of ^1^O_2_, which reacts with the prodrug to afford an afterglow-active 1,2-dioxetane precursor, that in turn is activated in the presence of endogenous ONOO^–^, triggering the release of the anticancer drug hydroxycamptothecin and initiating the afterglow luminescence. Moreover, the activatable afterglow luminescence continues to excite the AIE-based PS via resonance energy transfer to give NIR emission and large amounts of ^1^O_2_, which amplifies the anticancer effects. Additionally, the released anticancer drug hydroxycamptothecin could sensitize the “cold” tumor to photochemotherapy. Further studies revealed that these nanoassemblies can eradicate tumors and prevent tumor recurrence.

##### 2.2.3.5 H_2_S-Activatable Theranostic Probes in PDT

**Figure 52:**
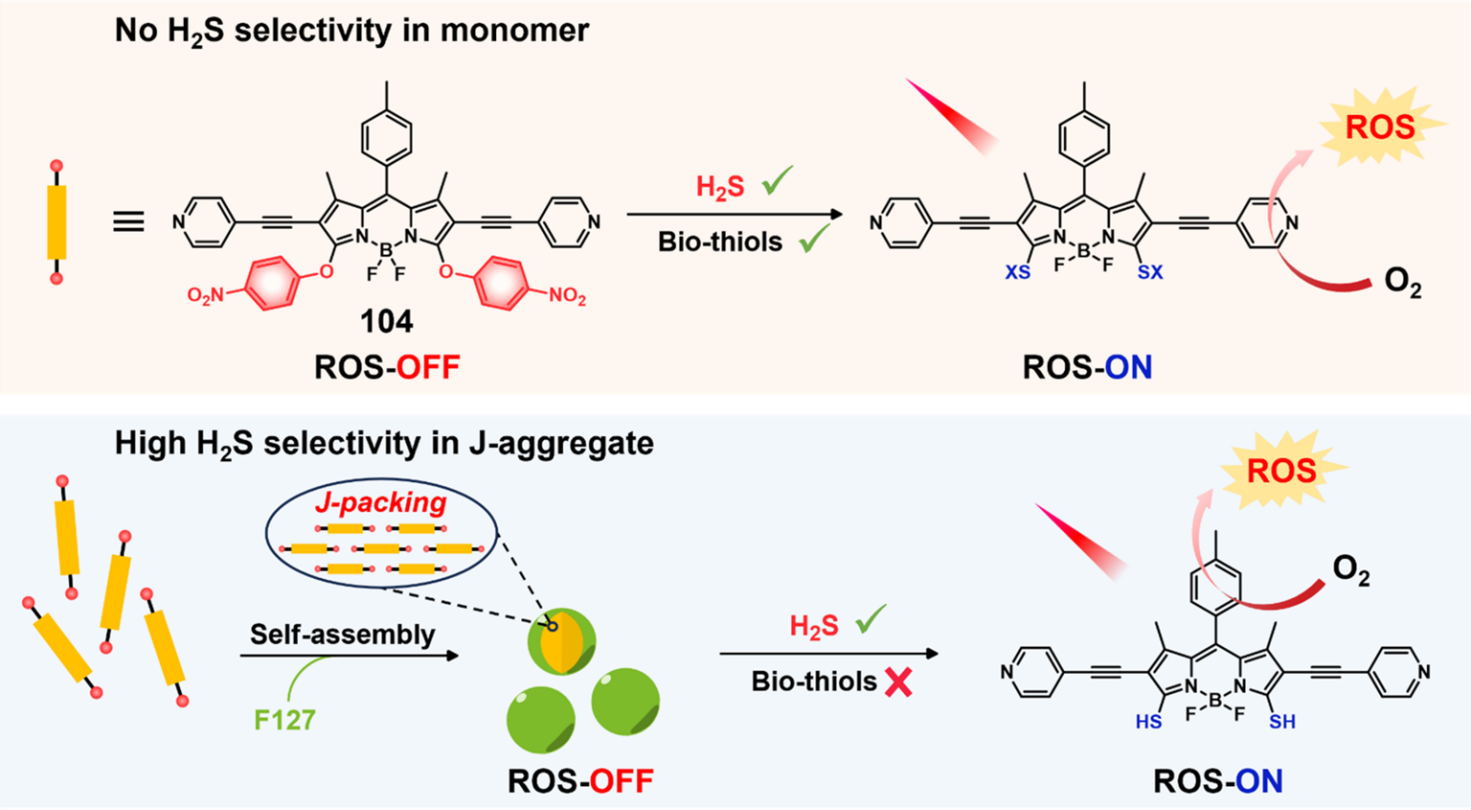
Schematic illustration of the insufficient H_2_S selectivity of free **104** and self-assembly enhanced selectivity toward H_2_S of templated self-assembly of **104**. Reproduced with permission from ref (365). Copyright 2022 Royal Society of Chemistry.

Hydrogen sulfide (H_2_S), a well-known nucleophile and reductant, is noted for its gasotransmitter role that involves numerous biological processes.^363^ Accumulating evidence points to increased concentrations of H_2_S (ca. 0.3–3.4 mM) in cancer cells, in particular, colon cancers.^364^ However, due to a complicated TME and the competitive cross interference by cellular GSH (ca. 0.5 to 10 mM), specific phototheranostics that only show a response to H_2_S remain a considerable challenge. One example of a selective phototheranostic was described by Yang et al. The authors discovered that self-assembled nanoaggregates with dense molecular packing would only allow small molecules like H_2_S to diffuse inside and could prevent unwanted interference from GSH, which is too large in size, thus realizing H_2_S-specific activation of a phototheranostic.^365^ A nitrophenyl ether-attached BODIPY derivative (**104**, Figure 52) has been designed and self-assembled in PF127 micelles to form orderly packed J-aggregates with a particle size of approximately 120 nm. Upon treatment with H_2_S, the blue absorption at around 623 nm gradually diminished together with a bathochromic shift in fluorescent emission, which could be attributed to the nucleophilic attack of the nitrophenyl ether by H_2_S, resulting in double −SH-substituted BODIPYs. Subsequent bioimaging and photocytotoxic studies have confirmed the effective responsiveness of the nanoassemblies toward endogenous H_2_S. Under light irradiation, the nanoassemblies exhibited significantly higher cytotoxicity with lower IC_50_ values against HeLa cells compared to dark controls. This represents the first example of applying J-aggregated pro-PS for H_2_S to achieve ultrahigh specificity over other biothiols.

**Figure 53:**
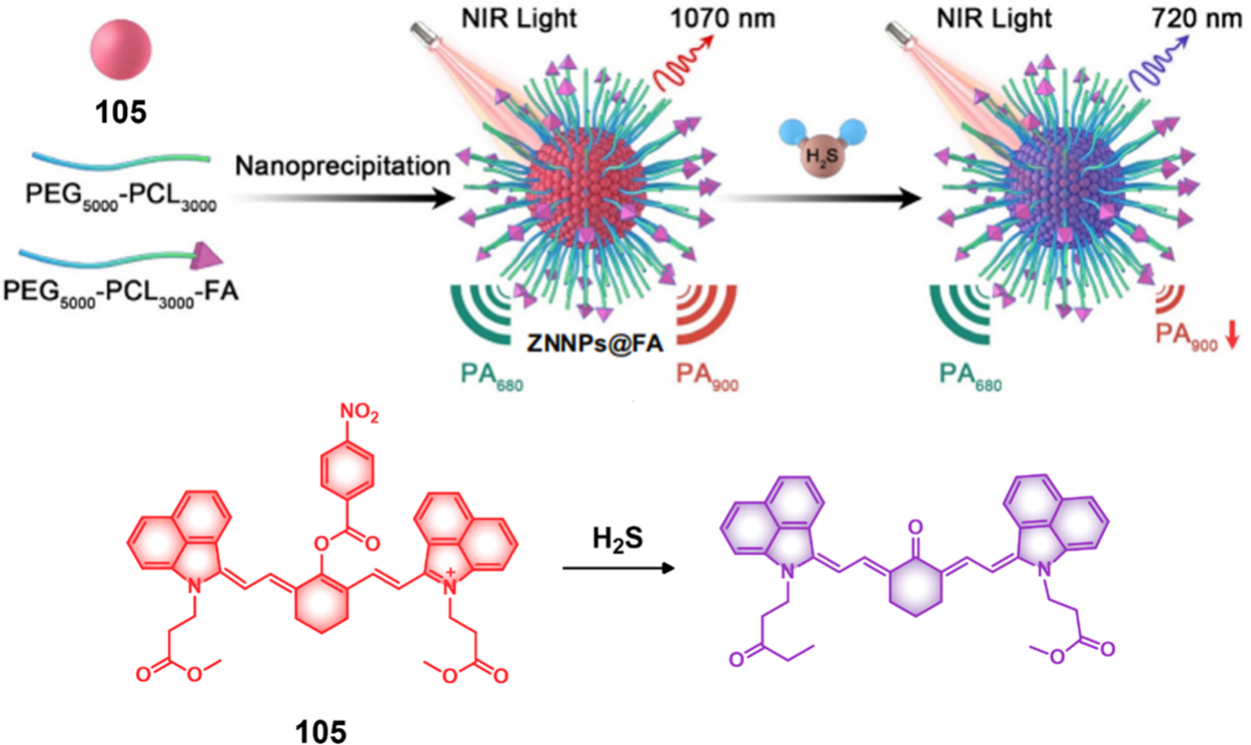
Fabrication of ZNNPs@FA and the principle of quantitative visualization of H_2_S are indicated by **105**. Reproduced with permission from ref (366). Copyright 2022 The Author(s).

Supplementing nitrophenyl ethers as H_2_S-sensitive triggers, Shi et al. reported on a nitrophenyl carboxylic ester modified π-extended heptamethine cyanine derivative (**105**, Figure 53), which showed an unprecedented NIR-II to NIR-I fluorescent response (*F*_1070_ → *F*_720_) and photoacoustic response (*PA*_680_/*PA*_900_) to nucleophilic attack by H_2_S, providing an exceptional opportunity for imaging the H_2_S content in various disease models, including tumors in living mice.^366^ To apply **105** to biomedical applications, it was self-assembled into polymeric nanoparticles using mPEG_5000_-PCL_3000_ as a stabilizer and mPEG_5000_-PCL_3000_-FA as a cancer-targeting group. The nanoparticles retained a similar H_2_S responsiveness and demonstrated improved ^1^O_2_ photogeneration ability upon H_2_S activation. In vivo studies revealed an H_2_S-activatable photocytotoxicity toward colorectal HCT116 cancer cells and subcutaneous tumors, making these nanoassemblies promising for treating cancers with elevated H_2_S levels.

**Figure 54:**
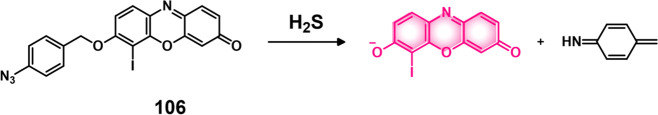
Activation of theranostic probe **106** by H_2_S.

As noted above, H_2_S is also a strong reductant, a property that has also been exploited for specific H_2_S probes. Azido-containing aromatics are among the most investigated H_2_S-responsive sensory moieties. Based on an azido phenyl ether-functionalized phenoxazine dye, Gunbas et al. developed an H_2_S-activatable iodinated resorufin pro-PS (**106**, Figure 54) for the photodynamic treatment of neuroblastoma-derived cancer cells.^367^ Pro-PS **106** turned out to be an excellent bioimaging reagent and phototherapeutic because it can selectively switch on its red emission at 606 nm and has good photoinduced ^1^O_2_ generation ability (Φ_Δ_ = 0.42) upon reaction with endogenous H_2_S. Surprisingly, **106** displayed markedly high photocytotoxicity (IC_50_ = ca. 3.29 μM) to H_2_S-overexpressed human neuroblastoma cells (SH-SY5Y) but was minimally photocytotoxic (IC_50_ > ca. 3.29 μM) to H_2_S-lacking noncancerous fibroblast cells (L929). One of the main drawbacks of this study may be the short excitation wavelength (<650 nm), which provides limited penetration depth in tissues. On the other hand, designing NIR-absorbing resorufin derivatives could overcome this issue.

##### 2.2.3.6 Bio-orthogonal-Activatable Theranostic Probes in PDT

**Figure 55:**
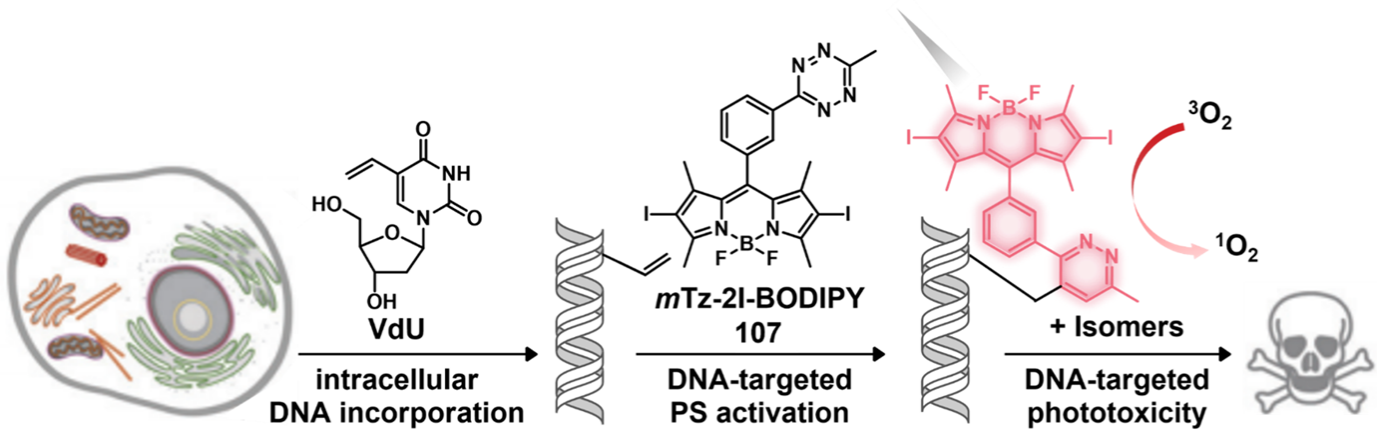
Chemical structure of **107** and outline of the DNA-targeted bio-orthogonal strategy for activating the phototoxicity. Reproduced with permission from ref (372). Copyright 2019 Wiley Intersciences.

The past two decades have seen exponentially increasing research interest in the exploration of bio-orthogonal chemistry for bioimaging and/or biomedical applications.^304,368−371^ Differing from conventional bioimaging reagents, bio-orthogonal theranostics typically involve a two-step labeling process: (1) living cells/organisms are first tagged with unique substrates with high bio-orthogonal reactivity, which can be metabolically anchored on certain parts of cells or organisms; (2) subsequently, the anchored substrates are specifically recognized and covalently integrated with another complementary bio-orthogonal luminogen. This two-step labeling approach not only offers extremely high spatial and temporal resolution but also allows facile visualization of certain subcellular organelles that can hardly be accomplished by traditional dyes. With respect to PDT, bio-orthogonal chemistry has been reported to be able to improve the cancer-targeting specificity and decrease the dark cytotoxicity and undesirable adverse effects of PSs. For example, Vázquez et al. studied the application of a bio-orthogonal activation strategy for PDT to realize tunable organelle-specificity and controllable photocytotoxic effects.^372^ An iodinated BODIPY featuring a tetrazine unit as a quenching group (**107**, Figure 55) was optimized to display maximal activation efficiency when reacted with a vinyl-bearing substrate. Notably, the bio-orthogonal activation not only turned the ^1^O_2_ generation quantum yields but also triggered a substantial photocytotoxic effect on the subcellular nucleus by metabolically preintegrating 5-vinyl-2′-deoxyuridine into the nuclear DNA. These gratifying results reinforced the feasibility and extended our vision toward various bio-orthogonal reaction pairs. Although the excitation wavelength of **107** is too short, these short wavelength-responsive probes could find applications in preclinical studies.

**Figure 56:**
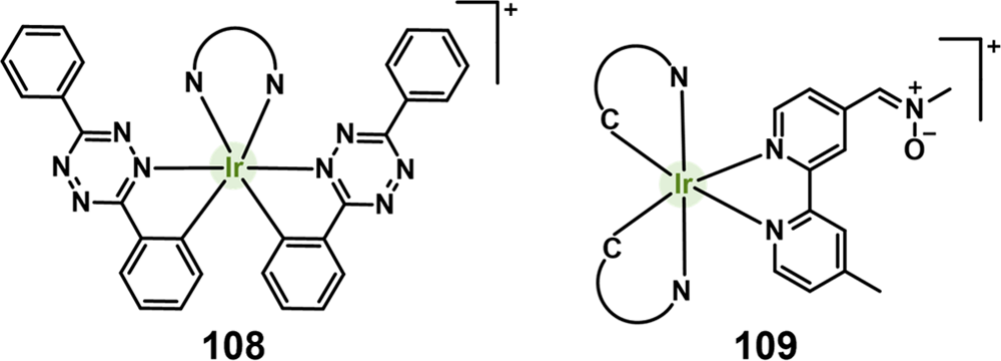
Chemical structures of theranostic probes **108** and **109**.

Instead of using organic dyes as fluorogenic probes and PSs, Lo et al. developed an interesting class of bis-tetrazine functionalized iridium(III) complexes (**108**, Figure 56) for biomedical applications, including luminogenic bioimaging and activatable PDT.^373^ Importantly, the tetrazine groups not only show remarkable bio-orthogonal reactivity but also efficiently suppress the luminescence properties of iridium(III) complexes (Φ_em_ < 0.09%). Upon reaction with strained alkynes, these complexes exhibited dramatically enhanced emission intensities (up to 2305-fold enhancement) and prolonged emission lifetimes (τ = ∼1.39 μs). Surprisingly, these complexes can also react with overdosed bis-alkynes to afford even larger enhancements in emission (up to 3885-fold brighter than the unreacted iridium(III) tetrazine complexes) and longer excited state lifetimes, which is attributable to the generation of cyclized iridium(III) complexes, as suggested by ESI-MS and RP-HPLC results. The enhanced rigidity of cyclized reaction products might account for the resulting higher emission and longer emission lifetimes, suppressing nonradiative decay and facilitating the emission process. Co-staining studies indicated that these complexes can be efficiently switched on by strained alkyne derivatives to produce an intense lysosomal emission. Remarkably, before activation, all the complexes displayed very low phototoxicity indices, indicative of minimal photocytotoxicity, while after reaction with strained alkynes to restore the photosensitizing ability (Φ_Δ_ = 0.62 to 0.77), low IC_50_ values (0.45 to 1.6 μM) with enhanced phototoxicity indexes were observed. Other than iridium(III) tetrazine derivatives, Lo’s group also developed an iridium(III) nitrone complex (**109**, Figure 56) that can similarly interact with an alkyne-tagged protein to activate the photosensitizing process.^374^

**Figure 57:**
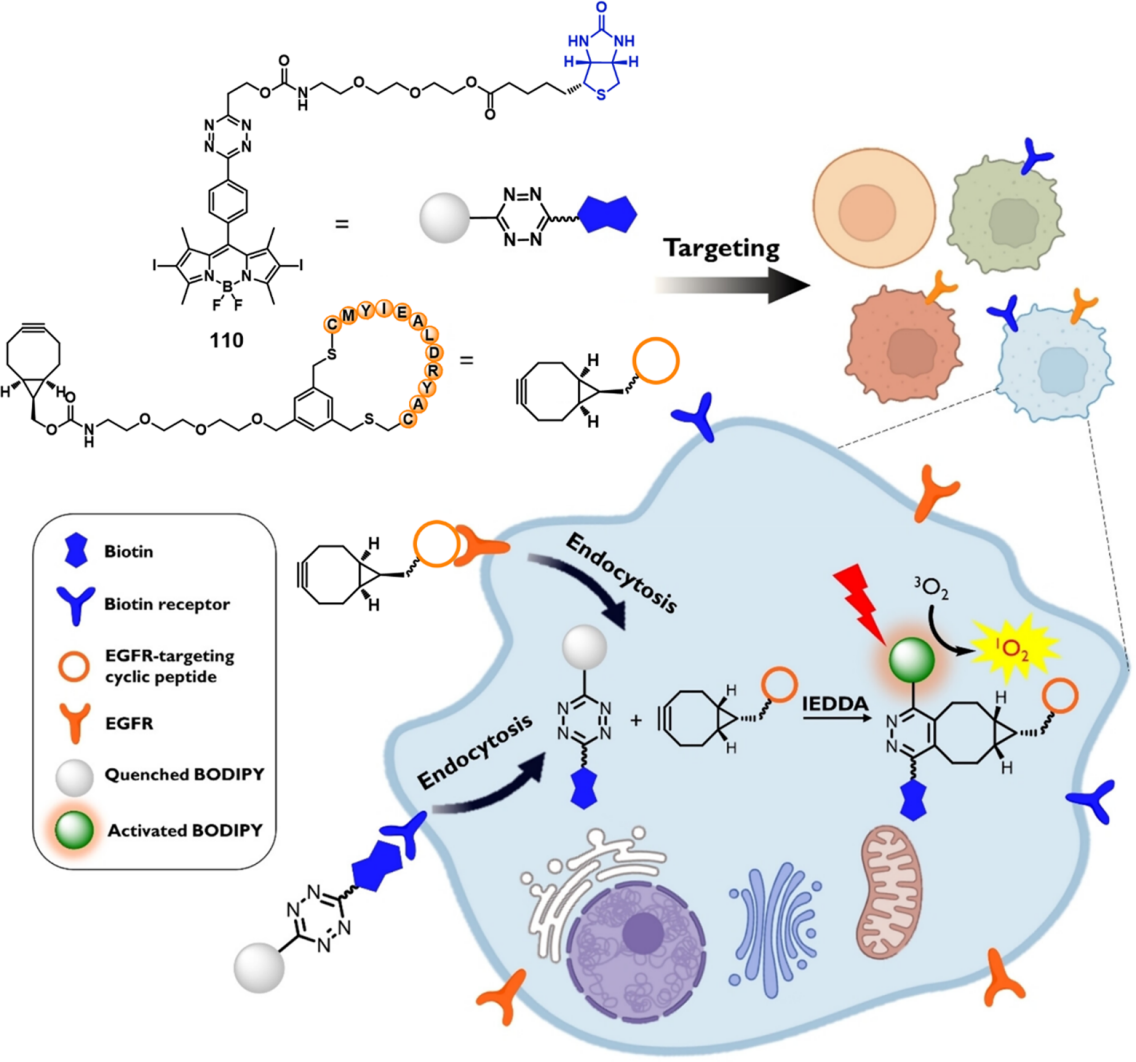
Schematic illustration of the dual receptor-mediated endocytosis of two bio-orthogonal partners (**110** and its reaction partner) into the target cells, followed by bio-orthogonal activation via the IEDDA coupling. Reproduced with permission from ref (375). Copyright 2022 Wiley Intersciences.

Most clinically used PDT agents lack sufficient cancer-targeting specificity, which inevitably causes undesired photodamage to surrounding normal tissues.^375^ To improve tumor-localizing properties, PDT agents are incorporated with various cancer-targeting biomacromolecules like antibodies, peptides, aptamers, and/or small molecules that can bind specifically to overexpressed biomacromolecules in cancers.^375^ However, unwanted uptake of these PS-ligand hybrids by healthy cells remains unavoidable. To achieve precise targeting, Ng et al. reported a unique dual receptor-involved bio-orthogonal activation strategy to optimize the cancer-targeting specificity of PDT agents. Initially, a biotinylated tetrazine-bearing BODIPY-originated pro-PS (**110**, Figure 57) and an epidermal growth factor receptor (EGFR)-targeting strained alkyne were delivered into living cancer cells that overproduced both the biotin receptors and EGFR-receptors and the two complementary bio-orthogonal units are accumulated in dual-receptor-overexpressing cells.^375^ Once these two parts encountered each other, rapid bio-orthogonal reactions occurred simultaneously, resulting in the recovery of the photosensitizing ability. Photocytotoxicity studies against a series of different cells demonstrated that **110** only showed drastic photocytotoxic effects on A549 cancer cells that overexpress both biotin and EGFR receptors and exhibited negligibly cytotoxic effects to other cells that did not express any receptor or express only one receptor, suggesting that this dual-receptor-assisted bio-orthogonal activation approach hold promise for precise PDT in specific cancers.

**Figure 58:**
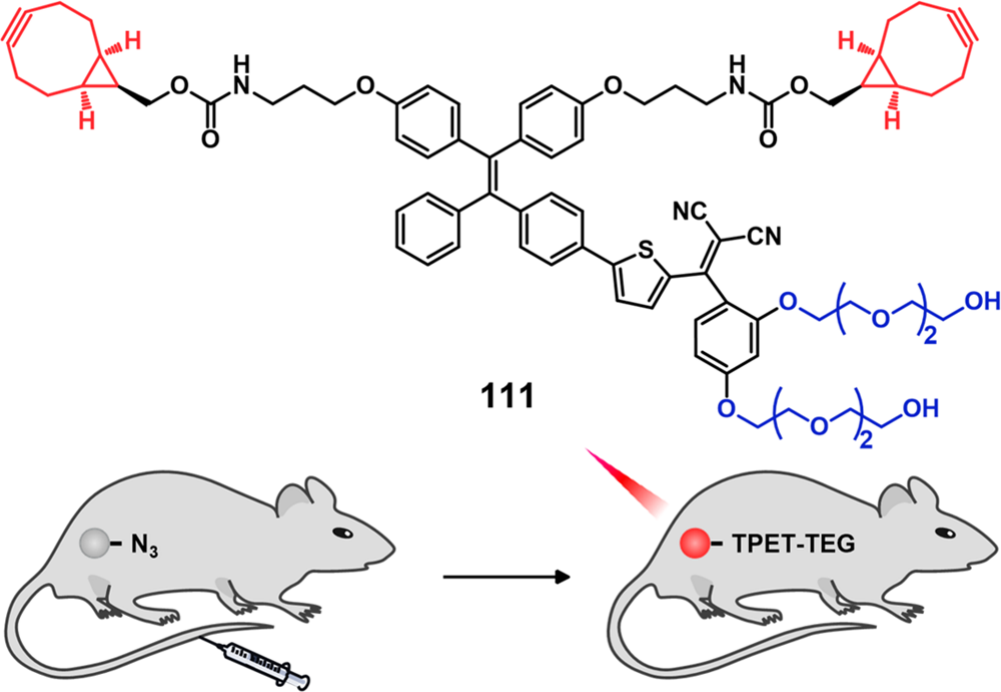
Chemical structure of theranostic probe **111**.

**Figure 59:**
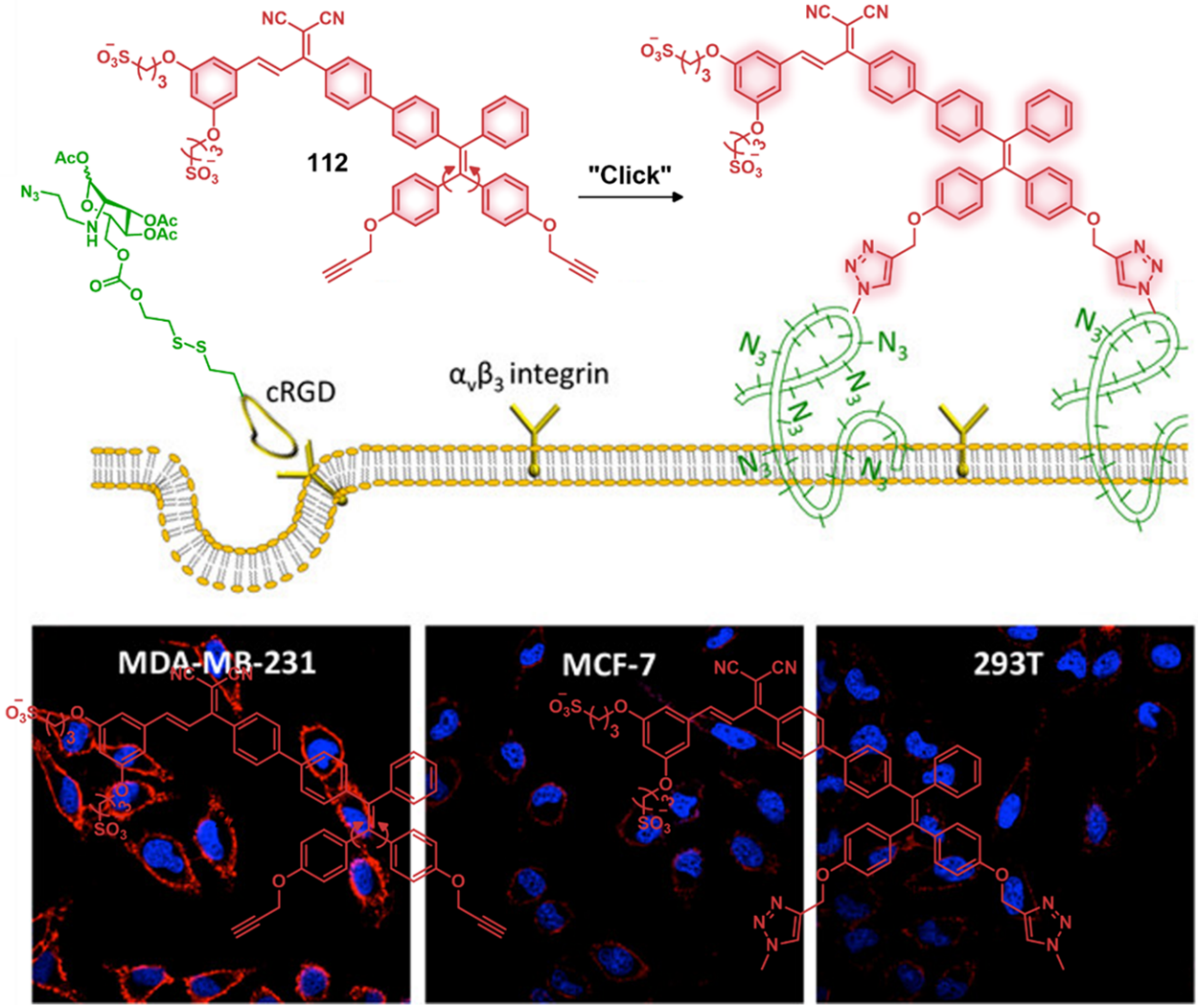
Chemical structure of theranostic probe **112** and confocal images of **112** (10 μM) labeled tumor cells (MDA-MB-231 and MCF-7) and normal 293T cells pretreated with cRGD-S-Ac_3_ManNAz for 2 h, followed by washing with 1× PBS and incubation in fresh DMEM medium for 2 days. Nucleus is stained with Hoechst 33342, λ_ex_ = 405 nm, λ_em_ = 430–470 nm; **112**, λ_ex_ = 405 nm, λ_em_ = >650 nm. Reproduced with permission from ref (377). Copyright 2018 American Chemistry Society.

Many bio-orthogonal-activable tumor-targeting methods are considered more effective in theranostic delivery than other targeting modalities since covalent conjugation results in tighter bonds to the tumor site and thus avoids clearance. Liu et al. presented the first bis-cycloalkyne-decorated AIEgen-based “Turn-On” probe (**111**, Figure 58) for bio-orthogonal tumor labeling and imaging-guided tumor PDT.^376^ Probe **111** exhibited minimal background emission in dispersed states but displayed intense fluorescence emission when cross-linked with 1,3,5-tris(azidomethyl)benzene. Notably, the burst emission was minimally affected by various surfactants and biomolecules, indicative of negligible nonspecific interactions with potential interference substances. Cellular studies manifested the applicability of probe **111** for targeted imaging of malignant 4T1 cells that were pretreated with AzAcSA, a metabolic precursor for overexpressed sialylated glycans in tumors. After cross-linking with azidoglycans on the cell membrane, the fluorescence of probe **111** was turned on rapidly, which not only allowed exclusive membrane labeling but also motivated the photosensitizing process, achieving a dose-dependent photocytotoxicity with an IC_50_ value of 10.4 μg mL^–1^. To further improve the cancer-targeting ability, Liu extended the bio-orthogonal-activation of phototheranostics by using another anionic AIEgen **112** (Figure 59) and a new precusor-cRGD-S-Ac_3_ManNAz,^377^ which is specifically bound by overexpressed α_*v*_β_3_ integrin and internalized selectively in tumor cells. Moreover, before cleavage of the disulfide bond by intratumoral GSH, cRGD-S-Ac_3_ManNAz was unable to be metabolized on cell membranes, which significantly enhanced the tumor-targeting specificity due to elevated GSH levels in tumor cells. Cellular MTT studies revealed that when the cancerous MDA-MB-231 cells and normal 293T cells were preincubated with Ac_3_ManNAz with no targeting ability, nondistinguishable photocytotoxicity toward these two cell lines was observed upon bio-orthogonal activation with **112**, while when cRGD-S-Ac_3_ManNAz was applied, a significantly higher photocytotoxicity against cancerous MDA-MB-231 cells (IC_50_ = 9.5 μM) resulted, compared to the IC_50_ values for the normal cell lines (IC_50_ > 9.5 μM), which confirmed the cancer-targeting efficiency. To summarize, specific bio-orthogonal activation in combination with fluorogenic AIEgen and cancer-targeting metabolic precursor provides additional niches for facile accessing of innovative phototheranostics.

**Figure 60:**
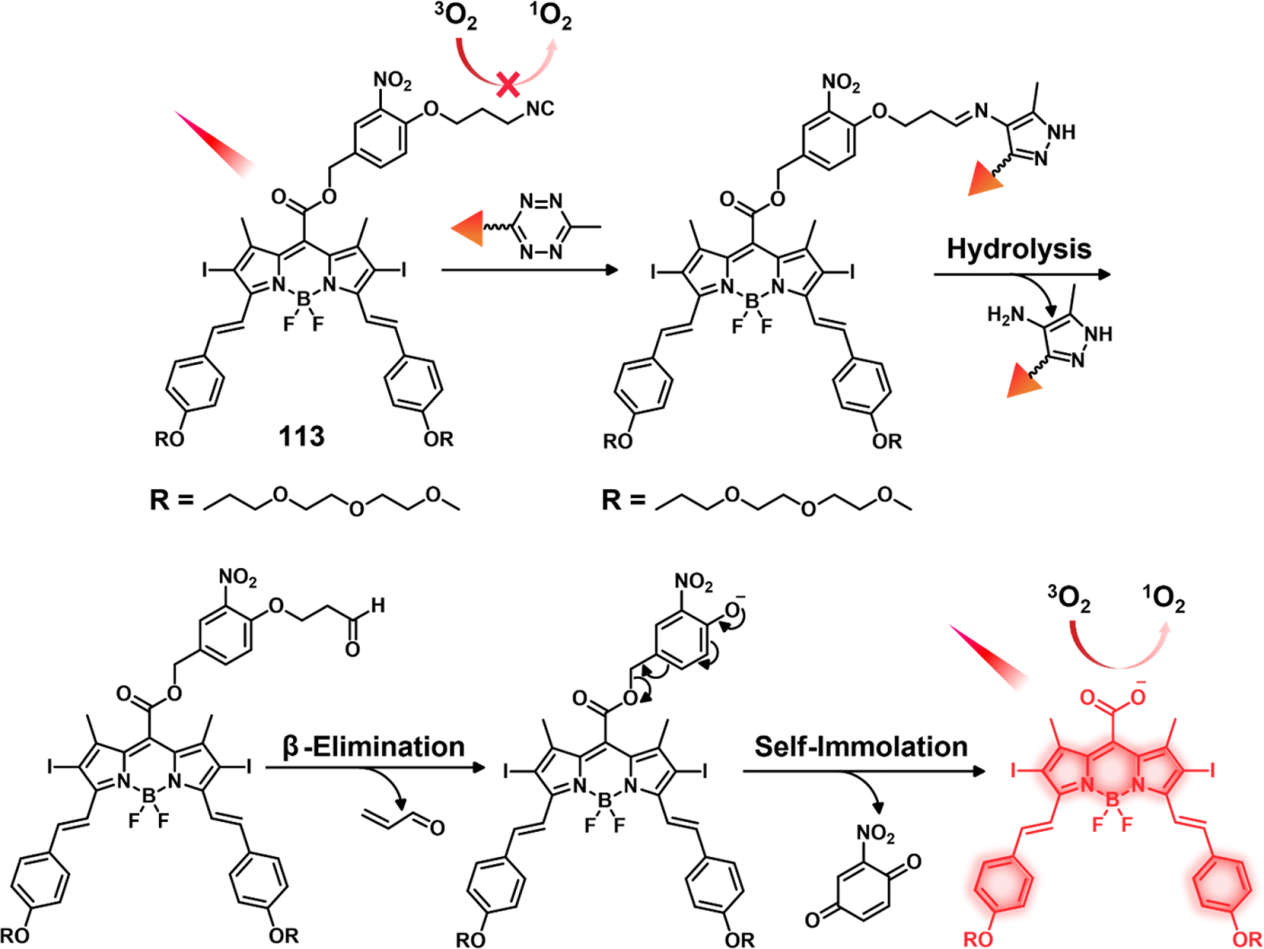
Chemical structure of **113** and its bio-orthogonal activation reaction. Reproduced with permission from ref (379). Copyright 2023 Elsevier Ltd.

Apart from bio-orthogonal ligation, the recently developed bio-orthogonal cleavage reaction has also received increasing attention.^378^ By applying a cleavable nitrobenzyl isonitrile-attached BODIPY PS (**113**, Figure 60), Ng and Lo et al. employed a dissociative bio-orthogonal reaction for tumor-targeted activation of PSs for specific anticancer PDT applications.^379^ The ^1^O_2_ generation of **113** is not suppressed by the isonitrile moiety but by the nitrobenzyl quenching unit. This strategy allowed controllable activation of the PSs via isonitrile-tetrazine cycloaddition-induced removal of the nitrobenzyl quenching group. After functionalization of tetrazines with a galactose moiety or the GE11 peptide, which targets overexpressed asialoglycoprotein receptors or EGFR receptors in cancerous cells, the specific reaction between **113** and functionalized tetrazines in live cancer cells resulted in drastic fluorescence enhancement and efficient ^1^O_2_ generation. Additionally, bio-orthogonal cleavage-activated photocytotoxic effects have also been realized in vivo with satisfactory results.

**Figure 61:**
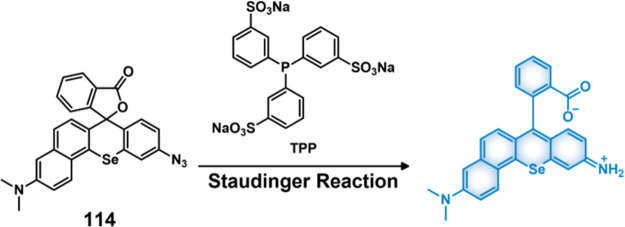
Activation of **114** by TPP.

Finally, the Staudinger reaction, converting azides to primary amines through the formation of an iminophosphorane intermediate, has drawn a lot of attention for biomedical applications owing to its rapidness, biocompatibility, and high reaction yield.^380^ Considering these merits, Liu prepared a π-extended long wavelength absorbing azide-decorated Se-rhodamine-derived pro-PS (**114**, Figure 61), which could be bio-orthogonally activated by a Staudinger reaction using triphenylphosphine.^381^ Interestingly, the azido-rhodamine resulted in the closure of the spiro-ring, with which the NIR emission of Se-rhodamine was completely quenched and the absorption maximum displayed a hypsochromic shift from 616 to 405 nm. Additionally, pro-PS **114** exhibited very limited photocytotoxicity (IC_50_ = 15.9 μM) before triphenylphosphine activation to HeLa cells but displayed a remarkable photocytotoxic effect (IC_50_ = 0.218 μM) to the same cell line after activation assisted by the Staudinger reaction.

#### 2.2.4 Enzyme-Activatable Theranostic Fluorescent Probes in PDT

Enzymes are critical to many metabolic processes, and elevated enzymatic activities are believed to facilitate several pathological processes, ranging from tumor angiogenesis and cell invasion to metastasis. So far, researchers have detected many overexpressed enzymes in various tumor types, and with the help of biologists, the biological substrates of these enzymes have been disclosed, which can be employed to construct “smart” phototherapeutic agents, whose photoactivity could be switched on by a specific enzyme, thus allowing the distinction of healthy cells from diseased ones and abolishing off-target damage to the skin and neighboring healthy cells.^304,305,382^

Compared to other tumor markers, such as acidic pH, redox conditions, and elevated ROS levels, most enzyme-catalyzed substrate reactions are fast, mild, and especially highly specific, endowing a high sensitivity and low false positivity rate to enzyme-activated PSs. Therefore, using cancer-associated enzymes to fabricate activable PSs has attracted great attention. In this section, we will discuss several typical examples of enzyme-activatable “smart” PSs, including those activated by nitroreductase (NTR), azoreductase, cathepsin B, β-galactosidase, alkaline phosphatases, tyrosinase, γ-glutamyl transpeptidase, and aminopeptidase.

##### 2.2.4.1 Nitroreductase-Activatable Theranostic Probes in PDT

Nitroreductase is a well-known specific enzyme overexpressed in hypoxic solid tumors due to the reductive stress of low intracellular O_2_ concentrations, which can effectively reduce nitroaromatics to the corresponding arylamines in the presence of reduced nicotinamide adenine dinucleotide (NADH) as an electron donor. Based on this reduction reaction, many hypoxia-triggered theranostic probes have been designed by installing nitroaromatic groups into dye scaffolds.^383−385^

##### 2.2.4.1.1 *p*-Nitrobenzoate Group

**Figure 62:**
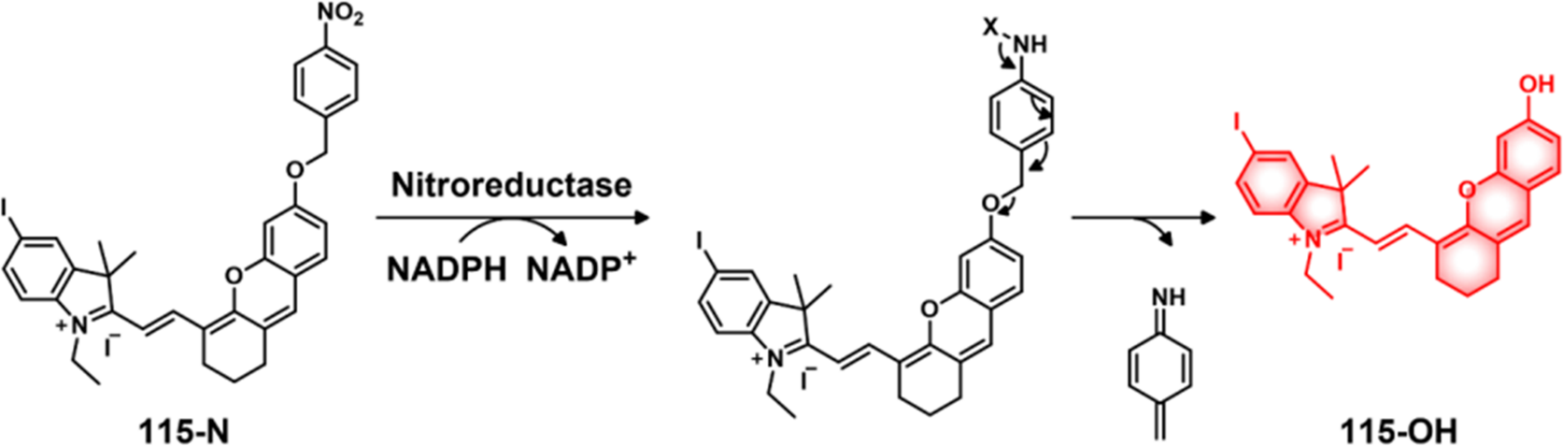
Recognition mechanism of **115**-N with NTR.

**Figure 63:**
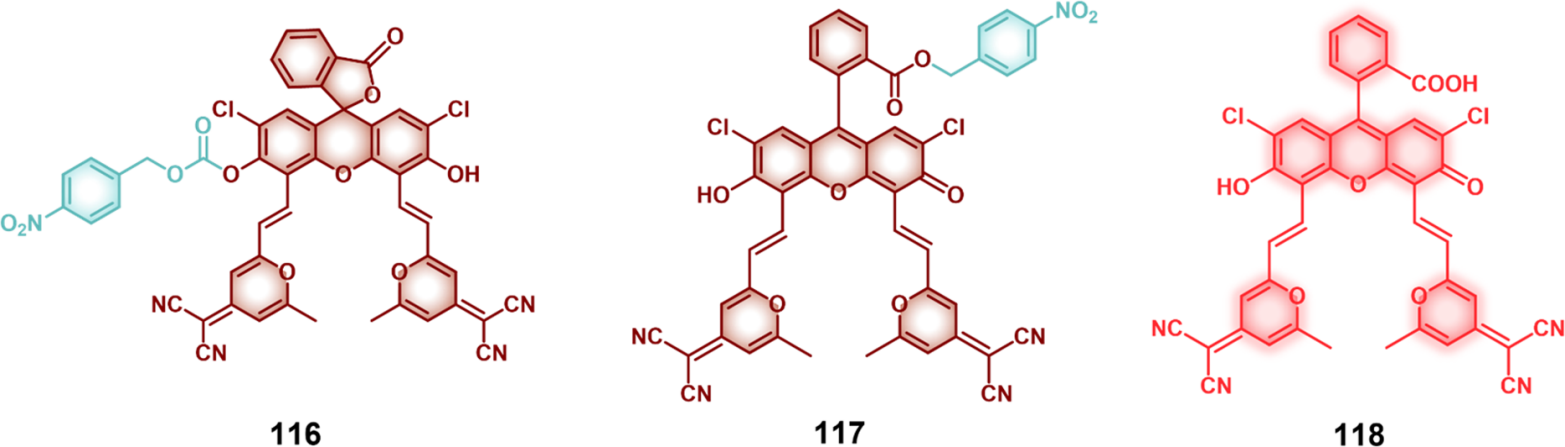
Molecular structures of **116**–**118**.

Owing to the strong hydrogen-bonding interaction and suitable spatial match with NTR, the *p*-nitrobenzoate group has been recognized as an ideal NTR substrate with a fast response, high sensitivity, and good selectivity. By introducing this functional group into different PS scaffolds, Peng’s group developed several NTR-activated phototheranostic agents mostly based on PET and ICT mechanisms. For instance, Peng’s group reported a hypoxia-activated D-π-A PS (**115**-N) by decorating the phenol hydroxyl of an iodine-substituted hemicyanine dye (NIR PS) with 4-nitrobenzyl bromide.^386^ As shown in Figure 62, the introduction of *p*-nitrobenzoate restrained the ICT process, leading to weak fluorescence and low ^1^O_2_ production. While in hypoxic cancer cells and tumor tissues, **115**-N was reduced to **115**-OH by intracellular overexpressed NTR. The latter exhibited a markedly increased photosensitivity due to the restoration of ICT after the removal of the caging group. In normal tissues, **115**-N was in a fluorescence-off state and exhibited almost no phototoxicity, while hypoxia-induced cell apoptosis and suppression of tumor growth under 660 nm light irradiation, highlighting the PS’s potential for selective tumor hypoxia imaging and PDT. Two additional NTR-activatable theranostic molecules (**116** and **117**) based on TADF fluorescein derivatives were designed to enable PDT in mildly hypoxic tumors (Figure 63).^387^ When subjected to screening, **117** performed better than compound **116** in terms of selectivity and response rate to NTR. Importantly, endogenous NTR in tumor cells can catalyze the enzymatic cleavage reaction of **117** to form **118**, achieving a high PDT efficiency even under 10% oxygen concentrations.

##### 2.2.4.1.2 2-Nitroimidazole Group

**Figure 64:**
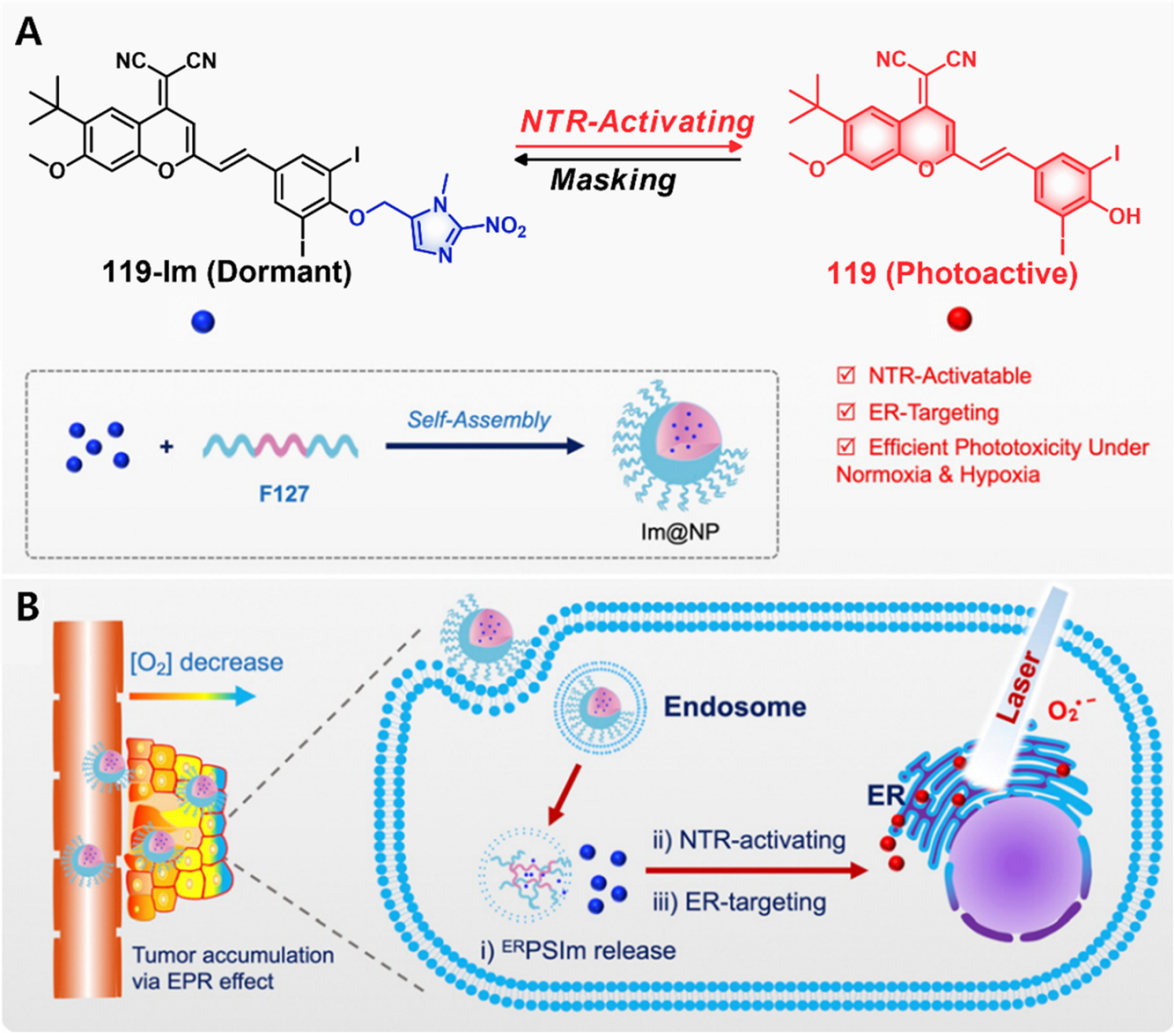
Schematic illustration of NTR-activatable PS **119-Im**. Figure reproduced with permission from ref (388). Copyright Royal Society of Chemistry.

Li and co-workers devised a novel NTR-activatable NIR PS (**119**), which features an intrinsic ER-targeting capability and low oxygen-depletion type I photosensitivity, endowing ^ER^PS with highly efficient phototoxicity against cancer cells under both normoxic and hypoxic conditions.^388^ As depicted in Figure 64, **119-Im** was constructed by caging its hydroxyl group with a 2-nitroimidazole-based triggering group as a recognition site for NTR. To improve the water stability and increase tumor enrichment, **119-Im** was further encapsulated within polymeric micelles to yield a spherical **119-Im-**NP nanoparticle with favorable colloidal stability. As expected, masking the phenolic hydroxyl group led to the quenching of fluorescence and photosensitizing activity of **119-Im** encapsulated in the **119-Im-**NP micelle, due to the suppression of the ICT process. After being taken up by cancer cells, the **119-Im** cargo was released from **119-Im**-NP, and then was specifically converted into active **119** by the overexpressed NTR in hypoxic cancer cells. The converted **119** was mainly localized in the ER with a Pearson’s correlation coefficient as high as 0.97, and 119 efficiently generated O_2_^–•^ and •OH upon irradiation, which induced severe ER dysfunction and protein misfolding. Overall, benefiting from hypoxia activatability, specific ER-targeting properties, and low oxygen depletion advantages of type I photosensitivity, **119-Im-**NP demonstrated a highly selective and efficient PDT-killing effect in hypoxic cells and inhibited solid tumor growth. This work provides a model PS for addressing both off-target effects and hypoxic resistance in tumor therapy.

##### 2.2.4.2 Azoreductase-Activatable Theranostic Probes in PDT

Azoreductase also is one of the typical hypoxia-responsive enzymes, which has been demonstrated to be overexpressed in hypoxic cells, particularly within solid tumors. Its capability to reduce azo groups and generate amino derivatives establishes it as an inherent biomarker for tumor hypoxia. Consequently, the azo moiety can be employed as a caging group to block the photosensitivity of PSs, thereby creating “switch on” PDT molecules specifically designed to fight hypoxic tumors.^389−391^

**Figure 65:**
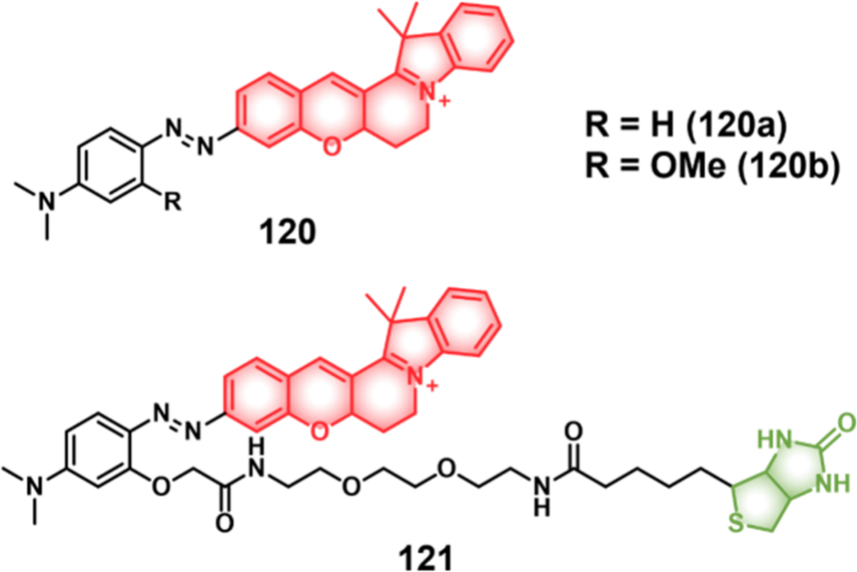
Structures of theranostic probes **120** and **121**.

Traditional PSs are faced with insufficient light penetration depth, hypoxic sensitivity, and poor tumor targeting, which seriously restricts the efficacy of PDT.^392,393^ To solve these problems at the same time, Kim et al. designed a hypoxia-responsive, two-photon excitable, type I PS that was further modified by a targeting group. Considering the deep penetration and greater spatial precision of two-photon excitation a compatible type I PS was chosen. To this end, they also developed a series of cyclized-cyanine derivatives (**120**), shown in Figure 65. These dyes demonstrated impressive fluorescence emission (Φ_F_ up to 60%) and excellent two-photon action cross sections (up to 103 GM).^394^ Notably, these heavy atom-free small-molecules displayed superior ROS-generating abilities compared with 5-ALA via a type I mechanism. Moreover, the two-photon excitation was demonstrated to be a more efficient approach than one-photon excitation. Azo groups were conjugated to the dye skeleton and two caged PSs (**120a** and **120b**) were obtained that can be activated by hypoxia. When taken up by cancer cells that overexpress azoreductase, **120** showed a rapid response and exhibited excellent cancer cell-killing abilities with negligible dark toxicity. More recently, these authors also linked the PS with a targeting unit (biotin) to realize a highly spatiotemporal-selective two-photon PDT agent for colon cancer (**121**, Figure 65).

**Figure 66:**
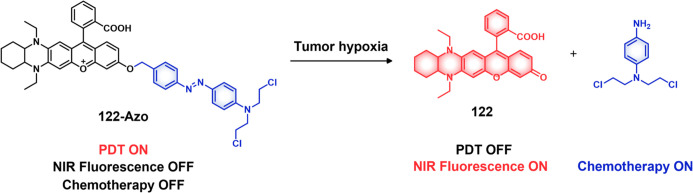
Design and chemical structure of the theranostic construct **122-Azo**.

Hypoxic tumor environments usually provide insufficient oxygen to support the operation of PDT, resulting in limited therapeutic efficacy, especially in the deep inner part of a solid tumor. To overcome this issue, Liu and Kim et al. designed a theranostic molecule (**122-Azo**) for hypoxia-responsive chemotherapy and phototherapy in solid tumors (Figure 66).^395^**122-Azo** was synthesized by linking a rhodol-based NIR fluorophore (**122**) with nitrogen mustard through an azo bond (−N=N−). Its fluorescence was fully inhibited by intramolecular π–π stacking and a blocked ICT process. Unlike the beforementioned activatable PSs, **122-Azo** displayed excellent ROS production (Φ_Δ_ = 0.14) while the active form does **122** not. Thus, the distinctive photosensitivity of **122-Azo**, with or without hypoxia stimuli, allowed it to perform via a different anticancer modality. On the surface layer of the tumor, **122-Azo** demonstrated an impressive PDT effect, eliminating normoxic cancer cells as the lower expression of azoreductase, presumably due to the fact that this enzyme is unable to cleave the azo bond. However, within the tumor, the reduction of the azo bond triggered the release of **122** and an active nitrogen mustard, enabling the effective killing of hypoxic tumor cells via chemotherapy. Additionally, this process facilitated in situ and real-time monitoring of dosage and kinetics during drug release. Overall, this strategy successfully eradicated cancer cells in both normoxic and hypoxic environments, maximizing therapeutic efficacy for solid tumors.

##### 2.2.4.3 Cathepsin B-Activatable Theranostic Probes in PDT

Cathepsin B (CTSB) is a cysteine protease present in lysosomes that is highly expressed in a variety of tumors, and when excreted it can degrade extracellular matrix components, such as collagen, laminin, or tenascins, thereby promoting tumor invasion and metastasis during cancer progression. Aberrant expression/activity of CTSB has been employed as a pathological biomarker in the design of theranostic agents for cancer-selective imaging and treatment.^305,396^

**Figure 67:**
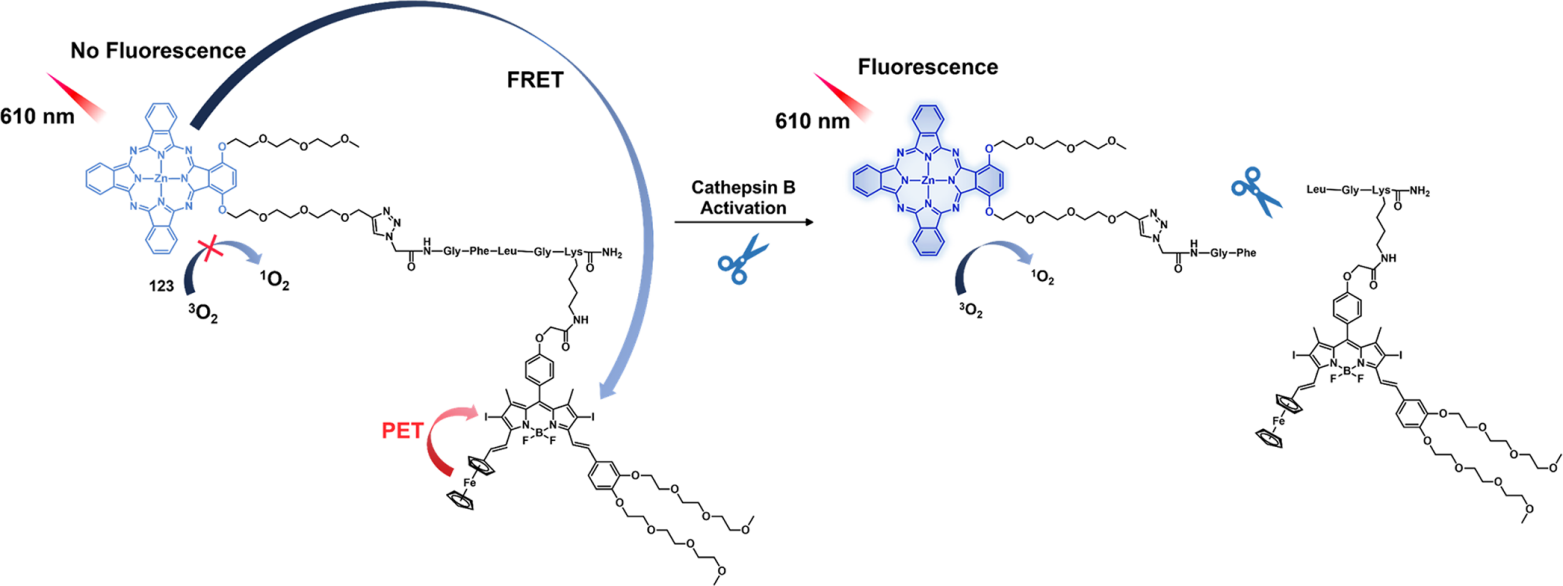
Schematic diagram showing the working principle of the cathepsin B-activatable PS **123** based on a FRET-PET process.

In this regard, Lo et al. devised a cathepsin B-responsive fluorescent probe and PS (**123**), composed of a zinc(II) phthalocyanine-based PS, a new ferrocenyl BODIPY dark quencher, and a cleavable substrate for cathepsin B (Gly-Phe-Leu-Gly-Lys).^397^ As depicted in Figure 67, in the absence of cathepsin B, conjugate **123** displayed weak NIR fluorescence and a low ^1^O_2_ generation ability because of FRET between the zinc(II) phthalocyanine and the BODIPY unit, followed by a PET process from the ferrocenyl moiety. Upon the addition of cathepsin B, the peptide substrate was cleaved, separating the phthalocyanine and ferrocenyl BODIPY units, thus preventing the FRET-PET effect and restoring the photosensitizing properties of the zinc(II) phthalocyanine. Under light irradiation at 610 nm, **123** exhibited a high photocytotoxicity in HepG2 cells (cathepsin B-positive cells) with IC_50_ of 0.32 μM, highlighting **123** as a highly efficient cathepsin B-activatable PS.

**Figure 68:**
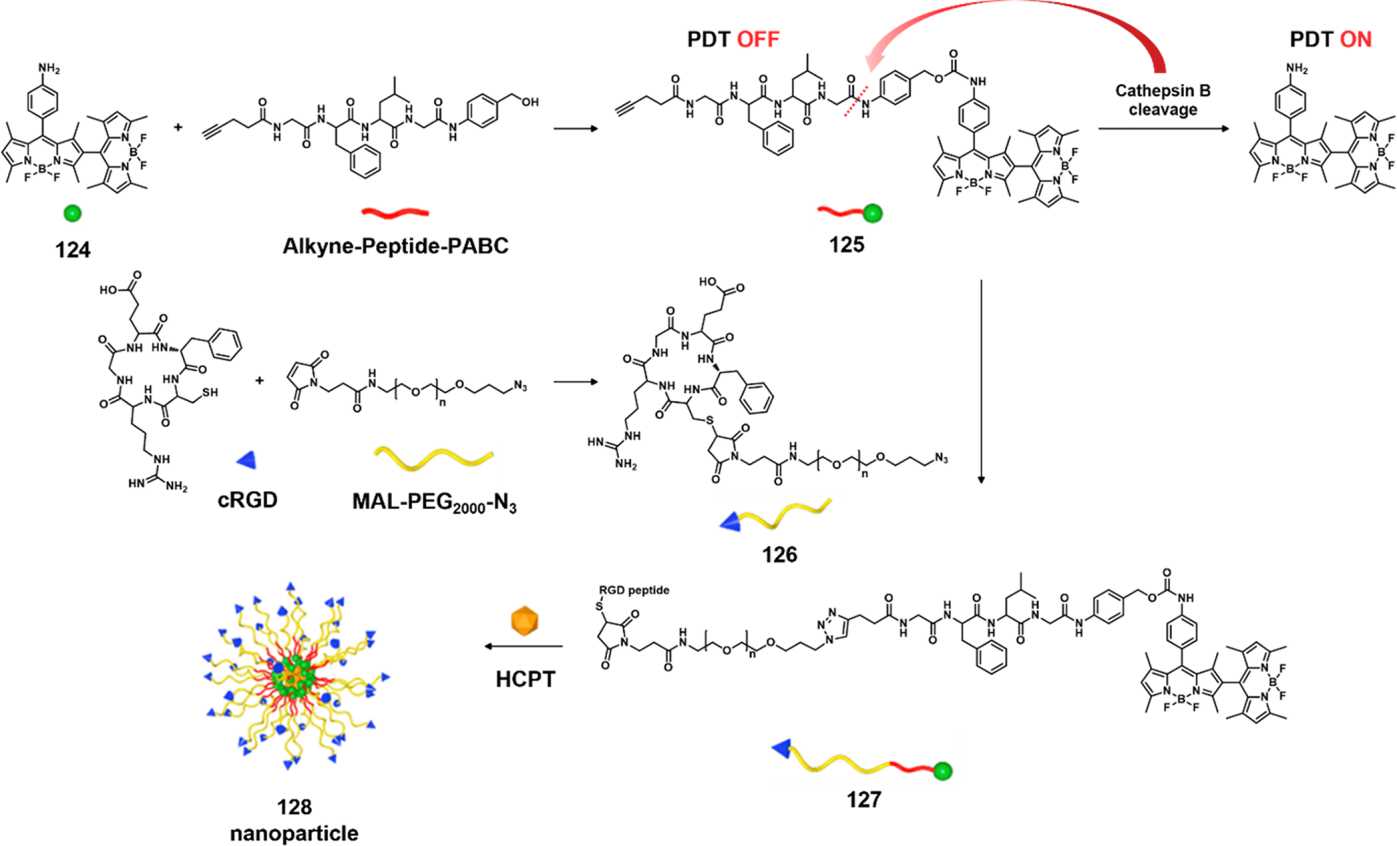
Schematic illustrations for the synthesis of **125**, **126**, and **127**, the formation of **128** nanoparticles, and the mechanism of the activatable PS.

In a different approach, Chen et al. developed a novel cathepsin B activated BODIPY-based PS, **125**, whose activation mechanisms of photosensitivity rely on the presence of an intramolecular charge transfer (ICT) effect.^398^ In **125**, an orthogonal BODIPY dimer-based PS, **124**, was conjugated with an alkynyl group-modified cathepsin B-cleavable peptide (alkyne-Gly-Phe-Leu-Gly) through a *p*-aminobenzyloxycarbonyl bridge (Figure 68). The cathepsin B-cleavable peptide substrate inhibited the electron-donating ability of the amino group of **124**, leading to a blocked ICT process and significant suppression of ^1^O_2_ generation. To improve the tumor-targeting ability and antitumor efficacy, **125** was then linked with a cRGD-modified PEG chain (**126**) to obtain **127**, which is used as a nanocarrier to further encapsulate and deliver 10-hydroxycamptothecin (HCPT), a hydrophobic anticancer agent. Benefiting from a tumor cell-targeting peptide (cRGD), the resulting **128** nanoparticles not only exhibit specific cellular uptake in integrin α_v_β_3_-positive 4T1 cells as compared to HeLa cells with a low α_v_β_3_ expression but also promoted tumor penetration in the 4T1 three-dimensional (3D) tumor spheroids models. Notably, after the cleavage of the peptide by intracellular cathepsin B, **128** activated the PDT activity of **124**, as the amino group is uncaged, while releasing HCPT, therefore effectively inducing apoptosis of 4T1 cells and shrinking the size of the 4T1 3D tumor spheroids through the combined effects of PDT and chemotherapy. This work reported a novel strategy for constructing cathepsin B-activated PSs and building a promising antitumor platform.

##### 2.2.4.4 β-Galactosidase-Activatable Theranostic Fluorescent Probes in PDT

β-Galactosidase (β-gal) is an essential glycoside hydrolase located within the lysosomes of cells, which performs a critical function by facilitating the breakdown of glycoside bonds and promoting the conversion of lactose into galactose.^399,400^ Despite being overexpressed in primary ovarian cancer and gliomas, β-gal is not commonly considered to be a biomarker for cancer. However, recent studies have unveiled its significance as one of the most important hallmarks of senescence, and numerous investigations have focused on developing methods to selectively eliminate senescent cells.

**Figure 69:**
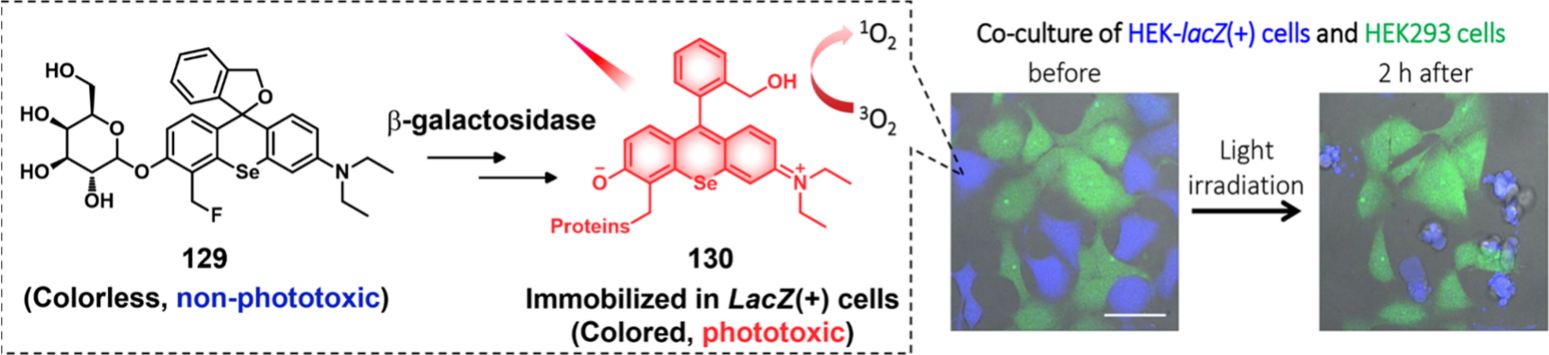
Activatable PS (**129**) targeted to β-galactosidase and time-lapse fluorescence imaging of a coculture of HEK293 and HEK-lacZ(+) cells. HEK-lacZ(+) cells and HEK293 cells were prestained with CellTracker Blue and CellTracker Green, respectively. Reproduced with permission from ref (401). Copyright 2019 American Chemical Society.

For example, Urano et al. designed an activatable PS **129** to specifically target and eliminate lacZ-positive cells (Figure 69).^401^ The PS was developed using a Se-substituted Rhodamine compound, where a fluoromethyl group served as an electrophilic site at the 4-position. The p*K*_cycl_ value of **129** was determined to be 5.4, indicating that, at the physiological pH of 7.4, the compound would predominantly exist in a cyclized, colorless, and nonphototoxic form. By contrast, the reference compound, **130**, exhibited a p*K*_cycl_ of 10.3 and a p*K*_a_ of 4.8, suggesting that the reference compound would predominantly exist in its open and phototoxic form (Φ_Δ_ = 0.36) at pH 7.4. The hydrolysis of **129** resulted in the activation of both its photosensitizing ability and reactivity to nucleophiles due to the formation of a quinone methide intermediate. As a result, the PS could be trapped inside lacZ-positive cells. Moreover, this mechanism enabled selective killing of β-galactosidase-expressing cells with single-cell resolution. The effectiveness of **129** was confirmed in Drosophila models, demonstrating its promising in vivo applicability.

**Figure 70:**
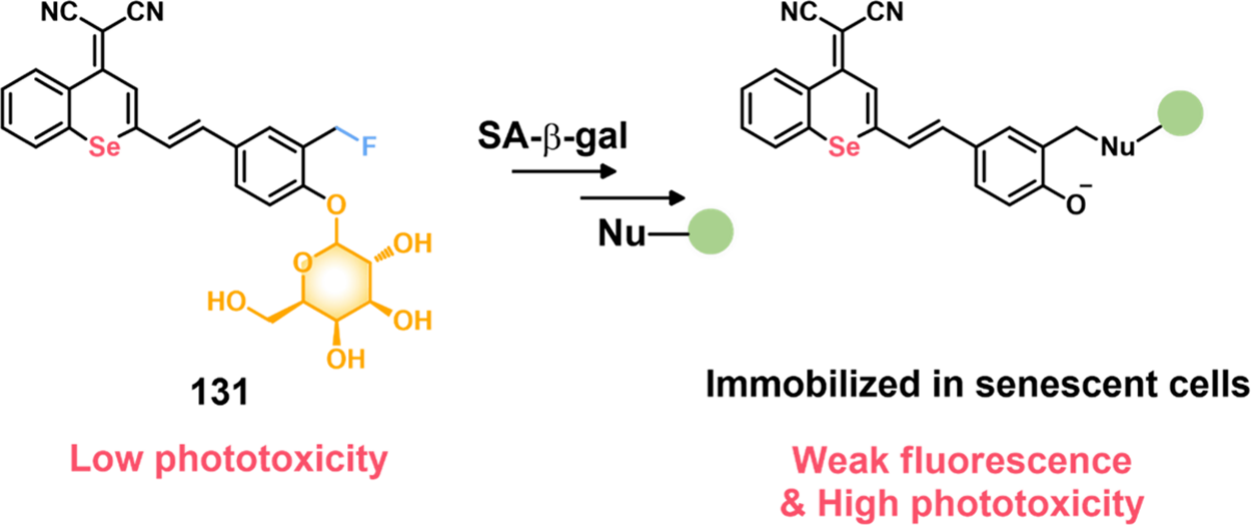
Integrated strategy used to design the senotherapeutic probe **131**.

Based on the same responsive group, Li et al. developed a photosensitive senolytic prodrug (**131**) that could be specifically activated in the presence of SA-β-gal (Figure 70).^402^ To enhance intersystem crossing, ensuring efficient phototherapy efficacy, the authors replaced the oxygen atom in the dicyanomethylene-4H-pyran-based skeleton with selenium. The presence of *E. coli* β-gal and BSA led to an increased Φ_Δ_ value of **131** from 0.07 to 0.20, while the relative fluorescence quantum yields decreased from 0.33 to 0.08. This modification allowed **131** to exhibit high efficacy and broad-spectrum activity against senescent cells while minimizing side effects on nonirradiated areas. In both a doxorubicin-induced senescence mouse model and naturally aged mice, **131**-mediated PDT demonstrated the selective elimination of senescent cells in tissues. This research provides a new perspective on monitoring and selectively removing senescent cells to regulate the aging process.

##### 2.2.4.5 Alkaline Phosphatase-Activatable Theranostic Probes in PDT

Alkaline phosphatase (ALP) is a type of extracellular plasma membrane-anchored hydrolase that is involved in the dephosphorylation processes during diverse cellular events, including regulation of protein phosphorylation, cell growth, apoptosis, and migration. Elevated activity of ALP is closely related to a range of diseases, such as breast cancer, prostate cancer, kidney tumors, and osteosarcoma; thus, it has been considered a significant biomarker used in clinical diagnosis.^403,404^ Large numbers of fluorescent probes have been developed for the selective fluorescent visualization of ALP-positive cancer cells, however, reports of activable PSs selectively responsive to ALP are still somewhat limited.

**Figure 71:**
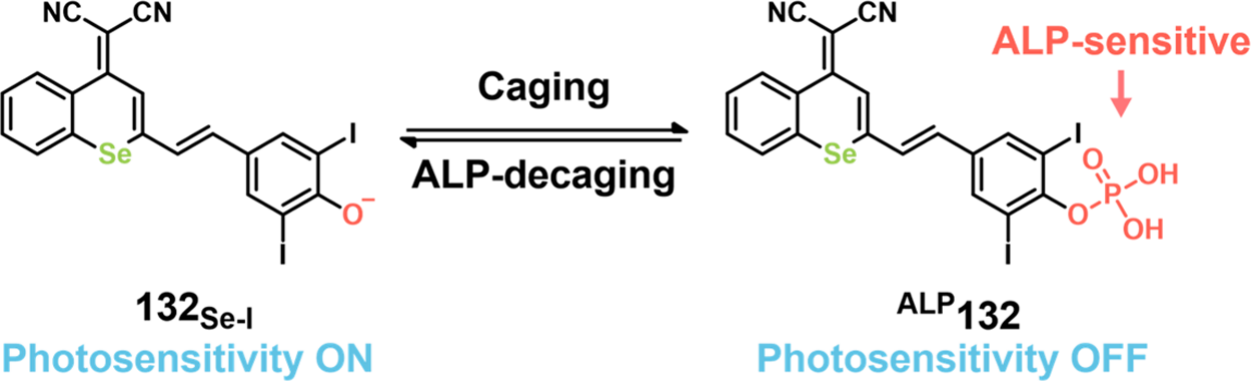
Schematic illustration of the design of an ALP-activatable PS (^**ALP**^**132**).

Li and co-workers developed a new type of D-π-A PS scaffold (denoted as **132Se-I**), by using a selenium-substituted dicyanomethylene-4H-chromene as the electron acceptor and 2,6-diiodo phenolate as the electron donor (Figure 71).^360^ In **132Se-I**, the introduction of heavy Se atoms contributed to a remarkable redshift of wavelength and significantly amplified the photosensitized ^1^O_2_ production, favoring highly efficient PDT in deep tumor tissues. Besides, the two ortho-iodo groups further enhanced the photosensitivity, and more importantly reduced the p*K*_a_ of the phenolic group, enabling PDT to a typical physiological pH of 7.4. Moreover, by conjugating phosphate (the protecting group and ALP recognition site) to the phenolic hydroxy group of **132Se-I**, a representative ALP-activatable PS was prepared (**^ALP^132**), whose photosensitivity was completely turned off, due to the suppression of an ICT process induced by the diminished electron-donating ability of the phenolate group in **132Se-I** after phosphorylation. Upon the addition of ALP or when incubated with living Hela cells overproducing ALP, **^ALP^132** was quickly converted into **132Se-I**, leading to full recovery of the quenched fluorescence and photosensitivity. Live/dead staining images and an MTT assay demonstrated that **^ALP^132** significantly decreased HeLa cell viability. Overall, this work demonstrated an effective strategy for designing ALP-triggered off/on switching of photosensitivity via the caging and uncaging of a phenol.

**Figure 72:**
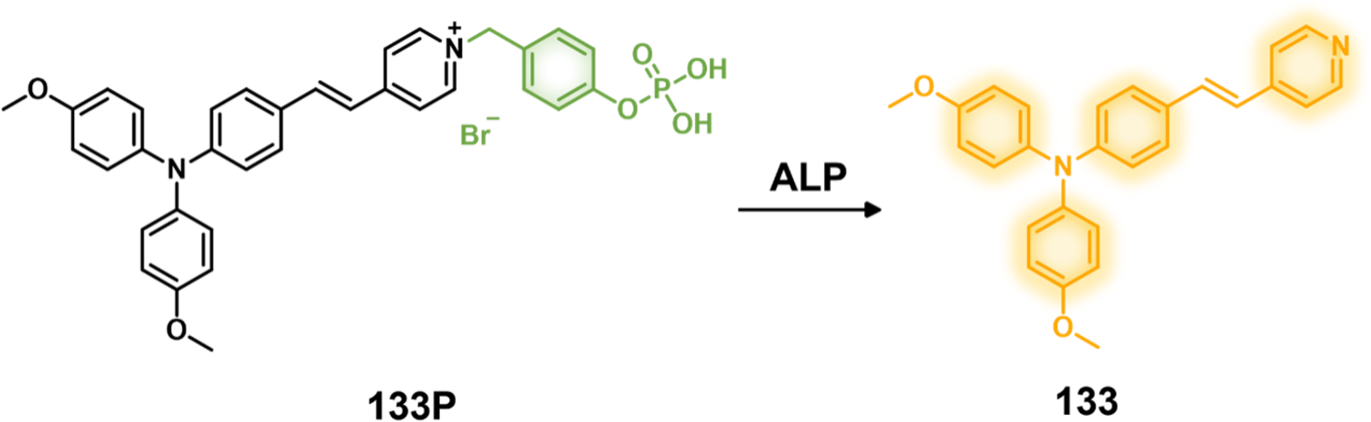
Schematic illustration of the hydrolysis of **133P** by intracellular ALP in cancer cells.

Conventional luminophores feature planar structures and usually suffer from severe aggregation-caused quenching (ACQ) through strong intermolecular π–π donor-acceptor interactions, which limits the concentration that can be applied to biological systems. Recently, aggregation-induced emission luminogens (AIEgens) have emerged as an alternative class of fluorescent materials, which have shown excellent performances in effective ROS generation as well as high fluorescence brightness in the aggregated state, while being highly resistant to photobleaching. Thus, these fluorophores offer unique advantages, such as improved imaging signal-to-noise ratios and phototherapy efficacies.^405^ So far, AIEgen-based-activatable fluorescent probes have been widely investigated for many biomedical applications.^313,314^ Very recently, Tang and He et al. developed an ALP-activated AIE PS, **133P**, which employs a triphenylamine derivative as the PS core and a phosphate group as an enzyme-labile moiety, respectively (Figure 72), to specifically target and kill the ALP-overexpressed cancer cells.^406^ Probe **133P** was nonemissive in aqueous solutions, while upon the addition of ALP its phosphate group can be efficiently hydrolyzed to obtain **133**, which further formed aggregates and emitted a yellow-colored fluorescence (λ_em_ = 540 nm). The specific fluorescent response to the presence of the ALP enzyme enabled **133P** to discriminate cancer cells from normal cells and to quantify the ALP level in the cells, as a function of the fluorescence intensity. Notably, under light irradiation, neither **133** nor **133P** could produce ^1^O_2_, but both can induce hydroxyl and superoxide radical generation. The ROS production efficiency of **133** is slightly greater than **133P** but higher than Ce6 (a commercially available PS). Given the difference in ALP expression and ALP-specific activation characteristics, the total ROS generation efficiency of **133P** aggregates in cancer cells is much lower than those **133P** in normal cells, therefore resulting in the selective killing of cancer cells. The present work provided a novel strategy for designing cancer-specific biomarker-responsive AIE PSs.

##### 2.2.4.6 Tyrosinase-Activatable Theranostic Probes in PDT

Melanoma is an aggressive malignancy with rapid growth and early metastasis. Tyrosinase (TYR), a copper-containing oxidase involved in melanin synthesis, which can convert monophenol or catechol to the corresponding o-quinone, is the rate-limiting enzyme for the biosynthesis of melanin from tyrosine. TYR is markedly overexpressed in melanoma cancer cells, thus making it an important biomarker for the diagnosis and treatment of melanoma.^407^

**Figure 73:**
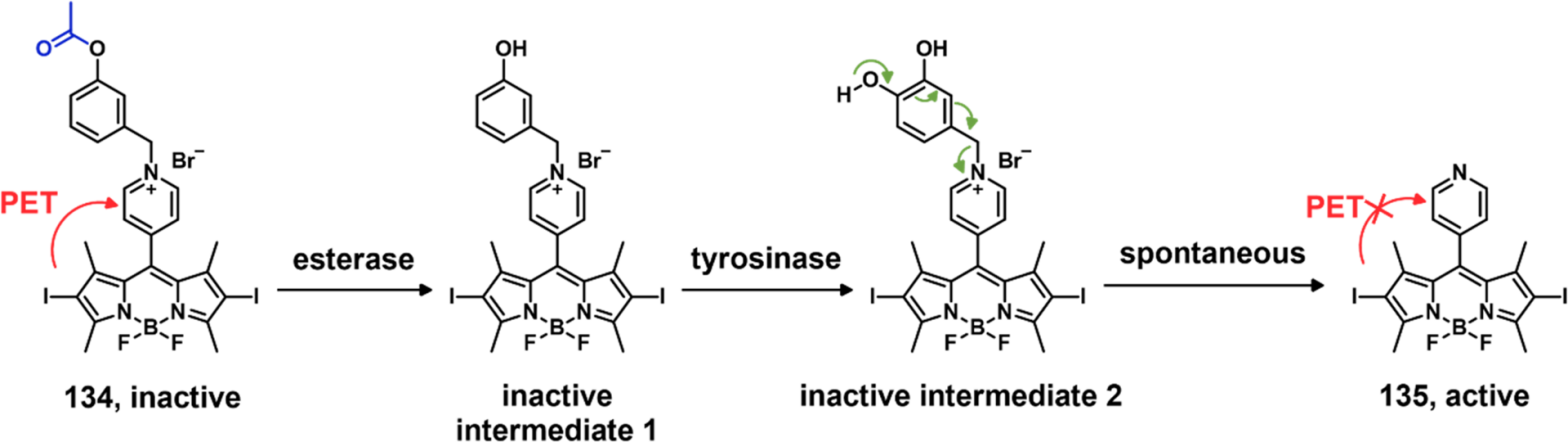
Structure of the enzyme-responsive PS **134** and the proposed mechanism of action. Inactive **134** is converted into active **135** through reactions catalyzed by esterase and tyrosinase.

Sundus Erbas-Cakmak and co-workers developed the first tyrosinase-activatable BODIPY-based PDT agent (Figure 73).^408^ In this work, an iodine-substituted BODIPY was chosen as the PS scaffold, and 3-hydroxy benzyl was used as a tyrosinase substrate motif. Meanwhile, an acetyl group was introduced to improve cellular internalization and tyrosinase-catalyzed oxidation. Due to a PET effect from the core BODIPY on the electron-poor pyridinium, the photosensitization activity of **134** was completely inhibited. After entering cancer cells, **134** was first rapidly hydrolyzed by esterase to produce intermediate 1 (**134a**), which was further oxidized into intermediate 2 (**134b**) by tyrosinase. Once the 3,4-dihydroxy benzyl group was generated, a spontaneous 1,6-elimination took place, to release **135**. As the PET acceptor is eliminated, ^1^O_2_ could be produced, with quantum yields determined to be 0.02 for **134** and 0.64 for **135**, respectively.

**Figure 74:**
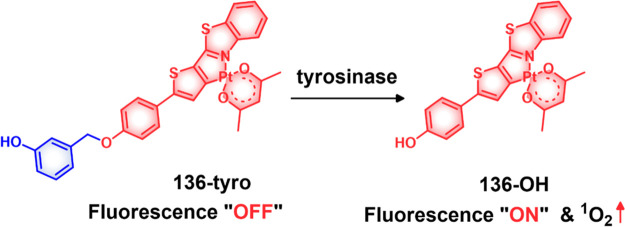
Scheme of **136-tyro** for tyrosinase imaging and PDT.

For fluorescence imaging and PDT of early melanoma, Yoon et al. developed a novel endogenous tyrosinase-activated cyclometalated Pt(II) complex (**136-tyro**), by connecting a 3-hydroxybenzyloxy moiety (as the tyrosinase recognition unit) to the phenyl pendent of a C^∧^N ligand of a cyclometalated Pt(II) complex (as the PSs core).^409^ As depicted in Figure 74, after reacting with tyrosinase, **136-tyro** was converted into **136-OH** through rapid rearrangement and elimination, leading to an obvious fluorescence off-on response at 530 nm. Moreover, the tyrosinase metabolite **136-OH** exhibited a dramatically improved photosensitizing ability relative to **136-tyro**, due to the longer-lived triplet excited state of **136-OH**. Consequently, **136-tyro** can specifically image endogenous tyrosinase in A375 cells (human melanoma cancer cells), and induces a significant growth inhibition toward A375 cells and tumors of A375-bearing Balb/c mice.

##### 2.2.4.7 GGT-Activatable Theranostic Probes in PDT

γ-Glutamyl transpeptidase (GGT), a well-known cell membrane-associated enzyme, can selectively cleave the γ-glutamyl bond of GSH to convert GSH into cysteinyl-glycine (Cys-Gly), to maintain the cellular homeostasis of GSH and cysteine. It has been shown that GGT expression is upregulated in the cell membranes of various malignant tumors, including glioma as well as liver, lung, and ovarian cancers. The elevated level of GGT has been implicated in promoting tumor progression, invasion, and drug resistance, thus making GGT an important marker in tumor diagnosis and an ideal target for tumor treatment. l-Glutamic acid linked through its side chain has been validated as a specific GGT trigger, to mask a fluorophore and sense GGT enzymes.^410,411^

**Figure 75:**
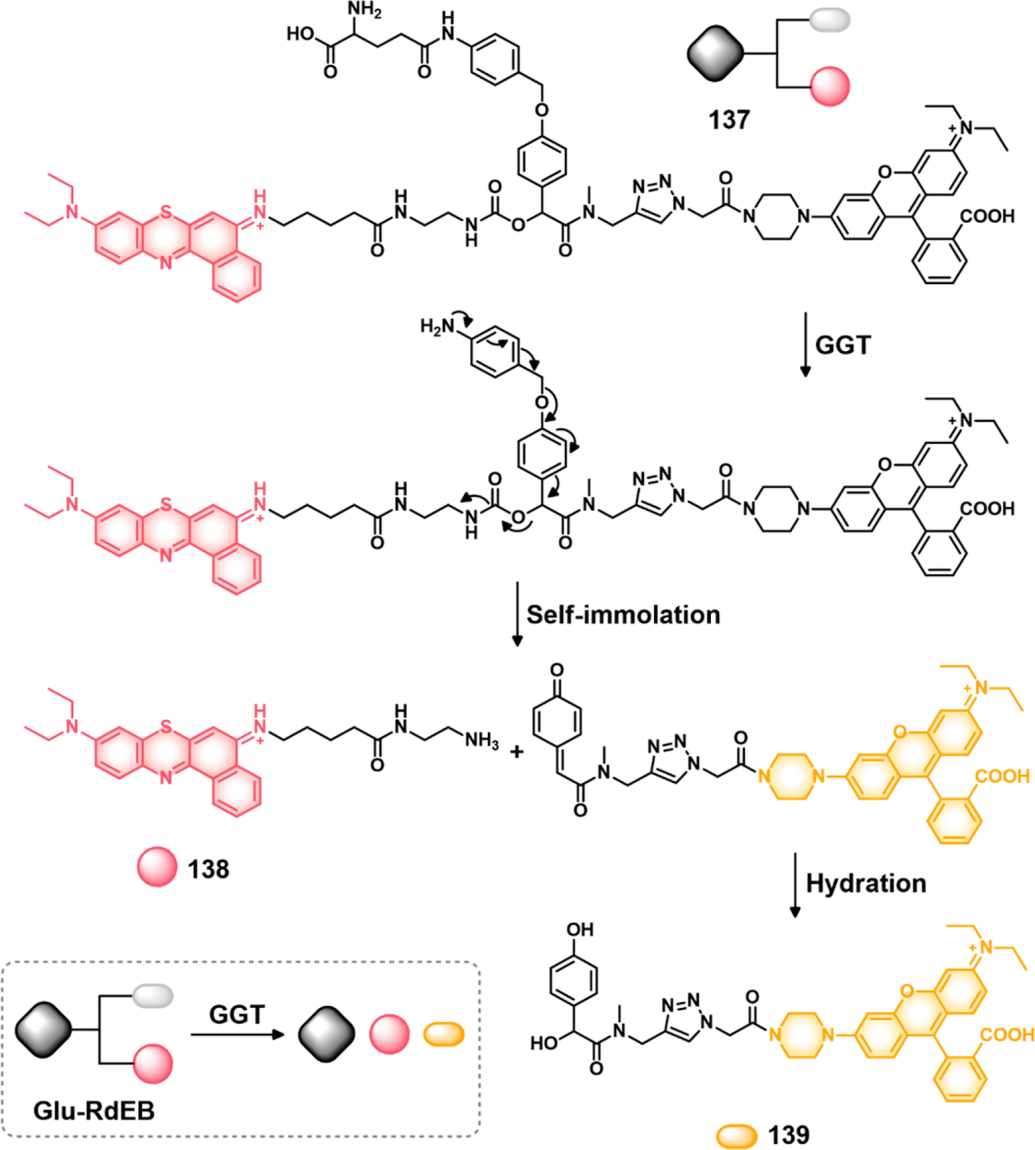
GGT-mediated release of theranostic probes **138** and **139** from **137**.

Based on this, to overcome the off-target biodistribution of PSs in existing PDT systems and achieve tumor-reporting fluorescence, Han and Li et al. designed and synthesized a trifunctional small-molecule probe, called **137**, that contains a type I PS (ENBS), linked to a fluorescence reporter (rhodamine, Rd) via a self-immolative linker, and an l-glutamic acid moiety as a GGT trigger (Figure 75).^412^ In **137**, both the fluorescence and the ROS generation properties of ENBS are almost unaffected by the rhodamine fluorescence, while the fluorescence of Rd is in an “off” state due to the quenching effect between ENBS and Rd. Upon catalytic activation by tumor-associated GGT, **137** releases rhodamine and ENBS, leading to activation of the rhodamine fluorescence. The released rhodamine and ENBS were accumulated in lysosomes, an organelle critical for triggering cell death. Under light irradiation, **137** effectively damaged lysosomes and induced a 2-fold enhancement of cell death in U87 cells (GGT+) over LO2 cells (GGT−). In BALB/c nude mice bearing subcutaneous U87 tumors, **137** exhibited an intense rhodamine fluorescence selectively confined to tumor foci rather than other organs, revealing the potential of **137** in discerning tumors from healthy tissues by a tumor-activated “Turn-On” fluorescent response. Meanwhile, the tumor-specific optical readouts help to direct light irradiation for precise PDT. Overall, this work demonstrated a fluorescently quenched dye–PS pair to yield PS-independent tumor-activatable fluorescence to guide PDT.

##### 2.2.4.8 Aminopeptidase *N*-Activatable Theranostic Probes in PDT

**Figure 76:**
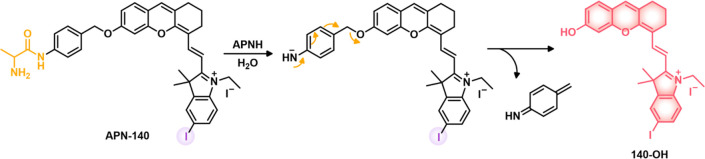
Response mechanism of **APN-140** with APN.

Aminopeptidase *N* (APN/CD13) is a typical membrane-binding zinc-dependent type II metalloproteinase that can specifically cleave an N-terminal leucine amino acid from a protein polypeptide chain. APN plays vital roles in the growth, division, metastasis, and angiogenesis of malignant tumors. Extensive studies have demonstrated that APN is significantly overexpressed on the cell membrane surface in various tumors (∼10-fold than normal tissue), especially in colon, ovarian, breast, and liver tumors. Thus, APN is widely recognized as a distinct marker for cancers and has received attention as a cancer-selective enzyme enabling the design of selective activatable fluorescent probes and theranostic prodrugs. Peng and co-workers reported for the first time an APN-activated near-infrared PS (**APN-140**) by linking an iodine-substituted hemicyanine and l-alanine through a 4-aminobenzyl alcohol linkage (Figure 76).^413^ In normal cellular environments, **APN-140** is maintained in an “OFF” state, with quenched fluorescence and phototoxicity as a result of an inhibited ICT process. By contrast, overexpressed APN in tumor cells specifically lysed the amide bond between l-alanine and the 4-aminobenzyl alcohol group, which is followed by a spontaneous 1,6-elimination to form **140-OH**. **140-OH** displayed a greatly enhanced NIR fluorescence signal and ^1^O_2_ yield compared to **APN-140**, and specifically accumulated in the ROS ultrasensitive mitochondria. Under 660 nm light irradiation, **APN-140** efficiently induced cancer cell apoptosis by destroying mitochondria and more importantly could distinguish cancer cells from normal cells, leading to the selective killing of cancer cells and a distinct tumor suppression effect.

##### 2.2.4.9 Other Examples

Apart from the aforementioned activation types, researchers have also made significant advances in the development of other types of activatable PSs. Some examples primarily focus on tailored PSs for specific diseases by employing specific markers as activation triggers.

##### 2.2.4.9.1 F^–^ Activation

**Figure 77:**
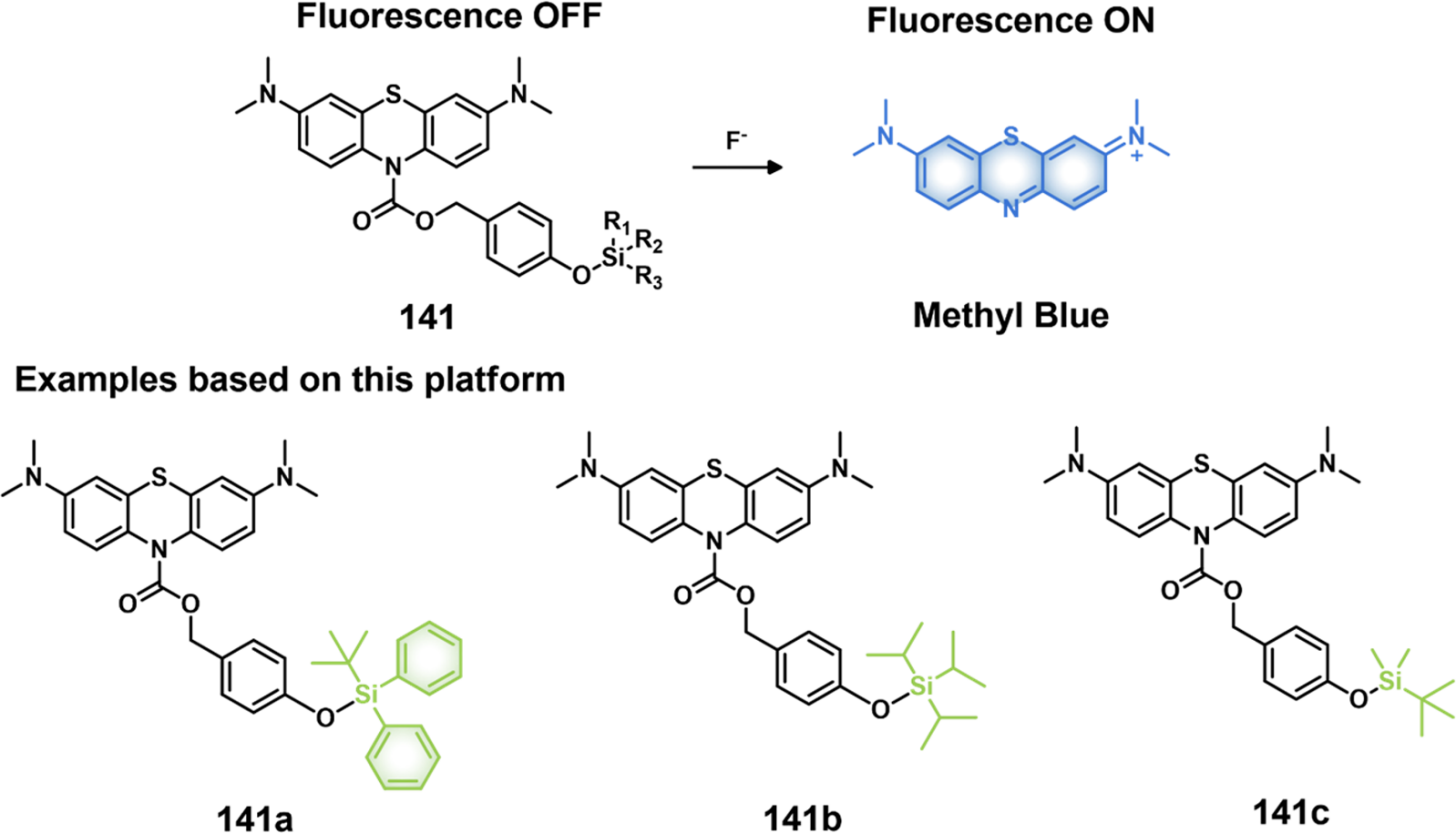
Fluoride-activated MB releasing platform of **141** and three candidate theranostic probes in this work.

Fluoride is extensively utilized as an anticaries agent in oral health care, in which fluoride is absorbed by the dental plaque biofilm, thereby acting as a fluoride reservoir in the oral cavity. With this in mind, Yi et al. designed a panel of fluoride-activated PSs (**141**) for imaging and treatment of human dental plaque (Figure 77).^414^**141** was synthesized by linking leuco-MB to different silyls with different substituents (TBDPS, TIPS, and TBDMS), as silyl ethers are selectively and rapidly cleaved by fluoride. In vitro experiments revealed that **141c** possessed the best responsiveness toward fluoride, including a quick desilylation rate and high selectivity as well as a low detection limit. **141c** can not only be used to map naturally grown human plaque biofilms by the NIR fluorescence of MB but can also be used to realize fluoride-regulated antimicrobial PDT.

##### 2.2.4.9.2 Photoactivation

**Figure 78:**
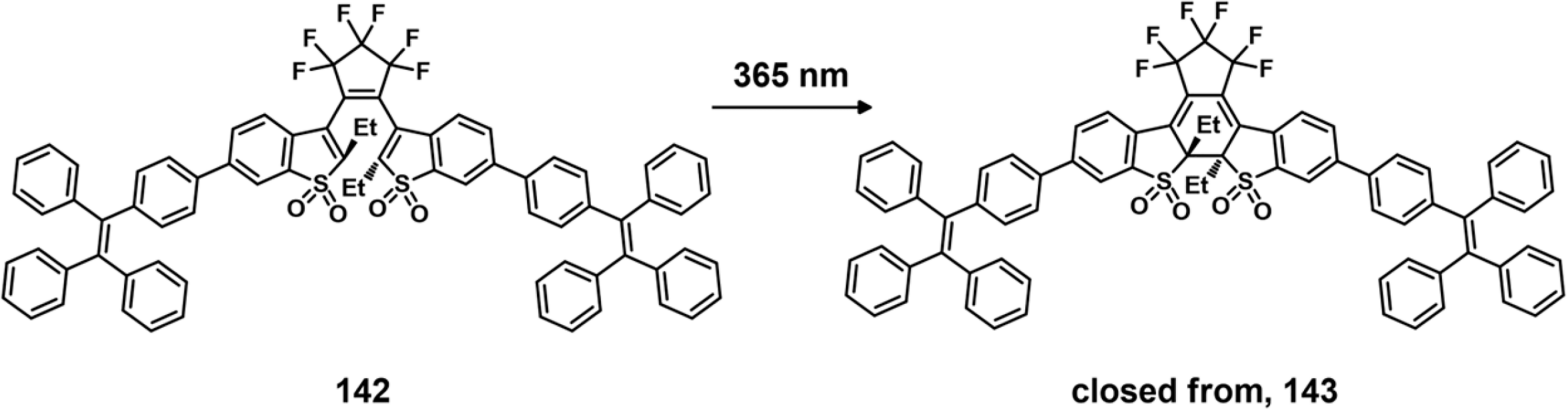
Chemical transformation of theranostic probe **142** (closed form) to **143** (open form) under light irradiation.

Photoswitching molecules can also be used in activation strategies. For example, in the context of fluorescent probes and super-resolution imaging, researchers have explored diverse photo-switches based on different dye skeletons.^415,416^ Very recently, Tang et al. developed a UV light-activatable PS (**142**), which integrated tetraphenylethylene (TPE) with a diarylethene (DAE) at the 6-positions of constituent benzothiophenes (Figure 78).^417^ The open form (OF) of **142** demonstrated short-wavelength absorption/emission and AIE characters; once activated by UV light (365 nm), its closed form (CF) quickly formed, accompanied by a bathochromic-shift towards the visible light range and an enhanced fluorescence intensity (λ_em_ = 660 nm) as well as a dramatically strengthened photosensitization capability. **142** was coated with F-127 to form **142** nanoparticles (**142-**NP) with the aim of improving the water solubility and cellular uptake. Strikingly, **142** retains its photo-switching properties inside the NPs, and the cyclization can be fully finished within 300 s. Finally, a PDT effect of **142**-NP was elicited in HeLa cells, in which photoinduced cancer cell death was found to be closely related to apoptosis or necrosis pathways. This research exemplifies the utilization of photo-switches for the development of light-activatable PSs with high spatiotemporal resolution.

##### 2.2.4.9.3 Enzyme-Responsive Double-Locked Molecular Beacon

**Figure 79:**
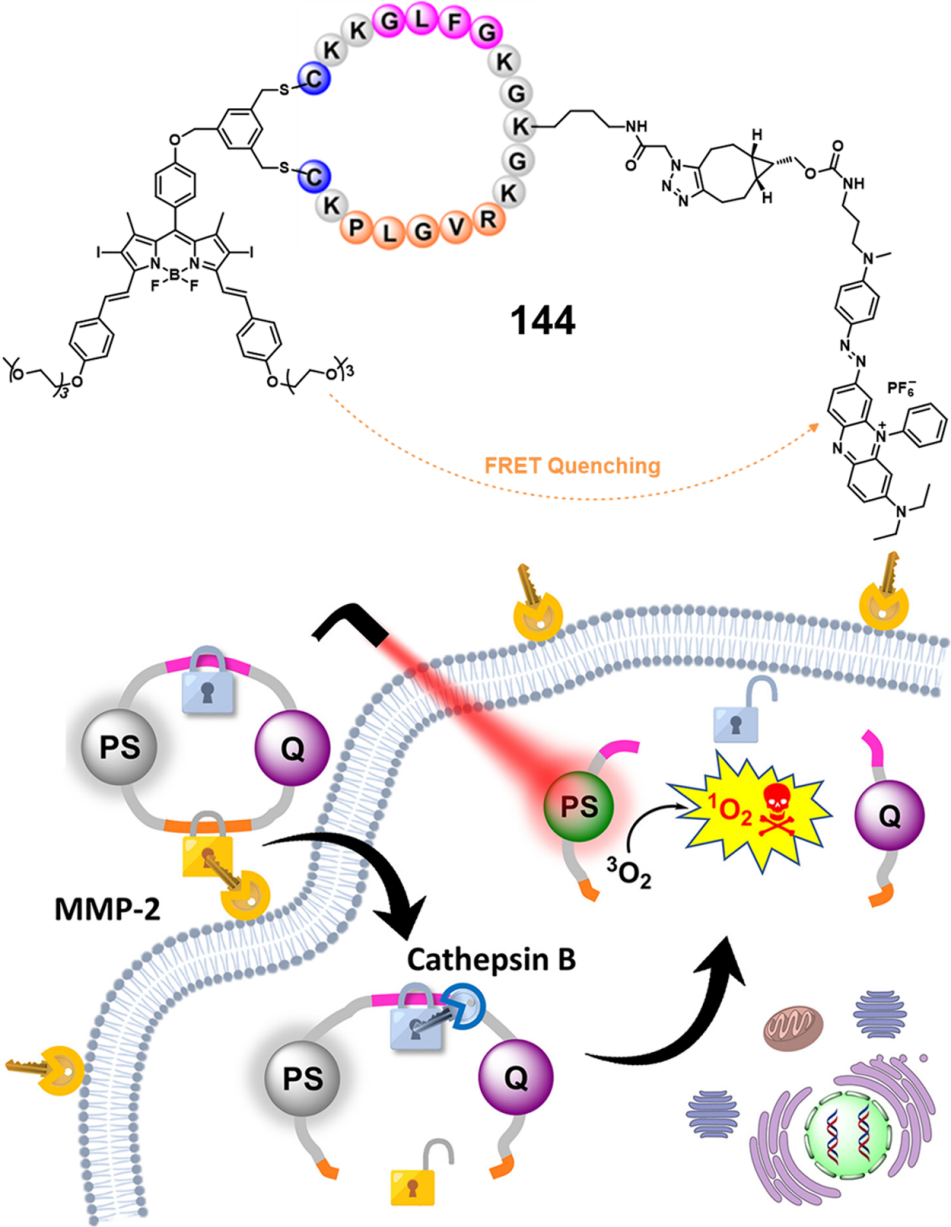
Molecular structure of the double-locked theranostic probe **144**. Reproduced with permission from ref (418). Copyright 2023 American Chemical Society.

A photodynamic molecular beacon (PMB) is an inherent self-quenched PS due to intramolecular energy or electron transfer. Generally, PMB molecules are composed of three parts, (i) a PS, (ii) a quencher, and (iii) a linker that can be recognized by specific stimuli (e.g., enzymes). In PMB molecules, utilizing a resonance energy transfer between the PS and quencher is a common strategy, where the excited state energy of the PS is transferred to the quencher after excitation and the resultant dark state of the PS further hinders ROS generation. However, when the linker is cleaved at a specific site (tumor or other diseased cells), the resonance energy transfer loses efficacy, owing to the spatial separation of the PS and the quencher, further switching on the photo-induced ROS generation. Ng and Lo et al.^418^ constructed a photodynamic molecular beacon molecule **144** by utilizing the classic iodinated-BODIPY scaffold, Black Hole Quencher 3 (BHQ-3) as a quencher, and a cyclic peptide containing PLGVR and GFLG peptide sequences that can be recognized and cleaved by metalloproteinase-2 (MMP-2) and cathepsin B, as shown in Figure 79. In **144**, the excited state of the BODIPY scaffold was fully quenched by BHQ-3 and kept **144** in an “inactive” state (Φ_F_ = 0.01, Φ_Δ_ = 0.02), thereby reducing its adverse side effects on normal tissues. Once taken up by MMP-2 and cathepsin B overexpressing cancer cells (e.g., the A549 cell line), cascaded hydrolysis releases the photoactive DSBDP and restores its photodynamic activities. Moreover, the released dye is selectively localized in the lysosomes of cancer cells and causes significant tumor cell death (IC_50_ = 0.78 ± 0.04 μM for A549) after irradiation (λ > 610 nm, 23 mW cm^–2^, 28 J cm^–2^). In an A549 tumor-bearing mice model, **144** effectively suppressed the tumor growth without notable side effects. In particular, **144** does not induce any degree of skin photosensitivity, which is a well-recognized side effect of conventional “always-on” PSs.

### 2.3 Theranostic Fluorescent Probes for PTT

#### 2.3.1 Tumor Microenvironment (TME) Activated Theranostic Probes in PTT

PTT utilizes mild and highly penetrating infrared light to convert external light energy into heat in the presence of a PS, thereby increasing the local temperature. When this photoactivation takes place in a tumor, the in situ generated heat can kill cancer cells without the need for cytotoxic agents being used. PTT has garnered significant research interest due to its non-invasiveness and biosafety due to the use of light to precisely control heat production and the preclusion of drug resistance. TME modulates tumor survival, progression, and metastasis, and has thus become a potential target for activatable tumor imaging and treatment. Considering the inadequate therapeutic efficacy of traditional photothermal agents (PTAs), TME-activatable PTAs have been extensively developed in recent years for targeted cancer therapy. The TMEs exploited include acidic pH, hypoxic conditions, and cancer-specific enzymes.

**Figure 80:**
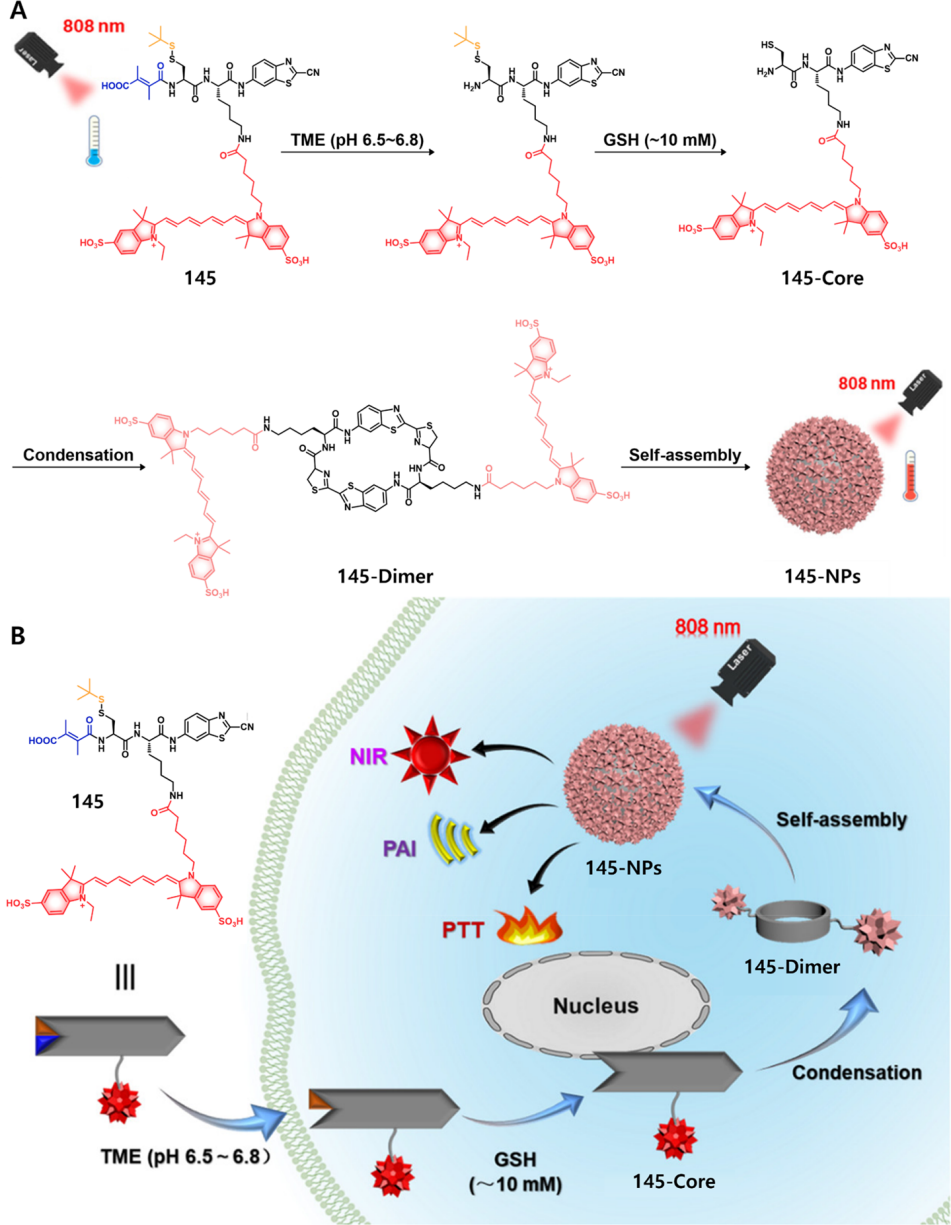
(A) Schematic diagram of the formation of **145-**Dimer mediated by low pH and GSH to in situ generate **145-**NPs with recovered photothermal conversion. (B) Schematic illustration of the use of **145-**NPs for activated bimodal imaging and PTT of cancer cells. Reproduced with permission from ref (419). Copyright 2020 American Chemical Society.

The TME is characterized by weakly acidic pH conditions resulting from abnormal glycolysis, offering promise for targeted, activatable imaging and therapy of cancer. Shi et al. reported an intelligent near-infrared (NIR) photosensitive probe Cy-1 (**145**), which can be activated under the synergy of acidic pH and GSH to self-assemble into nanoparticles (**145**-NPs) in situ in cancer cells (Figure 80).^419^ When **145** was internalized, it underwent simultaneous citric acid hydrolysis under weakly acidic pH and a disulfide bond cleavage by GSH to produce **145**-Core. In tumor cells where a high level of active **145**-Core exists, formation of an amphiphilic cyclic dimer (**145**-Dimer) occurs. Subsequently, π–π stacking between **145** species generated **Cy-NPs** with enhanced retention in tumors, as well as activated NIR/photoacoustic (PA) imaging modalities and PTT in vivo.

**Figure 81:**
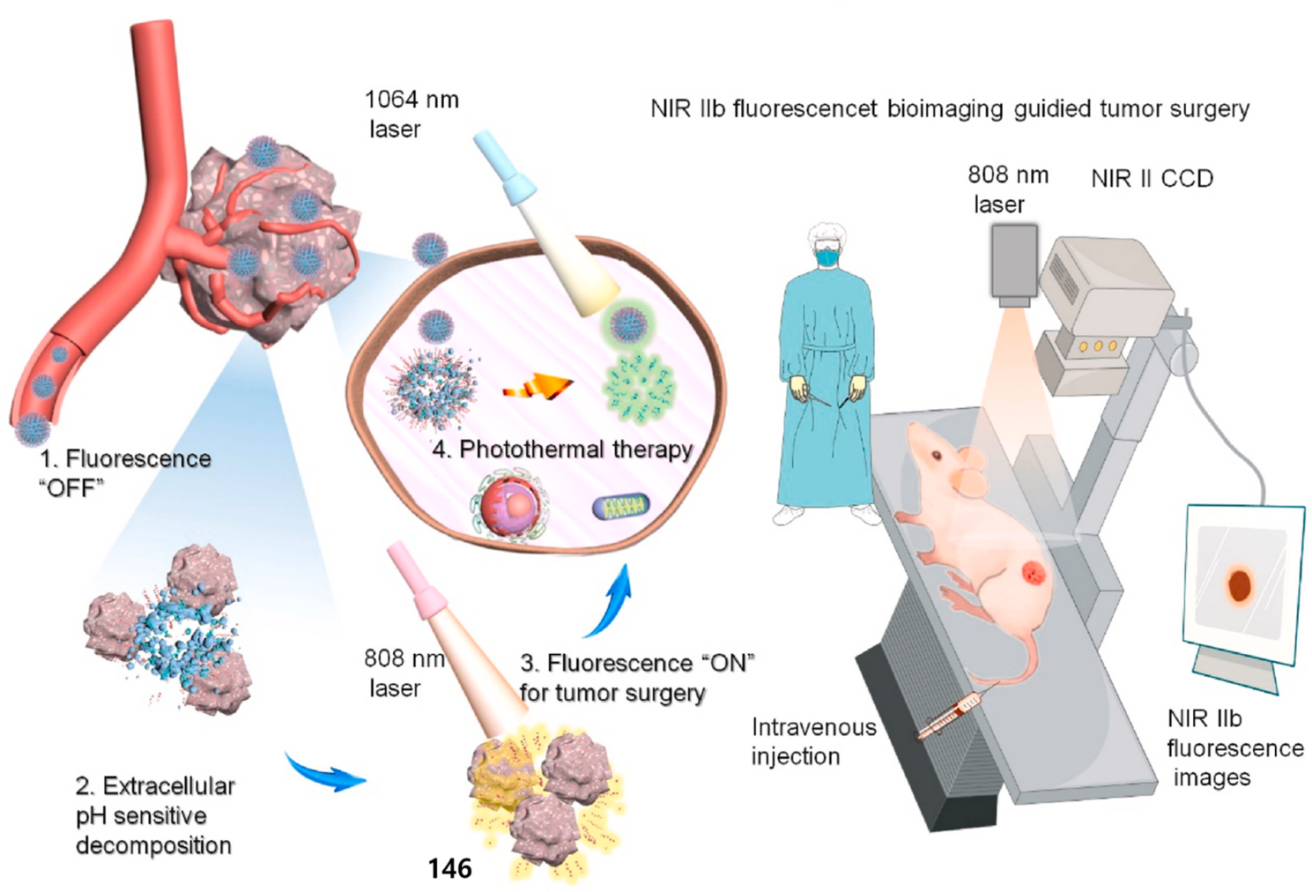
NIR fluorescence of theranostic probe **146** is activated by low pH to achieve NIR-IIb fluorescence imaging-guided PTT of solid tumors. Reproduced with permission from ref (420). Copyright Elsevier Ltd.

The expression level of biomarkers varies from individual to individual. To improve the sensitivity of PTAs to a given TME, Liu et al. developed a pH-sensitive nanoprobe (Figure 81).^420^ The nanoprobe was composed of NIR-IIb quantum dots PbS@CDS, which were conjugated to the surface of a hollow MnO_2_ nanoshell. A molecular probe IR1061 was then loaded to obtain HvMnO_2_@Qds-IR1061 (**146**). The fluorescence signal was absent in normal cell tissues due to the absorption competition-induced emission (ACIE) mechanism. However, in the weakly acidic TME, the HvMnO_2_ shell was degraded, releasing the encapsulated IR1061 for NIR imaging. Then, treatment using 1064 nm laser irradiation for 5 min increased the local temperature of the tumor from 21 to 72 °C to kill cancer cells, thus achieving imaging-guided PTT, which holds significant promise for surgical applications.

**Figure 82:**
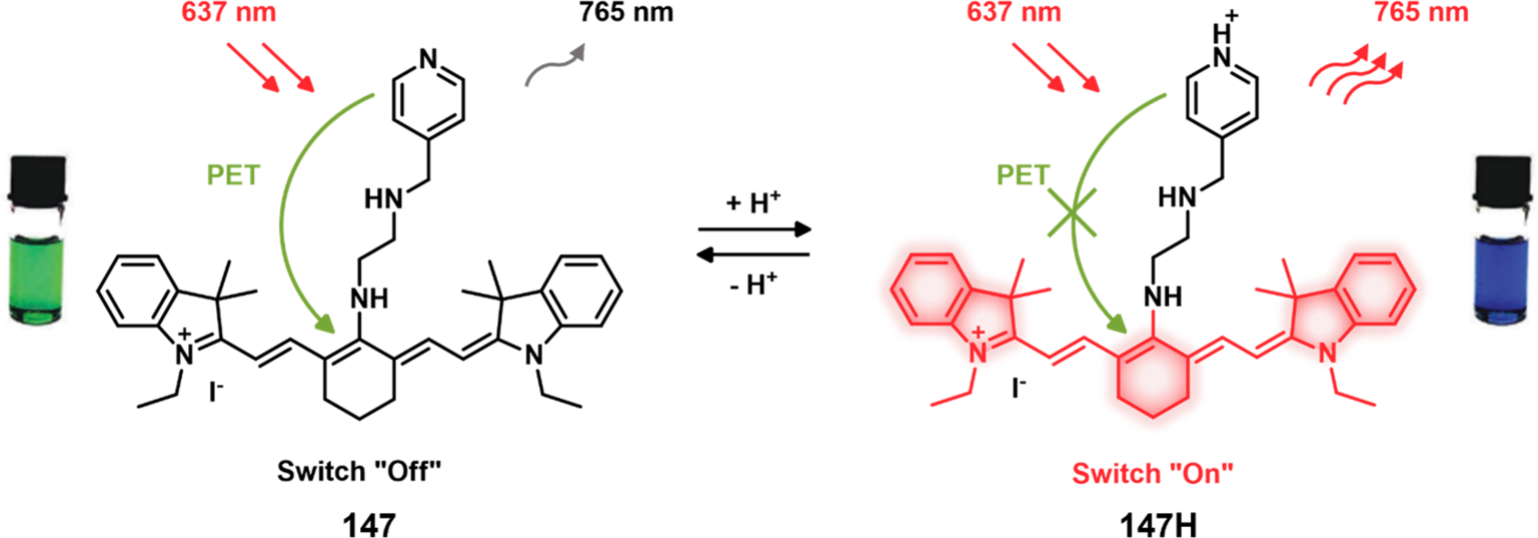
Chemical structures and schematic illustration of **147** as a pH-responsive theranostic probe for NIRF/PA dual-modal imaging-guided PTT therapy. Reproduced with permission from ref (422). Copyright 2017 Royal Society of Chemistry.

Based on a similar strategy, Park et al. developed a pH-responsive nano-probe that consists of carbonized crosslinked poly(ethylene glycol-*g*-poly(sulfobetaine methacrylate)) loaded with a photothermal dye IR825 (FNP-I).^421^ Cai et al. used IR-822 as the PTA to which a proton receptor N1-(pyridine-4-methyl) ethane-1pr 2-diamine (PY) was introduced to form a fluorophore-spacer-receptor molecular probe **147** with quenched fluorescence (Figure 82).^422^ After being protonated in the acidic TME, the probe allows for NIRF/PA dual-modal imaging-guided PTT applications.

It has been reported that the rough surface of viruses is composed of thorn-like structures that strongly bind to the cell membrane during the process of virus invasion. In 2019, Liu et al. presented a nano-probe that utilizes the biomimetic targeted therapy characteristic.^423^ The bionic method was used to coordinate the infrared fluorescent IR825 with the chemotherapeutic drug PEM and rare earth metal particles (Nd^3+^, Nd^2+^) to design a self-targeting NIR-II nano-sheet. The Nd^3+^ ion was used as a converter to transform them into virus-like nanoparticles. Tumor acidity-sensitive PEG was used to cover the surface of the virus-like nanodrug, resulting in the creation of virus-core and sphere-shell hierarchical nanostructures under physiological conditions. When triggered by a weakly acidic tumor microenvironment, the “sphere-to-virus” shape reversal occurred, which led to an enhancement in photothermal conversion efficiency, an increase in cell adhesion through a virus-like rough surface, and the activation of folate-receptor-mediated self-targeting realized biomimetic targeted therapy effect of the tumor.

**Figure 83:**
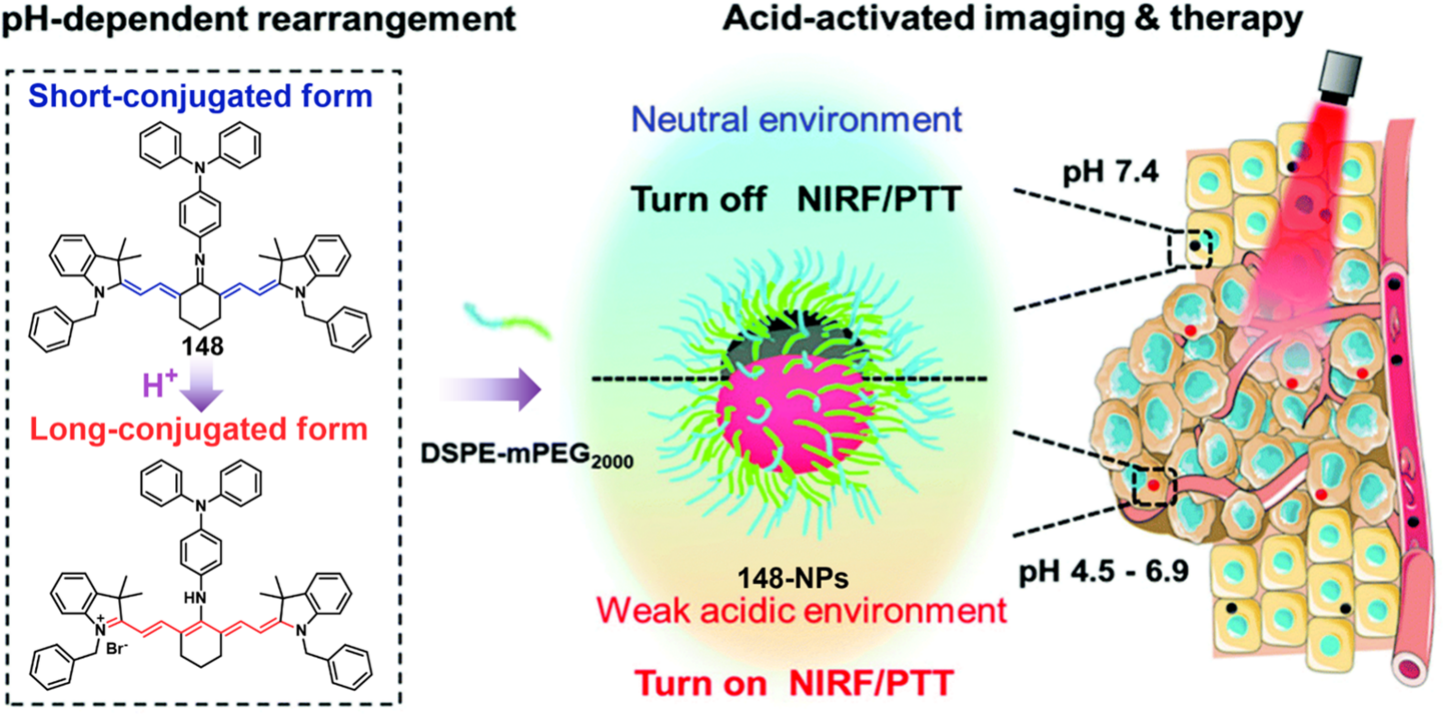
Schematic diagram of **148** NPs that rearrange in a weakly acidic environment to “Turn-on” NIRF imaging-guided PTT. Reproduced with permission from ref (425). Copyright 2022 Royal Society of Chemistry.

Furthermore, Yan et al. investigated a nanoplatform for simultaneous photothermal and photodynamic (PTT/PDT) therapy with acid activation and when subjected to external radiation, which can be used for accurate tumor-targeting near-infrared (NIR) image-guided therapy.^424^ The pH-responsive brominated asymmetric cyanine (BAC) was an activable near-infrared PTT/PDT-in-one reagent for accurate tumor-targeted therapy. BAC was connected to persistent luminescence nanoparticles (PLNPs), then biotin (BT) functionalized polyethylene glycol (PEGBT) was introduced into the PLNP to form PTT/PDT-in one nanoplatform (PLNP-BAC-PEGBT), which showed accurate tumor-targeted imaging and strong PTT/PDT effects. In 2022, Yin et al. constructed an organic nanoprobe with acid-activated heptamethine cyanine (Cy-TPA, **148**) (Figure 83).^425^ The nanoprobe can “Turn on” the PTT effect and restore near-infrared fluorescence in a weakly acidic tumor microenvironment. Simultaneously, **148** NPs can accumulate in the tumor site, prolong its retention time, and significantly improve the PTT effect, fluorescence signal activated by the acid environment also provides guidance for PTT therapy. Moreover, Liu et al. developed a series of acid-triggered NIR upconversion NPs (NRhD-PEG-XNPs), which were constructed by rhodamine dyes conjugated with PEG^426^ and showed good upconversion luminescence and an enhanced photothermal effect in a weak acid environment. NRhD-PEG-XNPs could achieve accurate tumor targeting and PTT without showing side effects, providing a new research strategy for activatable theranostic nanoplatforms.

Human serum albumin with good carrier performance was coated with pH-responsive fluorescent dyes to form a self-assembling complex, when triggered by a weakly acidic tumor microenvironment, the complex forms nanoparticle aggregates at tumor sites, which effectively achieves photodynamic and photothermal treatment effects of tumors. Liu et al. developed albumin-dye nanoparticles (HSA-Croc) by pH-sensitive croconaine (Croc) dye self-assembled with HSA.^427^ While under acidic conditions, Croc dye has strong absorption at 790 nm and HSA-Croc nanoparticles exhibit accurate tumor-specific photoacoustic imaging and PTT. With the help of similar functions of biological proteins, Yi et al. developed BSA-pH-PTT nanoparticles using pH-sensitive asymmetric cyanine dye (pH-PTT) self-assembled with BSA.^428^ Under acidic conditions, pH-PTT was converted into a larger conjugated structure with strong absorption at 808 nm, and the self-assembled system formed nanoparticles, which could be concentrated at the tumor site to achieve an effective photothermal therapeutic effect.

#### 2.3.2 Hypoxic Activated Theranostic Fluorescent Probes in PTT

Hypoxia is a common feature in most solid tumors and TME, resulting from uncontrolled proliferation of tumor cells, abnormal blood vessels, and insufficient oxygen supply. However, healthy tissues do not contain hypoxic regions, making hypoxia a specific target for selective cancer treatment. Various mechanisms promote the adaptation of tumor cells to this adverse environment and ultimately enhance drug resistance and survival.^429,430^ PTT is a promising treatment strategy for hypoxic tumors due to its non-oxygen dependence and high spatiotemporal accuracy.^431^ PTT uses photothermal agents to trigger local hyperthermia under near-infrared light (NIR) irradiation, with high therapeutic efficiency, leading to irreversible ablation of tumor cells.^432,433^ To achieve hypoxic reactivity in PTT, hypoxic targeting vectors can be used to deliver photothermal agents or hypoxic-responsive groups can be introduced to activate them.^434,435^ These strategies are essential for triggering photothermal properties, and the development of novel hypoxia-induced photothermal agents is crucial for controlling the “on/off” state of PTT.^436^

**Figure 84:**
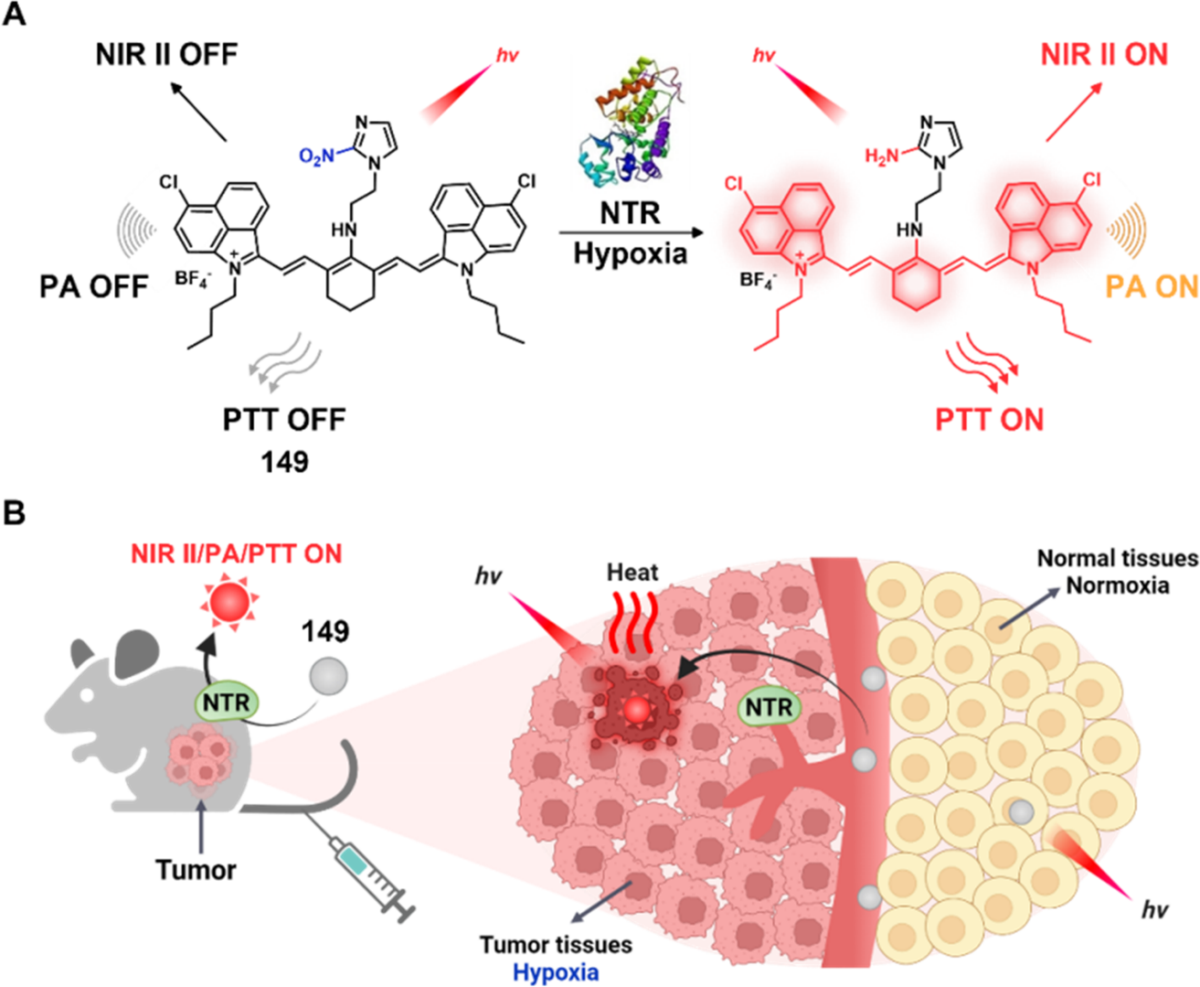
Schematic illustration of the NTR-responsive NIR-II fluorescence/PA probe **149** for visualizing tumors and inducing an NTR-triggered PTT effect.

PTT based on the principle of photothermal conversion has great potential in the effective treatment of cancer, with high tumor ablation efficiency and minimal side effects on normal tissues.^437^ Cai et al. developed a novel single-molecule probe **149**, which was triggered by hypoxia and enzymatic reaction with nitroreductase (NTR). The probe was conjugated with a nitro imidazole group as a specific hypoxia trigger with an IR-1048 dye as a NIR-II/photoacoustic (PA) signal reporter, allowing for high-contrast tumor visualization by NIR-II fluorescence imaging and deep-tissue penetration using 3D PA imaging. Theranostic probe **149** exhibits significant photothermal effects and NIR-II fluorescence when activated at hypoxic tumors, providing more accurate and deeper tissue imaging of tumor location and boundaries for tumor ablation without recurrence. Therefore, the multifunctional **149** probe represents a rapid and sensitive NIR-II fluorescence/photoacoustic imaging probe for hypoxia, that can be used as an activated small molecule PS for PTT (Figure 84).^438^

**Figure 85:**
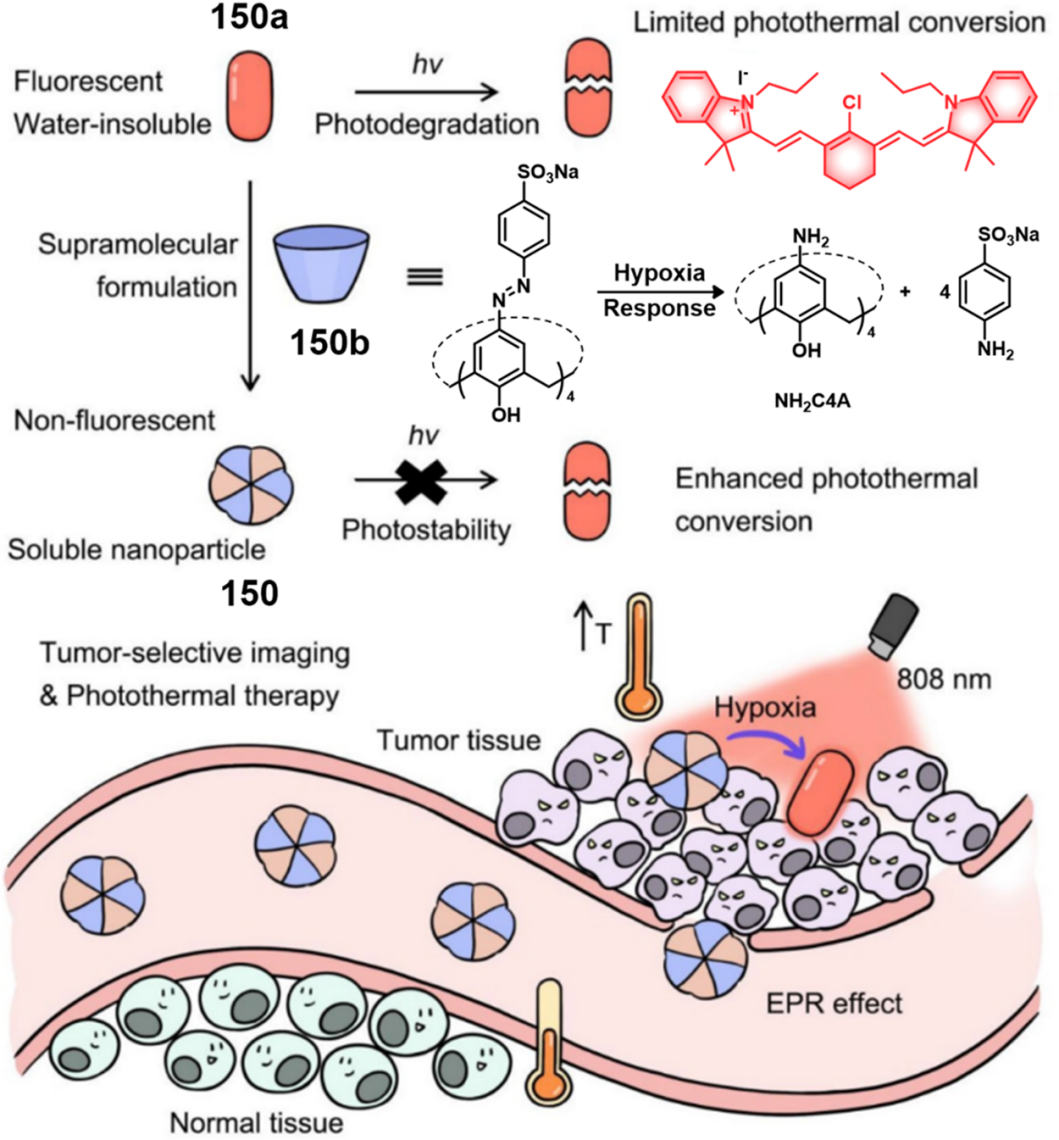
Schematic illustration of supramolecular Photothermal agents **150** constructed using **150a** and **150b**. And the tumor-selective imaging and PTT application of the supramolecular nanoformulation **150**. Adapted with permission from ref (439). Copyright 2022 Ivyspring International Publisher.

In order to overcome the hydrophobicity and photodegradation of traditional photothermal reagents. Guo et al. developed a supramolecular PTT system **150** by complexing sulfonated azocalix arene (SAC4A) with organic small molecule photothermal agent IR780, which significantly improved the solubility, photostability, and photothermal conversion rate of the photothermal agent. SAC4A and its reductase reduction products NH_2_C4A were able to increase the photostability and photothermal conversion of IR780, further enhancing PTT efficacy. This hypoxia-responsive probe-based release of IR780 (fluorescence ON state) from **150** (fluorescence OFF state), enables tumor-selective imaging and supramolecular PTT of **150** in vitro and in vivo (Figure 85).^439^

**Figure 86:**
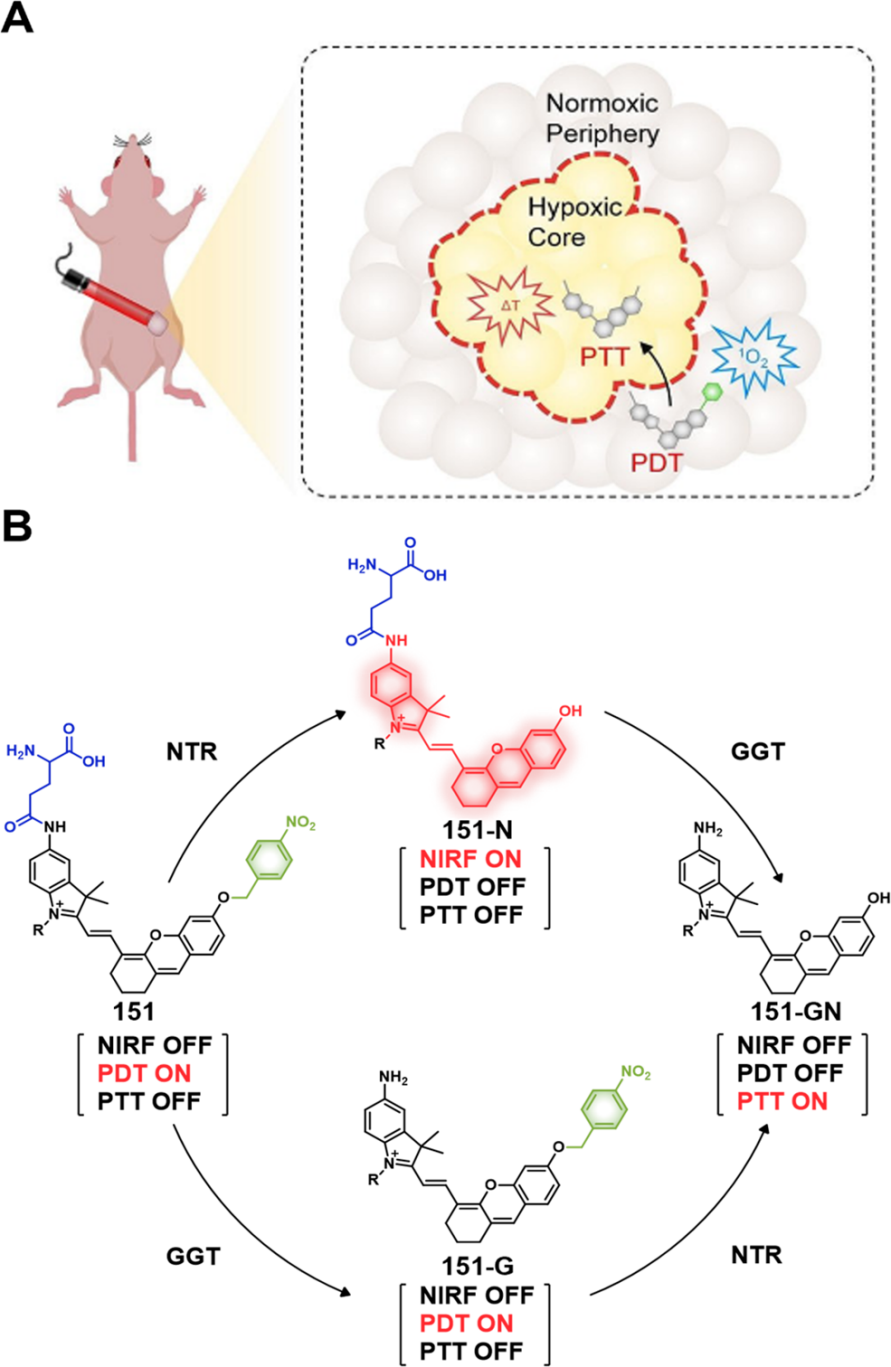
(A) Schematic illustration for autoregulated PDT-PTT in the tumor. (B) Scheme describing the molecular mechanism of **151** for real-time imaging of tumor and autoregulated PDT-PTT in the presence of GGT and NTR. Reproduced with permission from ref (440). Copyright 2022 Wiley Intersciences.

**Figure 87:**
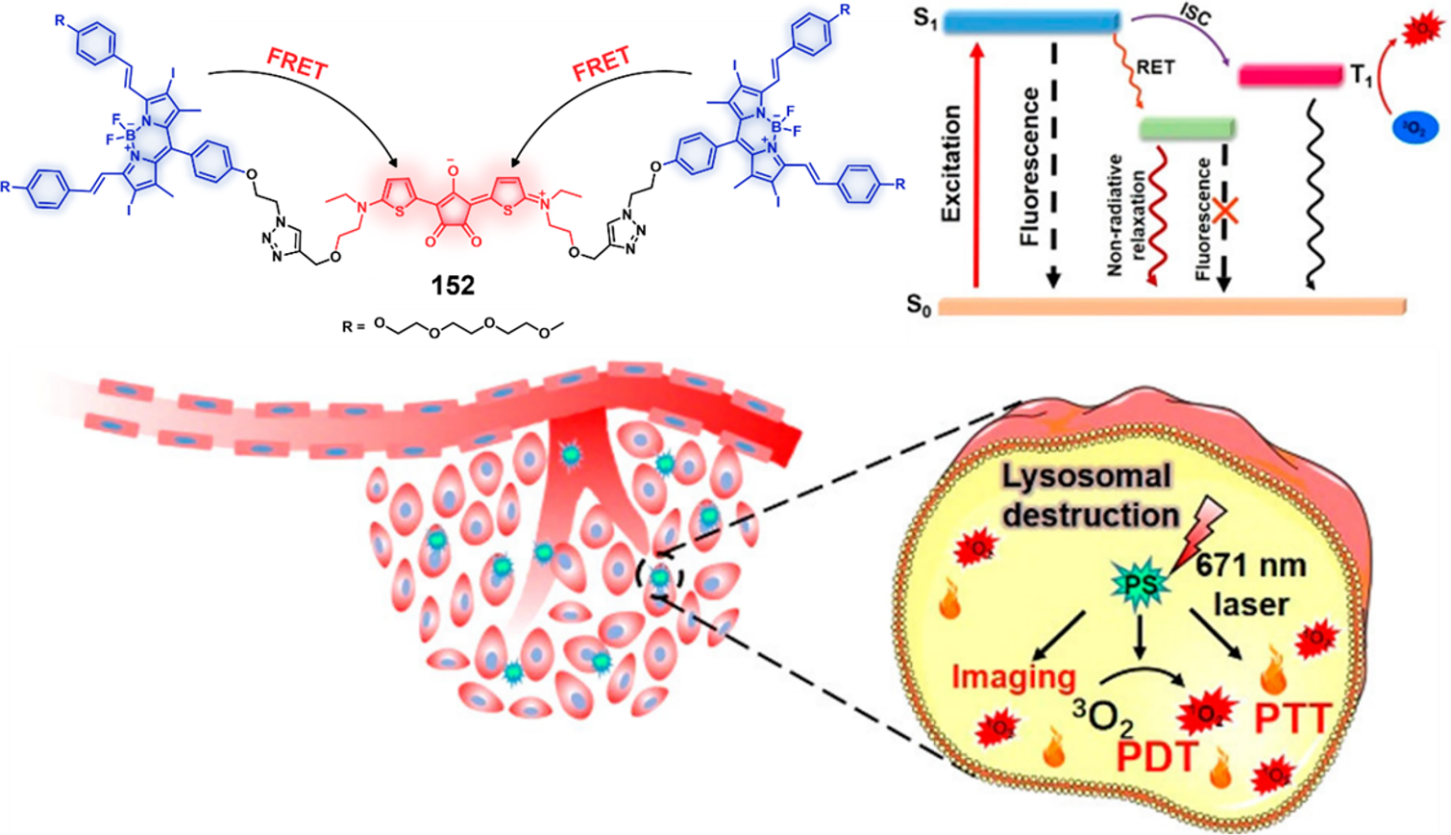
Schematic illustration of the smFRET-based combination phototherapy mechanism (**152**) and light-triggered cancer cell death.

Activatable phototheranostics exhibit promise for the precise treatment of cancer. However, most probes in this category only offer PDT or PTT, which may be less effective due to cellular hypoxia and complex TME. To address these issues, Pu et al. have developed a dual-locked activatable phototheranostic probe **151**. This probe emits near-infrared fluorescence (NIRF) signals in tumors, triggers PDT in response to a tumor-periphery biomarker, and switches from PDT to PTT when a hypoxic biomarker is detected in the tumor core. This PDT-PTT auto-regulated probe can generate cytotoxic ^1^O_2_ around the tumor with a single laser source, producing thermotherapeutic effects in the tumor core that lead to complete tumor ablation. This double-locked probe thus shows promise as a molecular design strategy for precise cancer phototheranostics (Figure 86).^440^ Moreover, Peng et al. used the smFRET-guided smart molecule design methods incorporating diiododistyrylbodipy (BDP) and croconaine (CR) to construct PS BDP-CR (**152**), which showed an encouraging “1 + 1 > 2” effect on the combination of PDT and PTT in tumors, due to the synergistic effect of PTT and PDT, broadened the spectral absorption range in the NIR region 600–850 nm and improved the light-harvesting ability. Under normoxic conditions, the absorbed photon energy mainly sensitizes oxygen to generate ROS for PDT and stimulates CR by FRET for PTT. Under severe hypoxic conditions, the photoenergy will mainly kill cancer cells via O_2_-independent PTT (Figure 87).^441^ To overcome the low efficacy of PDT in hypoxic tumor environments, Liu et al. developed a novel boron dipyrromethene (BODIPY)-based PS (Ion-BDP), which was regulated by nitroreductase that is overly expressed in the hypoxic tumor microenvironment.^436^ When Ion-BDP was exposed to near-infrared light, it produced ROS for PDT while also consuming oxygen. In anaerobic microenvironments, nitroreductase cleaves 4-nitrobenzyl from Ion-BDP and converts it into a photothermal agent called BDP for PTT. Studies have shown that Ion-BDP was fluorescent, but this fluorescence was weakened by the PeT effect of activated BDP. These “on–off” PSs provide a model for the development of single-wavelength light sources to stimulate photodynamic and photothermal therapies, simplifying multi-mode phototherapy treatments. Additionally, other dual-function probes with synergistic PDT/PTT therapeutic properties also have been developed, such as 2TPAVDPP, TPATPEVDP, 2TPEVDPP,^442^ AzoCyS-N NPs,^443^ and LCT-CyI-TPZ.^444^

**Figure 88:**
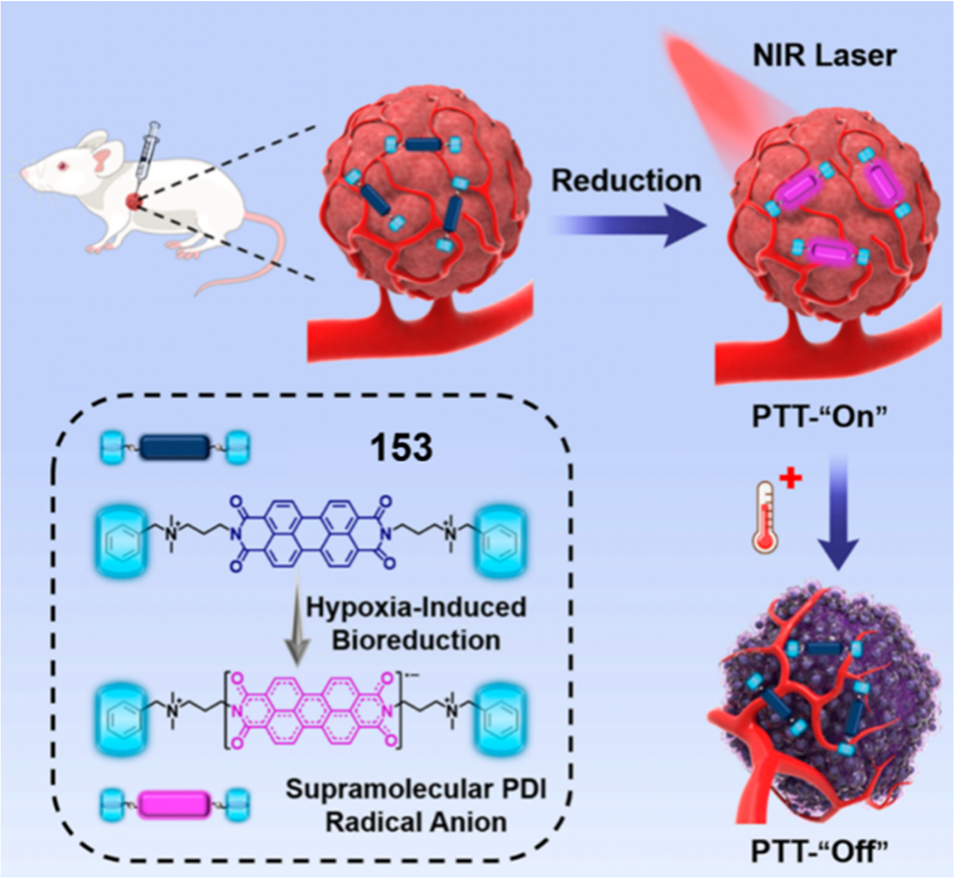
Schematic representation of in situ hypoxia-induced supramolecular PDI radical anions in tumors for specific PTT with controlled “on–off” states. Reproduced with permission from ref (445). Copyright 2022 American Chemical Society.

To enhance the photothermal conversion efficiency of supramolecules in tumor hypoxia, Zhang et al. created supramolecular complex **153** by combining a PDI derivative with CB[7].^445^ It was shown that **153** produced a supramolecular perylene imide radical anion in the hypoxic environment, which could be controlled by restoring oxygen supply to the tumor. Through quenching of the supramolecular PDI radical anions, the “on–off” states could be achieved, significantly improving the photothermal conversion efficiency and inhibiting the expression of HIF-1 (Figure 88).

**Figure 89:**
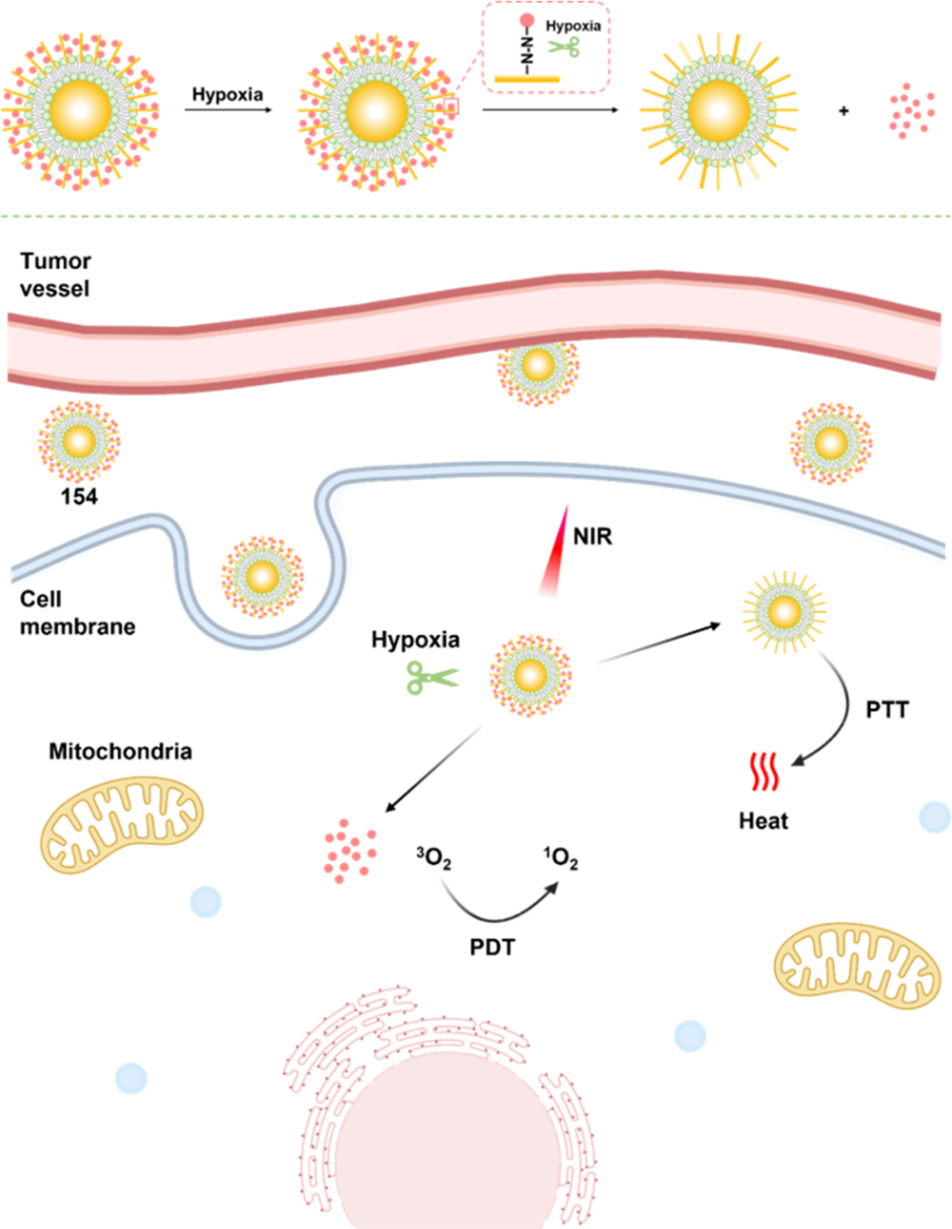
Mechanism of **154-**NPs for the detection of hypoxia and the PDT/PTT of tumors. Reproduced with permission from ref (446). Copyright 2013 Royal Society of Chemistry.

In addition to PDT and PTT synergistic probes, other multi-functional systems based on anaerobic environmental activation have also been developed, such as PTT/bio-imaging and drug/gas synergistic therapy. Chen et al. have designed a dual-function nanomaterial Cy-C-S-NPs (**154-**NPs) for precision tumor therapy and imaging,^446^ which consists of a gold nanomaterial with a surface-modified phospholipid layer (C-S-NPs) that is then coated with near-infrared fluorescent dye Cy-DM. The fluorescence of Cy-DM was quenched by C-S-NPs in a non-hypoxic environment. However, the unique hypoxic microenvironment of tumor cells leads to the breaking of the azo bonds, releasing Cy-DM and generating a fluorescence response. Therefore, **154-**NPs can achieve dual imaging by fluorescence molecular imaging and Raman imaging, and generate PDT and PTT simultaneously under laser irradiation for efficient synergistic therapy at the tumor site. Moreover, **154-**NPs exhibited minimal obvious damage to normal organs such as the heart, liver, spleen, lung, and kidney, indicating its potential application in cancer therapy (Figure 89).

Furthermore, strategies that combine drug and photothermal/photodynamic properties have been developed. Lin et al. developed a multifunctional BAC prodrug that combines the chemotherapeutic drug camptothecin (CPT) and the fluorescent photothermal agent BODIPY through hypoxia-responsive azobenzene linkers.^447^ This prodrug was then encapsulated in human serum albumin to generate nanoparticles (HSA@BAC) to enhance solubility and tumor accumulation. In hypoxic cancer cells, overexpressed azoreductase reduces the BAC, releasing CPT for effective chemotherapy. When irradiated with a 730 nm laser, the HSA@BAC nanoparticles generate hyperthermia through oxygen-independent PTT, achieving irreversible cancer cell death. Moreover, Zhao et al. developed NP1, a nanovesicle composed of a hypoxia-responsive conjugated polymer (P1), polymetric H_2_S donor (P2), and near-infrared light-harvesting aza-BODIPY dye (B1) for delivering H_2_S and synergistic H_2_S gas therapy/PTT.^448^ The scaffold of NP1 decomposes in hypoxic environments, triggering the hydrolysis of P2 to continuously release H_2_S. B1 exhibits high photothermal conversion efficiency and superior photothermal ability under NIR light irradiation, which can inhibit the expression of cytochrome *c* oxidase (COX IV), cut off the generation of ATP, and inhibit mitochondrial respiration. This enhancement improves the antitumor efficacy of H_2_S gas therapy/PTT in hypoxic environments.

#### 2.3.3 Tumor-Specific Enzyme and Other Biomolecules Activated Theranostic Probes in PTT

**Figure 90:**
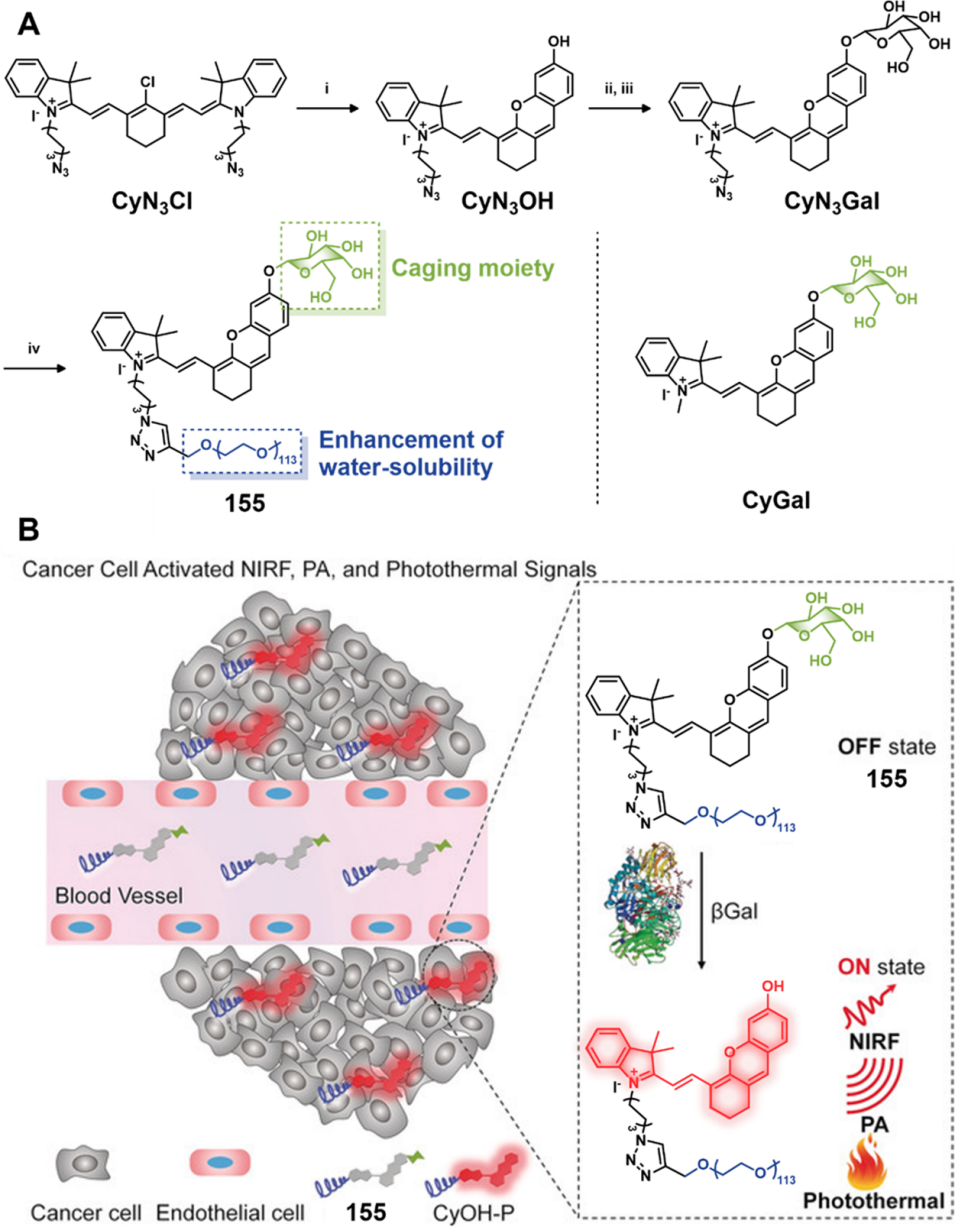
(A) The synthetic steps of **155**. (B) Schematic diagram of the activation mechanism of **155** in the tumor. Reproduced with permission from ref (450). Copyright 2018 Wiley Intersciences.

Enzymes as important biomarkers are often used to develop diagnostic and therapeutic tools for tumors. γ-Glutamyl transpeptidase (GGT) is a biomarker that is significantly upregulated in tumor tissues. Liu et al. reported a GGT-responsive near-infrared nanoprobe (NRH-G-NPs) that, through an amide bond couple γ-glutamate (γ-Glu) and cyanine fluorophore (NRH-NH_2_),^449^ the nanoprobe (NRH-G-NPs) was converted to NRH-NH_2_-NPs under the specific reaction with GGT, and the activation product NRH-NH_2_-NPs exhibited a 180-fold fluorescence signal enhancement and excellent photothermal therapeutic properties. Furthermore, this research found that β-galactosidase was overexpressed in ovarian cancers. In 2018, Pu et al. developed an activatable macrotheranostic probe (CyGal-P, **155**) that was composed of a d-galactose-caged NIR hemicyanine dye (CyOH) linked with a long poly(ethylene glycol) (PEG) chain,^450^ which released its near-infrared fluorescence (NIRF), photoacoustic (PA), and photothermal signals by β-galactosidase enzyme activation, realizing enzyme-activated imaging-guided PTT providing a new direction for the development of β-gal-activated theranostic agents (Figure 90).

**Figure 91:**
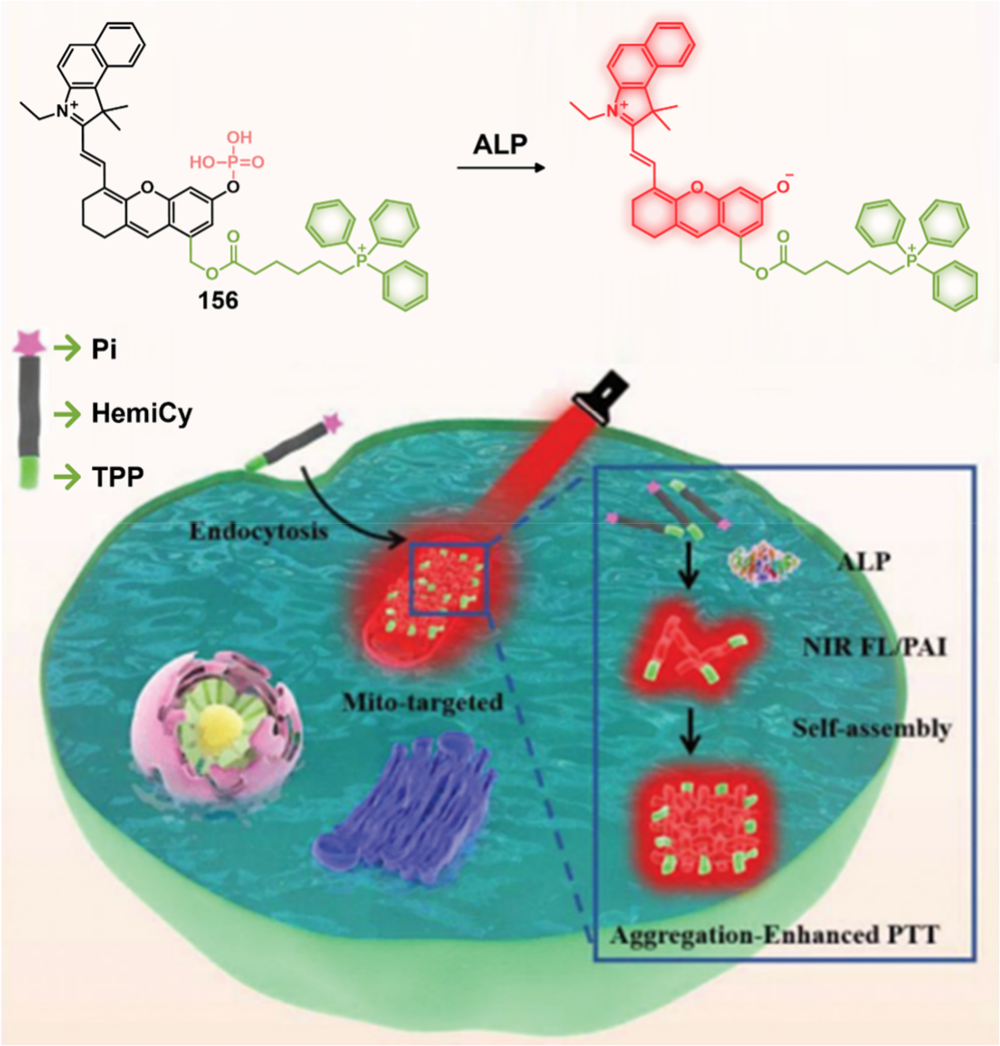
Chemical structure and molecular mechanism of probe **156** in tumors. Reproduced with permission from ref (451). Copyright 2019 Royal Society of Chemistry.

Similar studies have found that alkaline phosphatase (ALP) is overexpressed in metastatic prostate cancer. Yang et al. developed a mitochondria-targeting probe **156** that was activated by ALP and provided NIR FL/PA signals for imaging ALP activity, which undergoes simultaneously in situ self-assembly into a supramolecular structure, enhancing the photothermal therapeutic efficiency toward prostate cancer.^451^ As such accumulation enzyme activation, fluorescence/photoacoustic imaging synergistic photothermal therapeutic properties provide more scope for practical applications as diagnostic probes (Figure 91).

**Figure 92:**
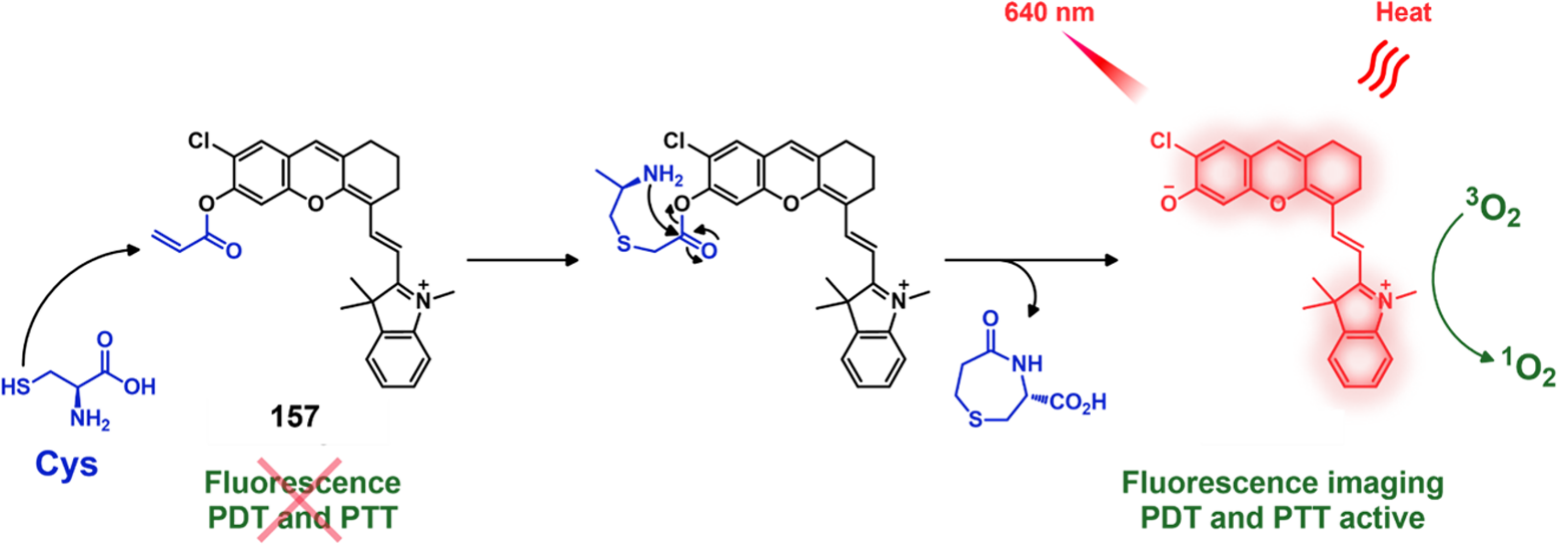
Molecular mechanism of the interaction between **157** and Cys, which results in NIRF and “Turn-On” PPT/PDT.

In addition to enzymes, many biomolecules are important activating molecules to develop diagnostic tools, e.g., cysteine (Cys), hydrogen sulfide (H_2_S), and GSH. Clinical studies have shown that high concentrations of Cys exist in tumor cells and play an important role in the balance of redox homeostasis. Kolemen et al. introduced a cysteine (Cys)-activatable chlorinated hemicyanine probe **157** that used chlorinated hemicyanine as the fluorescent core and an acrylate unit attached to the core as a cysteine recognition group.^452^ Upon reacting with Cys, Cl-Cys turns on its near-infrared fluorescence signal and activates its ^1^O_2_ generation as well as photothermal conversion potential. This study is the first report of organic small molecules with integrated photothermal and photodynamic diagnostic properties based on Cys activation. It provides a research direction for the design of biomolecular-activated diagnosis and treatment probes in tumors (Figure 92).

**Figure 93:**
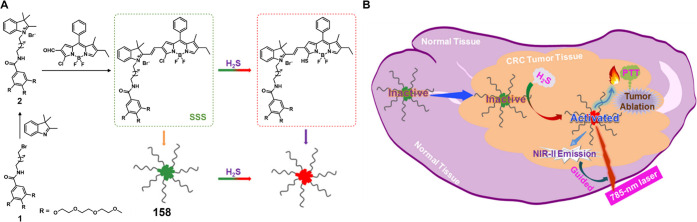
(A) Synthesis of H_2_S-responsive SSS and self-assembly process of **158**. (B) Schematic diagram of **158** NPs activation in tumor for NIR fluorescence-guided PTT. Reproduced with permission from ref (453). Copyright 2018 American Chemical Society.

Moreover, H_2_S is a potential pharmacological target for the imaging-guided treatment of colorectal cancer (CRC). Recognizing this, Zhao et al. developed a hydrogen sulfide (H_2_S)-activatable nanostructured photothermal agent (**158**) composed of a benzene ring with three PEG chains that was connected to a hemicyanine as a hydrophilic tail and a monochlorinated BODIPY core as an activatable unit.^453^ under solution conditions, the probe self-assembles to form nanostructured complexes (**158** NPs) due to hydrophilic and hydrophobic properties. In the presence of H_2_S, **158** NPs produced efficient photothermal conversion under 790 nm laser irradiation and successfully achieved effective photothermal ablation of colorectal cancer under image guidance (Figure 93).

**Figure 94:**
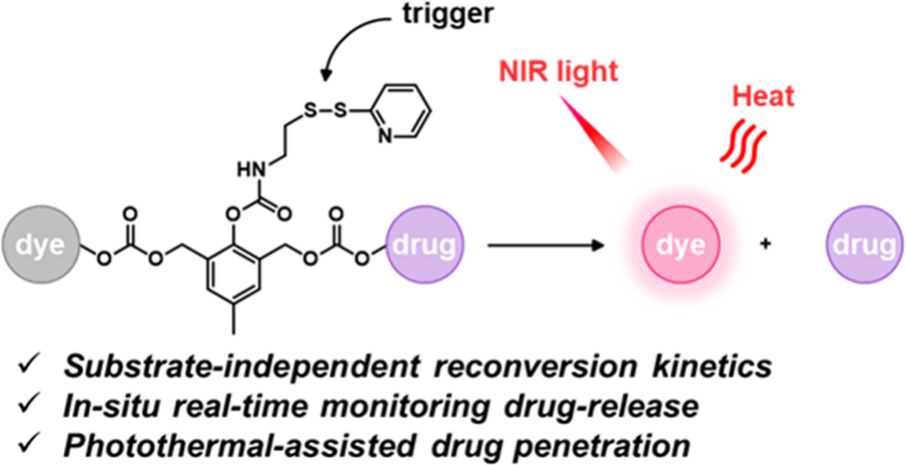
Schematic diagram of the molecular mechanism of drug release, fluorescence activation, and PTT activation strategy in response to GSH.

Similarly, GSH is a characteristic biomolecule found in tumor cells. As such, numerous GSH-triggered “Turn-On” theranostic systems have been developed. In 2018, Jiang et al. developed a self-immolative drug-dye conjugated (DDC) prodrug system that was GSH-responsive,^454^ which utilized 2-hydroxy-5-methyl-1,3-phenylenedimethanol as its core with carbonate linkages coupling fluorescent dyes and drug groups. In a tumor microenvironment, the disulfide bond was cleaved by high concentrations of GSH and the drug and fluorescent dyes can be released simultaneously. The released fluorescent dyes act as photothermal therapeutic agents to facilitate the penetration of drugs, and thus enhance the therapeutic effect (Figure 94).

To increase the retention time of the therapeutic drug at the tumor site, Lan et al. reported an in situ self-assembly strategy aimed at enhancing the PTT of glioblastomas.^455^ The probe (ICG-PEP-c(RGD)fk) was constructed by GSH-reactive self-assembling polypeptides as the skeleton, coupled to indocyanine green (ICG) as a theranostic agent and cyclic Arg-Gly-Asp [c(RGD)fk] peptides as the targeting groups. In the glioblastomas microenvironment, the disulfide bond was cleaved by GSH, while GSH-reactive self-assembling peptides increased the diagnosis and treatment system retention time and improved the photothermal therapeutic effect. Moreover, multimodal imaging synergistic therapy systems based on GSH-responsive systems have also been developed. Yang et al. reported a novel GSH-responsive theranostic nanoparticle for dual-modal imaging and PTT,^456^ the nanoparticle consisted of a disulfide-bond-linked hydroxyethyl starch paclitaxel (PTX) conjugate (HES-SS-PTX) and a near-infrared (NIR) cyanine fluorophore DiR. The conjugated PTX and loaded DiR were released when the disulfide bond was cleaved by GSH, and the released PTX could exert its photothermal therapeutic effect.

### 2.4 Theranostic Fluorescence Probes in SDT

In order to improve the efficacy of cancer treatment and reduce side effects, noninvasive therapy has become an attractive approach for the treatment of malignant tumors.^457^ SDT is a promising noninvasive cancer treatment, which was first described by Umemura et al. in 1989 to be a substitute of PDT.^458^ The sonosensitizer was activated through high penetration depth ultrasonic irradiation to generate ROS that can kill cancer cells,^459^ so as to achieve SDT. It is well-established that the SDT mechanism relies on ultrasonic cavitation and thermal destruction.^459,460^ Different from traditional cancer therapies, such as chemotherapy,^461^ radiotherapy,^462^ and PDT,^463,464^ SDT has the advantages of deep penetration, low cost, convenient use, and fewer adverse reactions.^465^ However, the combination of diagnosis with real-time monitoring for the delivery and action of sonosensitizers remains a challenge. In recent years, advanced imaging technology has provided an opportunity to solve this problem. Precision medicine implemented through imaging technology can enable an accurate diagnosis, and monitor the delivery and biodistribution as well as the therapeutic response.^466^ Generally, theranostic probes with imaging function and SDT properties are loaded on nanocarriers.^467^ Therapeutic probes are initially silent. When they enter the tumor microenvironment, the therapeutic probes are activated to produce imaging signals with high signal-to-noise ratios, and high sensitivity.^468^ In order to improve the efficacy of SDT, theranostic fluorescence probes are necessary to enable an accurate clinical judgment by physicians. Small molecule drugs loaded on nanoplatforms can realize the purpose of precise tumor localization. Nanoplatforms can specifically break down/release drugs and therapeutic agents at tumor sites on demand, which not only improves the bioavailability of these drugs but also reduces damage to normal cells or tissues. Therefore, SDT-based nanoplatforms play an indispensable role in the diagnosis and treatment of tumors. In fact, the combination of SDT and imaging technology to prepare theranostic probes can provide a more effective treatment plan for achieving the ultimate goal of tumor eradication. Studies along these lines may be divided into four categories: TME-activated, hypoxia-activated, H_2_O_2_-activated , and multifactor synergistically activatable theranostic fluorescent Probestheranostic probes.

#### 2.4.1 TME-Activatable Theranostic Fluorescent Probes in SDT

The tumor microenvironment (TME) provides appropriate conditions for tumor survival and metastasis.^469^ Tumor areas usually contain antioxidants, specific enzymes,^470^ lower blood oxygen saturation, and increased acidity,^471^ which provide directions for tumor treatment. In addition, specific nanocarriers can undergo ligand exchange, decomposition, or aggregation in the TME, resulting in their accumulation within the tumor, with enhanced therapeutic effects.^472−477^

**Figure 95:**
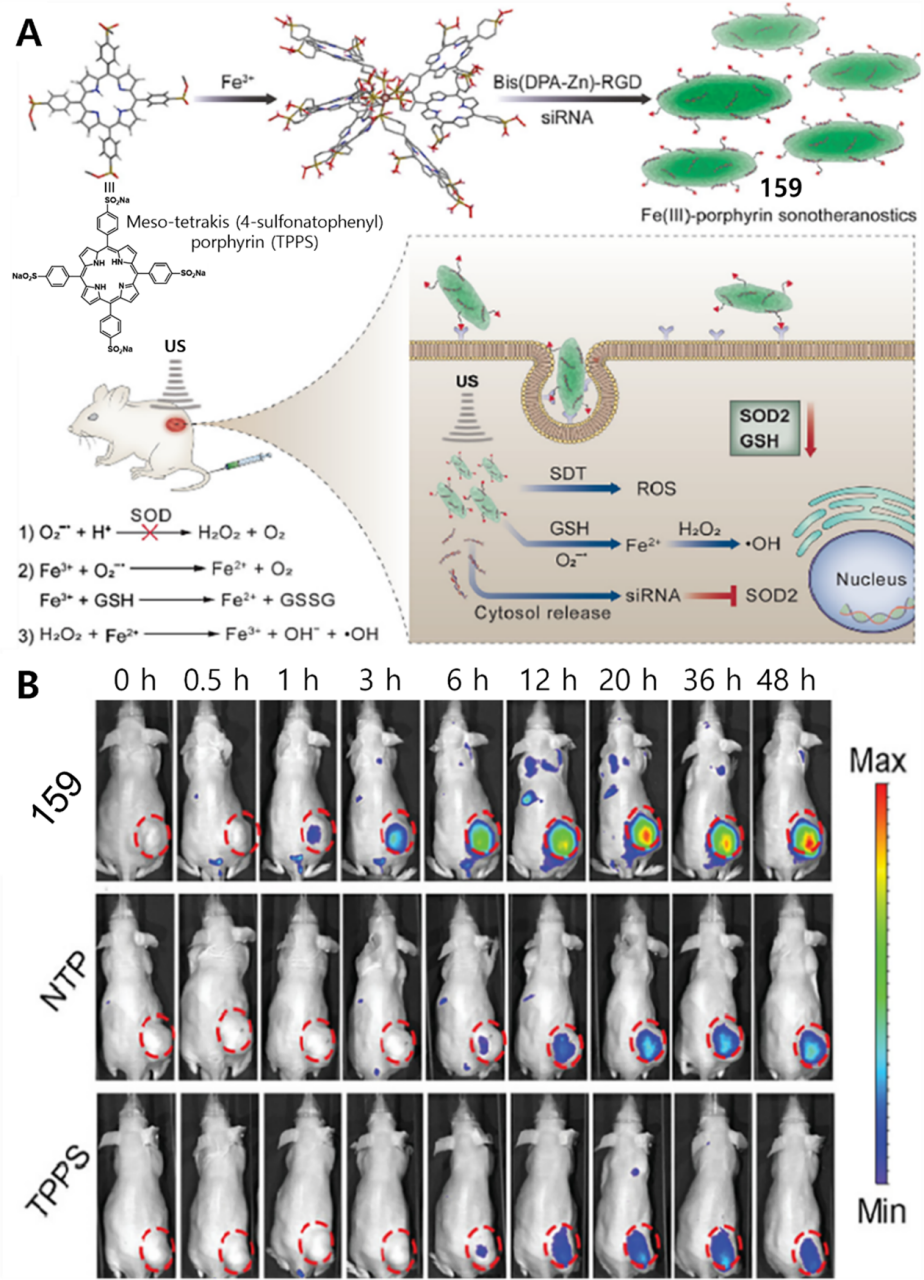
(A) Theranostic **159** as a multifunctional sonosensitizer. (B) In vivo FL images. Reproduced with permission from ref (478). Copyright 2019 Wiley Intersciences.

In general, over-expressed GSH and manganese superoxide dismutase (SOD2) play important roles in tumor cells’ resistance to ROS damage. In this regard, Zhu et al. prepared Fe(III)-porphyrin nanosensitizers (NTP) through the self-assembly of Fe(III) with meso-tetrakis (4-sulfonatophenyl) porphyrin (TPPS), followed by siRNA loading to form R-S-NTP (**159**) (Figure 95).^478^ The loaded siNTP can effectively down-regulate the expression of manganese superoxide dismutase (SOD2). The porphyrins and Fe(III) in NTP not only endow it with an admirable MR/FL imaging capability but can also effectively increase the ROS production, to improve the SDT efficiency. In addition, Fe(III) induces a cascade of bioreactions within tumor cells, which reduces the level of intracellular GSH and enables a cytotoxic Fenton reaction, leading to cancer cell death. **159** produces more ROS than RNTP, NTP, and TPPS, ascribable to a decrease of cellular antioxidant defense and the occurrence of a Fenton reaction, enhancing the efficiency of SDT. After intravenous injection, fluorescence signals were observed in tumors in the **159** group. The **159** + US group of tumor inhibition efficiency reached 89.96%, suggesting an excellent capability to prevent tumor growth. This work provides a promising approach to overcome the challenges of SDT in clinical settings, due to its excellent efficacy and novel combination of therapeutic modalities.

**Figure 96:**
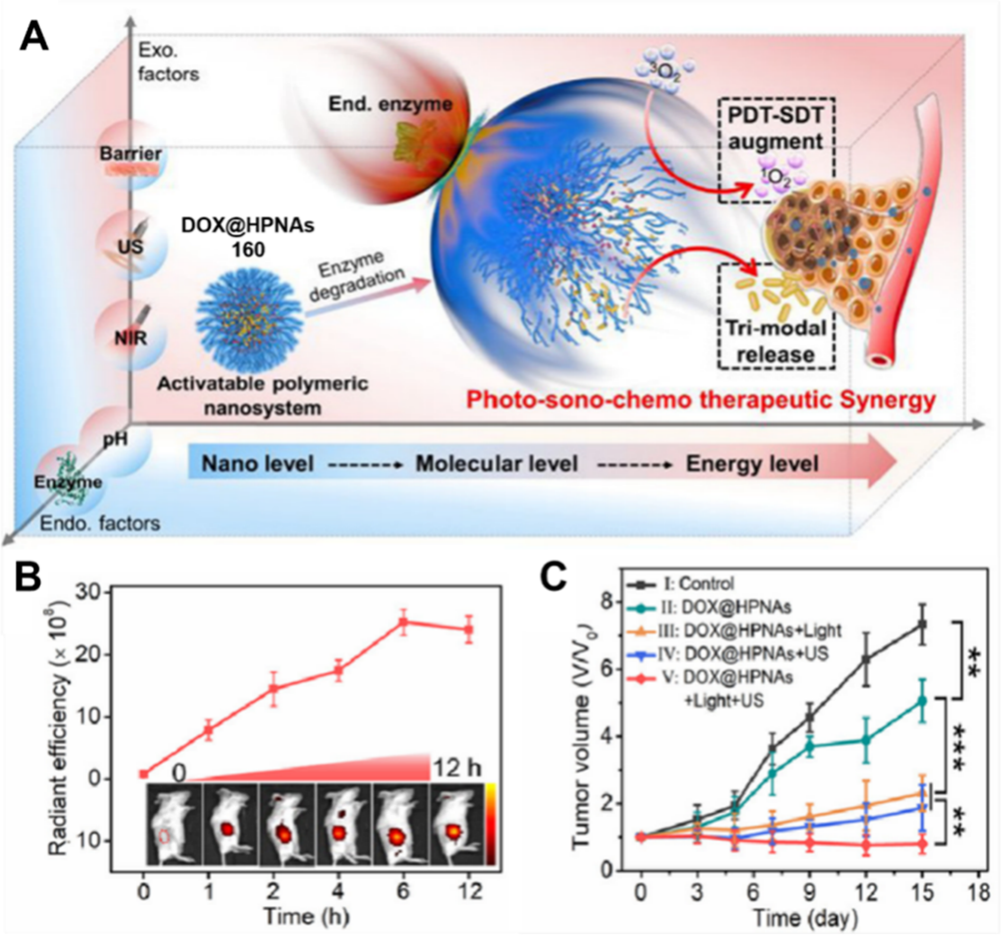
(A) Scheme illustrating the dynamic effects of endogenous (pH, HAase) and exogenous (NIR, ultrasound, and tissue barrier) factors on activatable polymeric nanosystems (**160**). (B) Time-dependent fluorescence images and intensities of tumors after the intravenous injection of DOX@HPNAs (**160**). (C) Relative growth rates of tumors after different treatments. Reproduced with permission from ref (480). Copyright 2022 American Chemical Society.

Hyaluronidase (HAase) is an enzyme that is overexpressed in tumors.^479^ As an endogenous enzyme, hyaluronidase plays a crucial role in the tumor specificity and drug release rate of theranostic molecules. In this regard, Qiu et al. fabricated a nanosystem (DOX@HPNAs, **160**) by synthesizing a negatively charged HA-PpIX nano-assembly and then loading the positively charged DOX with a 24.6% load efficiency, to achieve an effective three-mode treatment.^480^ Under acidic conditions, HAase rapidly degrades HPNA, which leads to the breakdown of nanostructures and the release of drugs. Under light and/or ultrasonic excitation, the nanosystem clustered at the tumor sites and produced fluorescence (Figure 96), and the ^1^O_2_ produced by enzymic degradation was 2.5 times that the original HPNA, showing an enhanced photoacoustic sensitization. The results showed that tumor growth was significantly inhibited in the treatment group compared to the fast-growing tumors of the control group. Histological examination of tumor sections showed that the inhibition rate of tumor growth reached 94.4% in the treatment group, indicating an excellent therapeutic effect. This work increased the Dox release efficiency through a trimodal stimulus, including acidity, ultrasonic vibration and enzyme degradation for photo-sono-chemo synergistic therapy, providing meaningful guidance for the future development of novel enzyme-activated nanosystems.

**Figure 97:**
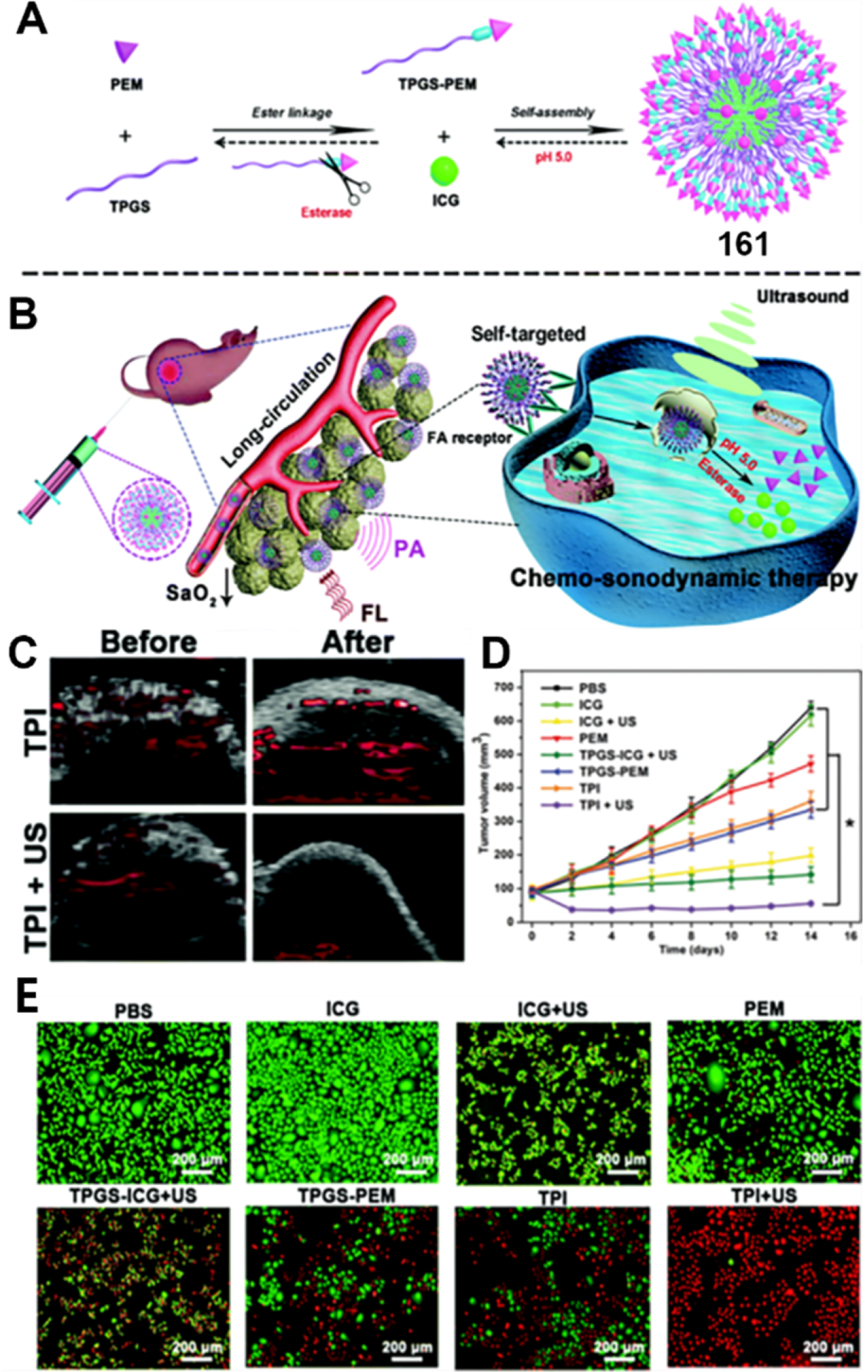
(A) TPGS-PEM prodrug and **161** self-assembly synthesis route. (B) Intensive chemotherapy and sonodynamic therapy with **161-**guided by dual imaging. (C) Changes of SaO_2_ levels in each group. (D) Changes in tumor volume after different treatments within 14 days. (E) HeLa cells stained with calcein AM/PI were incubated with different groups, and **161** (TPI) for 24 h, with or without US irradiation. Reproduced with permission from ref (482). Copyright 2021 Royal Society of Chemistry.

TME blood oxygen saturation (SaO_2_) has played a crucial role in the sustained and stable growth and development of cancer cells.^481^ To reduce SaO_2_, Yang and co-workers used a self-assembly approach to synthesize a self-targeting therapeutic nanoplatform called TPGS-PEM-ICG (TPI, **161**) for fluorescence/photoacoustic (FL/PA) imaging and enhanced chemo-sonodynamic therapy (Figure 97).^482^ The **161** nanoplatform can selectively identify tumor cells and deliver active targeted drugs on demand through multiple triggers, being the acidity of the TME, lysosomal acidity, esterases, and external ultrasound, combined this reduces the side effects on normal tissues around the tumor. In addition, PEM ligands on the surface of **161** have a strong affinity for overexpressed FA receptors on the surface of HeLa cell membranes, showing prominent tumor enrichment and cellular uptake. The excellent fluorescence signal of FA receptor-overexpressed solid tumors indicated that the nanoplatform can be used to diagnose cancer. PA imaging results showed that the SaO_2_ signal of **161** dropped sharply after US irradiation, indicating that **161** + US could achieve “starvation therapy” by reducing the SaO_2_ signal. The killing effect of **161** + US group on tumor cells was more significant than that of other groups, demonstrating a strong antitumor effect. The results of anti-tumor experiments in vivo showed that almost all tumors in the **161** + US group were eliminated, indicating that **161** + US has a spectacular anti-tumor effect. In summary, this strategy, combined with FL/PA imaging, is a good example of real-time monitoring and active targeted tumor SDT, giving guidance to future designs.

**Figure 98:**
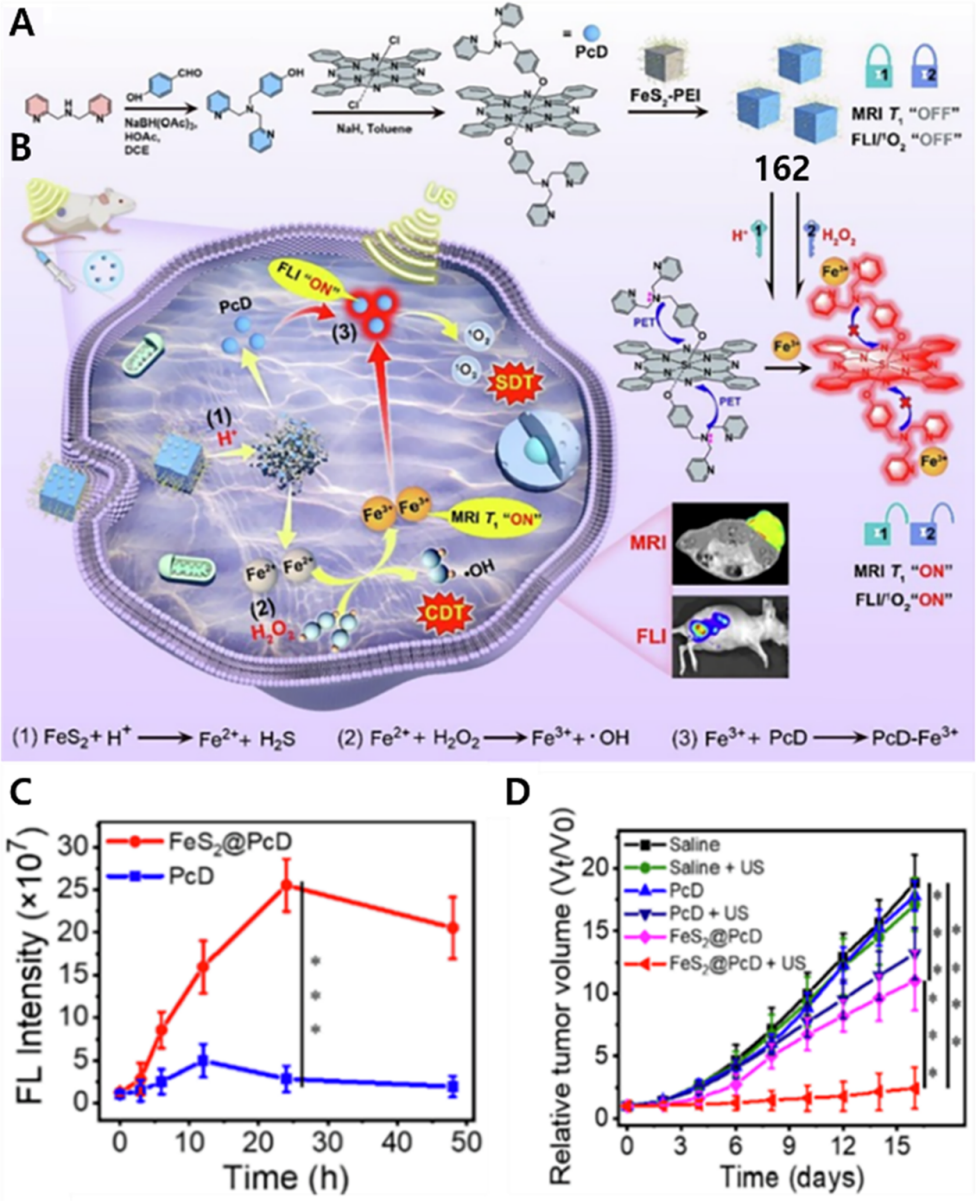
(A) Preparation and programmable mechanism of **162**. (B) The action of **162** in HepG2 hepatoma cells. (C) The mean fluorescence intensity of HepG2 tumor-bearing mice at different time points after intravenous injection of **162** and PcD. (D) Tumor growth curve. Reproduced with permission from ref (484). Copyright 2023 Elsevier Ltd.

Tumor cells prevent their acidosis by excreting excess lactic acid, causing the low pH state characterstic of the TME.^483^ On this basis, Li et al. designed a novel phthalocyanine-iron complex **162** as a theranostic nanoreactor for fluorescence/magnetic (FL/MR) resonance dual-mode image-guided SDT/CDT.^484^ In the acidic TME, **162** reacts with protons to release PcD, forming Fe^2+^, which is then further oxidized by H_2_O_2_ to form Fe^3+^, finally restoring the fluorescence of PcD (Figure 98). Through this programmable response, the tumor site of **162**-treated mice showed a strong fluorescence signal, which was 10.49 times stronger than that of PcD-treated mice, thus demonstrating that **162** causes a pronounced fluorescence imaging (FLI) signal in a tumor. It is worth noting that the magnetic resonance signal of **162** also switched from the “OFF” state to the “ON” state under this programmable regulation by acidity and H_2_O_2_. In addition, **162** combined with US irradiation had a significant inhibitory effect on the growth of tumors in mice, with an inhibition rate of 87.15%. In conclusion, **162** achieves specific fluorescence and MR dual-mode image-guided SDT/CDT and represents a promising image-guided tumor therapy approach.

#### 2.4.2 Hypoxia-Activatable Theranostic Fluorescent Probes for SDT

SDT can generate ROS to induce oxidative damage to cancer cells, and oxygen is one of the main sources of ROS. However, due to abnormal cell proliferation, vascular system abnormalities, lymphatic system dysfunction, and other reasons,^485^ solid tumors remain in a state of hypoxia for a long time, which severely limits the efficiency of SDT. Meanwhile, the rapid consumption of oxygen at the tumor sites by SDT process can also lead to the exacerbation of local hypoxia, which further inhibits SDT through a chain reaction, leading to poor therapeutic effects and poor prognosis.^486^ Hypoxia can also lead to drug resistance for chemotherapy and radiotherapy,^487^ affecting their therapeutic effectiveness. Therefore, methods to diminish the influence of hypoxia on the efficiency of SDT have become a key research area.

**Figure 99:**
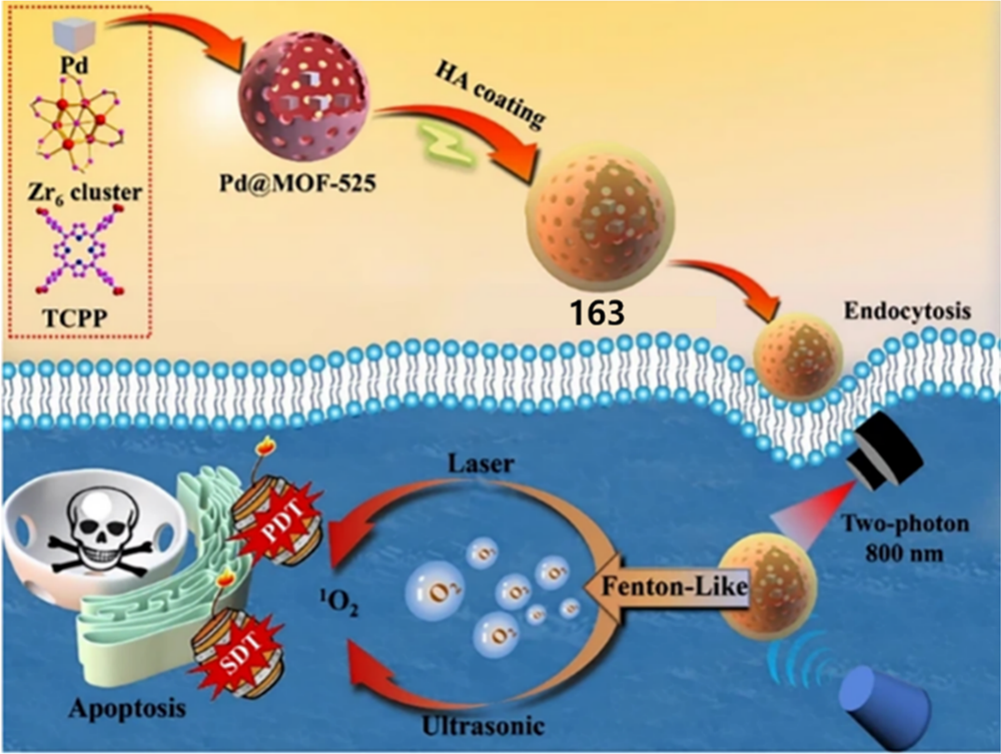
Preparation of theranostic probe **163**, highlighting the process of enhanced photodynamic and sonodynamic therapy. Reproduced with permission from ref (491). Copyright 2022 BioMed Central Ltd.

It has been shown that upregulation of O_2_ content in tumors through tumor-targeted O_2_ delivery and intratumoral oxygen production strategies are effective ways to alleviate tumor hypoxia. For example, the introduction of Fenton or Fenton-like reactions is a promising method to trigger oxygen production.^488−490^ Duan et al. developed a nanoplatform **163** involving Fenton-like reactions (Figure 99), which alleviates tumor hypoxia through this oxygen-producing strategy and improves the therapeutic effect of SDT and PDT.^491^ Among them, porphyrin MOF-525 can not only produce ^1^O_2_ for SDT and PDT but also can be used as a two-photon-responsive moiety for NIR photoinduced PDT. Pd nanocubes generate hydroxyl radicals (•OH) and O_2_ through a Fenton-like reaction, which leads to cell apoptosis and greatly alleviates tumor hypoxia. The surface modified of Pd@MOF-525 by hyaluronic acid (HA) enhances biocompatibility and provides a specific targeting ability toward cancer cells. The cell viability experiments showed that the survival rates of QSG7701 cells treated with **163** were higher than that of HepG2 cells, and the survival rate of HepG2 cells after the addition H_2_O_2_ was lower than the control group, indicating that **163** is harmless to normal cells, while •OH can be produced in cells with high H_2_O_2_ expression, resulting in decreased cell viability. A cytotoxicity evaluation revealed that the cell survival rate of HepG2 cells after light and ultrasonic irradiation was 10%, which confirms that the nanoplatform exhibits outstanding synergistic therapeutic PDT/SDT effects. This work provides a suitable approach to design novel oxygen-producing nanomaterials that can enhance oxygen-dependent antitumor treatment.

**Figure 100:**
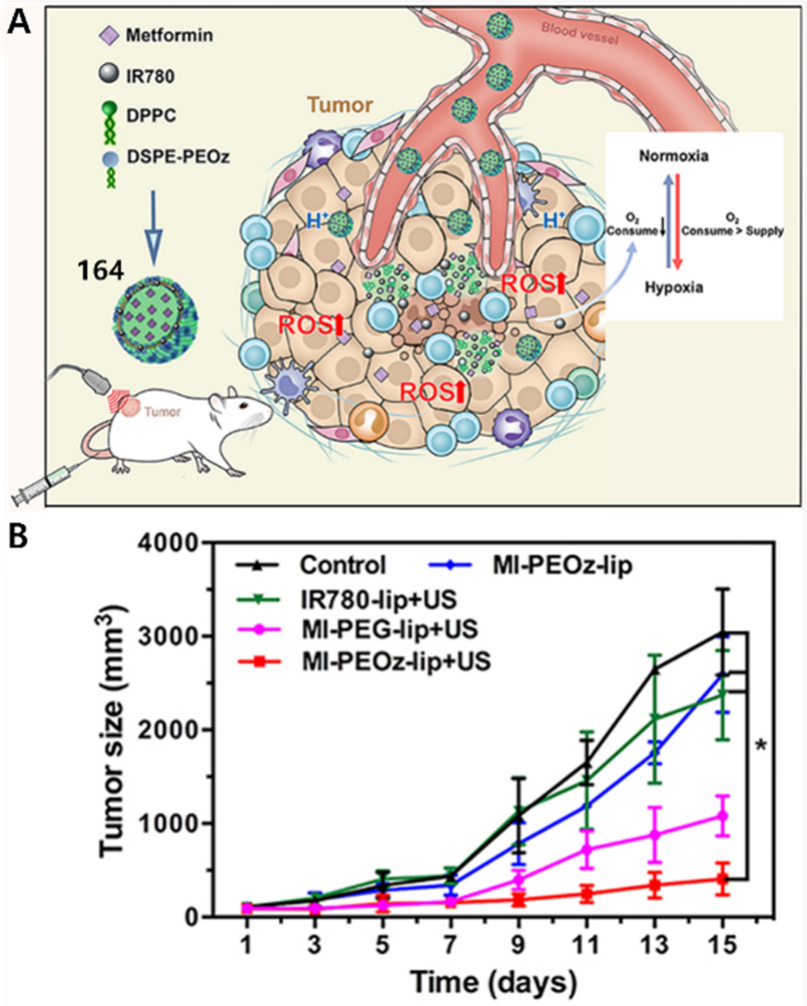
(A) Schematic illustration of self-synthesized **164** (MI-PEOz-lip) and its proposed antitumor mechanism. (B) Tumor growth curves of the five groups after receiving various treatments. Reproduced with permission from ref (501). Copyright 2020 Dove Medical Press Ltd.

Since hypoxia within the tumor can only be temporarily alleviated by oxygen production or transport to solid tumors,^492^ some problems remain such as low oxygen production efficiency or oxygen leakage during these processes.^493−497^ Therefore, reducing oxygen consumption may represent a feasible solution to replace the methods mentioned above. Since the main function of mitochondria-related oxidative phosphorylation (OXPHOS) is to generate energy by consuming oxygen,^498−500^ inhibition of OXPHOS activity can be an effective way to reduce oxygen consumption. Therefore, Zhang et al. prepared a pH-responsive drug-carrying liposome (**164**) to reduce oxygen consumption in tumor areas.^501^ Metformin molecules in **164** are released in acidic tumor tissues and selectively accumulate in tumors to inhibit the mitochondrial respiratory chain (Figure 100). While the sonosensitizer IR780 was released into the tumor areas to produce ROS for killing the cancer cells under US irradiation. In addition, intravenous **164** could effectively deliver encapsulated drugs to the hypoxic sites of tumors due to its enhanced permeability and retention (EPR) effect. Theranostic **164** exhibits excellent PA/FL imaging capabilities both in vitro and in vivo applications. The results indicated that tumor growth was significantly inhibited in the **164** + US treatment group. Notably, tumor growth was significantly retarded after treatment due to rapid drug release in the acidic microenvironment of the tumor, while rapid tumor growth remained in the control groups. This research has resulted in a nanoplatform able to overcome hypoxia-induced resistance to cancer therapy by interfering with normal energy metabolism processes to reduce oxygen consumption.

**Figure 101:**
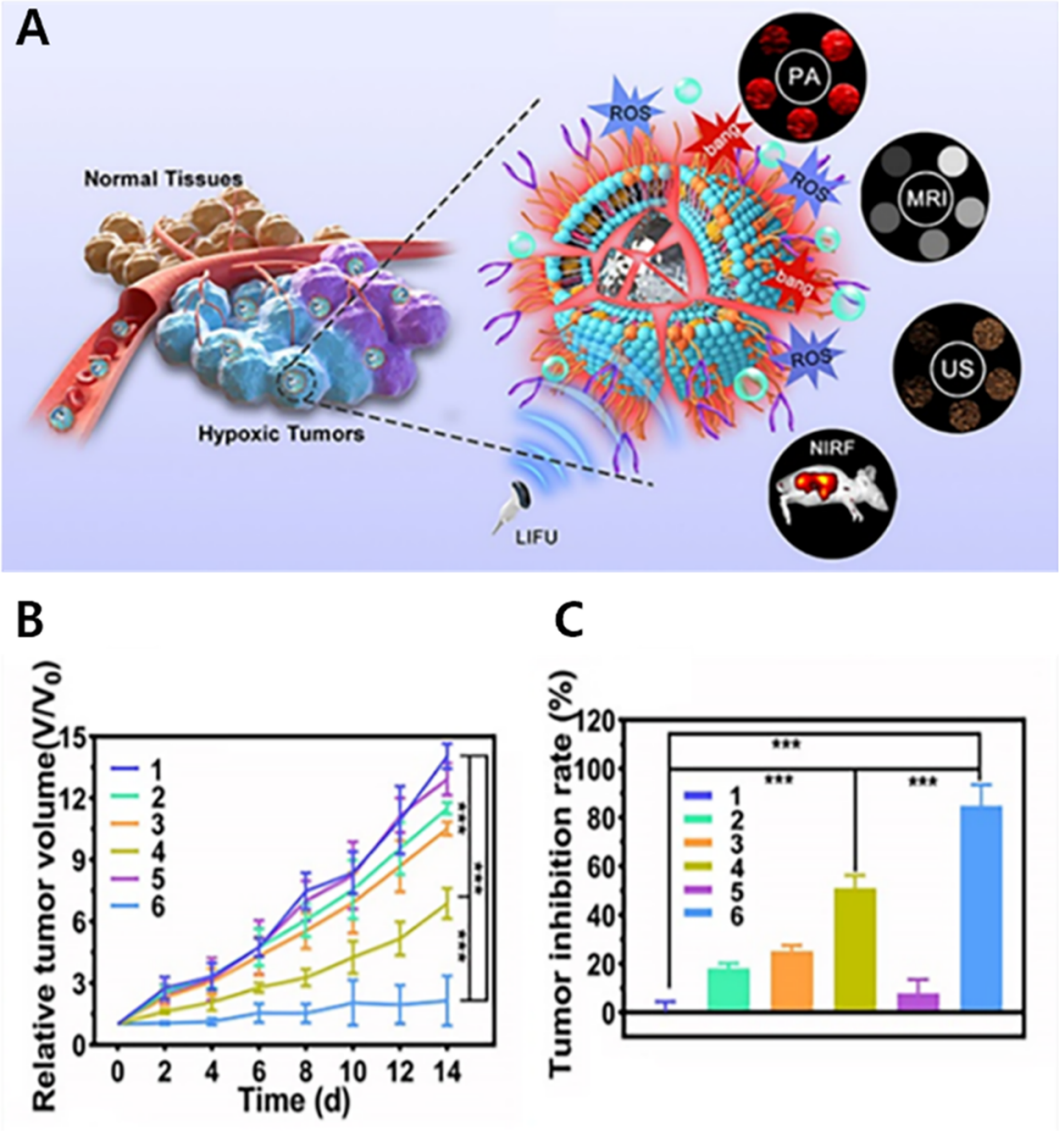
(A) The generation of ROS and ADV mechanisms for synergistic hypoxia-tolerant SDT against solid hypoxic tumors under multimodal imaging guidance. (B) Relative tumor volume changes of the mice after various treatments. (C) Tumor inhibition rate of the mice after multiple treatments. Reproduced with permission from ref (513). Copyright 2022 Dove Medical Press Ltd.

Although many methods have been developed to alleviate hypoxia by generating/transporting oxygen^502−506^ or reducing oxygen consumption within solid tumors, the therapeutic effectiveness may under some conditions be unfavorable and may even have adverse consequences, including barotrauma and hyperoxic seizures.^465^ Acoustic droplet vaporization effect induces a rapid phase-shift mode for liquid perfluoropentane (PFP) to gas phase upon ultrasonic triggering.^507^ Microvesicles can improve the ablation effects of high-intensity focused ultrasound (HIFU), reduce the required acoustic energy, and enhance tumor damage. Hence, phase change materials with similar therapeutic behavior are expected to regulate the acoustic environment of hypoxic tumors.^508,509^ Therefore, the PvP-based cavitation effect is an attractive strategy that can effectively induce anoxic tumor death without the need for oxygen. In addition, CGNKRTR (tLyP-1) is a cell-penetrating peptide used as a ligand for targeting neuroproteinase-1 receptor (np-1) and can effectively penetrate deep into tumor cells through the endocytosis/extracellular transport pathway (CendR pathway).^510−512^ Based on the above considerations, Luo and co-workers fabricated a functionalized liposome based on tLyP-1, and then loaded porphyrin monomethyl ether gadolinium (H(Gd)) as a sonosensitizer into the phospholipid bilayer to afford PFP@tLyP-1-LIP-H(Gd) (**165**).^513^ The liposome can target MDA-MB-231 tumor cells through the specific adhesion of cell-penetrating peptides to specific cells overexpressing NPR-1 and can effectively penetrate hypoxic tumors to facilitate US/NIRF/PA/MR imaging (Figure 101). Under low-intensity focused ultrasound (LIFU) irradiation, the acoustic droplet vaporization effect of perfluoropentane induces rapid “liquid-gas” transition and rapid bubble production to produce hydroxyl radicals (as deep penetrating nano-bombs, DPNB), resulting in cell death in both normoxic and hypoxic microenvironments. **165** exhibits enhanced cytotoxicity after treatment with LIFU. Tumor inhibition rates confirmed that acoustic droplet vaporization combined with SDT inhibited tumor growth. This study was the first to report oxygen-independent SDT based on ultrasonic cavitation effects and “liquid-gas” conversion.

#### 2.4.3 H_2_O_2_-Activatable Theranostic Fluorescent Probes in SDT

ROS include H_2_O_2_, ozone (O_3_), hypochlorous acid/hypochlorite (HOCl/ClO^–^), hydroxyl radical (•OH), nitric oxide (NO), and peroxynitrite (ONOO^–^).^507^ Among them, H_2_O_2_ is one of the most important species. It is produced in the mitochondria, mainly through the activation of the nicotinamide adenine phosphate dinucleotide oxidase complex (NADPH),^514^ which is an important signaling molecule for cell growth, proliferation, and differentiation.^515^ Increased concentrations of H_2_O_2_ at tumor sites are closely related to tumor cell growth, development, and apoptosis. In addition, the intrinsic enhancement of H_2_O_2_ levels in tumor cells induces the expression of metastasis-related growth factors, leading to invasion and migration,^516,517^ which is one of the most important causes of cancer death. Therefore, it has been evaluated as an important target for the design of novel anti-tumor strategies.^518^ However, currently, no theranostic fluorescent probes activated by H_2_O_2_ for SDT have been reported, meaning that more research is urgently needed in this area.

#### 2.4.4 Multifactor Synergistically Activatable Theranostic Fluorescent Probes in SDT

**Figure 102:**
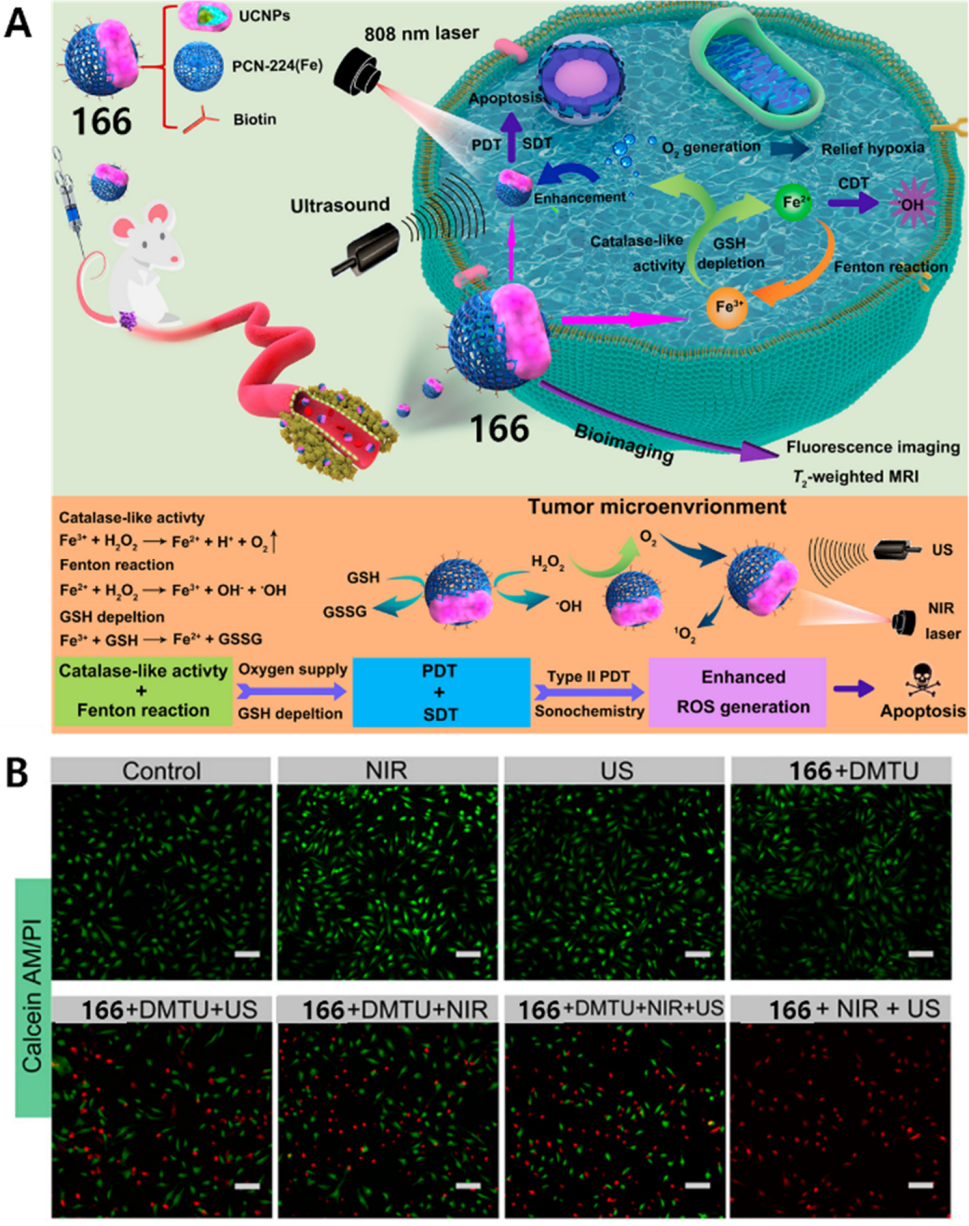
(A) Schematic Illustration of Antitumor Mechanism of **166**. (B) The detection of live/dead cells after various treatments. Live and dead cells were stained with calcein-AM (green) and PI (red), respectively. Reproduced with permission from ref (519). Copyright 2021 American Chemical Society.

Severe hypoxia, GSH over-expression, and high concentrations of H_2_O_2_ at tumor sites restrict the therapeutic effect of ROS in PDT, chemodynamic therapy (CDT), and SDT.^518^ To address this problem, Wang et al. designed a combination strategy consisting of precision-guided imaging, GSH consumption, targeting, and catalase activity to construct a bio-catalyzed Janus nanocomposite (**166**) based on an iron-based zirconium porphyrin metal–organic framework [PN-224 (Fe)], mediated by near-infrared (NIR) light and US (Figure 102).^519^ Fe^3+^ acts synergically in **166** as a catalase-like nanozyme, which not only catalyzes H_2_O_2_ to produce O_2_, relieving the TME hypoxia but also consumes excess intracellular GSH and promotes ROS generation. Fe^2+^ reacts with intracellular H_2_O_2_ to produce toxic •OH, which improves the CDT performance. **166** exhibits PDT properties when it is excited by 808 nm laser irradiation at high H_2_O_2_ concentrations. Synchronous activation using US achieved an additional SDT effect and an enhanced fluorescence signal. The results of cell activity evaluations indicated that the **166**+NIR+US group exhibited a high degree of apoptosis in tumor cells. Therefore, using a combination of multiple tumor sites characteristics and imaging guidance provides a promising strategy for improving the therapeutic efficacy of SDT, PDT, and CDT.

**Figure 103:**
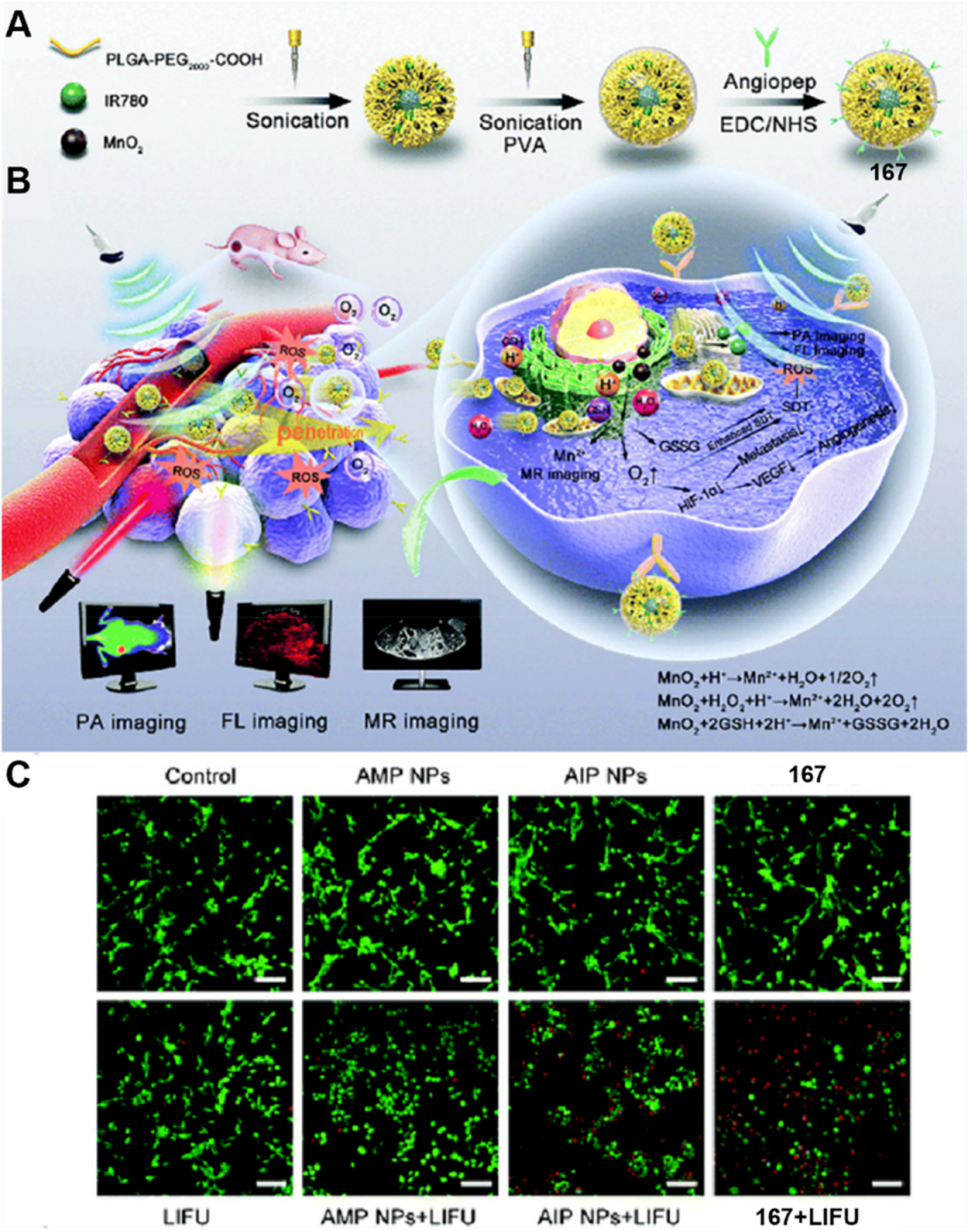
(A) Schematic illustration of **167** NPs synthesis. (B) Schematic illustration of **167** NPs with BBB and tumor targeting, mitochondrial targeting, deep penetration, enhanced SDT effect, and real-time PA/FL/MR imaging monitoring. (C) CLSM images of live/dead cells after various treatments. Reproduced with permission from ref (520). Copyright 2021 Royal Society of Chemistry.

Later, Liu and co-workers used the acidic TME to develop a multimodal (PA/FL/MR) image-guided multifunctional nanozyme AIMP NPs (**167**) by encapsulating IR780 and MnO_2_ within PLGA/Angiopep-2 to enhance SDT (Figure 103).^520^ Theranostic probe **167** can easily penetrate the blood–brain barrier (BBB) and target gliomas due to Angiopep-2. MnO_2_ is known to exhibit enzyme-like activity, it can react with high levels of protons, H_2_O_2,_ and GSH in the cancer TME to generate oxygen and degrade GSH. In addition, Mn^2+^ is used as an MRI contrast agent, and IR780 has PA/FL imaging capabilities enabling FL/PA/MR imaging-guided cancer therapy. After intravenous injection, fluorescence from Fe-TCPP in **167** was observed at tumor sites, indicating that biotin-modified **167** accumulates in the tumor area through the EPR effect. Cell viability tests indicated that SDT induced apoptosis of tumor cells under LIFU irradiation. Therefore, this work, using multiple mechanisms to maximize the SDT effect in U87MG xenografts, was able to significantly inhibit tumor growth and distal metastasis.

### 2.5 Theranostic Fluorescent Probes in Immunotherapy

In 1891, William Coley accidentally discovered that postoperative pyogenic streptococcal infections led to tumor regression in sarcoma patients, which was a prelude to tumor immunotherapy and has been a hotspot for the treatment of tumors ever since.^521^ Compared with traditional cancer therapies such as chemotherapy and radiotherapy, immunotherapy has the advantages of high selectivity, few side effects, and good efficacy, and has become a promising approach for clinical applications.^522−524^ Nowadays, cancer immunotherapies mainly include immune checkpoint therapy, adoptive cell therapy, and vaccines. Immune checkpoint blockade therapies based on CTLA-4 and PD-1/PD-L1 have good efficacy.^525^ Yet nearly 70% of patients do not respond positively to immune checkpoint inhibitors (ICIs). Besides, some adverse drug reactions may even lead to death.^526−528^ To address those problems and improve immunotherapy effects, researchers are beginning to explor combination therapies.

For example, some theranostic fluorescent probes with the capability of real-time imaging and immunotherapy have been developed by some researchers. These theranostic fluorescent probes with small molecule drugs can generate synergistic effects with checkpoint blockers to improve immunotherapeutic efficacy. In addition, due to the excellent penetration and appropriate half-life,^529^ theranostic fluorescence probes can provide real-time imaging for clinical diagnostics. Significantly, clinicians can obtain in vivo information rapidly and accurately using small molecule-based probes. As such, small molecule drugs are are appealing for preparing theranostic fluorescence probes for combination strategies.^530,531^ It has been reported that the metabolic state of the tumor microenvironment (TME) is essential to tumor immunotherapy.^532^ There are some typical characteristics for immune-related targets of the TME including H_2_O_2_ overexpression, aberrant physicochemical properties, and hypoxia.^533^ Taking advantage of these features, a diversity of theranostic probes have been developed toward the TME based on differences in pH,^534^ GSH,^535^ and H_2_O_2_^536^ concentrations as well as hypoxia.^537,538^ Such highly sensitive probes are suitable for the imaging of tumors, and provide valuable data for advanced analysis. This section summarizes the state-of-the-art of theranostic fluorescent probes based on TME, H_2_O_2_, and hypoxia-based activation.

#### 2.5.1 TME-Activatable Theranostic Fluorescent Probes in Immunotherapy

The TME is a complex system that contains tumor cells, immune cells, lymphovascular cells, and various metabolites.^539,540^ The metabolic state of the TME affects tumor immunity through multiple mechanisms.^532^ Immune cells can interact with tumor cells to activate an immune response or an immune tolerance.^541^ When persistent tumor antigen stimulation and immune activation responses are elicited, the relevant effector cells in the microenvironment are depleted or remodeled. As a consequence, the relevant effector cells cannot exert normal function and this can promote the malignant characteristics of tumors. Immunosuppressive TME largely reduces immunotherapy efficacy due to its limitation in recruiting and activating T cells.^542^ For example, immune checkpoint inhibitor (ICI) immunotherapy exhibits good effects in clinical cancer therapy,^543^ but relies on an immunosuppressive TME. Therefore, only 30% of patients demonstrate satisfactory ICI treatment outcomes.^528,544^ As such, mono-modal immunotherapy loses its advantage, and it becomes necessary to improve the immunosuppressive TME and boost immunotherapeutic efficacy. For this reason, immunotherapy has been combined with small molecule-based fluorescent probes to generate synergistic effects, which can not only increase immune cell activity but also induce PTT and PDT for adjuvant therapy.^545^ Real-time tumor imaging by theranostic probes can help clinicians monitor disease states in vivo and provide valuable treatment information. Therefore, TME-activated theranostic probes represent promising approaches for improved immunotherapy.

**Figure 104:**
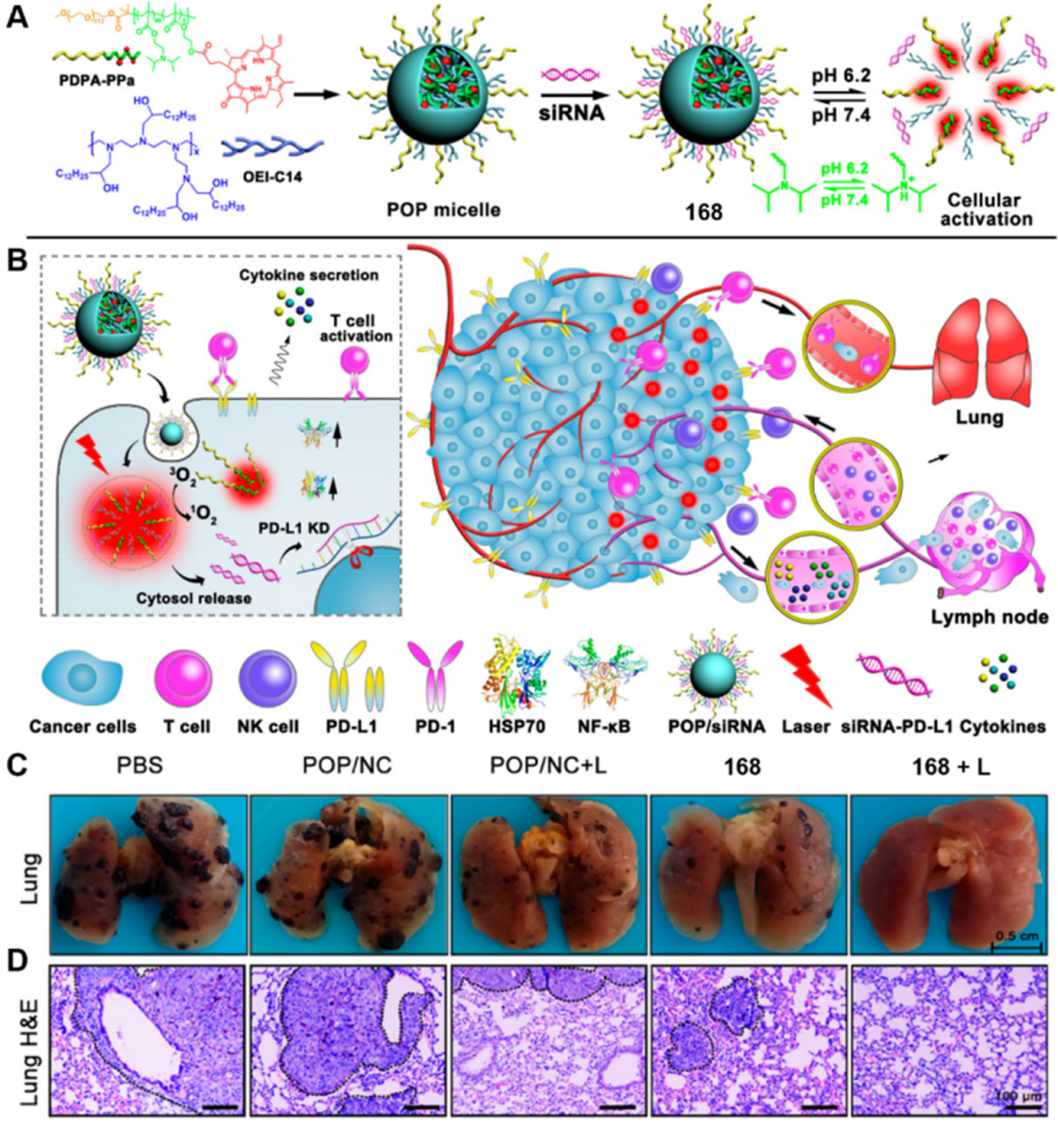
(A) Chemical structure of the acid-activatable **168**. (B) Schematic representation of **168**-mediated photodynamic cancer immunotherapy. (C) Photographs and (D) H&E staining of the metastatic foci of the B16-F10 tumors. Reproduced with permission from ref (547). Copyright 2016 American Chemical Society.

Over the past several decades, adaptive immune resistance has been identified as a hallmark of tumor development.^546^ Programmed cell death receptor 1 (PD-1) and programmed death 1 ligand (PDL1, B7-H1) are two crucial immune checkpoint molecules associated with immune resistance. PD-L1 is significantly expressed in many tumor cells, such as melanoma, breast, and renal cell carcinoma. In cancer cells, PD-L1 can bind with PD-1 of T cells to suppress the immune response and reduce immunotherapeutic efficacy.^523^ To improve immunotherapy, antibodies have been used to block PD-L1/PD-1 binding, and this strategy has resulted in significantly enhanced cancer treatment efficiencies. Taking low pH-activated drugs as a precursor, Li et al. combined these drugs with immune checkpoint blockade to develop a theranostic probe.^547^ Li and co-workers synthesized acid-activated multifunctional micelleplexes (**168**) that consist of the pheophorbide A (PPa) PS, PD-L1-specific siRNA, and the pH-responsive PDPA (Figure 104). These micelles are non-fluorescent and do not exhibit phototoxicity in normal physiological pH environments due to fluorescence resonance energy transfer from PPa. Under acidic TME conditions (pH < 6.2), PPa in these micelles was activated to generate fluorescence signals for real-time imaging. Moreover, the activated PPa promotes ROS production to stimulate an antitumor immune response. Additionally, siRNA was released to block the PD-1/PD-L1 pathway. After intravenous injection, fluorescence signals were observed in the **168** + laser treatment group. In sharp contrast to the control group, the concentration of TNF-α and IFN-γ in the micelles + laser group dramatically increased, suggesting the immune response has been greatly enhanced. In the **168** + laser group, tumors were eliminated, and subcutaneously re-challenging these mice with the same tumor cells failed to result in tumor growth in 75% of these mice. This result indicates that this acidic TME-activated drug platform can lead to excellent anticancer immune efficacy.

**Figure 105:**
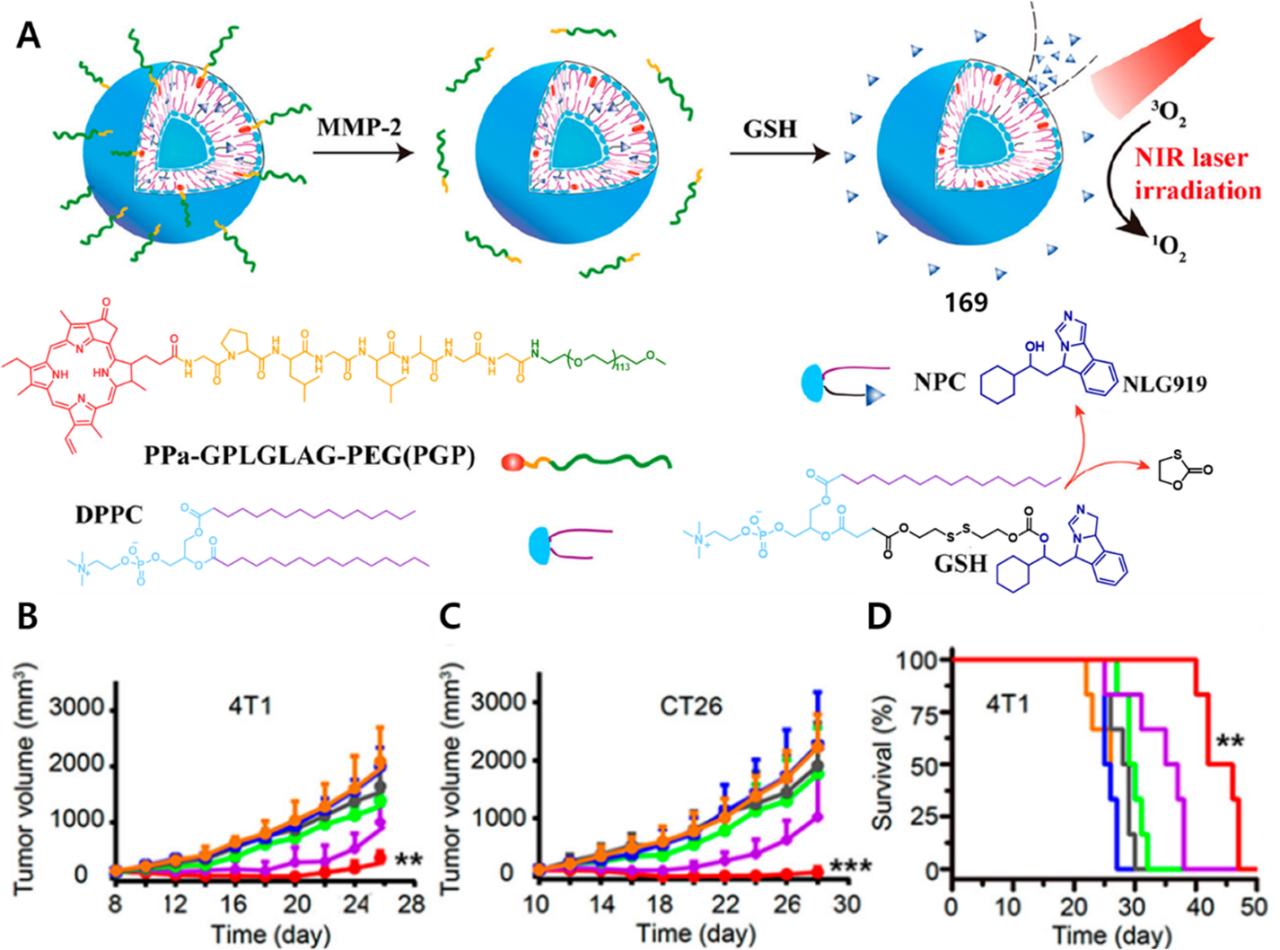
(A) Schematic illustration of the MMP-2-sheddable and GSH-activatable prodrug vesicle **169**. (B) Tumor growth curves of the 4T1 tumor model and (C) the CT26 tumor model. (D) Survival curves of 4T1-tumor-bearing mice. Reproduced with permission from ref (550). Copyright 2019 American Chemical Society.

GSH is a reductive tripeptide with a cellular antioxidative function;^488^ the conversion between GSH and its oxidated GSSG form can regulate cell redox environments.^548^ In the TME, GSH over-expression leads to a reductive environment that could be utilized for reduction-activated theranostic probes.^549^ Yu et al. designed a GSH-activated prodrug **169** that consists of a PS (PPa), an inhibitor (NLG919) of IDO-1 that is activated under reductive conditions, and a PEG corona.^550^ IDO-1 is an endogenous immune regulator, which can induce apoptosis in T cells, thus triggering the adaptive immune resistance response and reducing the efficacy of immunotherapy. NLG919 could effectively reduce IDO-1 activity to enhance the intratumoral infiltration of CTLs. **169** was silent in normal physiological environments. Since the PEG corona has good cell penetration, bio-compatibility and targeting ability, the prodrug specifically accumulated in tumors. In the reductive TME, **169** was activated by GSH and generated fluorescence signals for real-time imaging. NLG919 was released from **169** and suppressed IDO-1, to eliminate the adaptive immune resistance (Figure 105). In addition, under near-infrared irradiation (NIR 671 nm), PPa promoted ROS production to induce immunogenetic cell death (ICD). In the **169** + laser irradiation treatment group, the flow cytometry results showed that the maturation ratio of DCs was 1.7-fold higher than the control groups, and the intratumor invasion of IFN-γ and Tc was also significantly increased. These observations suggest that the immunotherapy efficacy was largely improved by this self-assembled system.

**Figure 106:**
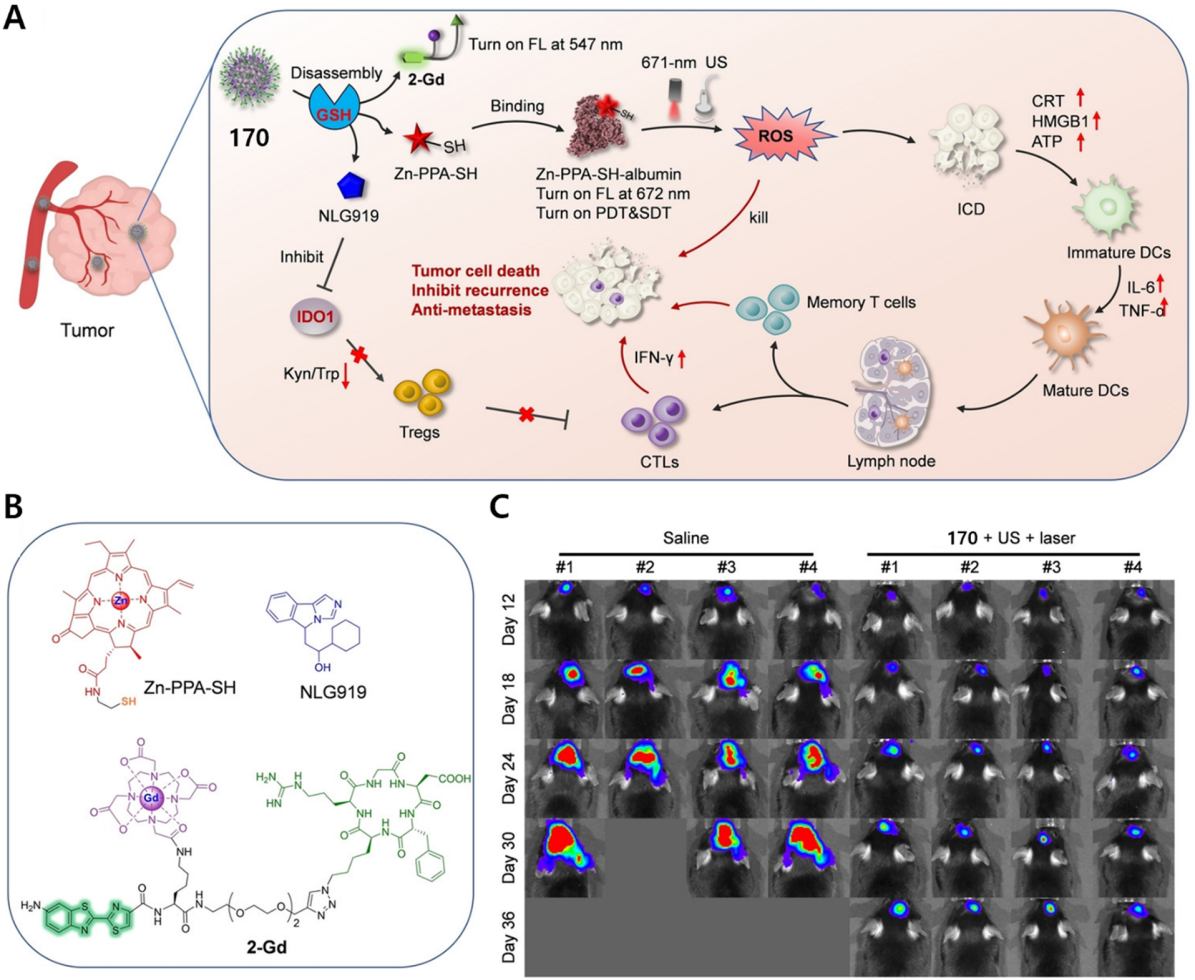
(A) Schematic mechanism of **170** for FL and MR bimodal imaging-guided sono-photodynamic immunotherapy of tumors. (B) The chemical structures of released 2-Gd, NLG919, and Zn-PPA-SH. (C) BL images in mice with **170**. Reproduced with permission from ref (551). Copyright 2023 Wiley Intersciences.

To achieve imaging-guided sono-photodynamic immunotherapy, Ye et al. synthesized a tumor-targeting and GSH-activated NIR Zn-chelated pheophorbide probe (Zn-PPA-SH), and then co-assembled it with the IDO1 inhibitor NLG919 and a hydrophilic Gd-DOTA chelate (2-Gd) for MRI to fabricate nanosensitizer **170**.^551^ Zn-PPA-SH can generate ROS under laser irradiation to improve both the efficiency of PDT and immunotherapy. Additionally, Zn-PPA-SH can produce a NIR fluorescence “Turn-On” signal at 672 nm for real-time imaging. After administration into tumor bearing mice, **170** can effectively penetrate tumor sites by cRGD-mediated delivery, which could be tracked through T1-weighted MR imaging. In the TME, a high concentration of GSH can reduce the disulfides of **170**. Consequently, Zn-PPA-SH and NLG919 are released (Figure 106). At 8 h post-injection, intense NIR fluorescence signals appear in the tumor areas, which exhibited 6.5-fold higher fluorescence than the control groups. The treatment group that was administered **170** in addition to US and laser irradiation exhibited prolonged survival, which extended to 54 days. In sharp contrast, all mice in the saline-treated group failed to survive more than 32 days. These results imply that GSH-activated **170** can improve the efficacy of cancer therapy.

#### 2.5.2 H_2_O_2_-Activatable Theranostic Fluorescent Probes in Immunotherapy

**Figure 107:**
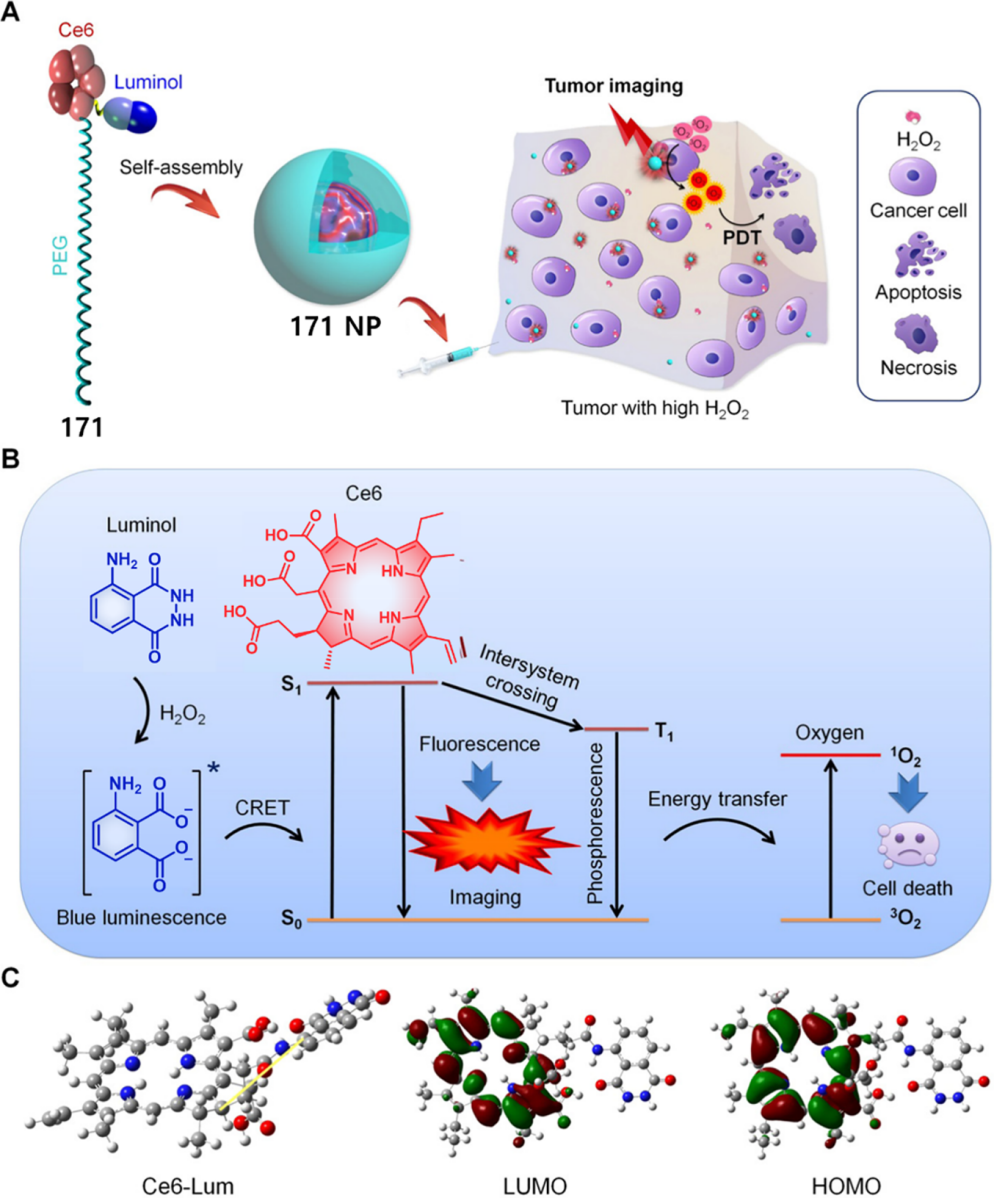
Design of a luminescent nanoprobe **171** NPs for imaging and treating tumors expressing high H_2_O_2_. Reproduced with permission from ref (552). Copyright 2020 American Chemical Society.

Zhang et al. developed self-illuminating nanoparticles **171** by conjugating a PS Ce6 (an amphiphilic conjugate of chlorin e6) with luminol and PEG (Figure 107).^552^**171** can simultaneously achieve diagnosis and therapy by virtue of chemiluminescence resonance energy transfer (CRET) between luminol and Ce6. In the presence of H_2_O_2_, the luminol unit is oxidized by H_2_O_2_ to emit blue luminescence. Due to CRET, luminol can generate intense fluorescence signals that correspond linearly to the H_2_O_2_ concentration. In addition, **171** can generate ^1^O_2_ for PDT and immunotherapy through CRET-mediated in situ excitation of Ce6. Animal experiments were performed to investigate the theranostic effect of **171**. After subcutaneous injection, clear luminescent signals appeared in the tumor areas. Moreover, the generation of ^1^O_2_ was sustained for approximately 8 ht. **171** was subject to a dose-dependent therapeutic efficacy, with the most effective result achieved at 3.25 mg/kg Ce6.

**Figure 108:**
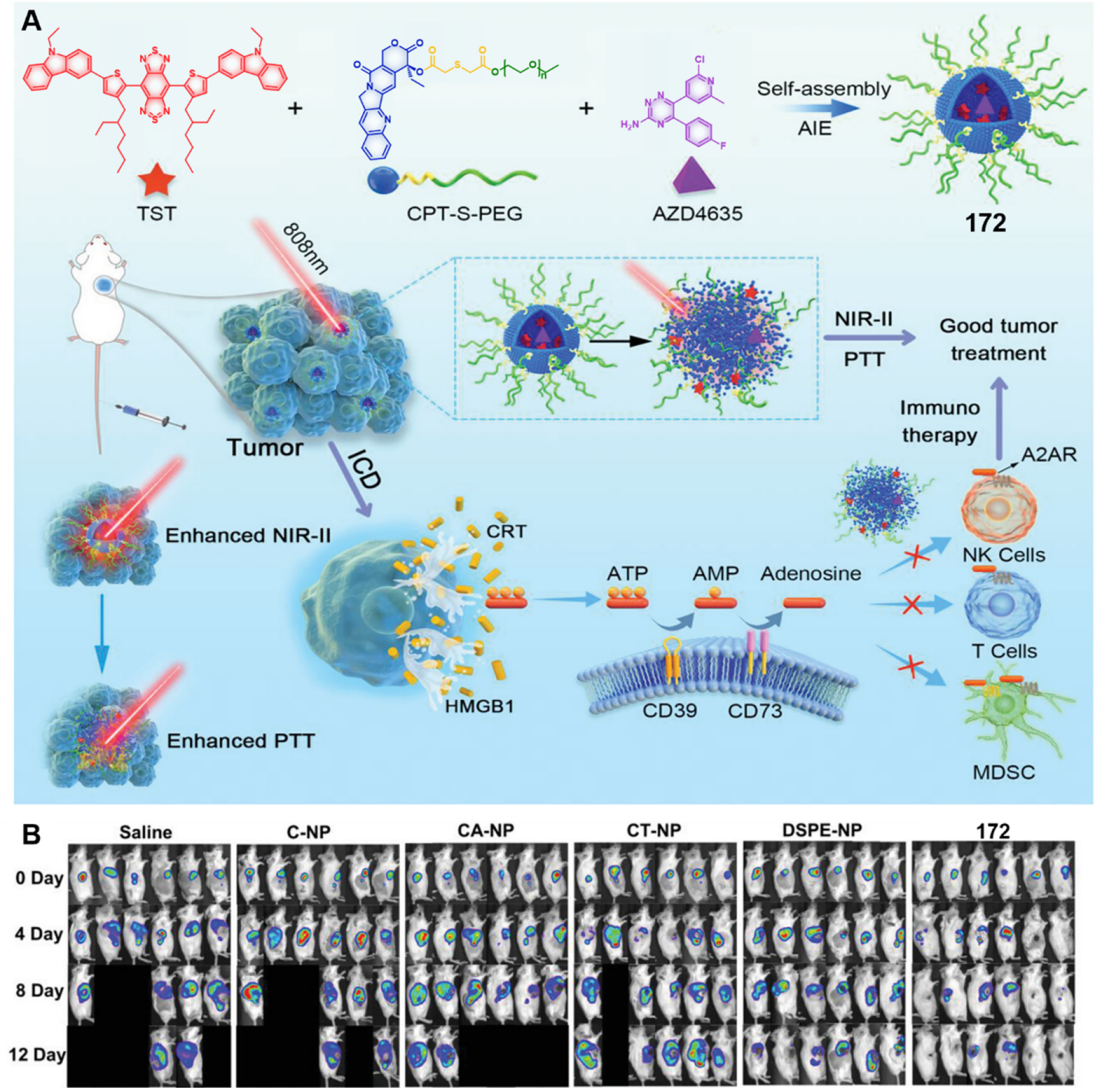
(A) Schematic mechanism of **172** for therapy of tumors. (B) Images of alive small animals after intravenous injection of **172** or controls. Reproduced with permission from ref (553). Copyright 2022 Wiley Intersciences.

The high concentrations of H_2_O_2_ in tumor cells provide new approaches for redox-sensitive theranostic probes. Yu et al. employed an AIE molecule TST with near-infrared luminescence and photothermal function to develop nanoparticles **172** by assembling camptothecin prodrug (CPT-S-PEG) and AZD4635 (an immune checkpoint inhibitor).^553^**172** was silent in a normal physiological environment. However, after entering tumor cells, the nanoparticles exhibit an enhanced fluorescence quantum yield of 15.32%, suggesting these nanoparticles can be used for fluorescence imaging. The redox-sensitive group of the pro-drug (CPT-S-PEG) was degraded by H_2_O_2,_ and active CPT was released into the TME. On the other hand, TST generated intense near-infrared luminescence suitable for real-time imaging (Figure 108). Moreover, TST promoted ROS production by PTT. Additionally, AZD4635 counteracted the immunosuppression generated by ICD to improve the efficacy of PTT and immunotherapy. After irradiating with a 808 nm laser for 5 min, the temperature of the nanoparticle system increased from 32.7 to 68.8 °C, suggesting that **172** exhibits good photothermal conversion efficiency. By injection into the mice’s tail vein, a clear NIR-II fluorescence image can be observed in the tumor, which was attributed to the EPR effect of the nanoparticles. Besides, the nanoparticles **172** exhibited good antitumor effect and satisfactory safety in vivo. After treatment with nanoparticles **172** for 12 days, the tumor in the mice disappeared. Although H_2_O_2_-based cancer diagnosis has been combined with chemotherapy, radiotherapy, PDT, SDT, and PTT^518^ to diagnose and treat cancer, there is still a long road ahead to fully realize clinical applications for these systems.

**Figure 109:**
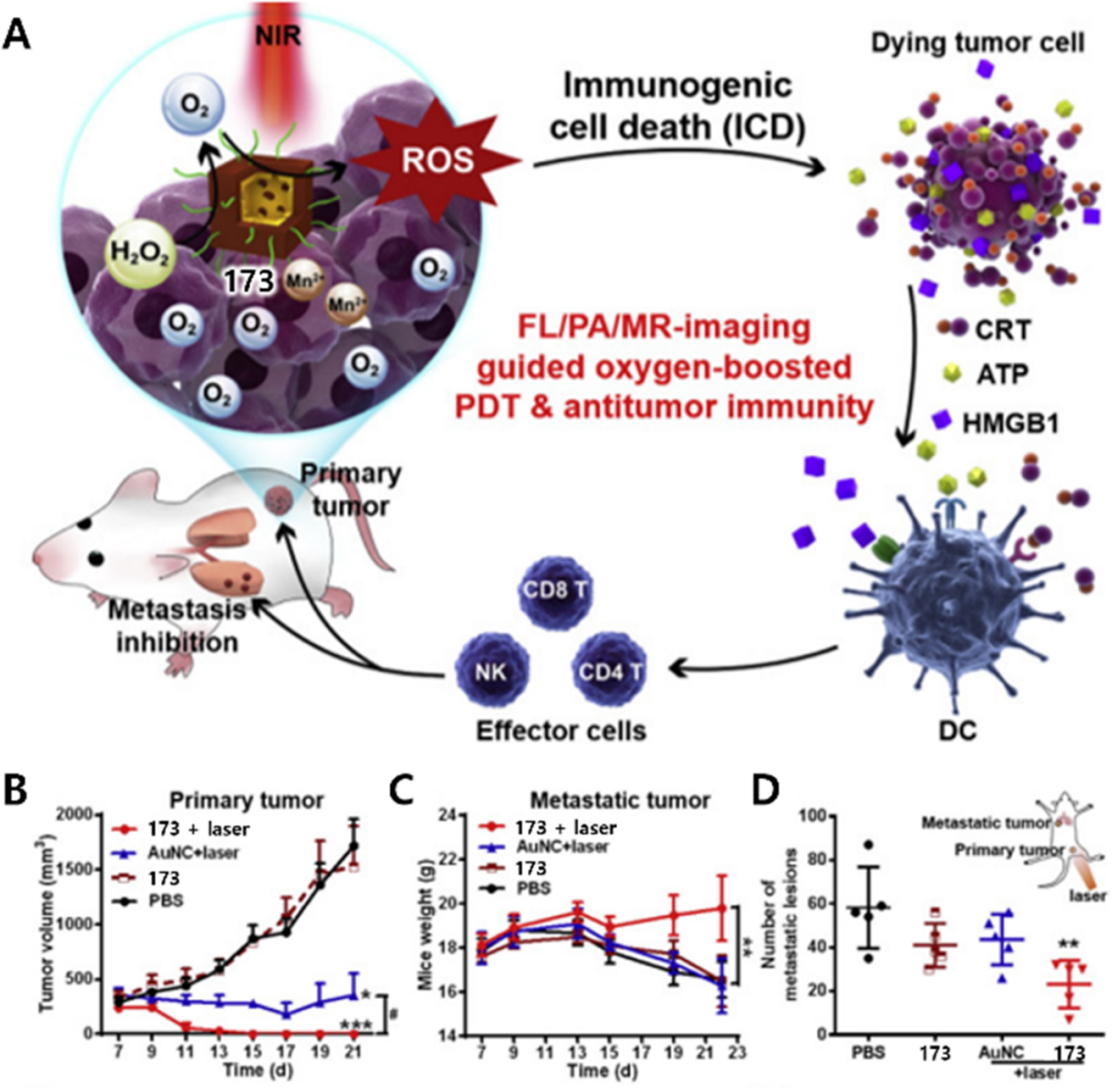
(A) The therapeutic mechanism of **173** (AuNC@MnO_2_) for therapy. (B) Tumor growth curves of different treated groups. (C) Mice weight of lung metastasis experiment monitored after PDT treatment. (D) The number of metastatic lesions counted from excised lungs. Reproduced with permission from ref (554). Copyright 2018 Elsevier Ltd.

Cai et al. prepared core–shell nanoparticles AuNC@MnO_2_ (AM, **173**) by coating gold nanocages with MnO_2_.^554^ The nanoparticles **173** as a PS displayed a good PDT effect under NIR irradiation (Figure 109). The MnO_2_ shell of AM can be decomposed in acidic and H_2_O_2_-rich circumstances of solid tumors to in situ produce oxygen and Mn^2+^, which can improve the oxygenation of TME and endow the nanoparticles 173 with good photoacoustic (PA)/magnetic resonance (MR)/fluorescence (FL) multimodal bioimaging ability. The oxygen can be converted to powerful ROS that can not only kill tumor cells but also elicit an immunogenic cell death (ICD)-mediated antitumor immune response. Under laser irradiation (808 nm), AM can generate enough ^1^O_2_ for PDT to induce ICD. For immunotherapy, AM can stimulate, recruit, and activate T cells to improve efficacy. After intravenous injection, the AM + laser treatment group showed the complete elimination of tumors within 15 days, and lung metastatic nodules from this group were also significantly fewer as compared to control groups.

#### 2.5.3 Hypoxia-Activatable Theranostic Fluorescent Probes in Immunotherapy

Hypoxia is an intrinsic character of the TME.^555^ To date, the effect of hypoxia on cancer therapy has been extensively studied.^556^ It has been shown that uncontrolled cell proliferation of tumor cells leads to hypoxia in the TME. This effect is ascribed to the malignant proliferation outgrowing its oxygen supply. On the other hand, this rapid proliferation promotes abnormal growth of new blood vessels, resulting in sluggish blood flow, which also contributes to hypoxia in the TME.^557^ Generally, the oxygen content in normal physiological environments ranges from 2% to 9%, while it decreases sharply to less than 2% in parts of the TME. Indeed, hypoxia is toxic to both normal and cancer cells. However, tumor cells can survive under hypoxia conditions due to genetic and adaptive changes.^558^ For immunotherapy, hypoxia plays some important roles: (1) it induces T lymphocyte apoptosis;^559^ (2) it weakens the effect of natural killer and natural killer T cells;^560^ (3) it guides programmed dendritic cell death;^561^ (4) it induces immunosuppressive cells contributing to immune tolerance.^562,563^ Therefore, hypoxia largely limits therapeutic efficacy. To improve the efficacy of immunotherapy, strategies have been reported to alleviate hypoxia,^564^ such as the direct delivery of O_2_ into the tumor, and the in situ O_2_ generation in the TME. Additionally, hypoxia-responsive targeted drug delivery strategies have been developed. To achieve diagnosis and treatment, hypoxia-activated theranostic probes are the subject of intensive research. Researchers developed hypoxia-activated theranostic probes based on Tirapazamine, TH302, PR104A, AQ4N, etc.

**Figure 110:**
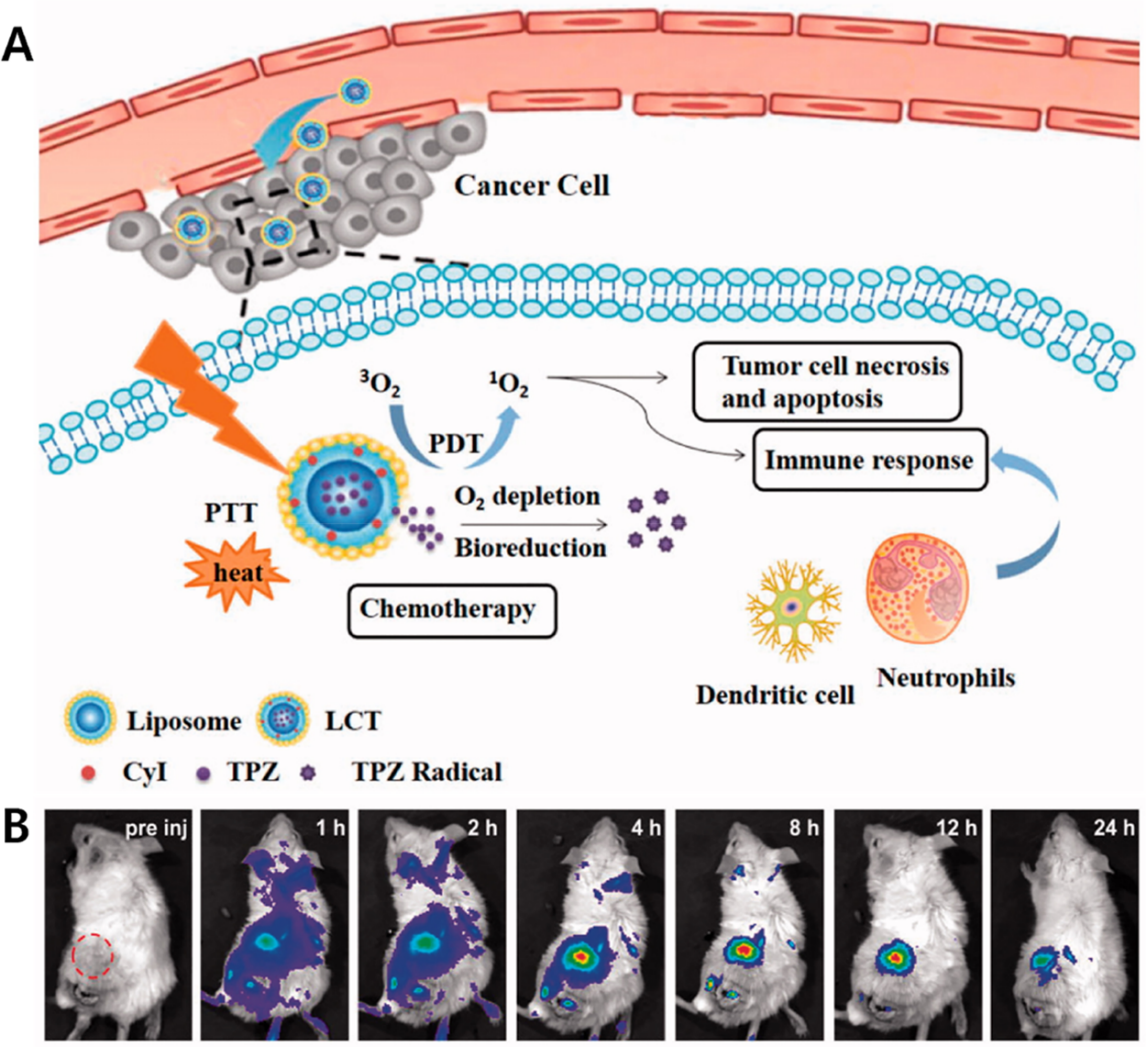
(A) Scheme of **174** for highly efficient synergistic PDT/PTT/immunotherapy combined with hypoxia-activated chemotherapy. (B) In vivo dynamics of **174** in tumor-bearing mice. Reproduced with permission from ref (444). Copyright 2022 Informa UK Limited, trading as Taylor & Francis Group.

Tirapazamine (TPZ) is a bio-reductively activated drug that can specifically induce cell death under hypoxia conditions. Cao et al. developed a hypoxia-activated theranostic probe **174** by co-loading liposome with the iodinated cyanine dye Cyl and TPZ.^444^ After intravenous injection, **174** specifically accumulates in the tumor. Under near-infrared laser irradiation (808 nm), Cyl in **174** can generate ROS for PDT and heat for PTT (Figure 110). Furthermore, Cyl emits NIR light, enabling tumor imaging. Moreover, PDT could stimulate the polar immune response, which recruits and activates CD4 helper T cells and CD8 cytotoxic T cells to promote the secretion of IFN-c, TNF-a, GM-CSF, and other cytokines for immunotherapy. Meanwhile, PDT consumes oxygen and this results in exacerbated hypoxia, aiding TPZ activation to selectively kill tumor cells. 1 h post-injection, fluorescence signals appeared at the tumor sites and the fluorescence was maintained for up to 24 h, suggesting LCT has a good imaging capability. After 21 days of treatment, the tumor volume of the **174** + laser treatment group was the smallest, and a good effect on the distal tumor inhibition was observed. This work developed a good combination strategy of PDT/PTT/CT/immunotherapy.

AQ4N can be specifically activated under hypoxic or anoxic environments. By virtue of this feature, AQ4N in combination with PDT and immunotherapy might be a good approach to improve tumor treatment efficacy. Yoon et al. developed a theranostic probe by combining the water-soluble phthalocyanine derivative PcN4 with AQ4N to improve PDT and immunotherapy.^565^ PcN4 can specifically bind with albumin in vivo to form supramolecular complexes, which endows it with excellent tumor-targeting ability, fluorescence imaging, and PDT antitumor activity. Moreover, PDT induces hypoxia enhancement in a tumor, and AQ4N activation is concomitantly increased in the tumor, which further improves the antitumor activity. After activation under hypoxic conditions, PDT also stimulates ROS production to induce immune checkpoint blockade (ICB) effect. As a consequence, tumor-bearing mice exhibit enhanced immunity under 655 nm laser irradiation. Importantly, the maximum capacity for systemic cancer-specific adaptive immune activation can be achieved by the combination of immune checkpoint blockade therapy with activated PcN4, which enables efficient abscopal responses and enhances anti-metastatic effects.

## 3 Theranostic Fluorescence Probes in Other Diseases

### 3.1 Alzheimer’s Disease

Apart from cancers, theranostic fluorescence probes have emerged as a promising tool for the early diagnosis and targeted therapy of other diseases like Alzheimer’s disease (AD). AD is a progressive neurodegenerative disorder that affects the brain’s ability to function properly.^566,567^ It is the most common form of dementia, accounting for about 60–80% of cases. The disease is characterized by the accumulation of the pathogenic β-amyloid (Aβ) plaques and tau protein tangles in the brain, which disrupts the normal functioning of neurons and eventually leads to their death. This results in cognitive decline and memory loss, as well as other symptoms such as mood changes, disorientation, and difficulty with language and communication. There is currently no cure for AD, and treatment options are limited.^568^ However, there are medications available that can help alleviate some of the symptoms and slow down the progression of the disease.^569^ Theranostic fluorescence probes hold great promise for the early diagnosis and targeted therapy of Alzheimer’s disease.

In principle, to obtain an effective theranostic fluorescence probe for AD, several key factors need to be considered, for example (1) the probes should have suitable lipophilicity and molecular weight to facilitate the blood–brain barrier (BBB) permeability; (2) the interactions between probes and the Aβ or Tau fibrillar proteins, e.g., π–π donor-acceptor interactions and hydrogen bonding, should be strong enough to avoid the competitive binding with other unspecific biocomponents; (3) the probes should have sufficient intermolecular interactions with specific amino acid residues (e.g., Aβ_42_) of Aβ or Tau fibril to interfere the fibril formation; (4) ideal biocompatibility and minimal toxicity are also desired. However, simultaneously integrating such important properties into a single molecule remains a challenge.

**Figure 111:**
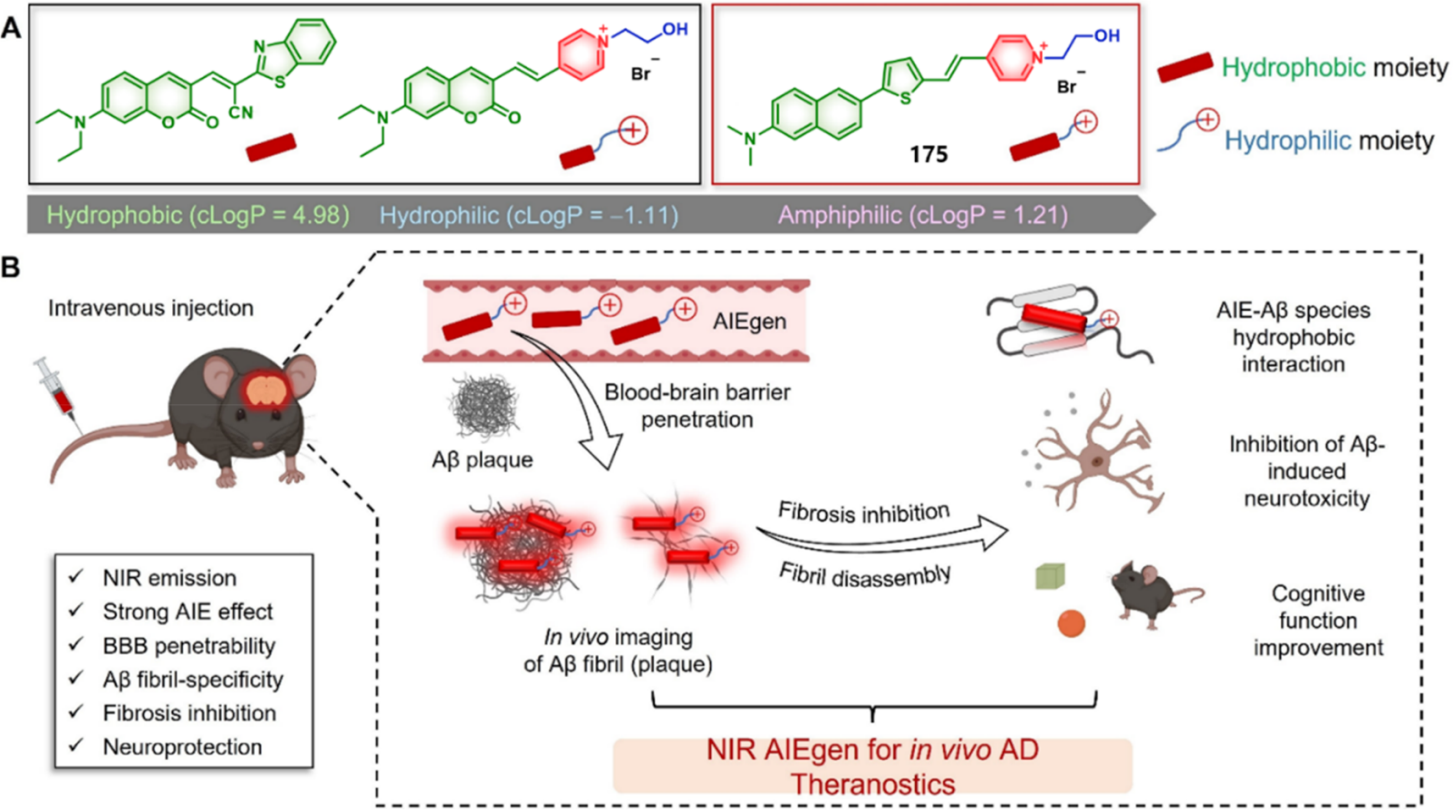
(A) Design and structure of Aβ-targeted NIR aggregation-induced emission (AIE) probe (**175**). (B) Theranostic mechanism of **175** for in vitro diagnostic imaging and inhibition of the formation of the Aβ protein. Reproduced with permission from ref (570). Copyright 2022 Wiley Intersciences.

In 2022, Tang et al. reported an Aβ-targeted aggregation-induced emission (AIE) theranostic probe for effective attenuation of Aβ-induced neurotoxicity in vivo.^570^ As depicted in Figure 111, benefiting from the balanced hydrophobicity–hydrophilicity molecular design strategy, probe **175** obtained an ideal lipophilicity (LogP = 1.21), enabling **175** to bypass the BBB and was capable of specifically recognizing and binding to the cavities of Aβ_42_ fibrils. Theoretical results showed that **175** exerted a strong disruptive effect on Aβ_42_ species through hydrophobic interaction and π–π stacking interactions. The half maximal inhibitory concentration of **175** on fibril Aβ activity was only 0.19 μM, much lower than that of reported Aβ inhibitors.^571^ Impressively, due to the merits of the AIE effect, aggregated **175** in the Aβ plaques exhibited intense NIR fluorescence signals, as a consequence allowing for imaging-guided early diagnosis of AD in a high signal-to-background manner. Remarkably, after treating APP/PS1 transgenic mice (i.e., AD mouse model) with **175** 6 times via intravenous injection, the AD mice exhibited obvious reduced Aβ plaques in the brain and rescued deficits in learning and memory recovery, indicating that **175** could serve as a theranostic fluorescence probe for real-time NIR imaging of Aβ plaques and AD therapy simultaneously.

**Figure 112:**
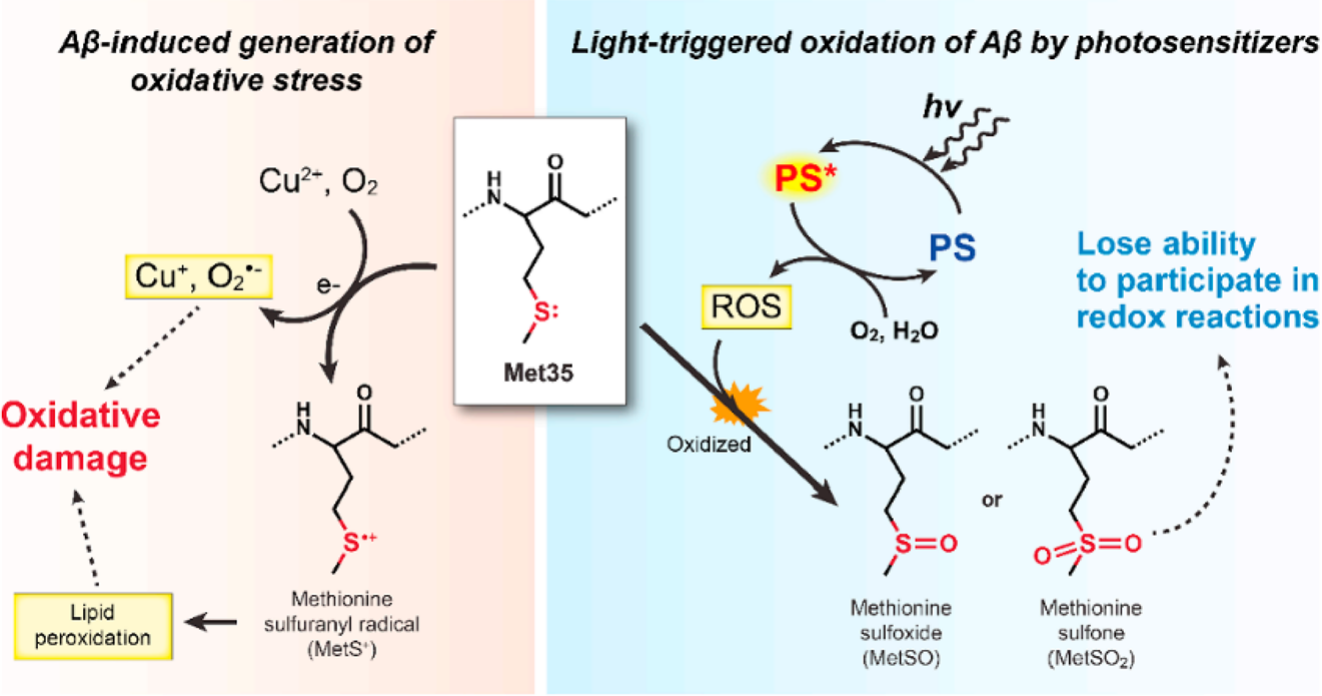
Schematic illustration of the light-triggered photo-oxidation of Aβ protein by PS. The oxidation of Met^35^ residue by PS takes away the ability of Aβ peptides to participate in the redox reactions, as a consequence leading to the aggregative propensity of amyloids decreased. Reproduced with permission from ref (572). Copyright 2018 Elsevier Ltd.

As mentioned above, PDT-based theranostics have shown great promise in cancer treatment through the mechanism of ROS-mediated biomolecular photo-oxidation. Actually, this strategy has also been used for Aβ plaque destruction. Upon photoirradiation, PS can generate ROS to oxidize certain amino acid residues of Aβ peptides, e.g., Met and His, leading to Aβ conformational changes and finally decreasing the aggregative propensity of the pathogenic amyloids (Figure 112). However, for photodynamic AD therapy to be used in a multicomponent system such as the brain, the rational design of AD-specific phototheranostics remains challenging.

**Figure 113:**
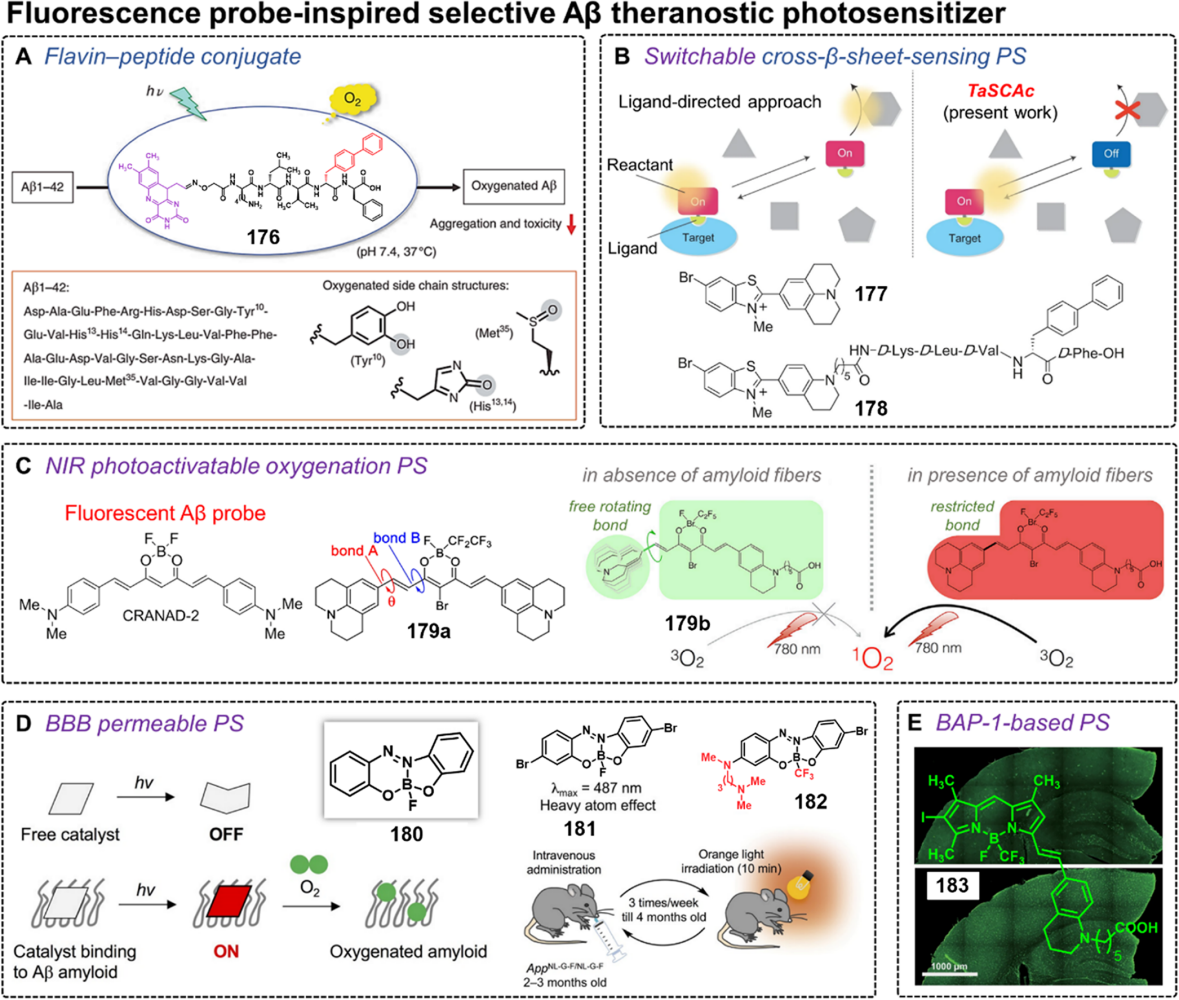
Fluorescence probe inspired theranostic PS design for selective Aβ photo-oxidation. (A) Aβ-binding flavin-peptide conjugate (**176**) for photo-oxidation of the Tyr,^10^ Met,^35^ His,^13^ and His^14^ residues of Aβ_1–42_ fibrils. Reproduced with permission from ref (573). Copyright 2014 Wiley Intersciences. KGaA, Weinheim. (B) Schematic illustration of a new phototheranostic PS design concept, i.e., TaSCAc, which exhibits “OFF–ON” PDT activities for selective oxidation of Aβ proteins. By conjugating with an Aβ-targeting peptide (i.e., modifying **177** to **178**), the treatment selectivity can be further improved. Reproduced with permission from ref (574). Copyright 2016 Springer Nature. (C) Fluorescent Aβ probe (CRANAD-2)-inspired selective phototheranostic **179a** and its mechanism of action. Adapted with permission from refs (575, 576). Copyright 2018 Elsevier Ltd. and Copyright 2018 Elsevier Ltd. (D) BBB permeable PS (**182**) inspired from the probe **180**. Reproduced with permission from ref (577). Copyright 2021 AAAS. (E) BAP-1-based phototheranostic (**P183**) for the clearance of aggregated Aβ in the brain of AD mice. Reproduced with permission from ref (578). Copyright 2021 Oxford University Press. Reproduced with the permission from ref (579). Copyright 2022 Elsevier Ltd.

At present, approaches to realize this goal are mainly concentrated on the ligand-assisted direction of PS. As shown in Figure 113, after conjugating a flavin-based PS to an Aβ-binding peptide, the resulting flavin-peptide conjugate (i.e., **176**) greatly improved the photo-oxidation selectivity of Aβ proteins in living cells compared to free flavin.^573^ The intrinsic fluorescence emission properties of PS in principle can be used to indicate the AD area, as such realizing phototheranostic simultaneously. However, this approach suffers from its inherent shortcomings, that is, the targeting efficiency depends highly on the expression level of pathological Aβ protein. If AD is only in its early stages (that is, low Aβ plaque formation), such ligand-assisted targeting strategy may lose its capability and cause off-targeted photodamage to normal brain tissues.

To address this, a new design concept for AD-targeted phototheranostic, termed targeting sensing catalyst activation (TaSCAc), hass emerged, as depicted in Figure 113.^574^ In this system, the PDT activity of PS (such as **177**) initially remains silent, which means it cannot generate ROS even under the condition of photoirradiation (i.e., “OFF” state); however, once sensing and binding to specific Aβ structures (e.g., the cross-β-sheet quaternary), PS becomes active and is able to photo-oxidize the Aβ proteins (i.e., “ON” state). With this mechanism of action, undesirable off-target and nonspecific photoactivation is therefore inhibited. Aβ-sensing fluorescence probe may emerge as feasible tools for designing such novel PS.

For example, by introducing a bromine atom into the chemical structure of fluorescence Aβ probe CRANAD-2, the resulting compound **179a** became a switchable PS (Figure 113).^575,576^ In **179b**, the TICT effect, ascribed to the bond rotation (e.g., bond A and bond B), significantly inhibits the intersystem crossing process, leading to negligible ROS generation. However, when binding to the Aβ fibers, the free bond rotation would be restricted, interfering with the formation of the TICT effect and in turn generating ROS under photoirradiation. Based on this switching mechanism, the further optimized **179b** selectively photo-oxidizes the aberrant Aβ aggregates in vitro, in cello, and in AD mice brain lysates via the generated ^1^O_2_, even in the presence of multiple off-target substrates. This strategy is also applicable to other TaSCAc systems, such as the BBB permeable compound **182** (inspired by probe **180**, Figure 113).^577^ Moreover, by modifying the BODIPY structure using an iodine atom, an Aβ fluorescence probe BAP-1-derivative **183** was obtained, achieving effective photoclearance of Aβ depositions in the brain of 7-month-old *App*^*NL–G–F/NL–G–F*^ AD mice (Figure 113).^579^

**Figure 114:**
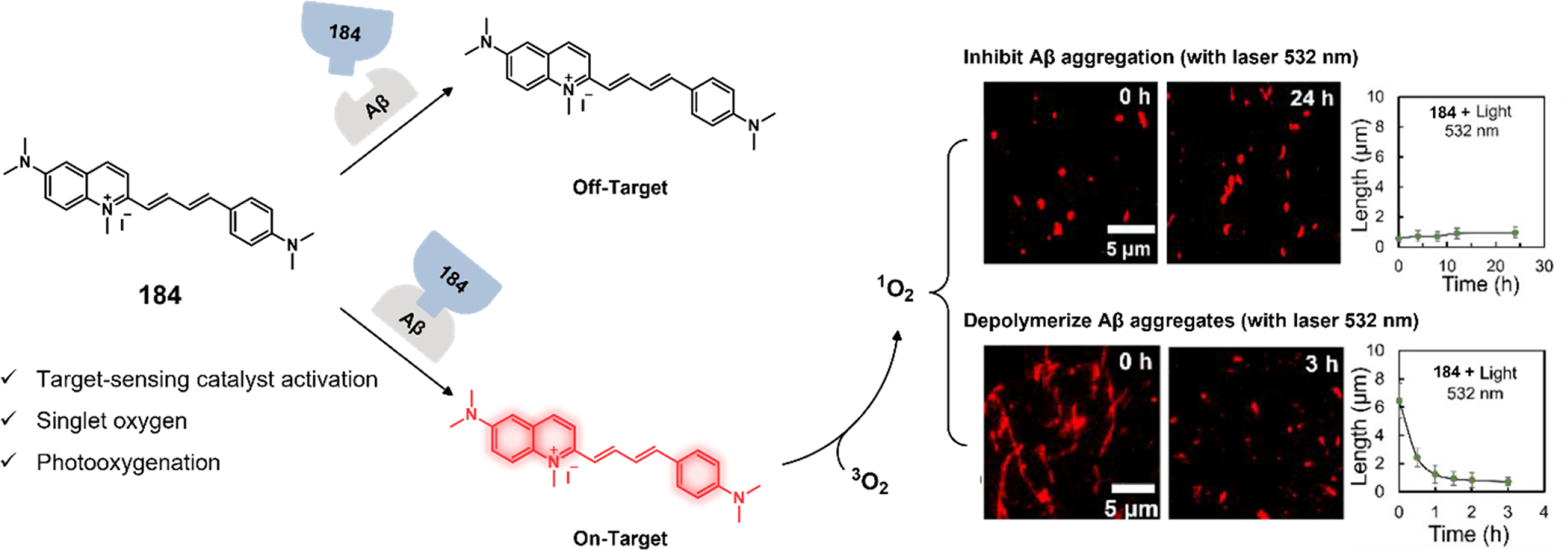
NIR phototheranostic **184** for specific fluorescent mapping and photodynamic oxygenation of Aβ aggregates. Reproduced with permission from ref (580). Copyright 2022 American Chemical Society.

Overall, these impressive studies shed new light on the development of accurate photodynamic AD treatments. Using such an approach, ROS formation can be confined specifically to the AD regions, thus significantly reducing untoward photodamage of surrounding normal tissues. In particular, if such a TaSCAc system also has a fluorescence emission property, concomitant fluorescent “Turn-On” signals can be further applied for AD diagnosis, thereby affording simultaneous AD diagnosis and therapy. As depicted in Figure 114, a TaSCAc system **184** was designed to acheive this goal.^580^ The fluorescence emission of **184** was initially silent due to the TICT effect, while the probe exhibited “Turn-On” fluorescence when bonded to Aβ aggregates, which therefore enabled an in situ mapping of Aβ aggregation. Importantly, this process occurred along with enhanced ^1^O_2_ generation, which then caused a potent photo-oxidation of Aβ aggregates and reduced their neurotoxicity in PC12 cells.

**Figure 115:**
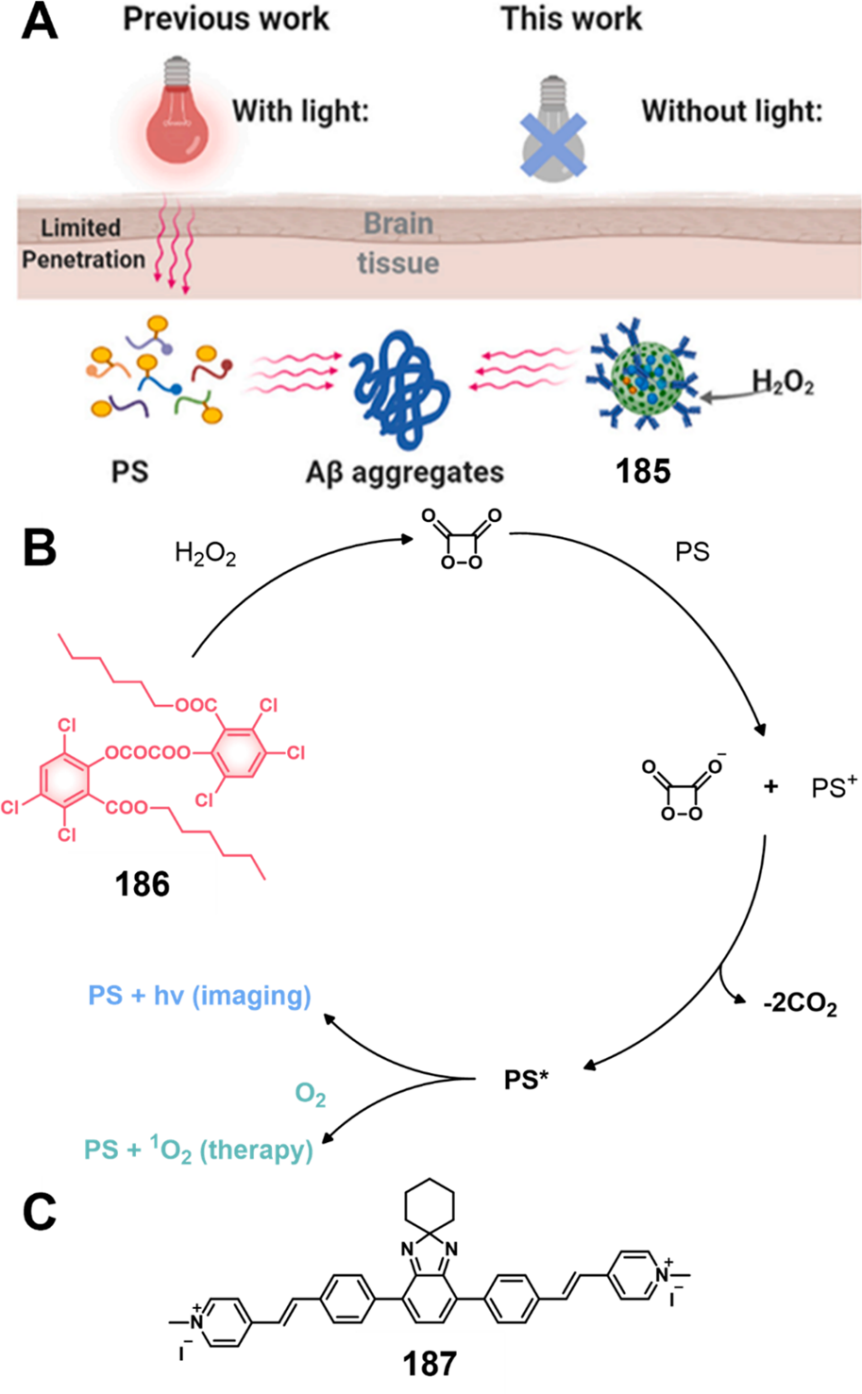
(A) Schematic illustration of chemiluminescence-mediated photo-oxidation of Aβ proteins. (B) Mechanism of action of chemiluminescence-triggered PS activation for ROS generation. (C) Chemical structure of **187**. Reproduced with the permission from ref (581). Copyright 2022 Elsevier Ltd.

Although PDT has been widely adopted as a noninvasive modality with the merit of remote control for disease treatments, the low penetration depth of light in tissues remains an intractable issue.^582^ To address this barrier, developing NIR PSs within the “phototherapeutic window" (650–900 nm)^583^ represents a facile avenue, using PSs such as phthalocyanine,^584^ BODIPY,^585,586^ cyanine,^587^ Nile blue derivates,^588−591^ and upconversion-based small molecules.^592,593^ As an alternative, designing self-luminescent systems might be another applicable strategy to overcome the light source-caused limited penetration depth in vivo. Very recently, Yan et al. reported a chemiluminescence-excited phototherapeutic nanosystem (**185**) capable of photo-oxidizing aggregated Aβ without the need for an external excitation source.^581^ As can be seen in Figure 115, **185** was prepared by loading **187** and **186** in mesoporous silica nanoparticles and then decorated with lactoferrin (Lf). After reaction with the H_2_O_2_ in the tumor microenvironment, **186** could generate a high energy intermediate 1,2-dioxa cyclic dione, which was able to produce chemiluminescence to excite **187**. Activated **187** subsequently emits fluorescence for imaging and sensitize surrounding oxygen to form ^1^O_2_, photo-oxidizing Aβ aggregates and thereby suppressing their neurotoxicity. Based on this study, it is concluded that such an in situ-triggered chemiluminescence might be also applicable to other neurologic diseases.

### 3.2 Senescence

Senescence refers to the biological process of aging in living organisms, which is characterized by a decline in various physiological functions and an increased susceptibility to disease and death.^594,595^ This process is believed to be influenced by both genetic and environmental factors. In humans and other animals, senescence is associated with a range of age-related diseases such as cancer, neurodegeneration, cardiovascular diseases, and metabolic disorders.^596−598^ Since pioneering research in 2011 revealed that the genetic removal of P16-positive senescent cells could significantly prolong the lifespan of mice, the development of interventions that can delay or prevent age-related diseases has become a research frontier.^599^ Nowadays, numerous approaches have been developed, aiming at selectively eliminating senescent cells without causing untoward effects on normal tissues or cells.^600,601^

In the context of senescence, theranostics can be used to develop diagnostic tools that can detect the presence of senescent cells and evaluate the effectiveness of senolytic therapies.^600,602^ One potential theranostic approach is the use of molecular imaging techniques, such as positron emission tomography (PET) and magnetic resonance imaging (MRI), to visualize the presence of senescent cells in vivo. These techniques rely on the use of imaging agents that can specifically bind to senescent cells and provide information about their location and quantity.^602^ Another potential theranostic approach is the use of biomarkers that are overexpressed specifically in senescent cells.^595^ Biomarkers are measurable indicators that can be used to monitor biological processes or disease states. In the context of senescence, potential biomarkers include markers of cellular senescence, such as p16INK4a and senescence-associated beta-galactosidase (SA-β-gal), as well as markers of inflammation and tissue damage.^603−605^ By combining these diagnostic tools with senolytic therapies, theranostics can provide a powerful approach for the precise targeting and elimination of senescent cells.

**Figure 116:**
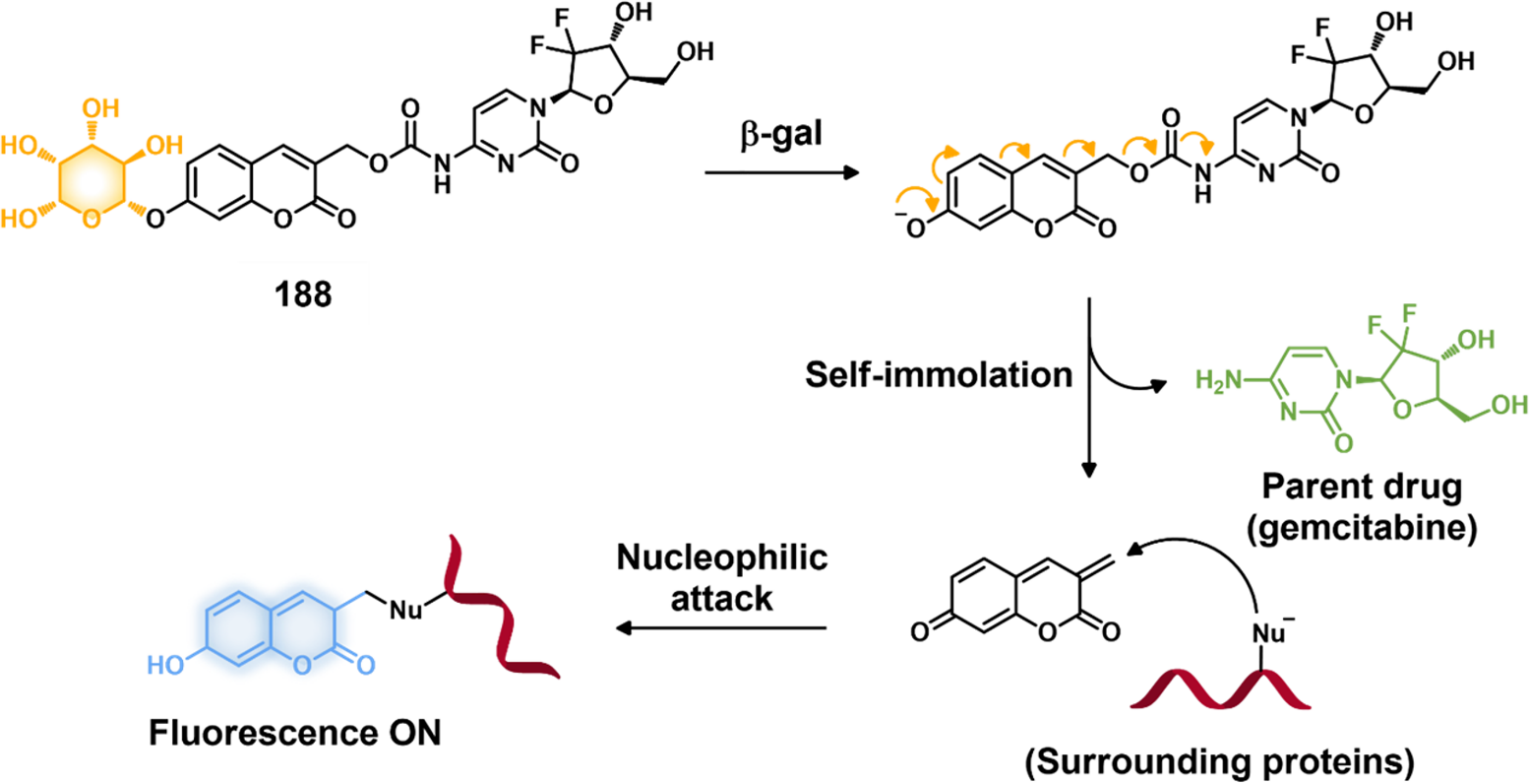
Molecular structure of **TSPD** and corresponding mechanism of action of SA-β-gal-triggered parent drug release for senescent cell elimination as well as fluorescence recovery for senescence diagnosis. Reproduced with permission from ref (606). Copyright 2022 The Author(s).

For example, Li et al. developed a senolysis-based fluorescence theranostic prodrug (**188**, Figure 116) for chronic renal failure.^606^ After exposure to intracellular overexpressed SA-β-gal, **188** could be specifically activated, sequentially releasing the parent drug gemcitabine to eliminate SA-β-gal-enriched senescent cells and the coumarin fluorophore for fluorescence tracking of the senescent cells. Interestingly, the authors incorporated a novel design that uses the generated coumarin intermediate (i.e., MBP) to bio-orthogonally bind to the nucleophilic residue of cellular proteins, endowing **188** with the ability to recognize senescence with single-cell resolution. In vivo experiments evidenced that in mice with chronic renal failure, abdominal administration of **188** remarkably attenuated the degree of kidney injury and improved kidney functions. This approach could ultimately lead to the development of more effective and personalized therapies for age-related diseases and disorders. However, more research is needed to fully develop and optimize senescence theranostics for clinical use.

**Figure 117:**
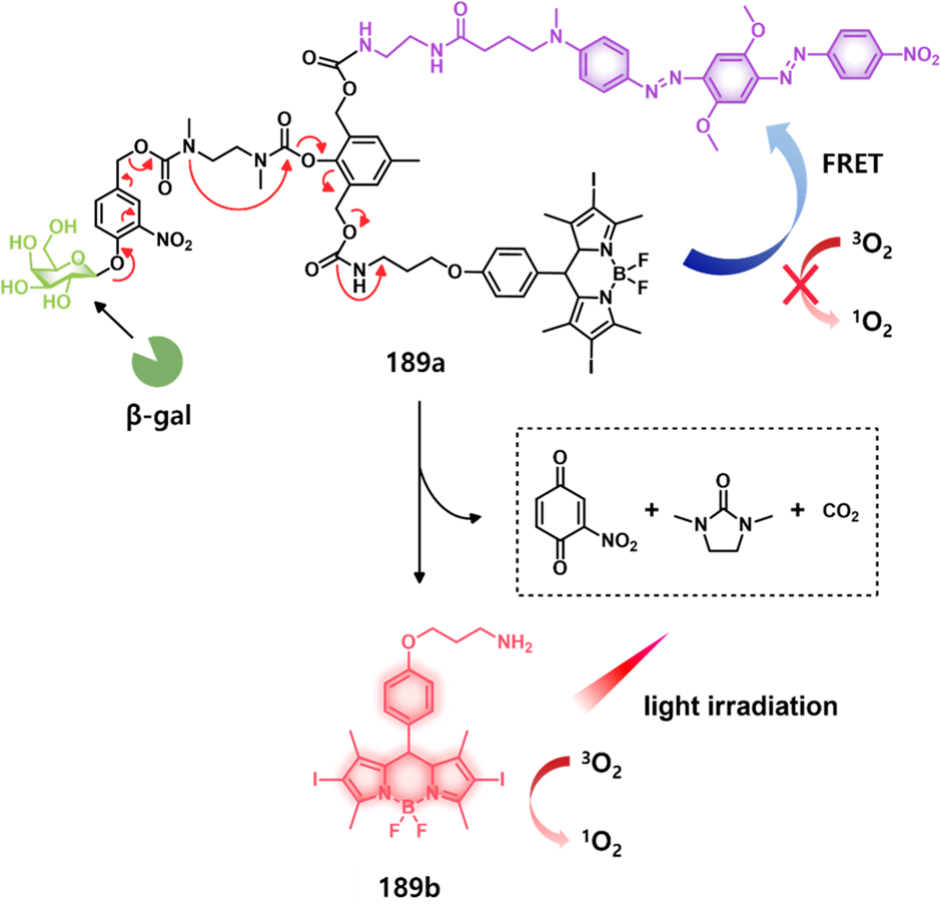
Molecular structure of **189a** for SA-β-gal-triggered photodynamic senescent cell eradication and corresponding self-immolation mechanism for the release of active **189b**. Reproduced with permission from ref (607). Copyright 2023 The Royal Society of Chemistry.

Several studies have also investigated the use of PDT-based phototheranostics for the elimination of senescent cells in vitro and in vivo. Recently, Dennis K. P. Ng and co-workers reported an SA-β-gal-activated phototherapeutic system **189a**.^607^ As illustrated in Figure 117, the tailor-made compound **189a** consists of an SA-β-gal substrate, a BODIPY-based PS (**189b**), and a black hole quencher (BHQ-2) connected via an AB_2_-type self-immolation linker. In the native form, **189b** stays closely in proximity to BHQ-2, which favors the formation of a Förster resonance energy transfer (FRET) effect and consequently inhibits the fluorescence emission and PDT activity of **189b**. After reacting with SA-β-gal, however, **189a** was dissociated, releasing active **189b** and showing strong fluorescence emission as well as ^1^O_2_ generation. In vitro detection and clearance of senescent cells demonstrated that after being incubated with **189a**, bright green fluorescence could be seen in senescent Hela and HT29 cells but not in nonsenescent cells. Meanwhile, under the conditions of light irradiation (λ > 515 nm, 25.5 mW/cm^2^, 20 min), **189a** generated ^1^O_2_ and potently killed the senescent HeLa cells. These results demonstrated that the photosensitizing capability of **189a** could be selectively activated by β-gal, resulting in specific and efficient photoeradication of senescent cells.

**Figure 118:**
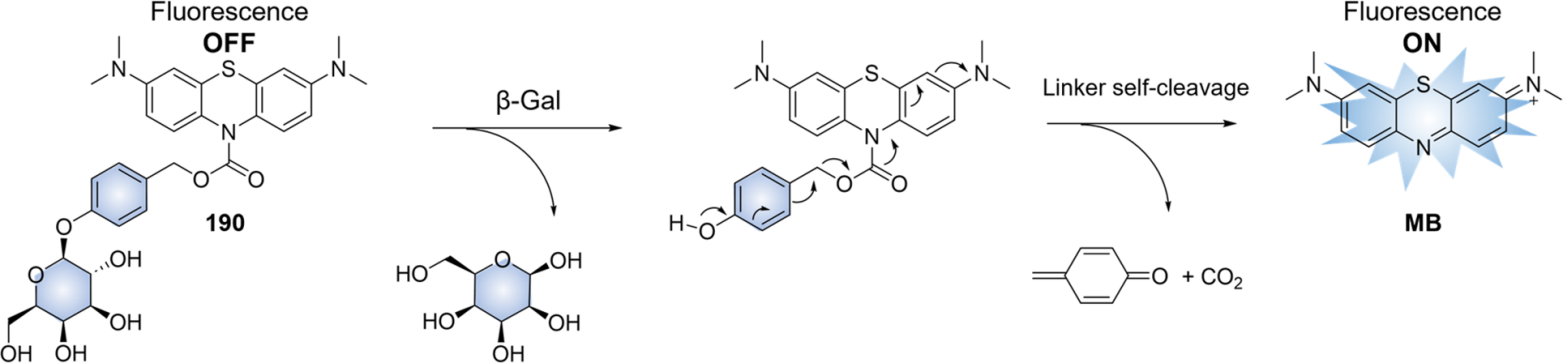
Molecular structure of **190** and corresponding mechanism of action of β-gal-triggered MB release via a self-immolation reaction. Reproduced with permission from ref (608). Copyright 2022 The Royal Society of Chemistry.

Using the same biomarker, Tung et al. reported another SA-β-gal-responsible PDT system (**190**, Figure 118) based on the FDA-approved PS methylene blue (MB).^608^**190** was prepared by integrating a β-gal recognition domain into the 10-N position of the MB skeleton via a self-immolation linker. With this smart design, the conjugation structure of MB was broken, thus completely blocking the absorption, fluorescence, and PDT activities of MB. In the presence of β-gal, the galactopyranoside was cleaved, followed by a self-immolation reaction via the quinone methide elimination to release the original PS MB. Notably, **190** exhibited an ultrahigh sensitivity in detecting β-gal, with a rapid absorption (60-fold increase in 15 min) and fluorescence recovery (95-fold increase in 15 min) in the presence of β-gal. This sensitivity is also applied to the senescent cell diagnosis. Further, under the condition of photoirradiation (665 nm LED light, 30 mW/cm^2^, 30 min), **190** effectively killed the β-gal expressing senescent C6/*LacZ* cells by generating a large amount of ^1^O_2_.

**Figure 119:**
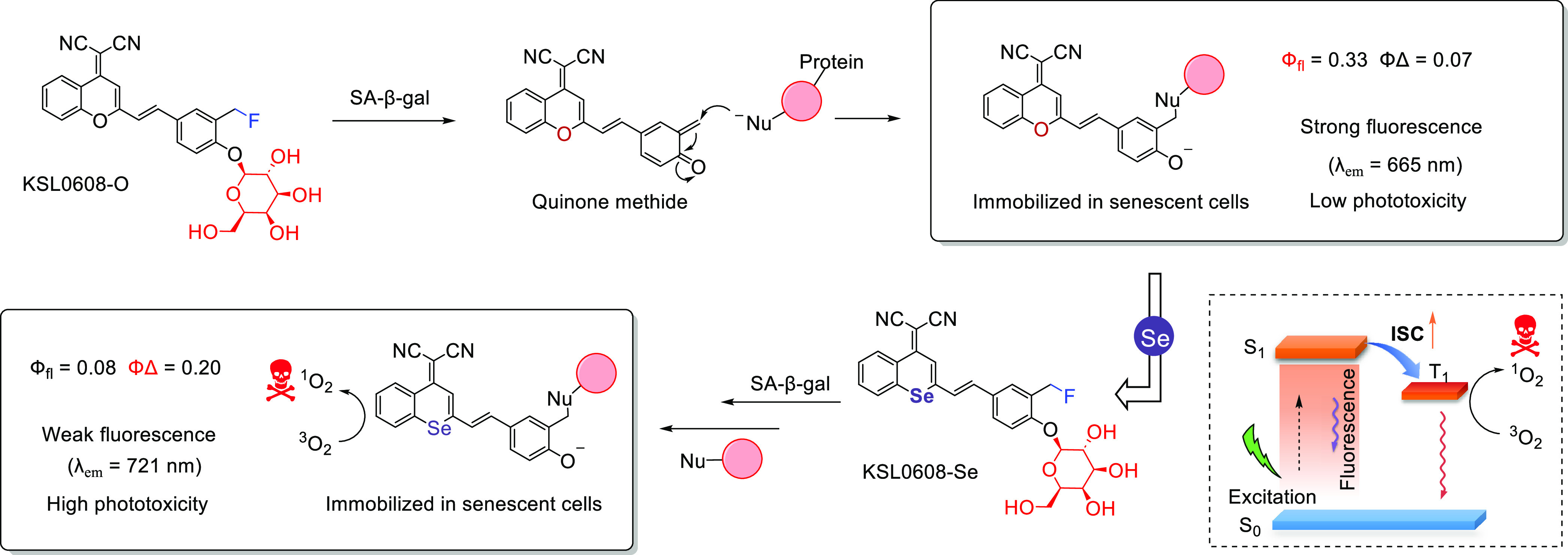
Molecular structure of control compound **191-O** and targeted phototherapeutic agent **191-Se**. Reproduced with permission from ref (402). Copyright 2023 The Author(s), under exclusive license to Springer Nature America, Inc.

These strategies show great promise in terms of phototheranostics for senescence treatments; however, photocontrolled senescence therapy remains in its infancy and few studies have explored their feasibility in vivo. Recently, Li, Guo, and their co-workers proposed a multiplex technology that integrates a SA-β-gal substrate with a fluorescence tag for accurate tracking of senescent cells, a bio-orthogonal receptor triggered by SA-β-gal for targeted-site anchoring of senescent cells with single-cell resolution, and a selenium atom-incorporated PS for achieving selective senescent cell elimination via activatable PDT (Figure 119).^402,609^ A SA-β-gal probe named **191-O** was prepared as a control and a photoactivatable senolytic prodrug named **191-Se** was constructed, wherein the Selenium atom-mediated heavy atom effect^610^ significantly enhanced the intersystem crossing efficiency to promote a high ^1^O_2_ quantum yield (Φ_Δ(191-Se)_ = 0.2 vs Φ_Δ(191-O)_ = 0.07). In the native form, **191-Se** exhibited poor fluorescence and negligible ^1^O_2_ generation. However, after reacting with SA-β-gal, a quinone methide intermediate (i.e., the bio-orthogonal receptor) was generated, which could covalently react with exposed nucleophilic groups on the surfaces of surrounding proteins nearby SA-β-gal, followed by NIR fluorescence signal and PDT “ON”. Importantly, such an “OFF-ON” behavior occurred only after reacting with SA-β-gal. This, therefore, enabled target-site anchoring to and accurate monitoring of senescent cells in a complex system, such as the coculture model of young cells and senescent cells shown in this work. Importantly, in vivo studies using naturally aged mice showed that **191-Se**-mediated PDT decreased the expression of senescence-associated genes and markers, successfully countered age-induced losses in liver and renal function as well as inhibited the age-associated physical dysfunction. This unprecedented integration strategy may provide a new method for the treatment of other diseases, including but not limited to senescence.

### 3.3 Other Diseases

Conventional antimicrobial drugs such as antibiotics, antivirals, antifungals, antiparasitics, etc. have been efficient and successful in many ways in treating microbial infections. However, numerous side-effects and growing resistance to antibiotic drugs are making antimicrobial drugs less effective against microbial infections. To overcome these limitations, new innovative treatment methods are needed, which can effectively fight against infections. Antimicrobial photodynamic therapy (aPDT) is a relatively new treatment approach that uses light and PSs to kill bacteria and other microorganisms. There are several advantages to using an aPDT approach over traditional methods, such as reduced side effects, improved accuracy, and increased efficacy. Smartly designed fluorescent probes can target antibiotic-resistant microbes and selectively kill them upon light irradiation, without harming the noninfected areas. Moreover, fluorescent theragnostic probes can monitor the treatment, by visualizing the infected area until the complete recovery of infection. Recent literature has shown several examples of theranostic fluorescent probes that were successfully applied against microbial infections.^341,611−614^

**Figure 120:**
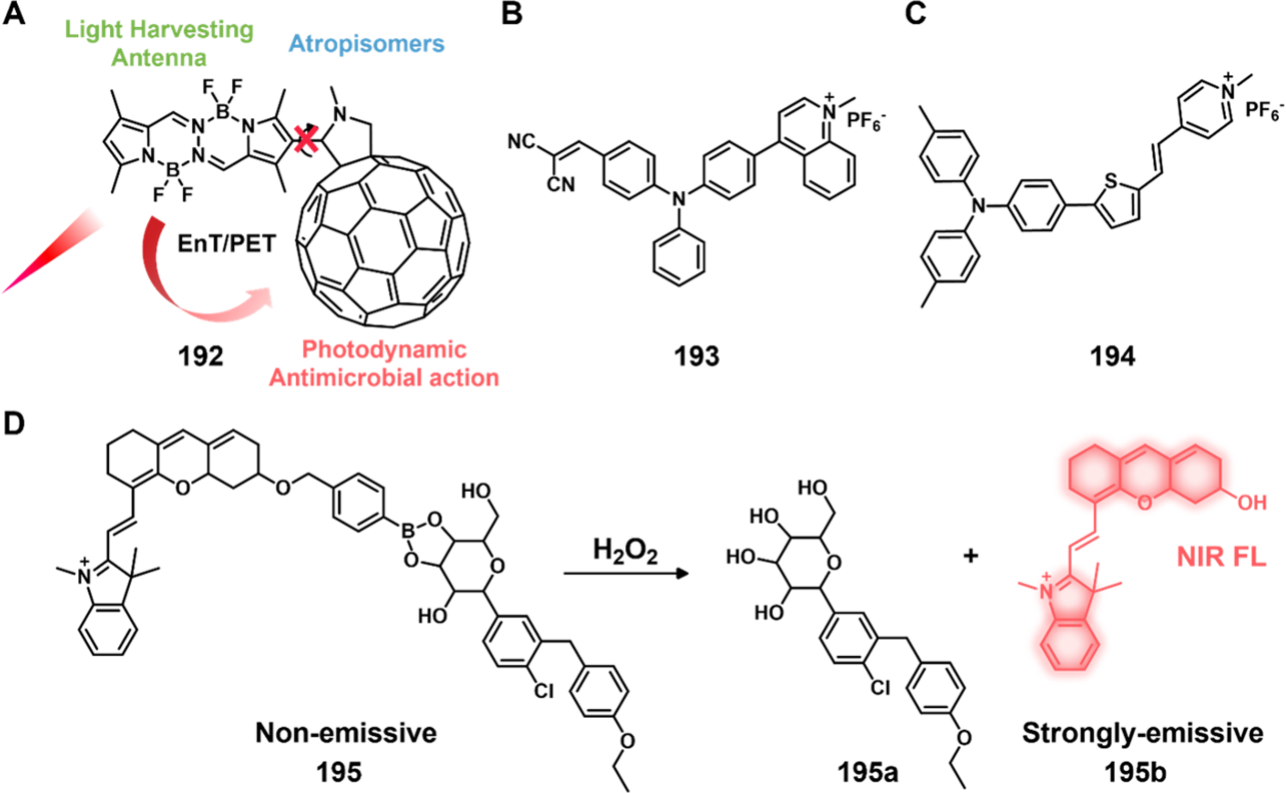
(A) Molecular structures of the theranostic fluorescent probe **192** for antimicrobial PDT. (B) Molecular structures of the theranostic fluorescent probe **193**. (C) Molecular structures of the theranostic fluorescent probe **194**. (D) Structure of the theranostic diad **195** and its response toward H_2_O_2_.

Recently, Lopez et al. designed and synthesized a BOPHY–fullerene C_60_ dyad (BP-C_60_, **192**) by covalently connecting a BOPHY fluorophore with *N*-methylfulleropyrrolidine (Figure 120).^615^ In this probe the BOPHY was utilized as an “antenna” to improve the visible light adsorption, resulting in effectively generated O_2_^•–^ and ^1^O_2_, thus enabling both type I and type II PDT. In *in vitro* experiments, the **192** successfully deactivated *Staphylococcus aureus* by generating efficient ROS under visible light irradiation. However, dyad **192** was not found to be an efficient aPDT agent against *Escherichia coli*. Tang and co-workers reported an asymmetrically cationic AIE PS, CN-TPAQ-PF_6_ (**193**) (Figure 120) that successfully inactivated drug-resistant bacteria with efficient type I and type II ROS generation under irradiation with mild sunlight.^616^ They modified a triphenylamine (TPA) moiety with a quinolinium hexafluorophosphate (PF_6_^–^) and a nitrile group to afford an asymmetric A-D-A type AIEgen theranostic fluorescence probe. The cationization and nitrile introduction enhanced the hydroxyl radical generation ability of the probe to make it 5.4-fold stronger than crystal violet (CV). The cationic charge on **193** bestows an exceptional bacterial recognition and binding ability, proving an excellent fluorescence image-guided aPDT efficacy, even for methicillin-resistant *Staphylococcus aureus* “super bacteria”. Although aPDT is a new and developing area, it is anticipated that theranostic fluorescent probes have the potential to make aPDT a more effective and safer treatment against drug-resistant infections.

Theranostic fluorescent probes can provide a potential solution to several kinds of eye diseases, including diabetic retinopathy, glaucoma, retinoblastoma, and bacterial endophthalmitis. Some of the potential advantages of theranostic probes for eye disease are targeted therapy, selective heat delivery, and a convenient means for monitoring the treatment. Although fluorescent probes are being developed for many eye diseases, theranostic probes are still rare. Here, we will discuss some representative theranostic fluorescence for eye diseases.^617^

Bacterial endophthalmitis (BE) is a common eye disease that can cause complete blindness. It is a bacterial infection that enters the eye through a corneal break or the bloodstream. Antibiotics are the conventional treatment for BE, but multidrug resistance and retinal toxicity pose challenges to successful therapy. Recently, Tang and colleagues reported a cationic aggregation-induced emission luminogen called triphenylamine thiophene pyridinium (TTPy, **194**, Figure 120) for a PDT of BE.^618^ TTPy (**194**) can selectively target and kill bacteria without harming normal ocular tissues. In an in vivo experiment on rats infected with *Staphylococcus aureus*, TTPy (**194**) not only diagnosed and treated BE but also induced an innate immune response to protect the retina from infection. Moreover, TTPy (**194**) was found to be a better theranostic probe than Rose Bengal, indicating its excellent potential for clinical applications in treating ocular infections.

Diabetes is a growing public health concern that can lead to life-threatening chronic diseases, including blindness, heart disease, stroke, kidney failure, and certain cancers.^619^ There are two types of diabetes: type I, which is a genetic condition that appears in early life, and type II, which is often associated with an unhealthy lifestyle. Diabetes is typically diagnosed based on excessive blood glucose levels and the feeling of extreme hunger. However, diabetes symptoms are often underdiagnosed, and significant damage may have already occurred before the confirmation of diabetes, making early diagnosis methods needed for the early stage treatment. While several fluorescence probes have been developed for early diagnosis of diabetes by estimating abnormal levels of chemicals, enzymes, pH labels, and viscosity in cell organelles, the development of theranostic fluorescent probes for early diagnosis and therapy of diabetes is still an evolving field.^620,621^

Under diabetic conditions, hyperglycemia leads to the production of excess ROS through glucose phosphorylation. H_2_O_2_ is one of the major ROS produced in these conditions and is relatively stable. Recent studies have shown that elevated levels of H_2_O_2_ in cells can signal diabetes and other related diseases. Therefore, the development of a theranostic fluorescence probe for real-time detection of elevated H_2_O_2_ can aid in the early diagnosis and timely management of diabetes. To demonstrate this concept, Yu et al. presented an H_2_O_2_-responsive dyad (**DX-B-DA**, **195**, Figure 120) consisting of a near-infrared fluorescent dye (DX) and the type II diabetes drug dapagliflozin (DA), linked through an H_2_O_2_-sensitive linker, for the diagnosis and treatment of type II diabetes. In vitro experiments confirmed that the dyad **195** is initially poorly fluorescent and has no therapeutic effect. However, in the presence of H_2_O_2_, the drug DA and dye DX are released, resulting in bright fluorescence and the initiation of therapy. The authors also successfully demonstrated that dyad **195** can selectively visualize diabetic liver/kidney damage and treat type II diabetes in mouse models.

## 4 Challenges in Clinical Translation

Despite several impressive examples of efficient and potent DDS mentioned above, the field of theranostic small-molecule drug delivery systems has not made significant progress beyond the preclinical drug development stages. Various factors contribute to this state of affairs and are outlined below.

First, while theranostic agents offer clear advantages in research settings by enabling the collection of spatiotemporal information on drug delivery and, in some cases, providing spatiotemporal control over therapeutic actions, their benefits in a clinical setting are sometimes less apparent. Even near-infrared light from the NIR-II window only allows penetration of a few centimeters, which limits their applications given the size of the human body.^622^ However, there are several applications for fluorescent DDS, such as fluorescence-guided surgery and the theranostic treatment of superficial cancerous tissues.

More significant challenges arise from the DMPK (drug metabolism and pharmacokinetics) profile, or rather the lack thereof, of DDS. While most DDS described in this review have demonstrated efficient results in cells and animal tests, they lack comprehensive characterization in terms of pharmacokinetics and metabolism. Small-molecule drugs are typically extensively characterized to determine properties such as solubility, lipophilicity, metabolic stability, plasma protein binding, off-target binding, clearance rate, mechanism, cellular penetration, etc.^623^ However, most DDS have not undergone such thorough characterization. A significant proportion of DDS is currently administered intravenously and enters cells through active (receptor-mediated) uptake, which eliminates some of the uptake challenges often encountered by small molecules. Additionally, several DDS could be applied topically to treat diseases, reducing the need for compounds with sufficient resistance to hepatic metabolism. However, challenges remain for DDS. In the case of theranostic chemotherapy, the properties of the released small-molecule drug are well-described, but the attachment of a linker potentially alters the profile and kinetics of formed metabolites. As a result, the differing clearance rates and mechanisms between (unactivated) DDS and their small-molecule fragments create a complex pharmacokinetic profile, necessitating further in-depth studies.

Similarly, the fluorescent probes used in DDS designs have often not been characterized from a DMPK perspective and may contribute to additional toxicity. As shown in this review, there is a trend for DDS to exhibit fluorescence with red-shifted wavelengths, which requires large, flat, often polycyclic aromatic structures. The strong π–π donor-acceptor interactions between these hydrophobic molecules severely limit solubility. Furthermore, these structures may undergo hepatic metabolism to form arene-oxides, which can be potentially carcinogenic.^624^ Therefore, in the design of the next generation of DDS, it is strongly recommended to study also the properties and potential toxicity of the fluorophores and their metabolites. The hepatic metabolism of molecules is often significantly faster for lipophilic compounds, so choosing a more water-soluble fluorophore would likely be beneficial not only for the overall solubility of DDS but also for their metabolic stability and to avoid off-target binding.^625,626^ As the development of red-shifted biocompatible fluorophores is crucial not only for DDS but for various applications, it is currently a major focus of research, and significant advances in fluorophore design will undoubtedly benefit DDS as well.

Although intravenous administration is most commonly used, DDS amenable to oral uptake could represent a significant breakthrough and facilitate clinical translation. It is evident that the DDS described in this review do not conform to the traditional metrics for small-molecule oral drug design, such as Lipinski’s rule-of-5 (RO5). However, it has become clear that orally available chemical space is not limited to RO5, as evidenced by the emergence of large macrocyclic drugs and proteolysis targeting chimeras (PROTACs). Several new descriptors for the molecular design of larger molecules with a reasonable chance of good oral availability have been described, mainly for macrocycles but with broad applicability. Consensus models have been described, e.g., MW ≤ 1000 Da, −2 ≤ cLogP ≤ 10, HBA ≤ 15, HBD ≤ 6^627−629^ (with MW: molecular weight, cLogP: the calculated pH-independent lipophilicity, HBA: the number of hydrogen bond acceptors, and HBD: the number of hydrogen bond donors). Other descriptors to improve oral availability have been proposed as well, for example, AbbVie’s AB-MPS score for large molecules, in which molecular design is adjusted to keep this score as small as possible^630^ (AB-MPS = Abs(cLogD – 3) + #AR + #RB; with cLogD: the calculated pH-dependent lipophilicity, #AR: the number of aromatic rings, and #RB: the number of rotatable bonds). While it may seem a challenging task to redesign DDS with these parameters in mind, which it undoubtedly is, the evolution of our understanding of what allows for good oral availability is still advancing and the clinical translation of DDS will likely benefit from these insights.

The development history of DDS shares similarities with that of PROTACs, which are also large molecules consisting of multiple components connected through a linker.^631,632^ Initially, PROTAC development faced significant skepticism regarding clinical translation, but the field has made remarkable advances in the past decade, resulting in orally available drugs, some of which can even cross the blood–brain barrier.^633^ Several PROTAC designs have reached Phase II clinical trials, and a first design recently entered Phase III clinical trials.^631,632^ Therefore, we believe that with better DMPK characterization of DDS in general, necessary structural optimizations, the use of fluorophores optimized for biocompatibility, and increased focus on oral availability, the DDS field could experience a rapid rise similar to that of the PROTAC field, potentially leading to widespread clinical applications.

## 5 Conclusion

In this review, we have highlighted recent advances in the design and application of theranostic fluorescent probes. These probes range from small molecules to nanoparticles and have become popular research tools for visualizing and treating tumors. However, their applications are expanding beyond cancer theranostics, with probes being reported in the fields of neurodegenerative diseases, aging, and antibacterials. Therapeutic applications for other indications will also emerge in the future.

The use of theranostic fluorescent probes offers great potential for reducing the systemic toxicity of drugs and providing a means to visualize drug delivery in terms of both spatial and temporal aspects. Some probes are designed to be activated by external stimuli, allowing for precise control over the therapeutic process, such as photodynamic, photothermal, or sonodynamic activation.

Small-molecule tumor-selective theranostic fluorescent probes can be activated in the tumor microenvironment through various mechanisms. These probes are sensitive to factors like low pH, overexpressed (bio)molecules in cancer cells (e.g., GSH), redox imbalances (e.g., H_2_S, ROS), enzymes activated or enhanced under hypoxia (e.g., DT-diaphorase, azo-reductase, nitro-reductase), as well as other enzymes with increased expression levels in cancerous tissues (e.g., esterases, proteases), and combinations thereof. Similarly, a wide range of design strategies have been reported for nanoparticles and nanovesicles. These designs are based on self-assembled structures of drug conjugates and nanoaggregates with biologically compatible materials such as HSA, biopolymers, and biocompatible synthetic polymers.

While there have been hurdles preventing these theranostic fluorescent probes from reaching their expected translation into the clinic, the potential economic benefits are substantial. With a better understanding of the behavior of large molecules as potential drugs and a growing acceptance of nanoparticles in clinical applications, we anticipate widespread clinical use of these probes in the coming decade(s). We believe that a bright future awaits this research domain.

## Abbreviations

AO: acridine orange
ACQ: aggregation-caused quenching
AD: Alzheimer’s disease
ADV: sound droplet evaporation
AEP: asparaginyl endopeptidases
AIEgens: aggregation-induced emission fluorogens
ALP: alkaline phosphatase
aPDT: antimicrobial photodynamic therapy
APN: aminopeptidase *N*
ASGP: asialoglycoprotein
Aβ: β-amyloid
BAC: brominated asymmetric cyanine
BBB: blood brain barrier
BDP: diiododistyrylbodipy
BE: bacterial endophthalmitis
BHQ-3: black hole quencher 3
BL: bioluminescence
BODIPY: 4,4-difluoro-4-bora-3a,4a-diaza-*s*-indacene
BP-C_60_: BOPHY–fullerene C_60_ dyad
CAE: *E*-cinnamic acid
CAIX: carbonic anhydrase IX
CDT: chemodynamic therapy
CESs: carboxylesterases
CLSM: confocal laser scanning microscopy
COX-2: cyclooxygenase-2
CPT: camptothecin
CRC: colorectal cancer
CRET: chemiluminescence resonance energy transfer
Croc: croconaine
CTSB: cathepsin B
CTSL: cysteine cathepsin L
CV: crystal violet
CY7: cyanine7
Cys: cysteine
Dabcyl: 4-(dimethylamino azo)benzene-4-carboxylic acid
DAE: diarylethene
DBS: 2,4-dinitrobenzenesulfonyl
DC: dendritic cell
DCA: dichloroacetic acid
DCM: dicyanomethylene-4H-chromene
DDS: drug delivery system
DMPK: drug metabolism and pharmacokinetics
DMSO: dimethyl sulfoxide
DNBS: 2,4-dinitrobenzenesulfonate
DNP: dinitrophenyl
Dox: doxorubicin
DPNB: deep penetrating nanombomb
DPP: pyrrolopyrrolidone
DSBDP: distyryl boron dipyrromethene
DSPE: 1,2-distearoyl-*sn*-glycero-3-phosphoethanolamine
DTD: DT-diaphorase
EB: ethidium bromide
ECM: extracellular matrix
ENBS: 5-(ethylamino)-9-diethylamino-benzo[a]phenothiazinium chloride
EPR: enhanced permeability and retention
ESI-MS: electrospray ionization mass spectrometry
FAM: 5(6)-carboxylfluorescein
FL: fluorescence
FMN: flavin mononucleotide
FRET: Förster resonance energy transfer
GEM: gemcitabine
GGT: γ-glutamyl transpeptidase
GOx: glucose oxidase
GSH: glutathione
HA: hyaluronic acid
HAase: hyaluronidase
HAS: human serum albumin
HCPT: 10-hydroxycamptothecin
Hcy: homocysteine
HDACs: histone deacetylases
HES: hydroxyethyl starch
HIFU: high-intensity focused ultrasound
HPNA: HA-PpIX nanoassembly
ICD: immunogenic cell death
ICG: indocyanine green
ICI: immune checkpoint inhibitor
ICT: intramolecular charge transfer
iNOS: inducible nitric oxide synthase
LCD: lysosome-mediated cell death
LIFU: low-intensity focused ultrasound
LPS: lipopolysaccharide
MB: methylene blue
MDR: multidrug resistance
MMP-2: metalloproteinase-2
MR: magnetic resonance
MTT: 3-(4,5-dimethylthiazol-2-yl)-2,5-diphenyltetrazolium bromide
NADPH: nicotinamide adenine dinucleotide phosphate
NEBI: *N*-ethoxybenzylimidazole
NIR: near-infrared
NQO1: NAD(P)H quinone oxidoreductase
NSAID: nonsteroidal anti-inflammatory drug
NTR: nitroredutases
OXPHOS: oxidative phosphorylation
PA: photoacoustic
PAE: poly β-amino ester
PBS: phosphate buffered saline
PD-1: programmed cell death receptor 1
PDK: pyruvate dehydrogenase inhibitor
PDL-1: programmed death 1 ligand
PDT: photodynamic therapy
PEG: polyethylene glycol
PEM: pemetrexed
PET: photoinduced electron transfer
PFP: perfluoropentane
PLA: polylactic acid
PLNPs: persistent luminescence nanoparticles
PMB: photodynamic molecular beacon
PPa: pheophorbide A
PROTACs: proteolysis targeting chimeras
PS: PS
PTAs: photothermal agents
PTT: photothermal therapy
PTX: paclitaxel
Rd: rhodamine
RGD: arginylglycylaspartic acid
RNS: reactive nitrogen species
RO5: Lipinski’s rule-of-5
ROS: reactive oxygen species
RP-HPLC: reverse phase-high performance liquid chromatography
RT: radiotherapy
SA-β-gal: senescence-associated beta-galactosidase
SCID: severe combined immunodeficiency
SDT: sonodynamic therapy
SOD: superoxide dismutase
SPECT: single photon emission computed tomography
TaSCAc: termed targeting sensing catalyst activation
TBDMS: *tert*-butyldimethylsilyl
TBDPS: *tert*-butyldiphenylsilyl
TICT: twisted intramolecular charge transfer
TIPS: triisopropylsilyl
TME: tumor microenvironment
TPA: triphenylamine
TPE: tetraphenylethylene
TPGS: *d*-α-tocopheryl polyethylene glycol 1000 succinate
TPP: tetraphenylporphyrin
TPPS: meso-tetrakis(4-sulfonatophenyl) porphyrin
TPZ: tirapazamine
TrxSS: thioredoxin
TYR: tyrosinase
US: ultrasound
β-gal: β-galactosidase
